# Integrative single-cell and bulk transcriptomic analyses identify DRAM1 as a candidate gene from fibroblast-associated transcriptional programs in colorectal cancer

**DOI:** 10.3389/fonc.2026.1862796

**Published:** 2026-06-11

**Authors:** Rudong Li, Zhipeng Zhao, Hengyu Liu, Chao Zhang, Zhicheng Wang, Siyi Wang, Xudong Wang

**Affiliations:** 1 Department of Gastrointestinal Nutrition and Hernia Surgery, The Second Hospital of Jilin University, Changchun, Jilin, China; 2 Department of Orthopedic Surgery, The Second Hospital of Jilin University, Changchun, Jilin, China

**Keywords:** colorectal cancer, DRAM1, fibroblasts, hdWGCNA, single-cell RNA sequencing, tumor microenvironment

## Abstract

**Background:**

Colorectal cancer (CRC) is characterized by marked cellular heterogeneity and dynamic remodeling of the tumor microenvironment. Fibroblasts represent an important stromal component of CRC and may contribute to tumor progression through complex transcriptional programs and intercellular interactions. However, fibroblast-associated candidate genes with potential biological relevance remain incompletely defined.

**Methods:**

We applied an integrative strategy combining publicly available single-cell RNA sequencing (scRNA-seq), pseudotime analysis, CellChat, high-dimensional weighted gene co-expression network analysis (hdWGCNA), bulk transcriptomic validation, machine learning prioritization, and experimental assays. Using the GSE221575 dataset, we mapped the cellular landscape of CRC and adjacent normal tissues and identified fibroblast-associated co-expression modules. Candidate genes were further prioritized across five independent GEO bulk transcriptomic cohorts. DRAM1 was selected for validation by qRT-PCR, western blotting, proliferation, colony formation, wound-healing, Transwell, and xenograft assays.

**Results:**

scRNA-seq analysis identified 17 cell clusters corresponding to 10 major cell lineages in CRC and adjacent normal tissues. Cell-cell communication analysis showed extensive signaling interactions among stromal, epithelial, endothelial, and immune cell populations, with fibroblasts occupying a prominent position in the communication network. hdWGCNA identified 31 fibroblast-associated modules, from which 123 candidate genes were extracted. Integrative bulk validation and machine learning analysis yielded five core genes, including INHBA, COL6A3, SPARC, DRAM1, and COL1A2. Among them, DRAM1 was selected for experimental validation. DRAM1 expression was elevated in CRC tissues and selected CRC cell lines. Loss-of-function assays showed that DRAM1 silencing enhanced CRC cell proliferation, migration, invasion, and xenograft growth, suggesting that increased DRAM1 expression may reflect a compensatory stress-response program rather than a purely oncogenic function.

**Conclusion:**

This study provides an integrative characterization of fibroblast-associated transcriptional programs in CRC and identifies DRAM1 as a candidate gene emerging from these programs. Functional assays support a tumor-restraining role for DRAM1 in CRC epithelial tumor cell models, whereas its direct role in fibroblast-mediated stromal-immune regulation requires further investigation.

## 1 Introduction

Colorectal cancer (CRC) remains a leading cause of cancer-related mortality worldwide (1). Despite substantial advances in screening, surgical management, and systemic therapy, recurrence, metastasis, and treatment resistance remain major clinical challenges, particularly in patients with advanced disease (2). Increasing evidence suggests that these features are shaped not only by tumor-intrinsic alterations but also by dynamic bidirectional interactions between malignant cells and the surrounding tumor microenvironment (TME) (3–5). In recent years, single-cell RNA sequencing (scRNA-seq) has greatly improved our ability to resolve TME heterogeneity and has provided a powerful framework for dissecting the interactions among malignant, stromal, and immune cell populations (6).

Fibroblasts constitute a major stromal component of the CRC microenvironment and exhibit marked phenotypic and functional diversity (7). These cells participate in extracellular matrix remodeling, cytokine and growth factor secretion, angiogenic regulation, and local immune modulation, thereby influencing tumor growth, invasion, and therapeutic response (8, 9). However, despite their recognized importance, the transcriptional heterogeneity of fibroblast subpopulations in CRC and their communication patterns with immune and other microenvironmental cell types remain incompletely understood (10, 11).

To address this complexity, computational approaches that connect cellular heterogeneity with regulatory programs are required. High-dimensional weighted gene co-expression network analysis (hdWGCNA) enables the identification of cell-type-associated co-expression modules and hub genes from scRNA-seq data (12, 13). When applied to stromal compartments, this framework can help identify fibroblast-associated gene programs linked to microenvironmental remodeling and tumor progression.

In the present study, we performed an integrative re-analysis of publicly available scRNA-seq data together with bulk transcriptomic validation, machine learning prioritization, and experimental assays to characterize fibroblast-associated co-expression programs in CRC. Specifically, we analyzed the public scRNA-seq dataset GSE221575 to map the cellular landscape of CRC and adjacent normal tissues, followed by pseudotime analysis, CellChat-based intercellular communication analysis, and hdWGCNA to identify fibroblast-associated transcriptional modules. Candidate genes derived from these modules were further prioritized across five independent GEO bulk transcriptomic cohorts. Among the prioritized genes, DRAM1 was selected for experimental validation in CRC epithelial tumor cell models.

## 2 Methods and statistical analysis

### 2.1 scRNA-seq data acquisition, processing, and cell annotation

scRNA-seq data for CRC, including adjacent normal tissues (GSM6886536 and GSM6886538) and tumor tissues (GSM6886537, GSM6886539, and GSM6886540), were obtained from the Gene Expression Omnibus (GEO) under accession number GSE221575 (14). Data preprocessing was performed using the Seurat package in R (15). Cells expressing fewer than 50 genes, cells with a mitochondrial gene proportion greater than 15%, and genes detected in fewer than three cells were excluded from downstream analysis.

Expression matrices were normalized using the LogNormalize method. The top 1,500 highly variable genes were identified using the variance-stabilizing transformation method. Principal component analysis was then performed, and batch effects among samples were corrected using the Harmony algorithm (16). Unsupervised clustering was conducted based on a shared nearest-neighbor graph using the Louvain algorithm at a resolution of 0.6, and cell clusters were visualized by t-distributed stochastic neighbor embedding. Cell identities were annotated using the SingleR package in combination with reference databases and canonical marker genes (17).

### 2.2 Trajectory inference and cell-cell communication analysis

To investigate developmental dynamics within the CRC microenvironment, differential genes defining each cluster were identified using thresholds of |log2 fold change| > 1 and adjusted P < 0.05. These genes were used to reconstruct cellular trajectories with the Monocle2 package using the DDRTree algorithm (18).

Cell-cell communication analysis was performed using CellChat (19). Based on the “Secreted Signaling” database, communication probabilities and signaling strengths were inferred from significantly overexpressed ligand-receptor pairs among cell types containing at least 10 cells.

### 2.3 High-dimensional weighted gene co-expression network analysis

To define fibroblast-associated transcriptional programs, hdWGCNA was performed on the fibroblast population (20, 21). In this study, “fibroblast-associated transcriptional programs” refers to co-expression modules derived from fibroblast-annotated cells in the scRNA-seq dataset and prioritized according to module connectivity and fibroblast specificity. This term does not imply that all candidate genes are exclusively expressed by fibroblasts or that their biological functions are restricted to fibroblast biology. Genes expressed in at least 5% of fibroblasts were retained. Meta-cells were constructed using the MetacellsByGroups function with k = 25 based on Harmony-corrected embeddings. A signed co-expression network was generated using a soft-thresholding power of 14 according to scale-free topology criteria. Module eigengene-based connectivity and hub-gene-associated module eigengene values were used to evaluate module robustness and fibroblast specificity.

### 2.4 Bulk transcriptomic validation and machine learning prioritization

Five independent bulk CRC transcriptomic cohorts (GSE14333 (22), GSE17536 (23), GSE20916 (24), GSE39582 (25), and GSE9348 (26)) were downloaded from GEO. Expression matrices were normalized and integrated, and batch effects were corrected using the limma and sva packages (27, 28). Candidate fibroblast-associated genes derived from hdWGCNA were intersected with genes available in the merged bulk dataset and further evaluated between CRC and normal tissues. Differential expression analysis was performed using limma, and significantly altered candidate genes were retained for subsequent machine learning-based prioritization.

For machine learning analysis, normalized expression values of the retained candidate genes were used as predictors, and disease status, defined as CRC or normal tissue, was used as the binary outcome. Model training and evaluation were performed using the caret package, version 6.0–90 (29). To ensure reproducibility, the random seed was set to 123. The integrated cohort was randomly divided into a training set and a testing set at a ratio of 7:3. Hyperparameter tuning was conducted in the training set using repeated 5-fold cross-validation with 10 repeats. The evaluated algorithms included partial least squares, random forest, decision tree, support vector machine, logistic regression, k-nearest neighbors, XGBoost, gradient boosting machine, and neural network models. Model performance was assessed by receiver operating characteristic curve analysis, and the area under the curve was calculated using the pROC package, version 1.17.0.1 (30).

Feature importance was assessed using the DALEX package, version 2.4.0, based on a root-mean-square error loss function calculated from predicted probabilities and true class labels (31). The top 10 ranked genes were visualized, and the top five genes were selected as core genes for subsequent biological interpretation and experimental validation. Chromosomal localization of the five core genes was visualized using the circlize package (32). Importantly, the machine learning framework was used for candidate gene prioritization rather than for establishing a clinically validated diagnostic classifier.

### 2.5 Immune infiltration and functional enrichment analysis

To evaluate the immune infiltration landscape in CRC, the CIBERSORT algorithm was applied to estimate the relative proportions of 22 immune cell subsets in bulk transcriptomic samples (33). Associations between candidate core genes and tumor-infiltrating immune cells were analyzed by Spearman correlation.

Single-sample gene set enrichment analysis was performed using the GSVA package to quantify hallmark pathway activity (34). Functional annotation of fibroblast-associated genes was performed by Gene Ontology and Kyoto Encyclopedia of Genes and Genomes enrichment analyses using the clusterProfiler package (35).

### 2.6 Human tissue specimens

Paired CRC tissues and matched adjacent normal tissues were collected from patients who underwent surgical resection at the Department of Gastrointestinal Nutrition and Hernia Surgery, The Second Hospital of Jilin University. No patient had received chemotherapy or radiotherapy before surgery. The tissue specimens were used for western blotting and qRT-PCR analyses. Written informed consent was obtained from all participants. The study was approved by the Ethics Committee of The Second Hospital of Jilin University (Approval No. 2026-037).

### 2.7 Cell culture, transfection, and expression analysis

The normal human colonic epithelial cell line NCM460 and CRC cell lines HCT116, SW480, SW620, and RKO were cultured in DMEM supplemented with 10% fetal bovine serum in a humidified incubator containing 5% CO_2_ at 37 °C. For knockdown experiments, cells were transfected with DRAM1-targeting small interfering RNAs (siRNAs) or negative control siRNA (si-NC) using GoldenTran^®^-R transfection reagent (Golden Trans Technology, Cat. No. 190425010) according to the manufacturer’s instructions. All siRNAs were synthesized by Sangon Biotech (Shanghai, China), and the siRNA sense-strand sequences are listed in **Supplementary Table 1**.

Total RNA was extracted using TRIzol reagent (Invitrogen, Thermo Fisher Scientific, Cat. No. 15596018CN), and complementary DNA was synthesized using TransScript^®^ Uni All-in-One First-Strand cDNA Synthesis SuperMix for qPCR with One-Step gDNA Removal (TransGen Biotech, Cat. No. AU341-02). Quantitative real-time PCR was performed using PerfectStart^®^ Green qPCR SuperMix (TransGen Biotech, Cat. No. AQ601-01-V2). Relative mRNA expression levels were calculated using the 2^-ΔΔCt method with ACTB as the internal control. The primers used for qRT-PCR were synthesized by Sangon Biotech (Shanghai, China), and the primer sequences are listed in **Supplementary Table 2**.

For protein analysis, total protein was extracted from tissues or cultured cells, separated by SDS-PAGE, and transferred onto PVDF membranes. After blocking, the membranes were incubated with primary antibodies against DRAM1, ACTB, or GAPDH overnight at 4 °C, followed by incubation with HRP-conjugated goat anti-mouse secondary antibody. Protein bands were detected using an enhanced chemiluminescence kit (Omni-ECL™ Enhanced Pico Light Chemiluminescence Kit; Epizyme, Cat. No. SQ101). Detailed information on the antibodies, including manufacturers, catalogue numbers, and dilution ratios, is provided in **Supplementary Table 3**.

### 2.8 *In vitro* functional assays

Cell proliferation was assessed using EdU incorporation and CCK-8 assays. For the EdU assay, transfected cells were seeded into 24-well plates at a density of 1 × 10^5 cells per well. When the cells reached approximately 70% confluence, EdU incorporation was detected using the BeyoClick™ Plus EdU Cell Proliferation Kit with AF555 (Beyotime, Cat. No. C0076S) according to the manufacturer’s instructions. EdU-positive cells were visualized by fluorescence microscopy and quantified from randomly selected fields.

For the CCK-8 assay, transfected cells were seeded into 96-well plates at a density of 5 × 10^3 cells per well. At the indicated time points, CCK-8 reagent (Beyotime, Cat. No. C0037) was added to each well and incubated for 1 h at 37 °C. Absorbance was then measured at 450 nm using a microplate reader.

For colony formation assays, transfected cells were seeded into six-well plates at a density of 3 × 10^3 cells per well and cultured for 10–14 days. Colonies were fixed with paraformaldehyde for 15 min and stained with crystal violet for 1 h. Visible colonies were photographed and counted.

Cell migration was evaluated using wound-healing and Transwell migration assays. For wound-healing assays, transfected cells were seeded into plates and cultured until they reached near confluence. A linear scratch was generated using a sterile pipette tip, and detached cells were removed by washing with PBS. Cells were then cultured in low-serum medium, and wound closure was photographed at 0 and 48 h. The wound area was quantified using ImageJ software.

For Transwell migration assays, transfected cells suspended in serum-free medium were added to the upper chambers of 24-well Transwell inserts with 8-μm pores (Corning). Medium containing serum was added to the lower chambers as a chemoattractant. After incubation for 48 h, migrated cells on the lower surface of the membrane were fixed, stained with crystal violet, photographed, and counted. For invasion assays, the upper chambers were pre-coated with Basement Membrane Matrix (MedChemExpress, Cat. No. HY-K6002), and the remaining procedures were performed as described for the migration assay, with an incubation time of 48 h.

### 2.9 *In vivo* xenograft assay

The animal study was approved by the Animal Ethics Committee of the School of Basic Medical Sciences, Jilin University (Approval No. 2025-729). Male BALB/c nude mice aged 4–6 weeks were used for the xenograft experiment. HCT116 cells transfected with si-NC or DRAM1-targeting siRNA were subcutaneously injected into the right axillary region of each mouse. Each group contained five mice.

Animals were monitored regularly throughout the experiment, including tumor growth and general health status. Humane endpoints were predefined as follows: tumor size reaching 15 mm in maximum diameter, tumor ulceration, obvious distress, cachexia, or the scheduled experimental endpoint at 2 weeks after inoculation. Once any humane endpoint was reached, the animals were euthanized as a terminal procedure.

Euthanasia was performed by CO_2_ inhalation using a gradual-fill method in a dedicated euthanasia chamber. Compressed CO_2_ was delivered at a flow rate of 5 L/min, corresponding to approximately 50% of the 10-L chamber volume per minute. CO_2_ exposure was maintained for at least 1 min after cessation of respiration to ensure death. Death was confirmed by the absence of respiration, heartbeat, and response to toe pinch before xenograft tumors were excised, photographed, and weighed. All procedures were performed by trained personnel, and all efforts were made to minimize animal suffering.

### 2.10 Statistical analysis

Statistical analyses were performed using R and GraphPad Prism. Quantitative data are presented as mean ± standard deviation (SD). Comparisons between two groups were performed using Student’s *t*-test, whereas paired *t*-tests were used for paired clinical specimens. Comparisons among multiple groups were performed using one-way analysis of variance (ANOVA) followed by appropriate *post hoc* testing. Correlations were evaluated by Spearman rank correlation analysis. ROC curves were used to assess the discriminative performance of the candidate-prioritization models within the integrated retrospective cohort. All tests were two-sided, and *P* < 0.05 was considered statistically significant.

## 3 Results

### 3.1 Single-cell transcriptomic profiling reveals the CRC cellular landscape and highlights fibroblasts as a major communication node

**Figure 1:**
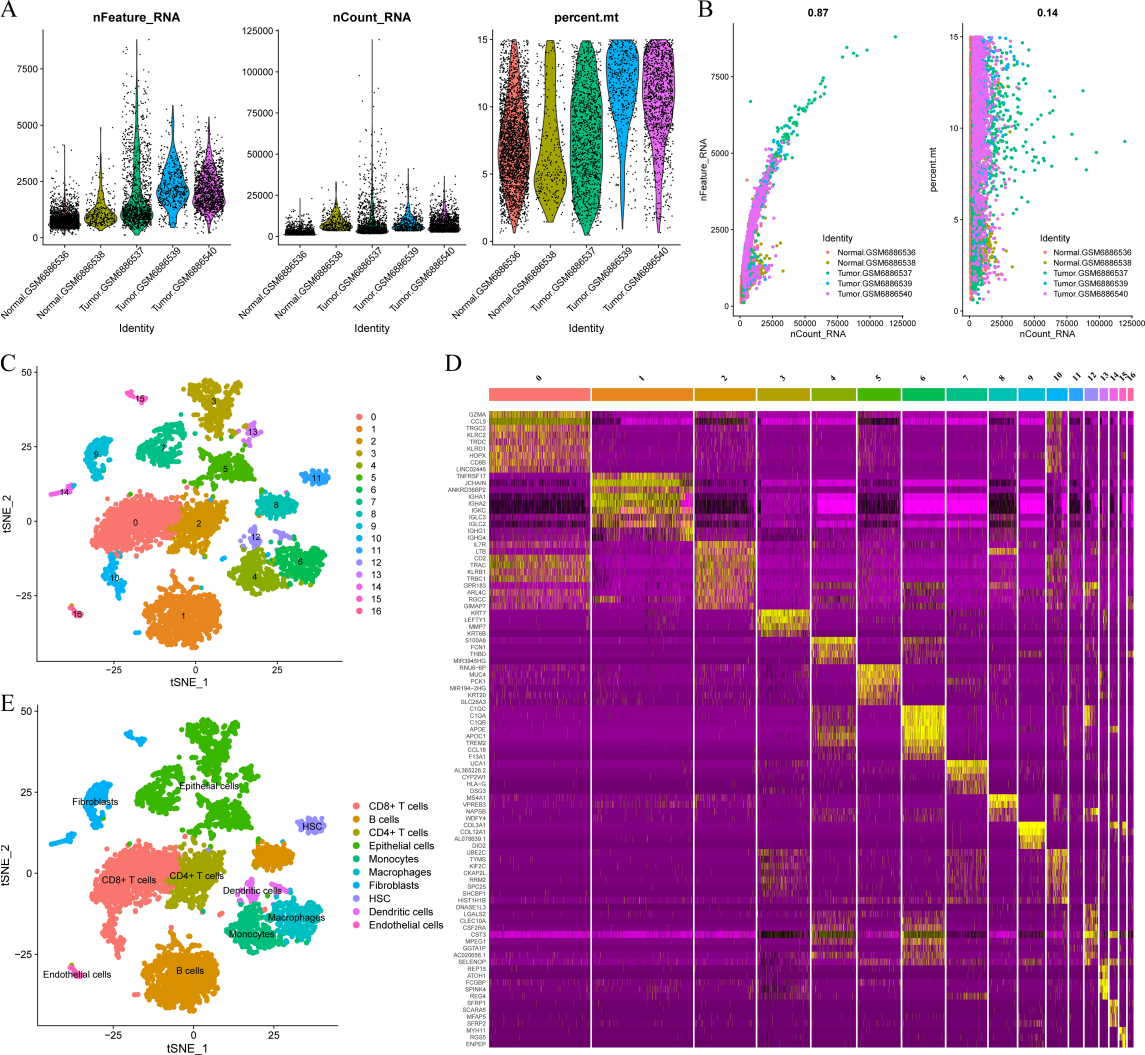
Single-cell transcriptomic landscape and cell-type annotation in CRC. **(A)** Violin plots showing the distributions of quality control metrics, including nCount_RNA, nFeature_RNA, and mitochondrial gene percentage, across normal and CRC samples in the GSE221575 dataset. **(B)** Scatter plots showing the relationships between nCount_RNA and nFeature_RNA before and after filtering. **(C)** t-SNE plot showing 17 cell clusters identified from the integrated dataset. **(D)** Heatmap showing the expression of representative marker genes across the 17 clusters. **(E)** t-SNE plot showing annotation of the 10 major cell lineages based on reference-based annotation and canonical marker genes.

After quality control and filtering of the GSE221575 scRNA-seq dataset (**Figures 1A,B**), 17 transcriptionally distinct cell clusters were identified by t-SNE analysis (**Figures 1C,D**). Based on canonical marker genes and reference-based annotation, these clusters were assigned to 10 major cell lineages (**Figure 1E**), indicating substantial cellular heterogeneity within the CRC microenvironment.

**Figure 2:**
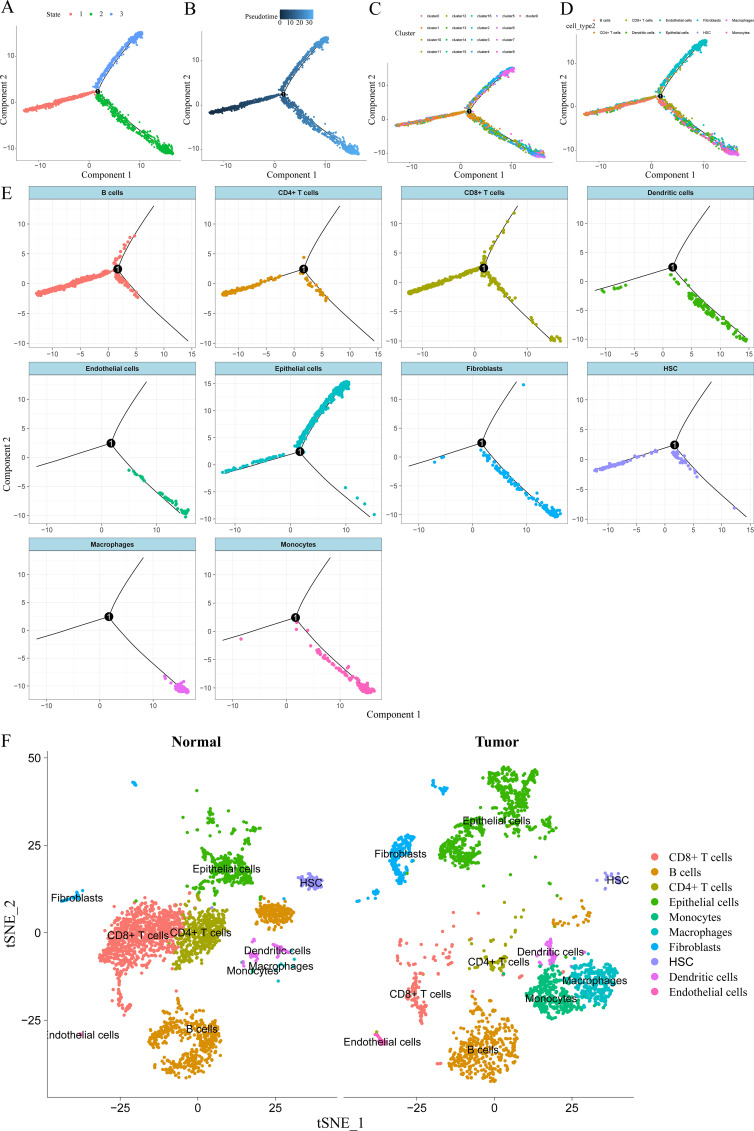
Pseudotime trajectory analysis of cell-state transitions in the CRC microenvironment. **(A)** Monocle2 trajectory plot showing three cell states inferred from single-cell transcriptomic data. **(B)** Pseudotime ordering of cells, with the color gradient indicating progression from early to late states. **(C)** Distribution of the 17 cell clusters along the inferred trajectory. **(D)** Distribution of the 10 annotated cell lineages along the inferred trajectory. **(E)** Pseudotime plots for individual cell lineages. **(F)** Split t-SNE plots showing the distribution of cell populations in normal colorectal tissues and CRC tissues.

To further examine cellular developmental dynamics, pseudotime analysis was performed using Monocle2 (**Figures 2A–E**). Immune cell populations, including B cells and CD4+ T cells, were mainly distributed at earlier pseudotime states, whereas fibroblasts and dendritic cells were more enriched in later states. In addition, split t-SNE plots revealed marked differences in the abundance and distribution of several cell populations between normal and tumor tissues, particularly fibroblasts, CD8+ T cells, and CD4+ T cells (**Figure 2F**).

**Figure 3:**
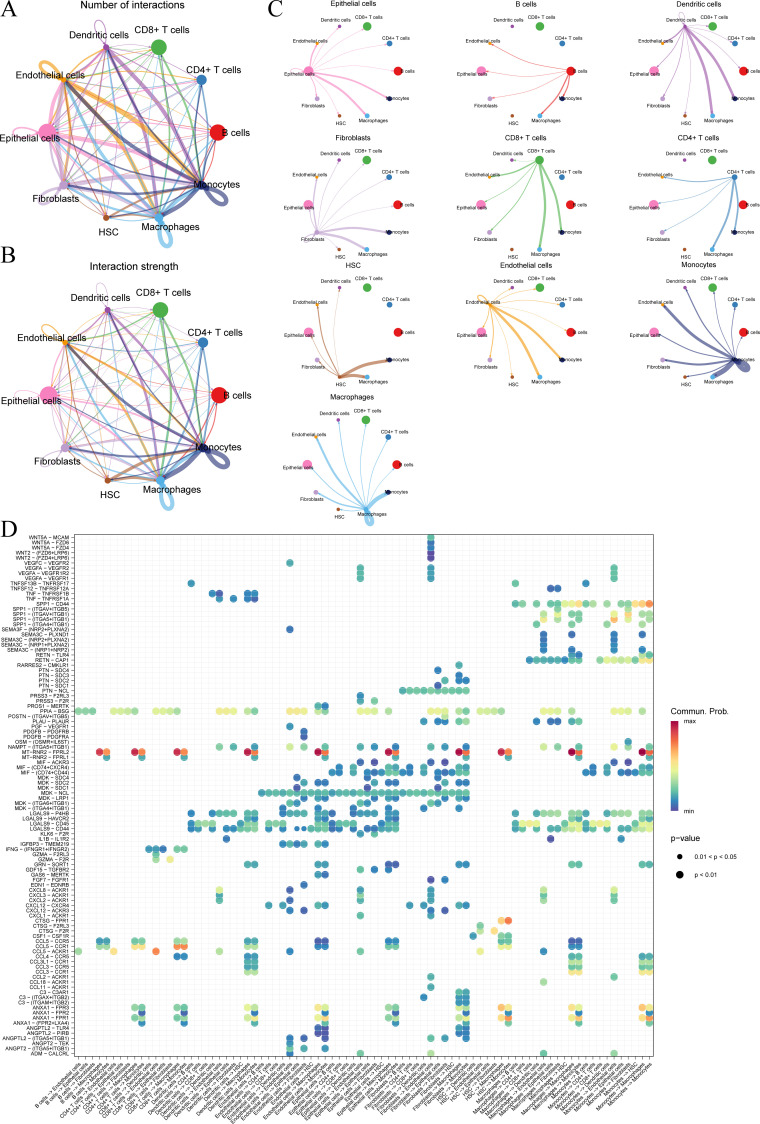
Cell-cell communication analysis of the CRC microenvironment. **(A)** Circle plot showing the total number of inferred interactions among the 10 annotated cell types. **(B)** Circle plot showing the overall interaction strength among the 10 annotated cell types. **(C)** Cell-type-specific communication network showing incoming and outgoing signaling patterns across major cell populations. **(D)** Bubble plot showing signaling probabilities for selected ligand-receptor interactions among interacting cell pairs.

We next explored intercellular communication using CellChat. Global interaction analysis demonstrated extensive signaling exchanges among the 10 annotated cell lineages (**Figures 3A,B**). Among these populations, fibroblasts showed strong incoming and outgoing communication with epithelial cells, endothelial cells, and multiple immune cell subsets (**Figures 3C,D**), indicating that fibroblasts occupy a prominent position within the CRC communication network.

### 3.2 hdWGCNA identifies fibroblast-associated co-expression modules linked to microenvironmental remodeling

**Figure 4:**
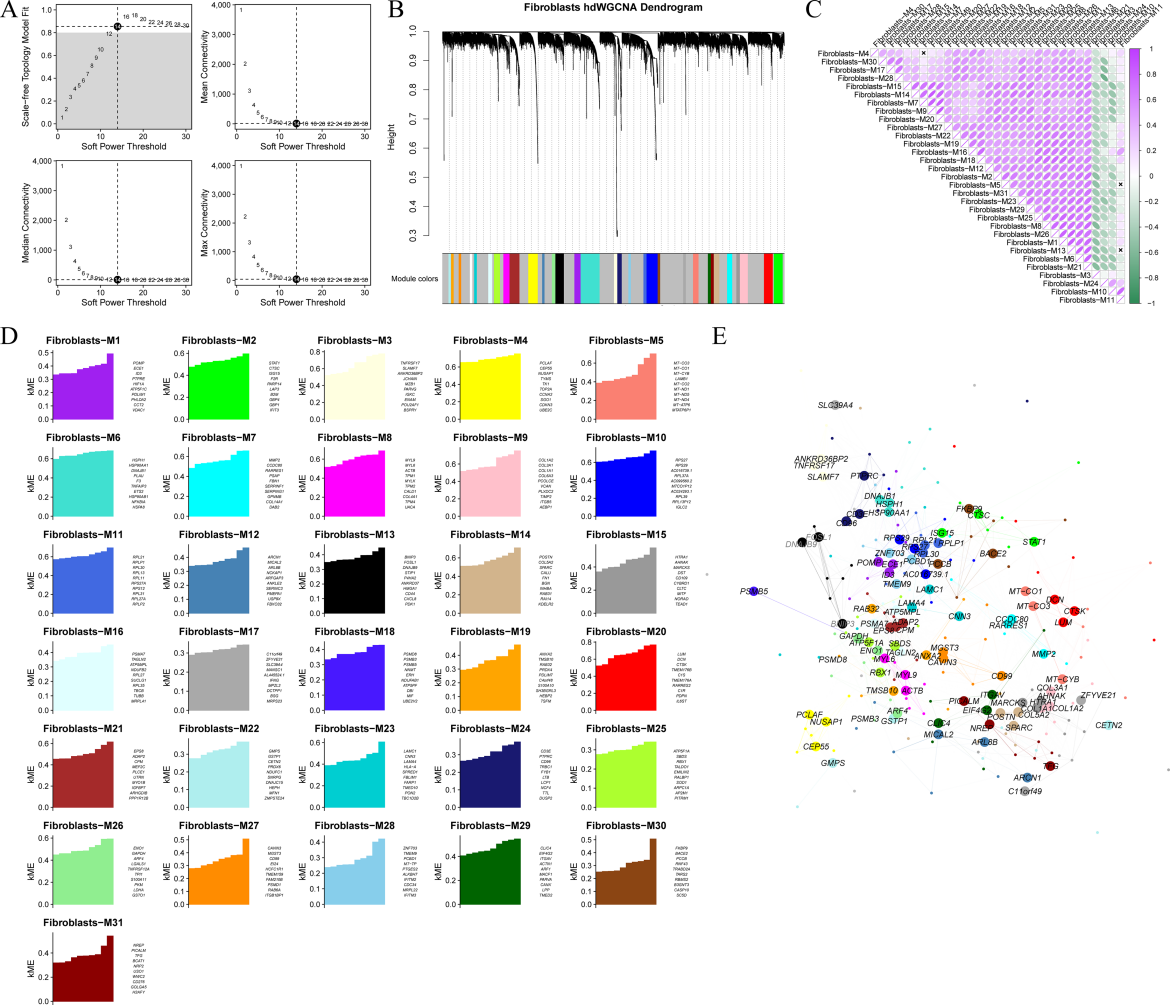
hdWGCNA identifies fibroblast-associated co-expression modules in CRC. **(A)** Scale-free topology fit index and mean connectivity across candidate soft-thresholding powers, showing selection of power = 14. **(B)** Hierarchical clustering dendrogram showing 31 color-coded co-expression modules identified in fibroblasts. **(C)** Heatmap showing correlations among the 31 co-expression modules. **(D)** Bar plots showing the top 10 hub genes in representative modules ranked by module eigengene-based connectivity. **(E)** Network plot showing relationships among representative hub genes across modules.

To characterize fibroblast-associated transcriptional programs, hdWGCNA was performed on the fibroblast compartment. Thus, the candidate genes extracted from these modules were interpreted as fibroblast-associated module genes rather than fibroblast-exclusive markers. A soft-thresholding power of 14 was selected to construct a scale-free co-expression network (**Figure 4A**). This analysis identified 31 co-expression modules within fibroblasts (**Figures 4B–E**; **Supplementary Table 4**), indicating substantial functional heterogeneity within this stromal population.

**Figure 5:**
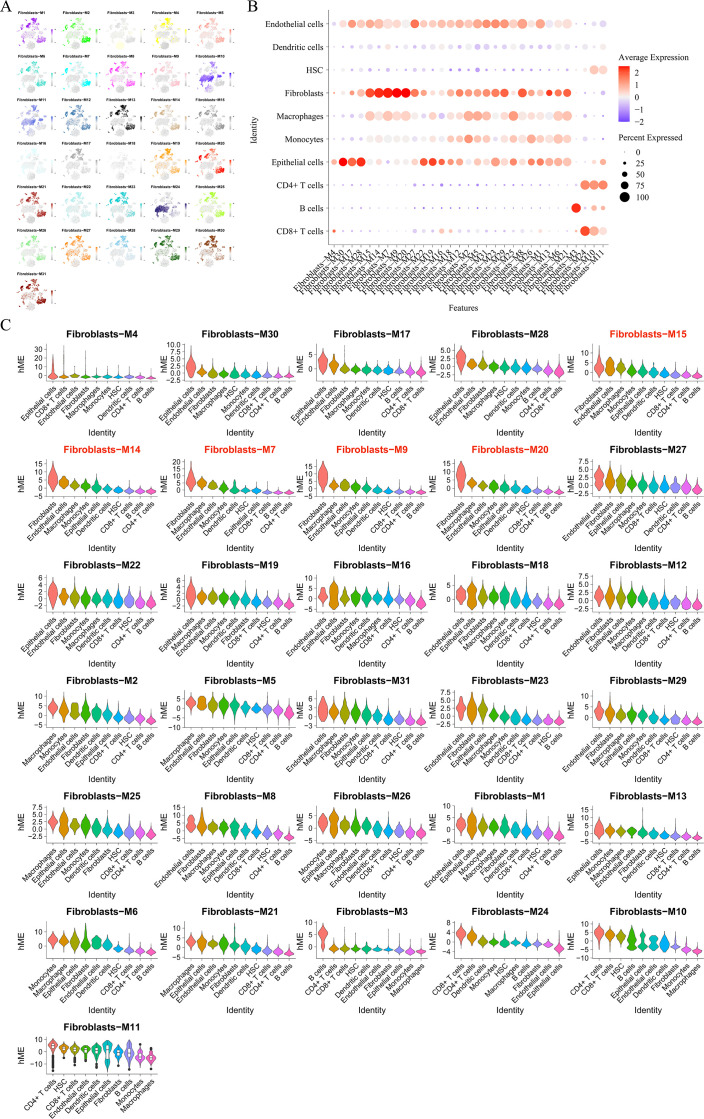
Identification of fibroblast-associated core modules in CRC. **(A)** Feature plots showing module activity patterns of the 31 identified fibroblast-associated modules at the single-cell level. **(B)** Dot plot showing the relationships between the 10 annotated cell lineages and the 31 modules. **(C)** Violin plots showing module eigengene values across cell types for each module. Modules 15, 14, 7, 9, and 20 are highlighted for downstream analysis.

To prioritize fibroblast-relevant modules, module eigengene-based connectivity metrics were evaluated across cell populations. Five modules (Modules 15, 14, 7, 9, and 20) showed the strongest association with fibroblast identity and were selected for downstream analysis (**Figures 5A–C**). From these modules, a total of 123 candidate fibroblast-associated genes were extracted (**Supplementary Figure 1**; **Supplementary Table 5**).

Functional enrichment analysis indicated that these genes were mainly enriched in extracellular matrix organization, regulation of cell proliferation, and microenvironment-related biological processes (**Supplementary Figure 2A**). Kyoto Encyclopedia of Genes and Genomes analysis further suggested involvement in pathways associated with tumor-stroma interaction and immune-related regulation (**Supplementary Figure 2B**).

### 3.3 Integrative bulk transcriptomic analysis and machine learning prioritize core fibroblast-associated genes in CRC

**Figure 6:**
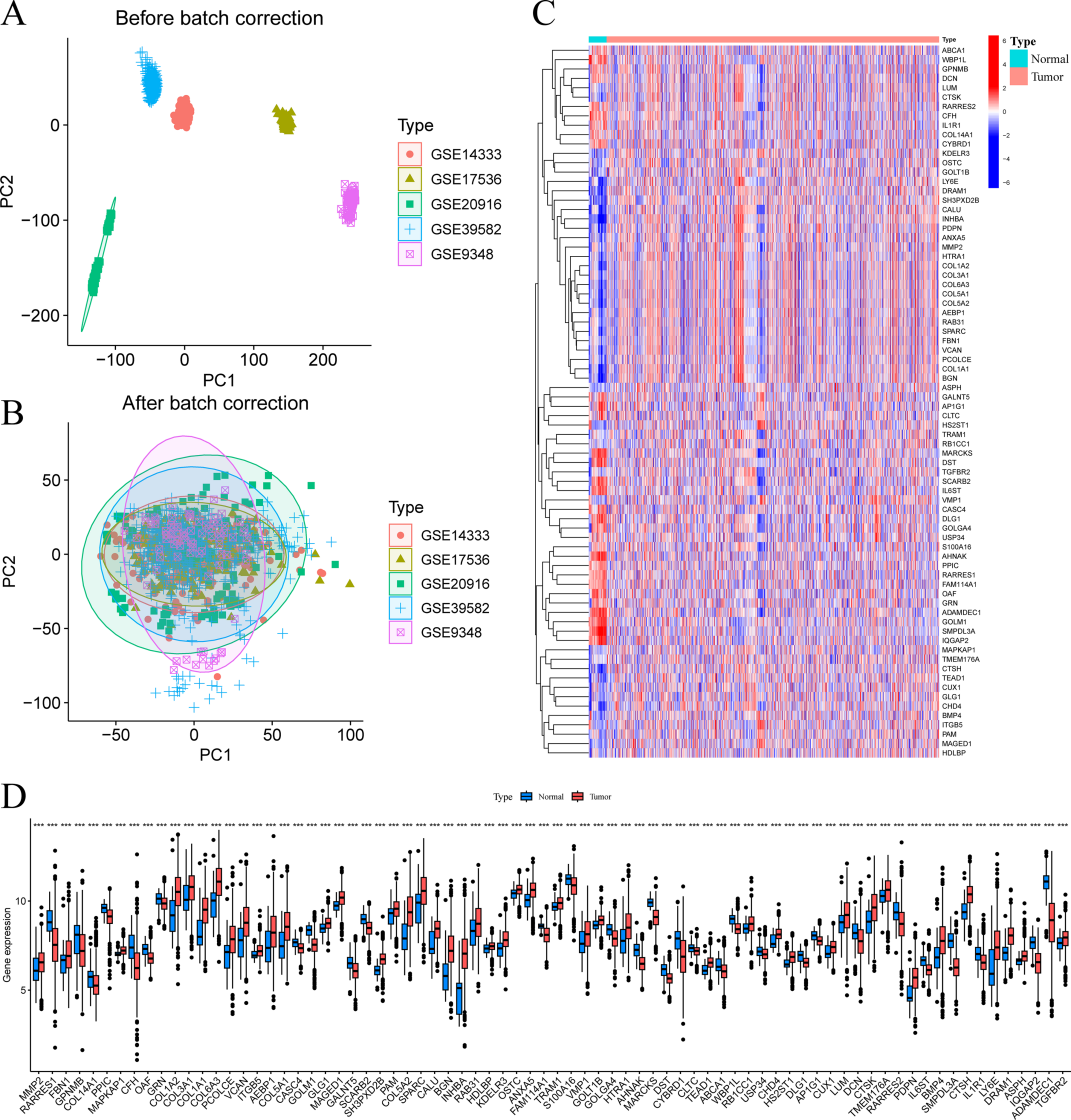
Bulk transcriptomic validation of fibroblast-associated candidate genes. **(A)** Principal component analysis plot of the five integrated GEO cohorts before batch correction. **(B)** Principal component analysis plot of the five integrated GEO cohorts after batch correction. **(C)** Heatmap showing the expression patterns of the 123 candidate genes across integrated normal and CRC bulk transcriptomic samples. **(D)** Boxplots showing expression differences of the 76 differentially expressed fibroblast-associated genes between normal and CRC tissues. ***p < 0.001.

To evaluate the robustness of fibroblast-associated candidates in independent cohorts, five GEO bulk transcriptomic datasets were integrated and batch-corrected (**Figures 6A–C**). Differential expression analysis of the 123 candidate genes identified 76 genes that were significantly different between normal and CRC tissues (**Figure 6D**).

**Figure 7:**
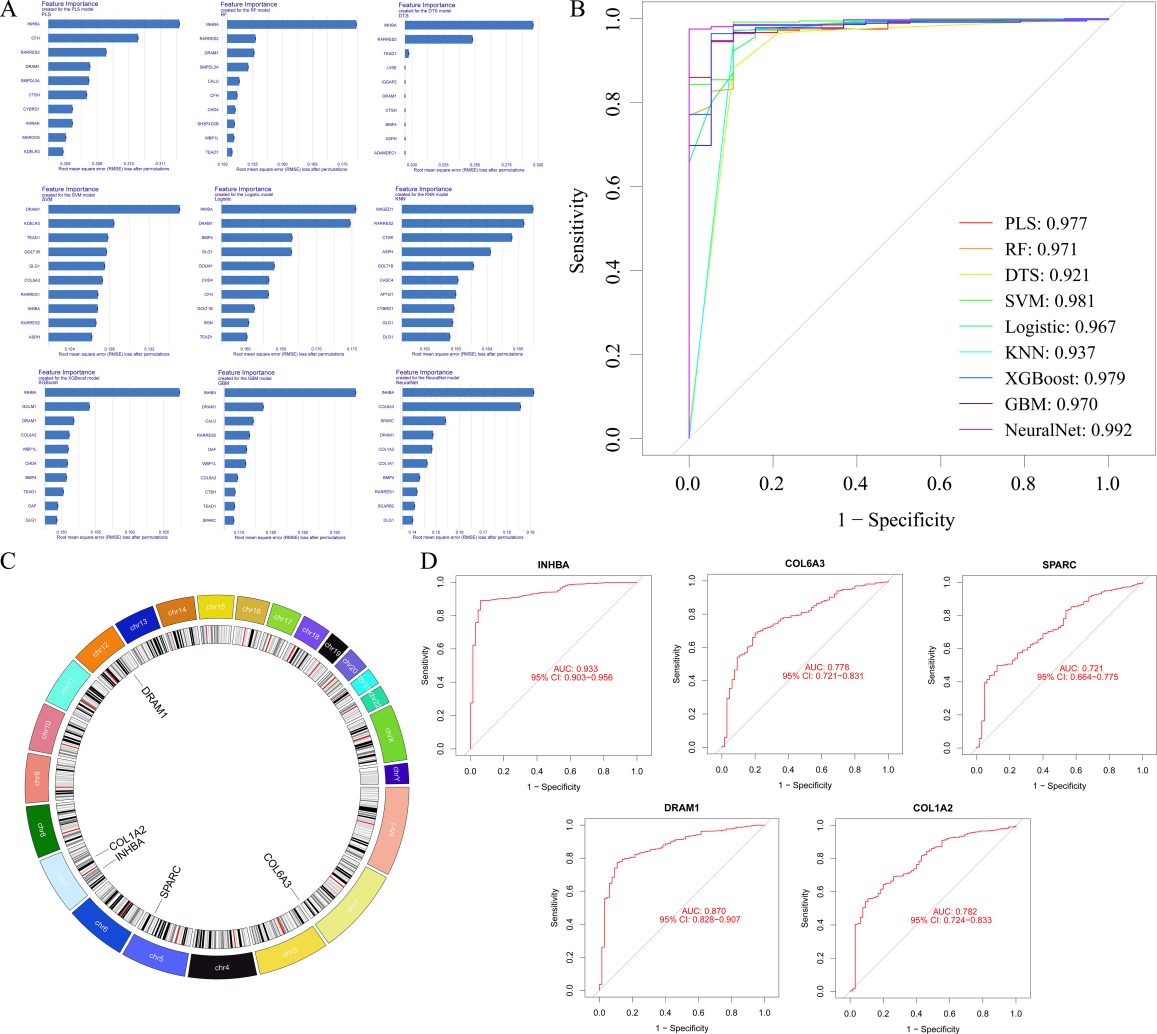
Machine learning-based prioritization of core fibroblast-associated genes. **(A)** Feature importance rankings of candidate genes across evaluated machine learning models. **(B)** Receiver operating characteristic (ROC) curves comparing the discriminative performance of the evaluated models, with the NeuralNet model showing the highest AUC. **(C)** Circos plot showing the chromosomal locations of the five core genes, including INHBA, COL6A3, SPARC, DRAM1, and COL1A2. **(D)** ROC curves showing the discriminative ability of the five core genes in distinguishing CRC from normal tissues.

These 76 genes were then subjected to machine learning-based prioritization. Although feature rankings varied across models (**Figure 7A**), the NeuralNet model showed the highest discriminative performance among the evaluated models, with an AUC of 0.992 (**Figure 7B**). Given the retrospective nature of the dataset and the lack of prospective external validation, this result was interpreted as supporting candidate gene prioritization rather than as evidence of clinical diagnostic performance. Based on this model, five genes—INHBA, COL6A3, SPARC, DRAM1, and COL1A2—were selected as core genes for further analysis. Chromosomal localization and individual ROC analyses further illustrated their discriminative signals within the integrated retrospective dataset (**Figures 7C,D**).

### 3.4 Core fibroblast-associated genes are linked to immune infiltration patterns and pathway activity in CRC

**Figure 8:**
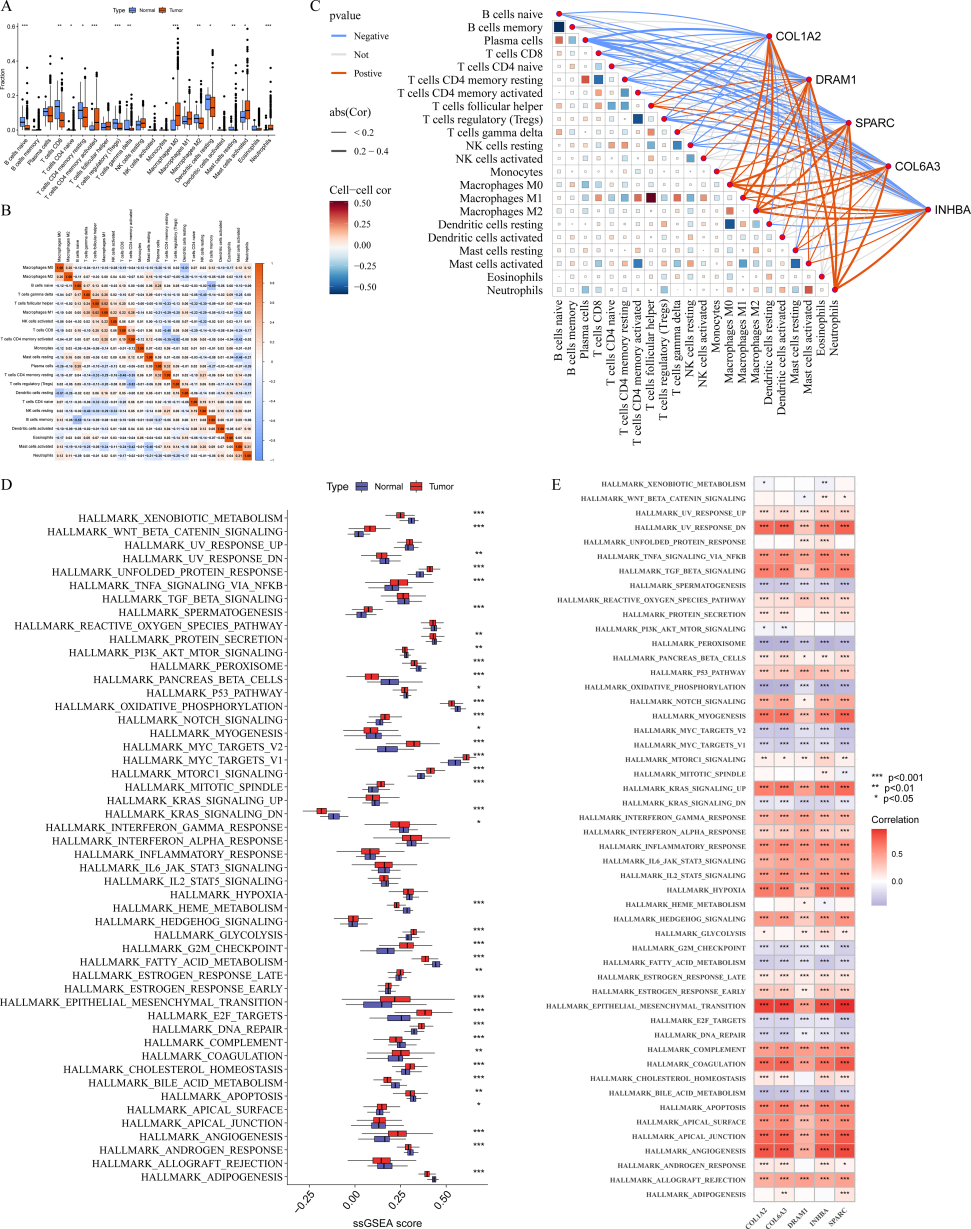
Immune infiltration patterns and pathway activity associated with core fibroblast-associated genes. **(A)** CIBERSORT analysis showing the relative proportions of 22 immune cell subsets in normal and CRC tissues. **(B)** Correlation matrix showing relationships among tumor-infiltrating immune cell subsets. **(C)** Heatmap showing correlations between the five core genes and immune cell infiltration levels. **(D)** Single-sample gene set enrichment analysis scores showing differences in hallmark pathway activity between normal and CRC tissues. **(E)** Heatmap showing correlations between the five core genes and hallmark pathway scores. *p < 0.05, **p < 0.01, ***p < 0.001.

To investigate the immune context associated with the five core genes, CIBERSORT analysis was first used to estimate the composition of tumor-infiltrating immune cells. The proportions of several immune cell subsets, including CD4+ T cells, CD8+ T cells, and macrophages, differed between normal and CRC tissues (**Figures 8A,B**), indicating substantial immune remodeling in the TME.

Spearman correlation analysis showed that the five core genes were associated with multiple immune cell populations (**Figure 8C**). In parallel, single-sample gene set enrichment analysis revealed altered activity of hallmark pathways between normal and tumor tissues (**Figure 8D**). Correlation analysis between gene expression and pathway scores further suggested that these genes were linked to both immune-associated and oncogenic signaling programs (**Figure 8E**).

Taken together, these findings indicate that the fibroblast-associated core genes identified in this study are associated not only with stromal features but also with broader immune and signaling changes in the CRC microenvironment.

### 3.5 DRAM1 suppresses malignant phenotypes of CRC cells *in vitro* and *in vivo*

**Figure 9:**
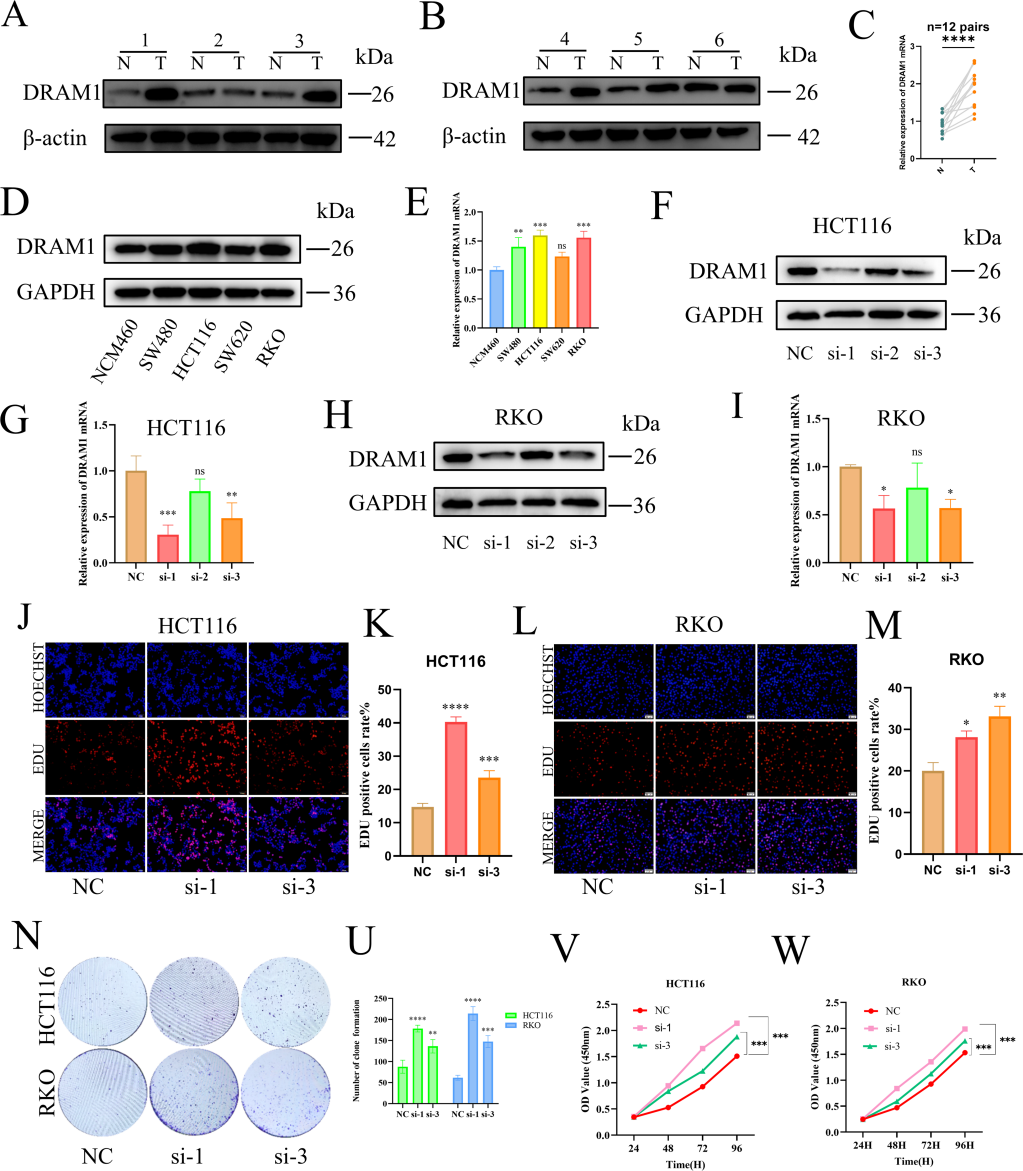
DRAM1 expression and effects on CRC cell proliferation *in vitro*. **(A, B)** Western blot analysis and quantification of DRAM1 protein expression in paired normal and tumor tissues from CRC patients. **(C)** qRT-PCR analysis of DRAM1 mRNA expression in paired clinical samples. **(D, E)** Western blot analysis and quantification of endogenous DRAM1 expression in the normal colonic epithelial cell line NCM460 and four CRC cell lines. **(F–I)** Validation of siRNA-mediated DRAM1 knockdown efficiency in HCT116 and RKO cells by western blotting and quantification. **(J–M)** EdU assays and quantification showing proliferation changes after DRAM1 knockdown in HCT116 and RKO cells. **(N–U)** Colony formation assays and quantification showing clonogenic changes after DRAM1 knockdown. **(V, W)** CCK-8 assays showing cell viability over time after DRAM1 knockdown. ns not significant, *p < 0.05, **p < 0.01, ***p < 0.001.

Among the five prioritized core genes, DRAM1 was selected for experimental validation because it was identified from fibroblast-associated modules, showed consistent differential expression in the integrated bulk analysis, was associated with immune- and pathway-related features, and remains less well characterized in CRC compared with extracellular matrix-associated genes such as COL1A2, COL6A3, and SPARC. Western blotting and qRT-PCR analyses showed that DRAM1 expression was higher in CRC tissues than in matched adjacent normal tissues (**Figures 9A–C**). In cultured cells, DRAM1 expression was also elevated in HCT116 and RKO cells relative to the normal colonic epithelial cell line, and these two CRC cell lines were therefore selected for loss-of-function experiments (**Figures 9D–I**).

Following siRNA-mediated knockdown of DRAM1, EdU and colony formation assays showed increased proliferative and clonogenic capacity in both HCT116 and RKO cells (**Figures 9J–U**). CCK-8 assays further supported enhanced cell viability after DRAM1 silencing (**Figures 9V,W**). These findings indicate that DRAM1 restrains CRC cell growth *in vitro*.

**Figure 10:**
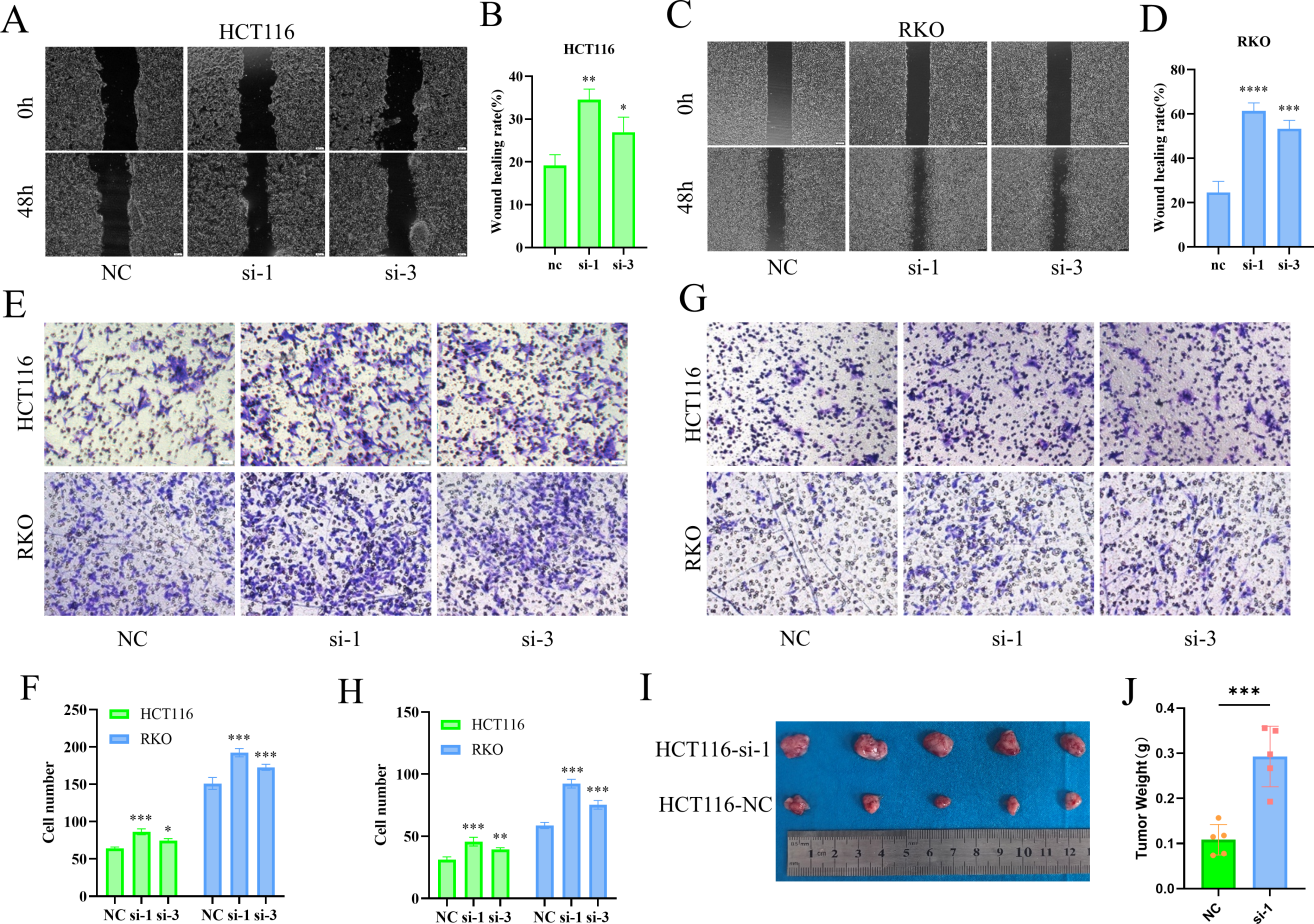
Effects of DRAM1 knockdown on CRC cell migration, invasion, and tumor growth. **(A–D)** Wound-healing assays and quantification showing migratory changes in HCT116 and RKO cells after DRAM1 knockdown. **(E, F)** Transwell migration assays and quantification showing migration changes after DRAM1 knockdown. **(G, H)** Matrigel-coated Transwell invasion assays and quantification showing invasion changes after DRAM1 knockdown. **(I)** Representative images of xenograft tumors derived from control and DRAM1-silenced HCT116 cells. **(J)** Quantification of xenograft tumor weights in the indicated groups. *p < 0.05, **p < 0.01, ***p < 0.001.

We next examined whether DRAM1 affects cell motility. Wound-healing and Transwell assays demonstrated that DRAM1 knockdown promoted migration and invasion of CRC cells (**Figures 10A–H**). Consistent with the *in vitro* results, xenograft experiments showed that tumors derived from DRAM1-silenced HCT116 cells were larger and heavier than those in the control group (**Figures 10I,J**). Collectively, these findings indicate that DRAM1 restrains malignant phenotypes in the tested CRC epithelial tumor cell models. Although DRAM1 was elevated in CRC tissues and selected CRC cell lines, its knockdown further enhanced tumor-promoting phenotypes, suggesting that DRAM1 upregulation may represent a compensatory stress-response mechanism rather than direct evidence of an oncogenic function.

## 4 Discussion

CRC remains the third most commonly diagnosed malignancy and a leading cause of cancer-related mortality globally. Despite advancements in standard treatments, including surgical resection and systemic therapies, the prognosis for patients with advanced or metastatic CRC is often unsatisfactory (36). CRC progression is driven not only by tumor-intrinsic genetic alterations but also by dynamic remodeling of the TME, in which stromal and immune components play indispensable roles (37). Among these components, fibroblasts are increasingly recognized as functionally heterogeneous regulators of extracellular matrix organization, intercellular communication, and immune contexture (38–40). However, the transcriptional diversity of fibroblast populations and their contribution to stromal-immune crosstalk in CRC remain incompletely understood (41). In the present study, we integrated single-cell transcriptomics, hdWGCNA, bulk transcriptomic validation, machine learning prioritization, and experimental assays to characterize fibroblast-associated programs in CRC and to evaluate the biological relevance of DRAM1.

Recent studies have emphasized that CRC-related biomarkers should be interpreted in the context of tumor heterogeneity, inflammation, and microenvironmental remodeling rather than as tumor-cell-intrinsic indicators alone. Clinically, established biomarkers such as CEA/CEACAM5, MSI/MMR status, RAS and BRAF mutations, HER2 amplification, NTRK fusions, and immune checkpoint-related markers are already used for disease monitoring, molecular stratification, prognosis evaluation, or therapeutic decision-making (42). Transcriptome-based classifications, including the consensus molecular subtypes, further demonstrate that CRC progression is shaped by integrated tumor-intrinsic, stromal, immune, and inflammatory features (43). Consistent with this view, recent single-cell and multi-omics studies have revealed immune heterogeneity, inflammatory remodeling, epithelial reprogramming, and prognostically relevant cell subpopulations during CRC development and progression (44–47). In this context, the five core genes identified in our study should be regarded as research-stage candidate biomarkers emerging from fibroblast-associated transcriptional programs and broader stromal-immune remodeling. Among them, DRAM1 was selected for functional validation; however, unlike established clinical CRC biomarkers, its clinical value remains exploratory and requires further validation in larger prospective cohorts, independent clinical datasets, and mechanistic fibroblast- or co-culture-based models.

By leveraging the scRNA-seq dataset GSE221575, we delineated a high-resolution cellular landscape of the CRC microenvironment and identified extensive intercellular communication among malignant, stromal, and immune cell populations (48, 49). Notably, fibroblasts exhibited strong ligand-receptor interactions with epithelial, endothelial, and multiple immune cell lineages, supporting the concept that fibroblasts are active participants in TME remodeling rather than merely structural components (50–53). In parallel, pseudotime analysis suggested dynamic state transitions of fibroblast populations during CRC progression, further supporting the presence of functionally distinct fibroblast states within the tumor milieu (54). From an immunological perspective, these findings indicate that fibroblasts may contribute to shaping both stromal architecture and local immune behavior in CRC.

To further resolve fibroblast-associated regulatory programs, we performed hdWGCNA and identified 31 fibroblast-associated co-expression modules, from which 123 representative genes were extracted. Functional enrichment analysis linked these genes to extracellular matrix remodeling (48, 55), TGF-β signaling (56), angiogenesis (57) and other pathways closely related to tumor progression and microenvironmental regulation (58). Integration of multi-cohort GEO validation with machine learning further prioritized five core genes, namely INHBA, COL6A3, SPARC, DRAM1, and COL1A2. Wentao Li et al. reported that INHBA promotes malignant progression in colorectal cancer by reprogramming tumor-associated macrophages toward an M2 phenotype in the tumor microenvironment and by inhibiting mitochondrial-dependent ferroptosis in CRC cells (59). Kim et al. showed that COL6A3 contains a tumor stroma-specific epitope that is highly presented across multiple solid tumors, supporting the possibility that COL6A3 may be relevant to stromal-targeted immunotherapeutic strategies in colorectal cancer (60). Xiang et al. showed that SPARC contributes to malignant progression and chemoresistance in colorectal cancer by enhancing aerobic glycolysis and promoting 5-fluorouracil resistance via the STAT3/HK2 axis (61). Chen et al. showed that COL1A2 contributes to colorectal cancer progression and metastasis through extracellular matrix remodeling downstream of the ANGPTL3/integrin αVβ3 signaling axis (62). Together, these genes showed discriminative signals within retrospective datasets and were closely linked to fibroblast-associated modules, suggesting that fibroblast-associated transcriptional programs may capture broader stromal and immune remodeling in CRC rather than merely reflecting isolated stromal features.

Among these prioritized candidates, DRAM1 was selected for in-depth functional validation due to its high computational ranking and its previously uncharacterized role in the context of fibroblast-associated CRC progression. While DRAM1 (DNA damage regulated autophagy modulator 1) is recognized for its function in autophagy and lysosomal homeostasis (63), its specific contribution to TME-mediated tumor cell behavior was unclear. In hepatocellular carcinoma, Rui Zhang et al. identified the interaction between DRAM1 and VAMP8 as a critical mechanism driving HCC metastasis (63). In the context of alcohol-related liver disease–associated hepatocellular carcinoma, Jie Tan et al. reported that DRAM1 expression was markedly upregulated in alcohol-related liver disease, where it may facilitate malignant transformation by exacerbating hepatocyte inflammation and apoptosis (64). In lung adenocarcinoma, Li et al. reported that DRAM1 suppressed cell proliferation and migration, further supporting the possibility that DRAM1 may exert context-dependent or tumor-restraining effects in certain cancer settings (65). Taken together, DRAM1 exhibits context-dependent roles in cancer, promoting metastasis via autophagy while exerting tumor-suppressive effects through p53-dependent DNA damage responses, warranting further investigation (66). In our study, DRAM1 expression was elevated in CRC tissues compared with matched adjacent normal tissues, as confirmed by both western blotting and qRT-PCR analyses of patient specimens. Consistently, DRAM1 was also highly expressed in HCT116 and RKO cells. At first glance, the observation that DRAM1 is upregulated in CRC tissues while DRAM1 silencing enhances malignant phenotypes appears paradoxical. However, elevated expression of stress-responsive genes in tumors does not necessarily indicate a purely oncogenic function. DRAM1 was originally identified as a p53-regulated lysosomal protein that links DNA damage responses to autophagy and programmed cell death, processes that are closely associated with tumor suppression (67). In addition, DRAM1 has been implicated in the regulation of autophagy, lysosomal function, and apoptosis-related pathways, suggesting that its biological effects may depend on cellular context and stress conditions (68). Therefore, increased DRAM1 expression in CRC tissues may reflect an adaptive or compensatory response to tumor-associated stress, including genomic instability, metabolic stress, hypoxia, or microenvironmental pressure. In this context, DRAM1 upregulation may represent an attempted tumor-restraining response rather than direct evidence of an oncogenic function. Consistent with this interpretation, our loss-of-function experiments demonstrated that DRAM1 knockdown significantly promoted CRC cell proliferation, clonogenicity, migration, and invasion *in vitro*. Moreover, these findings were corroborated *in vivo*, where DRAM1 silencing accelerated xenograft tumor growth. Together, these results support a tumor-restraining role for DRAM1 in the tested CRC models, while further mechanistic studies are required to define how DRAM1-mediated stress-response pathways influence CRC progression.

The inclusion of patient tissue data and *in vivo* xenograft results strengthens the biological relevance of our findings. The patient-derived western blotting and qRT-PCR results indicate that DRAM1 dysregulation is not limited to cultured cell models but is present in clinical CRC tissues. Meanwhile, the xenograft data provide *in vivo* support that reduced DRAM1 expression facilitates tumor growth. Taken together, these observations suggest that DRAM1 may function as a stress-responsive barrier against malignant progression, even though its expression is increased in tumor tissues. Such a phenomenon is not unprecedented, as molecules involved in autophagy, apoptosis, or DNA damage responses may be upregulated in tumors but still exert protective or compensatory functions that restrain further progression.

From an immunological and microenvironmental perspective, our integrative analyses further suggested that DRAM1 was associated with variation in immune infiltration and pathway activity. In particular, DRAM1 showed inverse correlations with several oncogenic and inflammatory hallmark pathways, including epithelial-mesenchymal transition, hypoxia, and inflammatory signaling, which are closely intertwined with fibroblast-tumor crosstalk. These findings raise the possibility that DRAM1 may participate in coordinating tumor cell stress responses with microenvironmental signaling states. In addition, the observed associations between DRAM1 and immune-related features suggest that DRAM1 may be linked to the broader immune context of CRC. Interestingly, previous transcriptomic profiling has shown that DRAM1 expression can be altered under PD-1 blockade conditions, indicating a potential connection between DRAM1-related stress pathways and immune checkpoint-associated signaling (69). Although our current study does not directly establish a causal role for DRAM1 in immune regulation, these findings support the hypothesis that DRAM1 may be associated with transcriptional programs that connect tumor-cell stress responses with broader microenvironmental states, although this hypothesis requires direct mechanistic validation.

Nevertheless, several limitations should be acknowledged. First, although DRAM1 was prioritized from fibroblast-associated co-expression modules, the functional assays in the present study were performed primarily in CRC epithelial tumor cell lines and xenograft models derived from these cells. Therefore, our findings support DRAM1 as a candidate gene emerging from fibroblast-associated transcriptional programs and as a tumor-restraining factor in CRC epithelial tumor cells, but they do not establish DRAM1 as a direct regulator of fibroblast function or fibroblast-mediated stromal-immune crosstalk. Future studies using fibroblast-specific perturbation, tumor cell–fibroblast co-culture systems, spatial transcriptomics, or multiplex immunofluorescence will be required to clarify the causal role of DRAM1 in stromal-immune regulation. Second, although the NeuralNet model achieved a high AUC in the integrated bulk transcriptomic cohort, this result should be interpreted cautiously. The model was developed using retrospective public datasets, and no prospective external validation cohort was available. Therefore, the machine learning analysis was used mainly as a feature-prioritization strategy rather than as a clinically validated diagnostic model. Future studies involving larger independent cohorts, prospective validation, and standardized transcriptomic platforms will be required to confirm the diagnostic or prognostic utility of the identified genes. Third, the scRNA-seq discovery dataset used in this study contained a limited number of CRC and adjacent normal tissue samples. Although this dataset enabled cell-type-resolved exploration of the CRC microenvironment, the limited sample size may affect the stability of inferred fibroblast-associated modules and cell-cell communication patterns. To partially mitigate this limitation, candidate genes derived from the scRNA-seq analysis were further evaluated across five independent bulk transcriptomic cohorts. Nevertheless, validation in additional large-scale scRNA-seq cohorts, spatial transcriptomic datasets, and experimentally defined fibroblast models will be necessary to confirm the generalizability of these transcriptional programs.

Overall, this study provides an integrative view of fibroblast-associated transcriptional programs in colorectal cancer by combining single-cell transcriptomics, co-expression network analysis, cross-cohort validation, and functional experiments. Through this framework, we identified DRAM1 as a candidate gene derived from fibroblast-associated transcriptional programs and demonstrated its tumor-restraining effects in CRC epithelial tumor cell models. The inclusion of patient tissue validation, cell-based assays, and xenograft experiments further strengthens the biological plausibility of this finding. Although the precise downstream mechanism of DRAM1 and its direct role in fibroblast-mediated regulation require further investigation, our results highlight fibroblast-associated molecular programs as a useful source for candidate gene discovery in CRC.

## 5 Conclusion

In summary, our integrative analyses identified DRAM1 as a candidate gene derived from fibroblast-associated transcriptional programs in colorectal cancer. Patient tissue validation, cell-based assays, and xenograft experiments further supported a tumor-restraining role for DRAM1 in CRC epithelial tumor cell models. These findings highlight fibroblast-associated transcriptional programs as a useful source for candidate gene discovery in CRC, while the direct involvement of DRAM1 in fibroblast-mediated stromal-immune regulation requires further mechanistic validation.

## Data Availability

The original contributions presented in the study are included in the article/**Supplementary Material**. Further inquiries can be directed to the corresponding author.

## Supplementary material

Supplementary Figure 1 Gene network for the top 25 genes in each fibroblast-associated module.

Supplementary Figure 2 **(A)** GO analysis, including biological processes (BP), cellular components (CC), and molecular functions (MF). **(B)** KEGG pathway analysis.
