# Supplementary figures and images for: Integrative single-cell and bulk transcriptomic analyses identify DRAM1 as a candidate gene from fibroblast-associated transcriptional programs in colorectal cancer
Source: Front Oncol. 2026 Jun 11;16:1862796. doi: 10.3389/fonc.2026.1862796 (PMC13293847; doi:10.3389/fonc.2026.1862796)

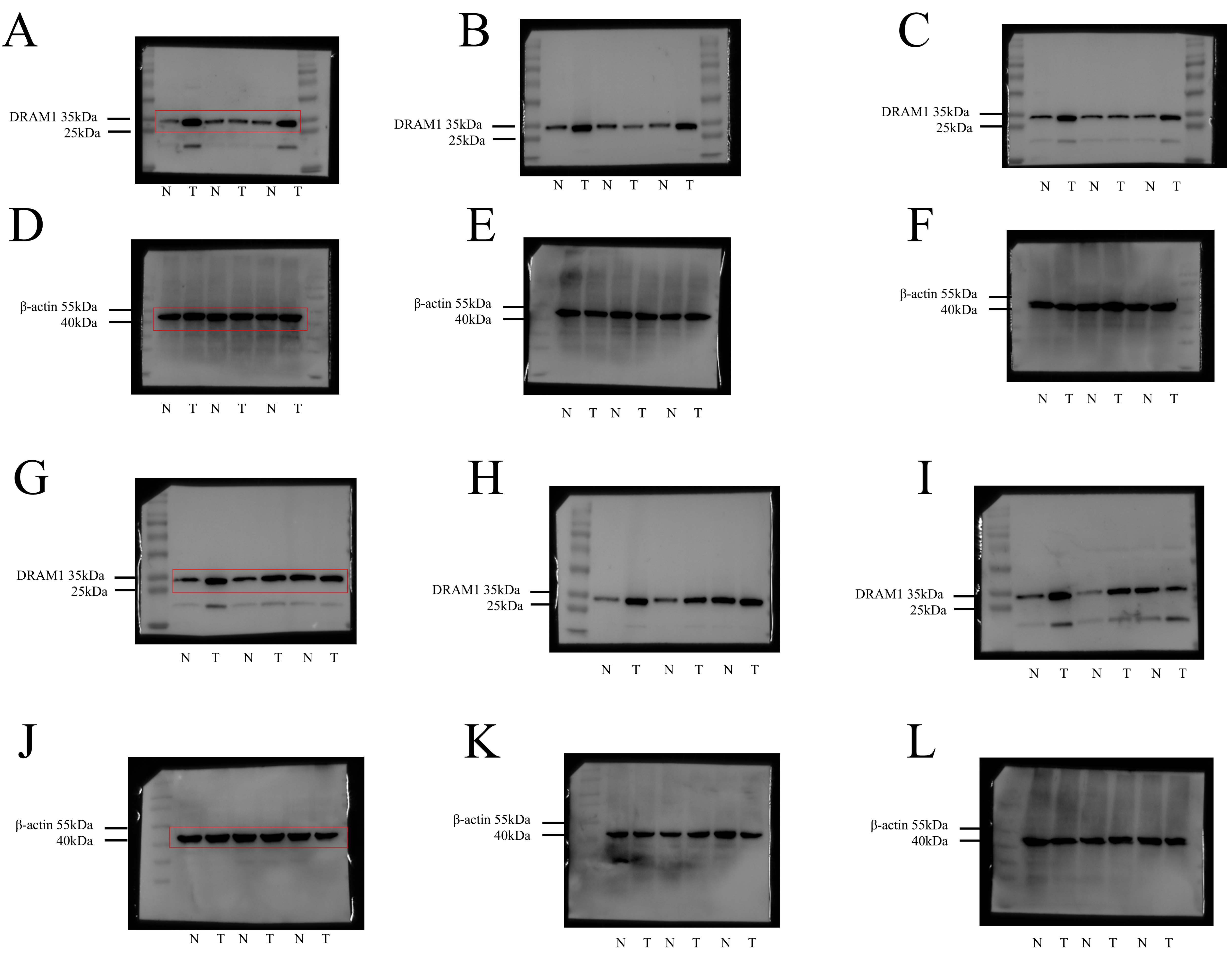

Supplement: Supplementary Figure 1 — Gene network for the top 25 genes in each fibroblast-associated module. [file Image1.tif]

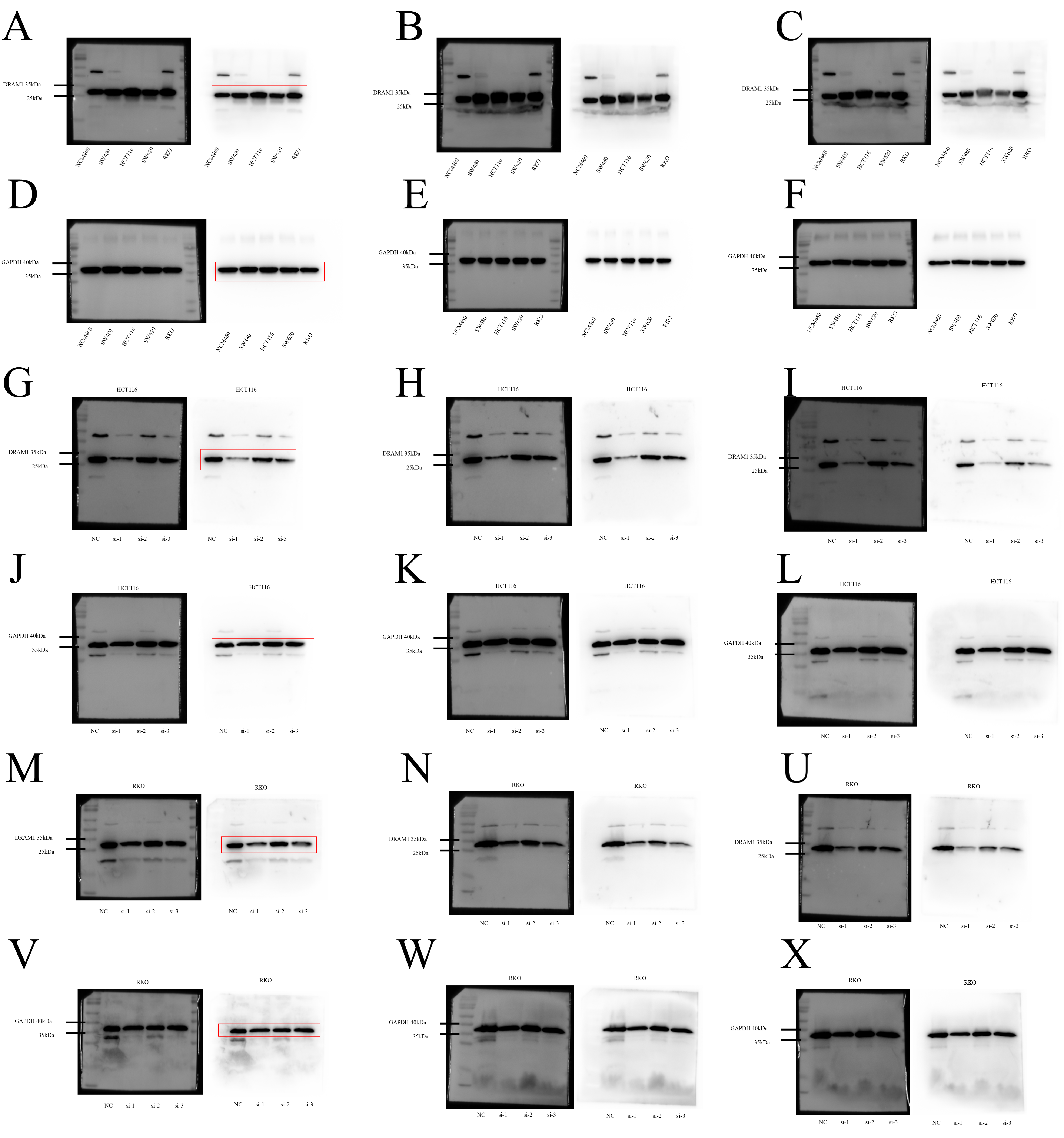

Supplement: Supplementary Figure 2 — (A) GO analysis, including biological processes (BP), cellular components (CC), and molecular functions (MF). (B) KEGG pathway analysis. [file Image2.tif]

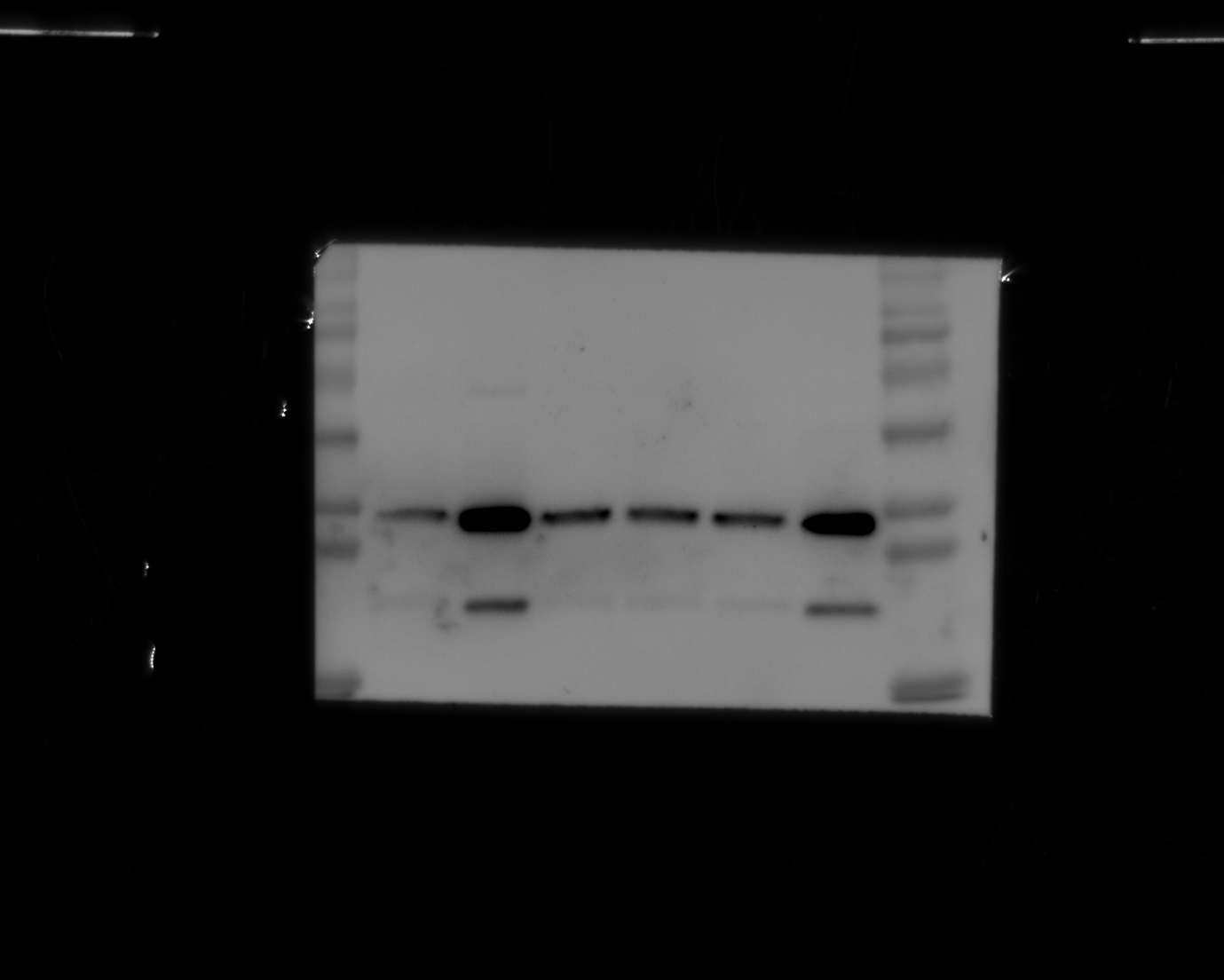

Supplement: Supplementary file 3 [file DataSheet1.zip › Supplementary_Raw_Blot/Supplementary_Raw_Blot_Fig9A_DRAM1---1.tif]

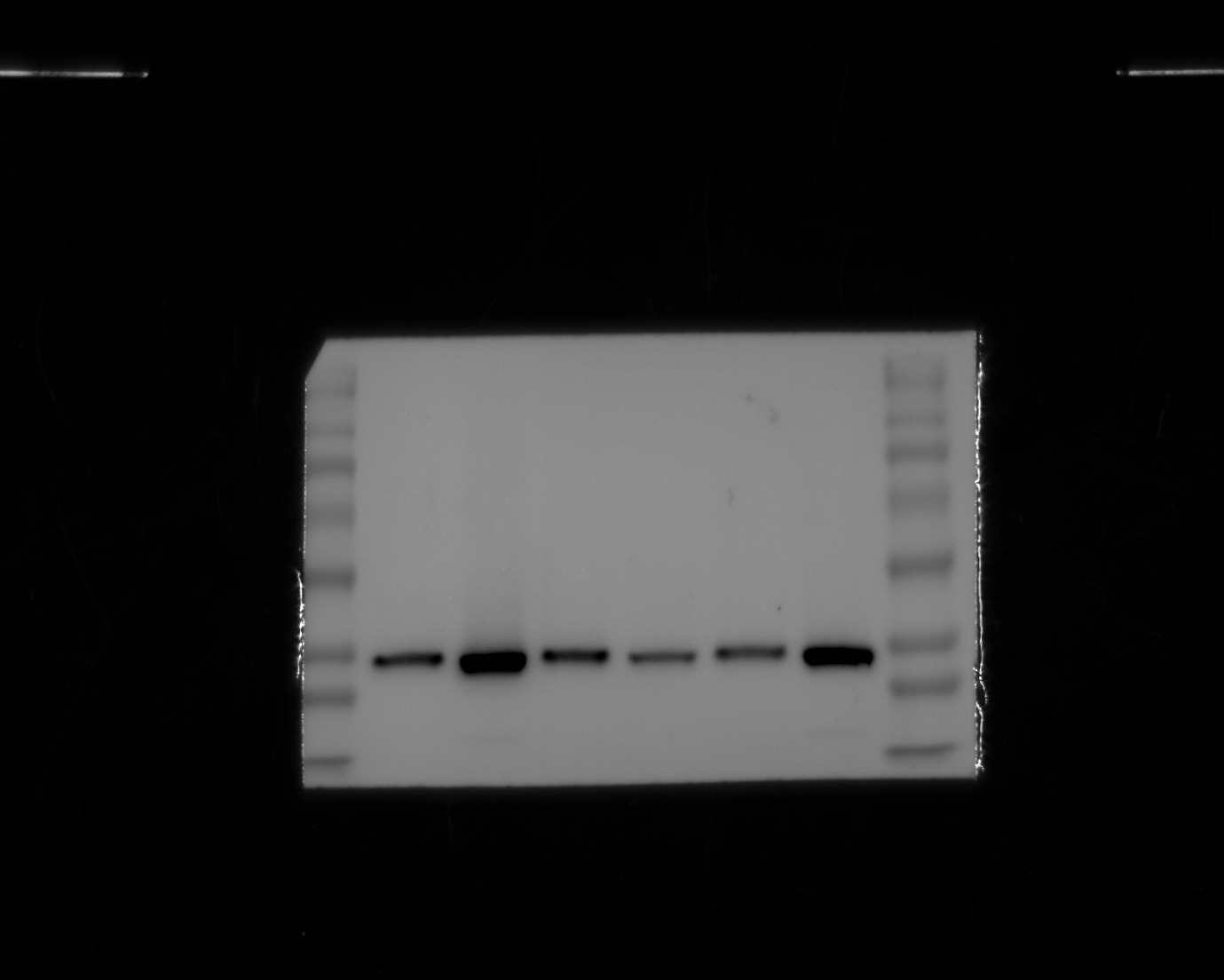

Supplement: Supplementary file 3 [file DataSheet1.zip › Supplementary_Raw_Blot/Supplementary_Raw_Blot_Fig9A_DRAM1---2.tif]

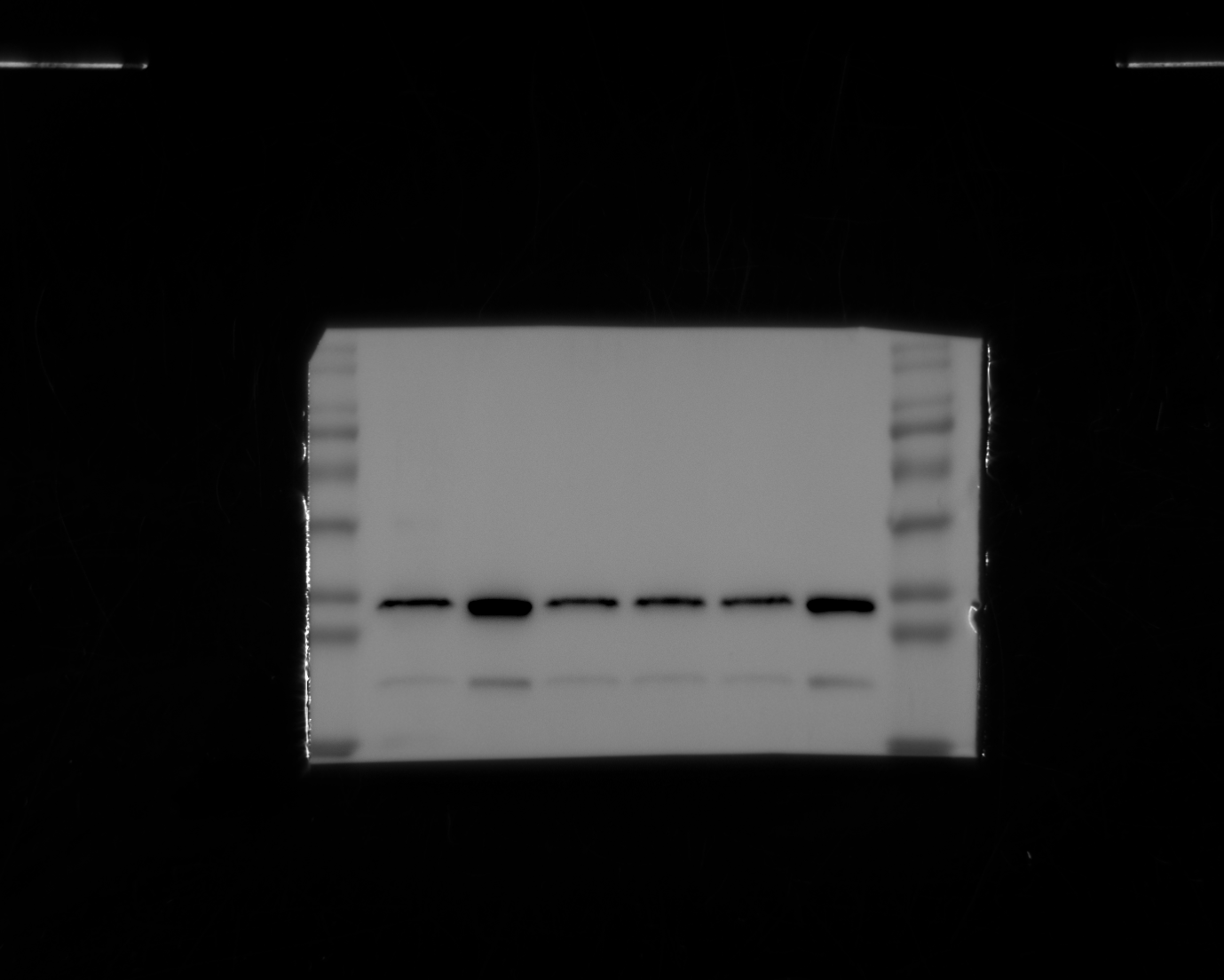

Supplement: Supplementary file 3 [file DataSheet1.zip › Supplementary_Raw_Blot/Supplementary_Raw_Blot_Fig9A_DRAM1---3.tif]

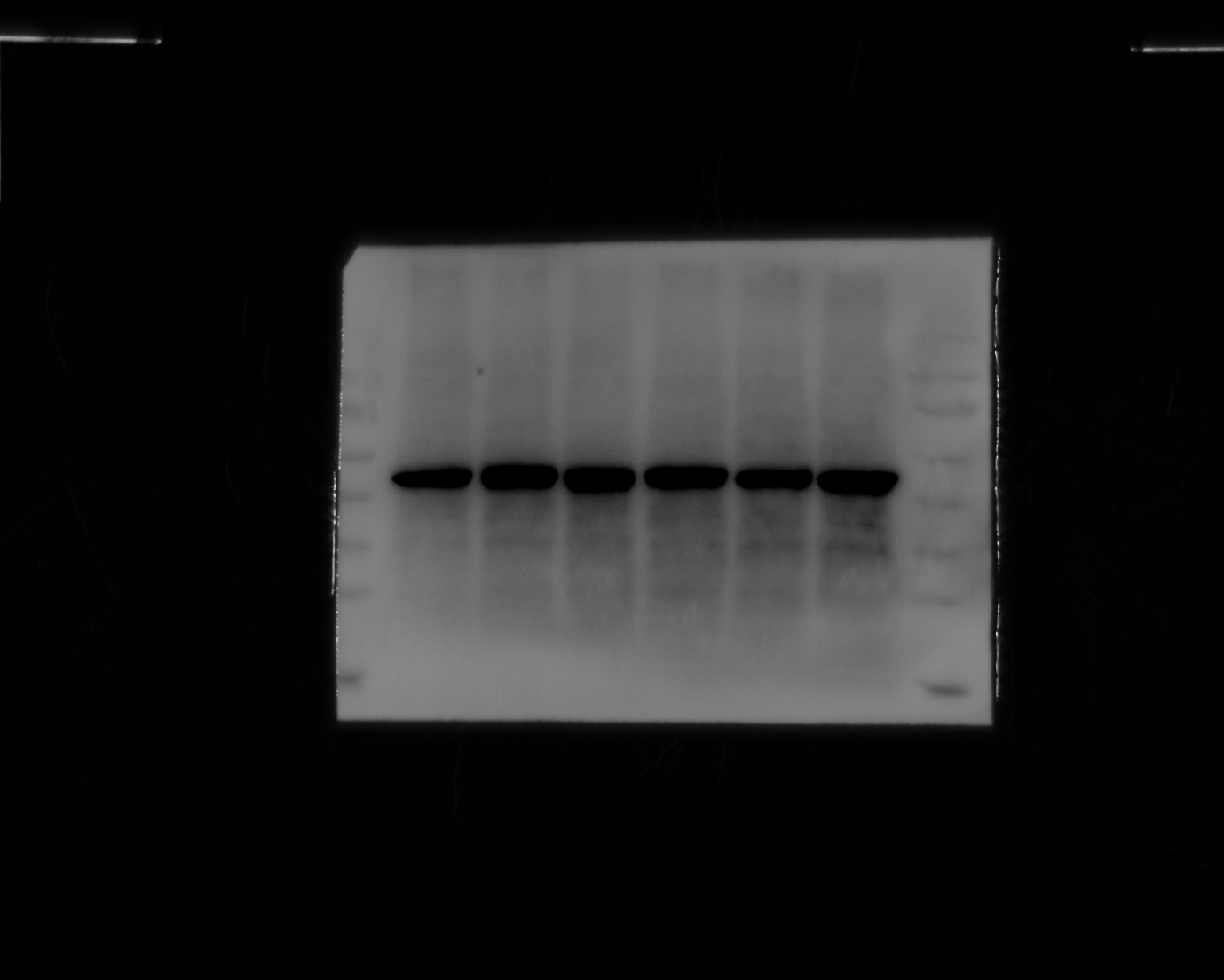

Supplement: Supplementary file 3 [file DataSheet1.zip › Supplementary_Raw_Blot/Supplementary_Raw_Blot_Fig9A_β-actin---1.tif]

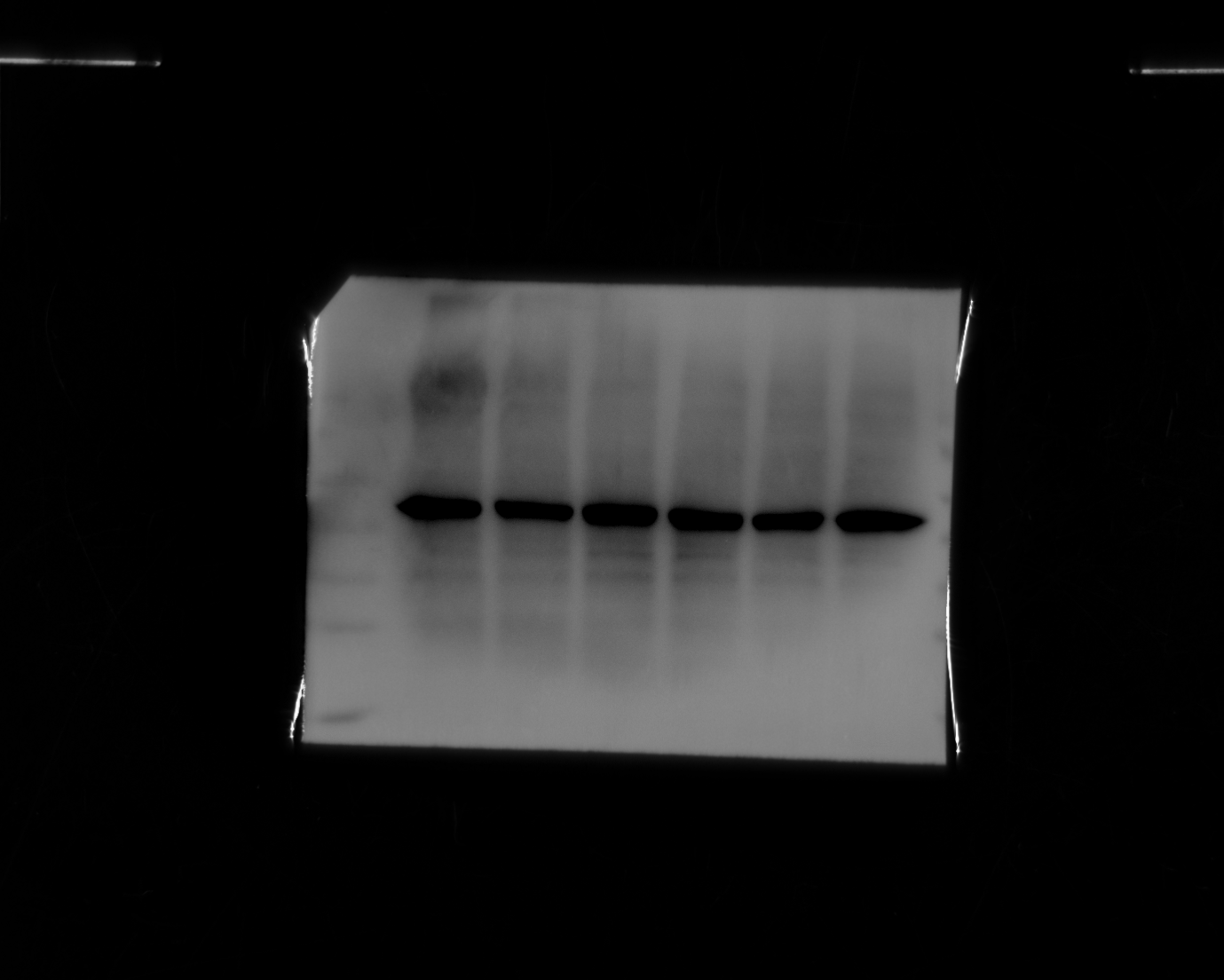

Supplement: Supplementary file 3 [file DataSheet1.zip › Supplementary_Raw_Blot/Supplementary_Raw_Blot_Fig9A_β-actin---2.tif]

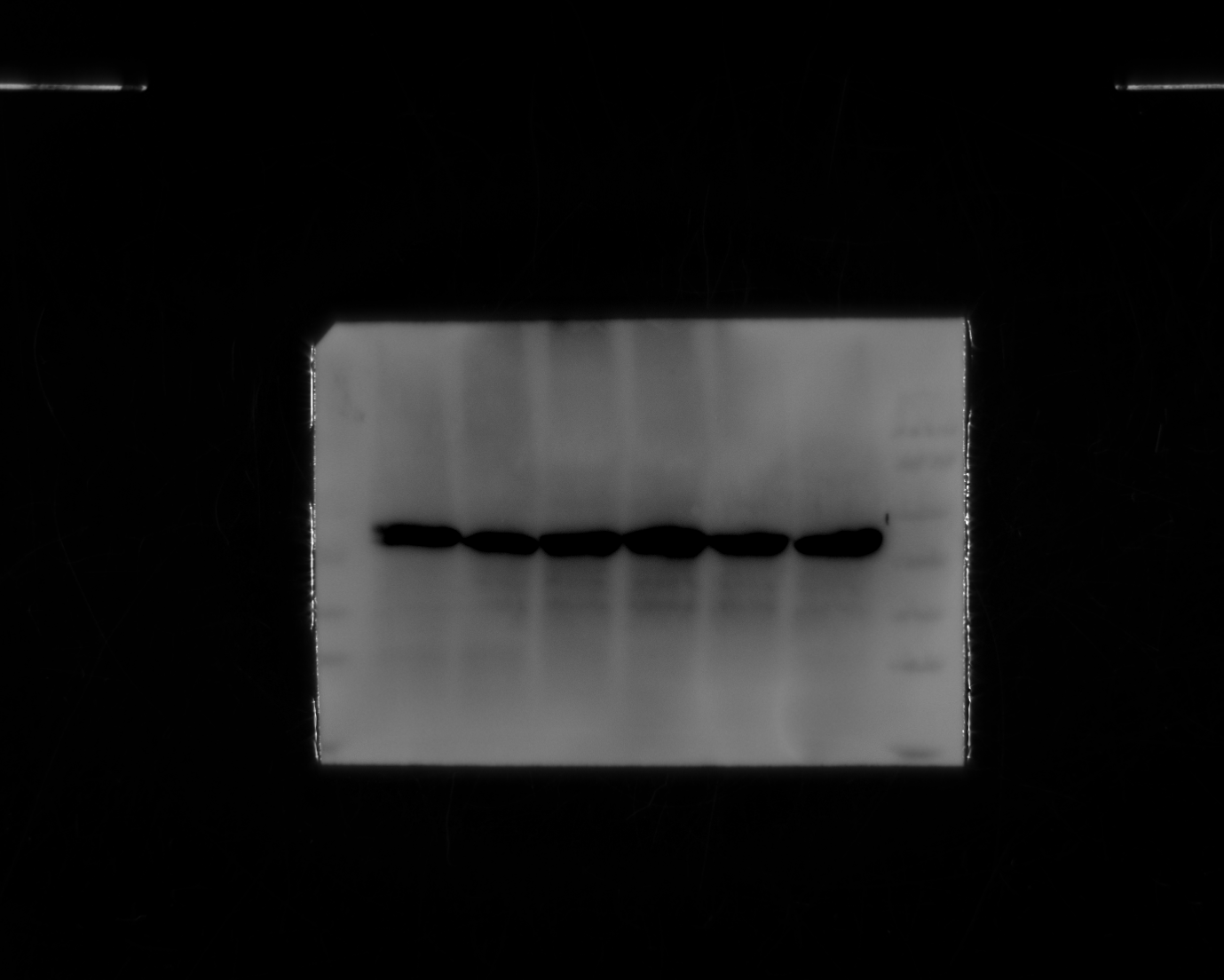

Supplement: Supplementary file 3 [file DataSheet1.zip › Supplementary_Raw_Blot/Supplementary_Raw_Blot_Fig9A_β-actin---3.tif]

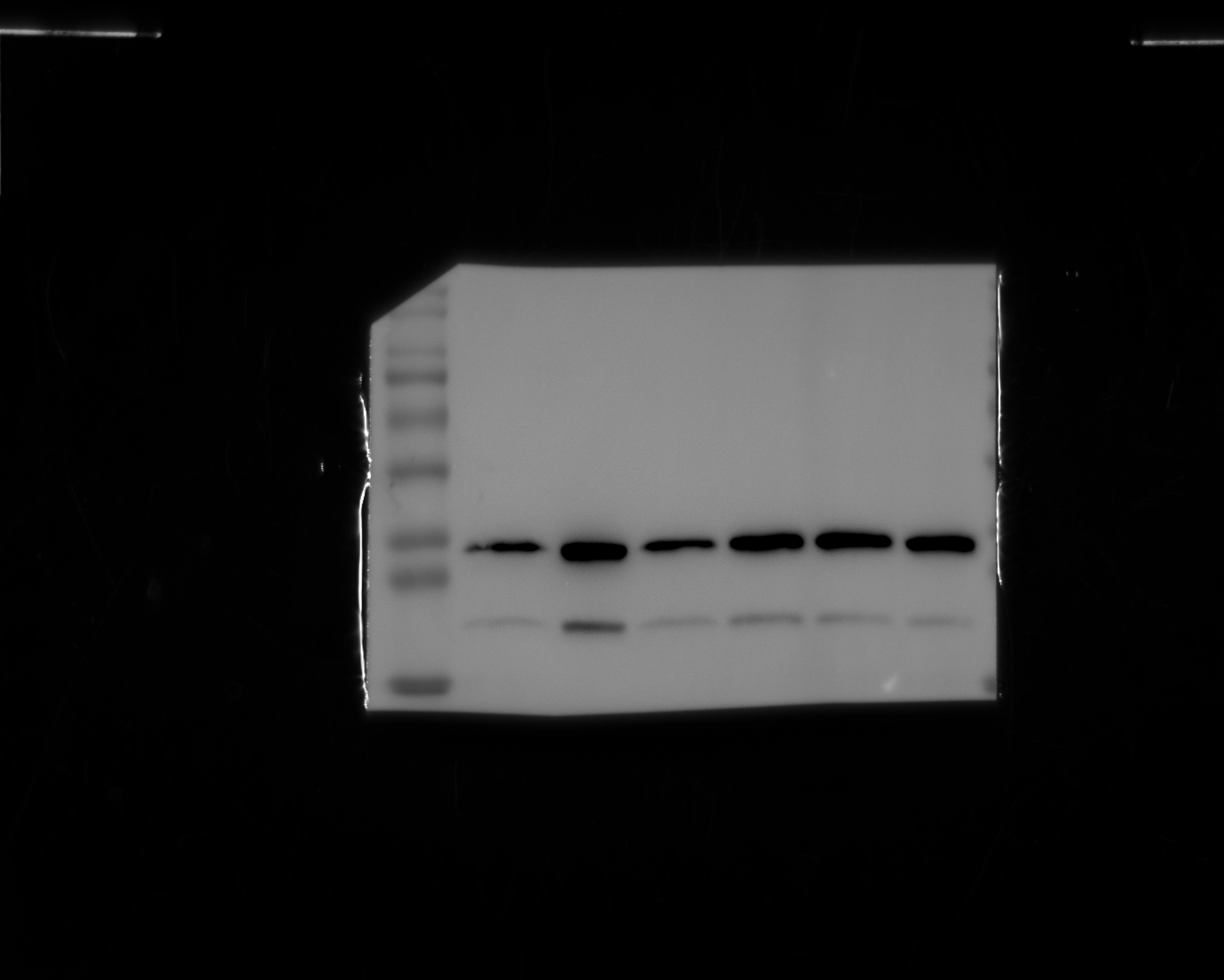

Supplement: Supplementary file 3 [file DataSheet1.zip › Supplementary_Raw_Blot/Supplementary_Raw_Blot_Fig9B_DRAM1---1.tif]

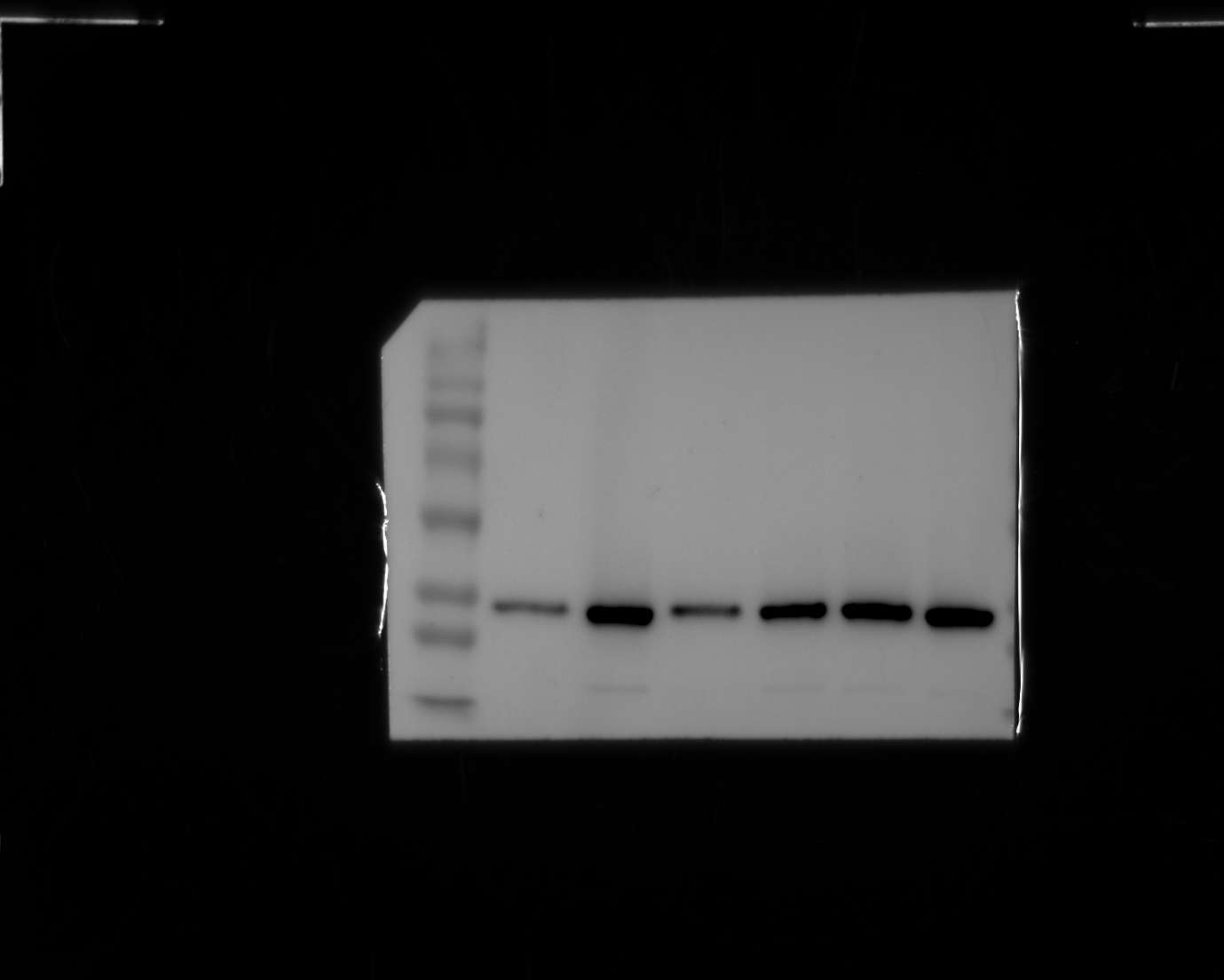

Supplement: Supplementary file 3 [file DataSheet1.zip › Supplementary_Raw_Blot/Supplementary_Raw_Blot_Fig9B_DRAM1---2.tif]

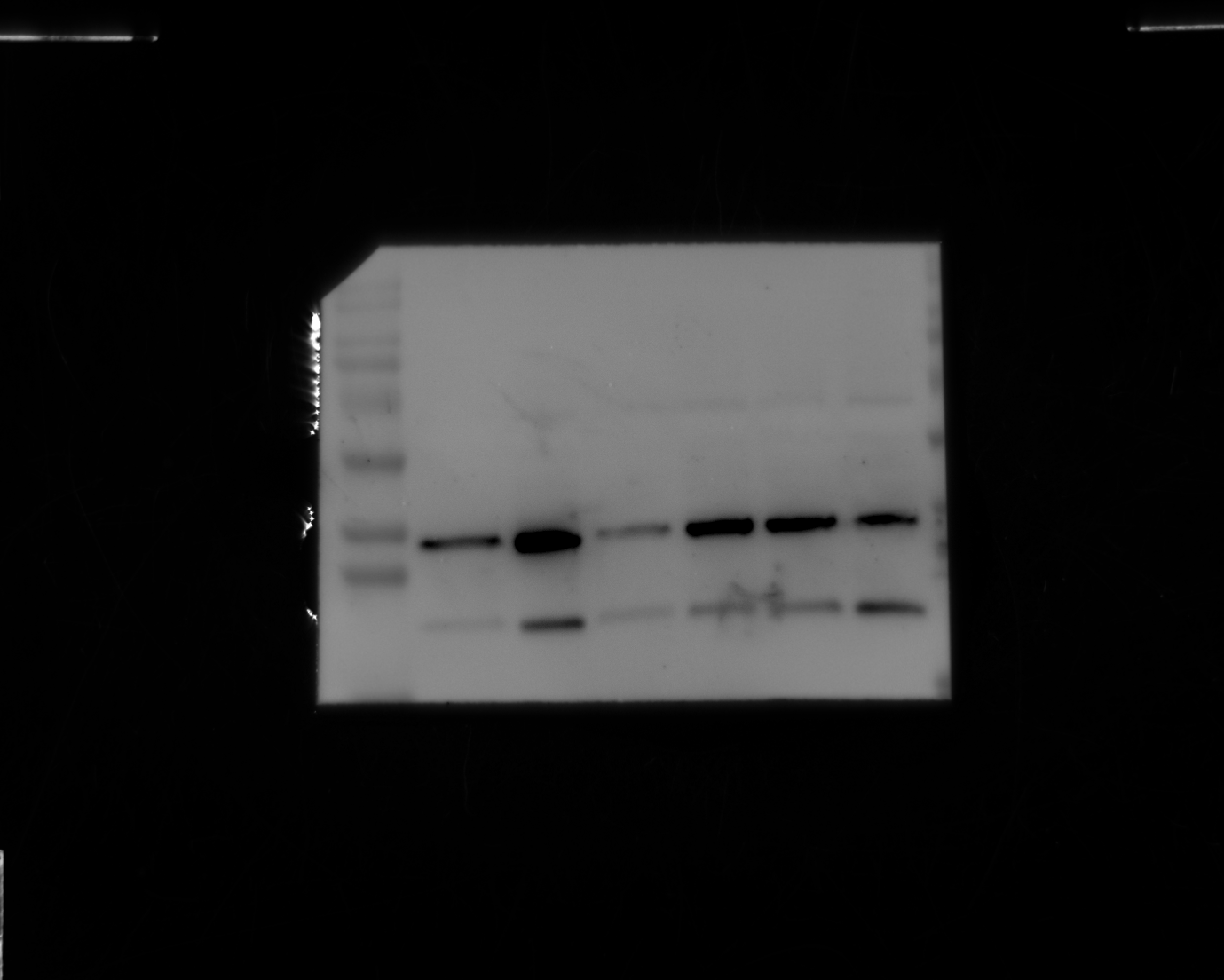

Supplement: Supplementary file 3 [file DataSheet1.zip › Supplementary_Raw_Blot/Supplementary_Raw_Blot_Fig9B_DRAM1---3.tif]

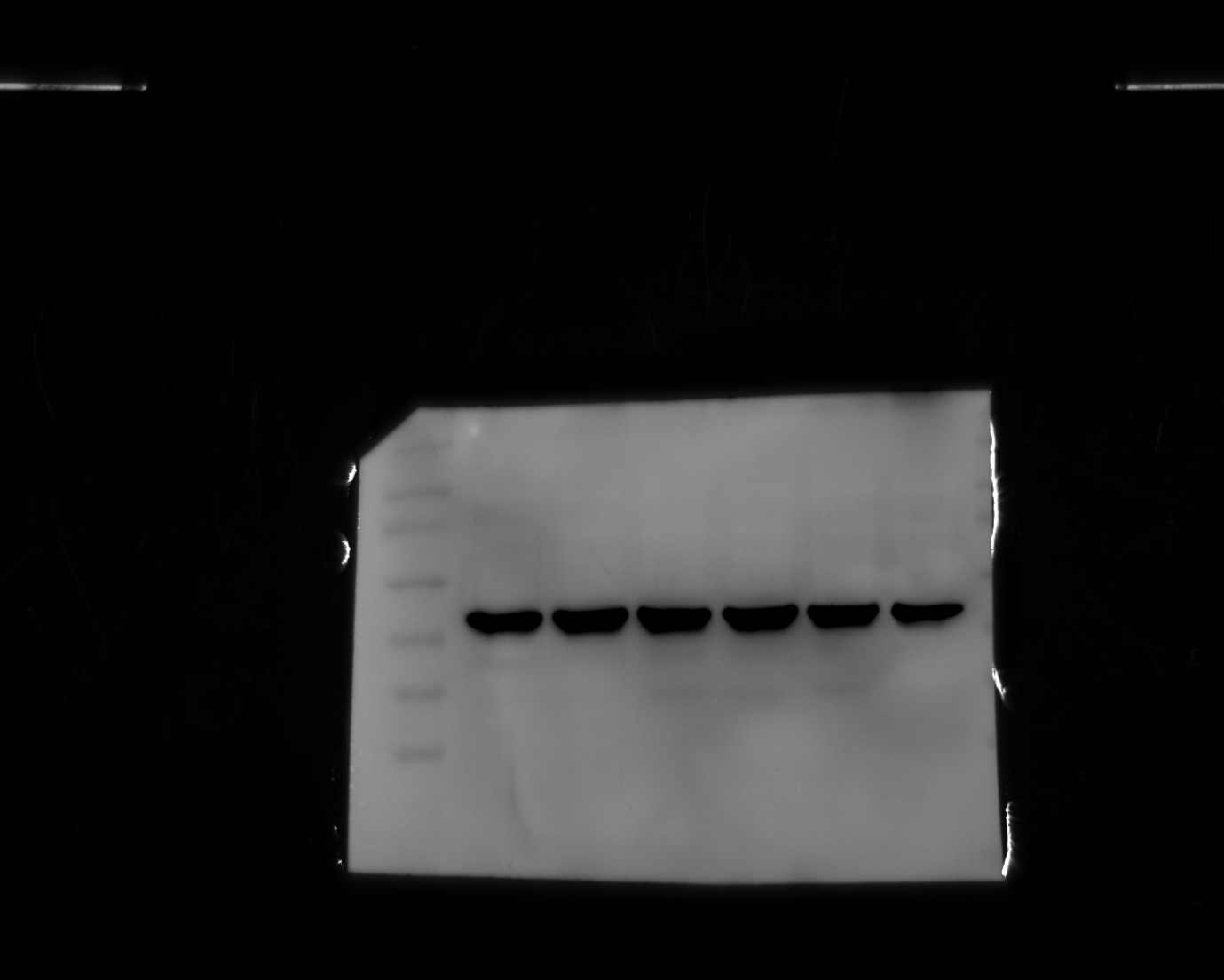

Supplement: Supplementary file 3 [file DataSheet1.zip › Supplementary_Raw_Blot/Supplementary_Raw_Blot_Fig9B_β-actin---1.tif]

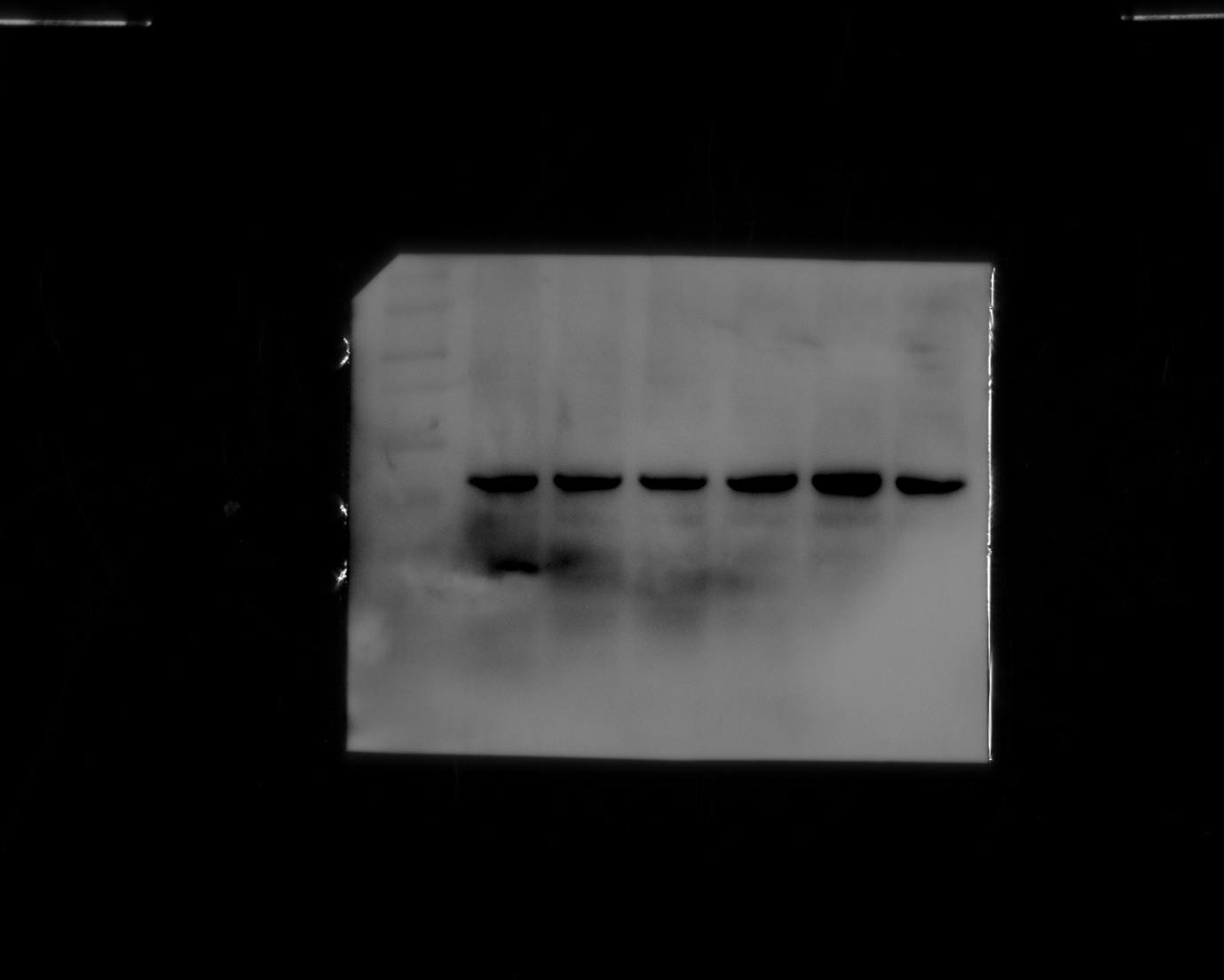

Supplement: Supplementary file 3 [file DataSheet1.zip › Supplementary_Raw_Blot/Supplementary_Raw_Blot_Fig9B_β-actin---2.tif]

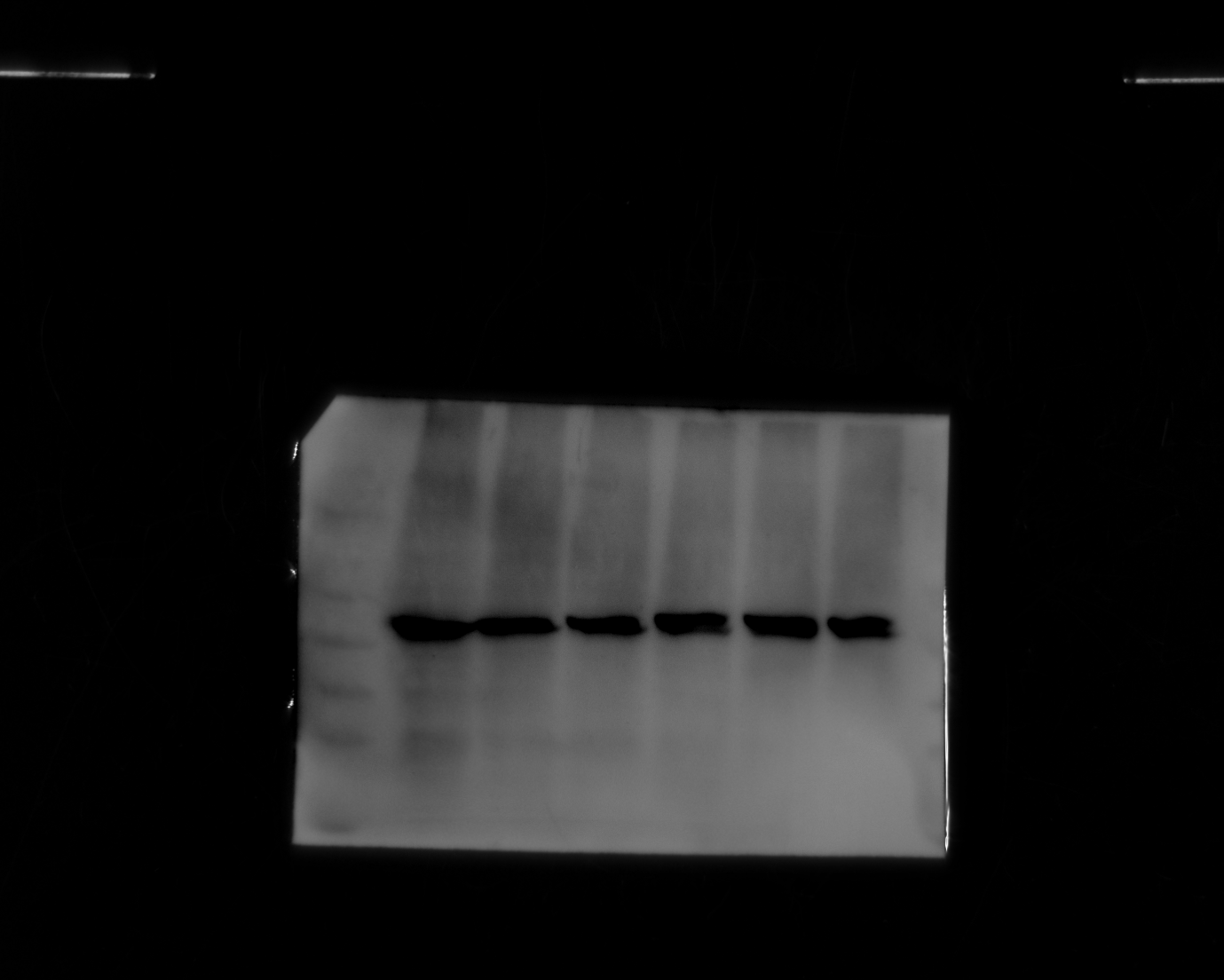

Supplement: Supplementary file 3 [file DataSheet1.zip › Supplementary_Raw_Blot/Supplementary_Raw_Blot_Fig9B_β-actin---3.tif]

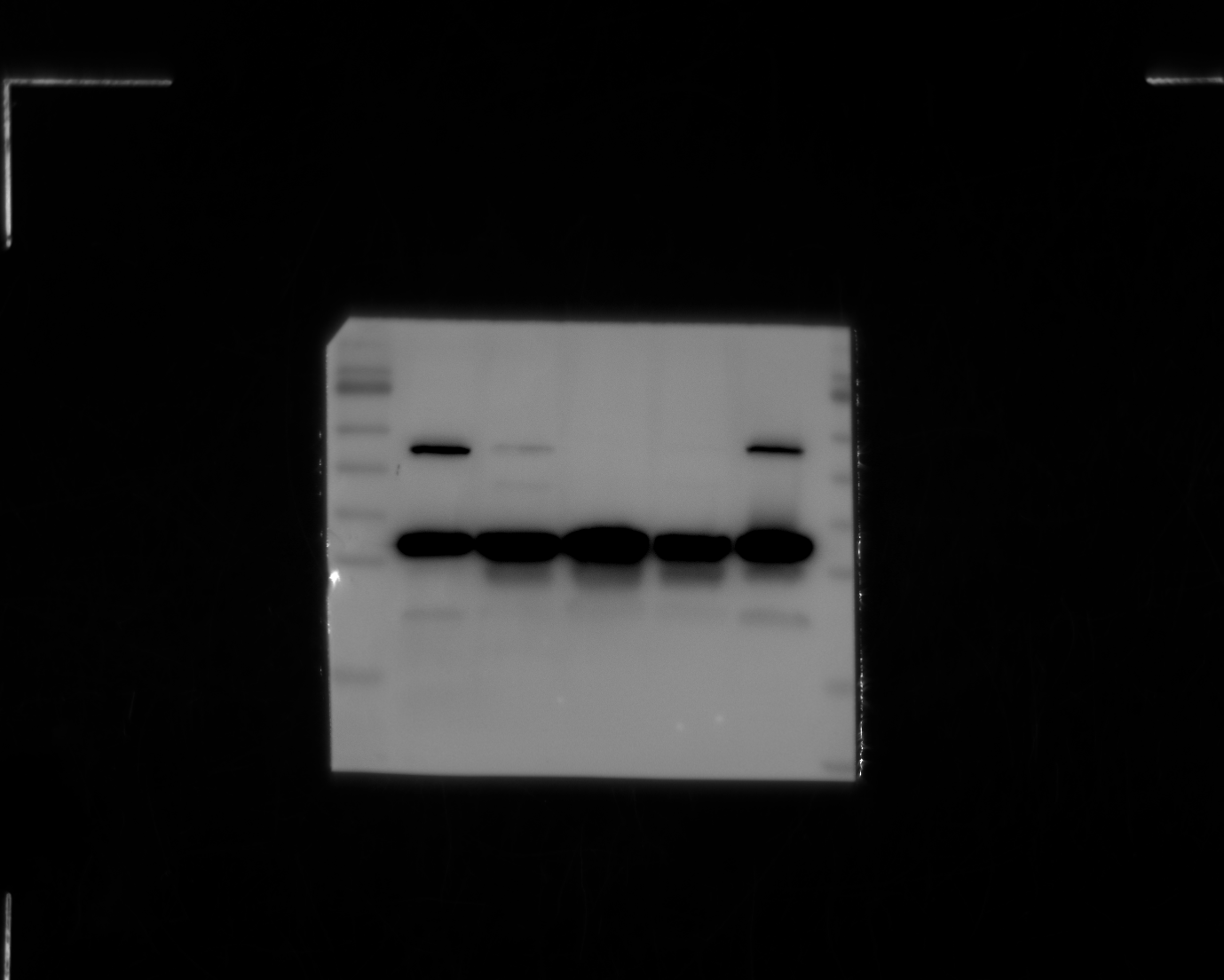

Supplement: Supplementary file 3 [file DataSheet1.zip › Supplementary_Raw_Blot/Supplementary_Raw_Blot_Fig9D_DRAM1---1---(black background).tif]

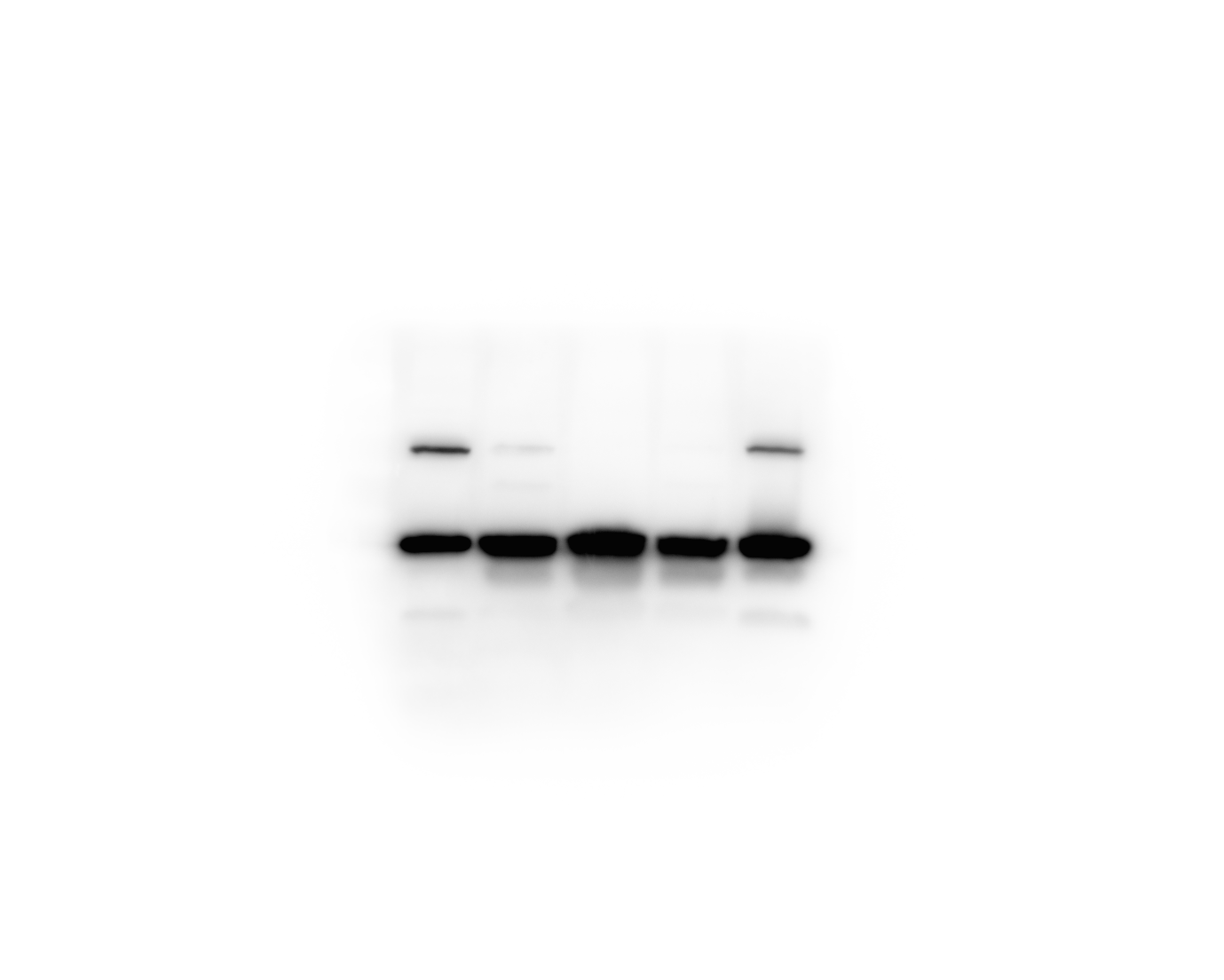

Supplement: Supplementary file 3 [file DataSheet1.zip › Supplementary_Raw_Blot/Supplementary_Raw_Blot_Fig9D_DRAM1---1---(white background).tif]

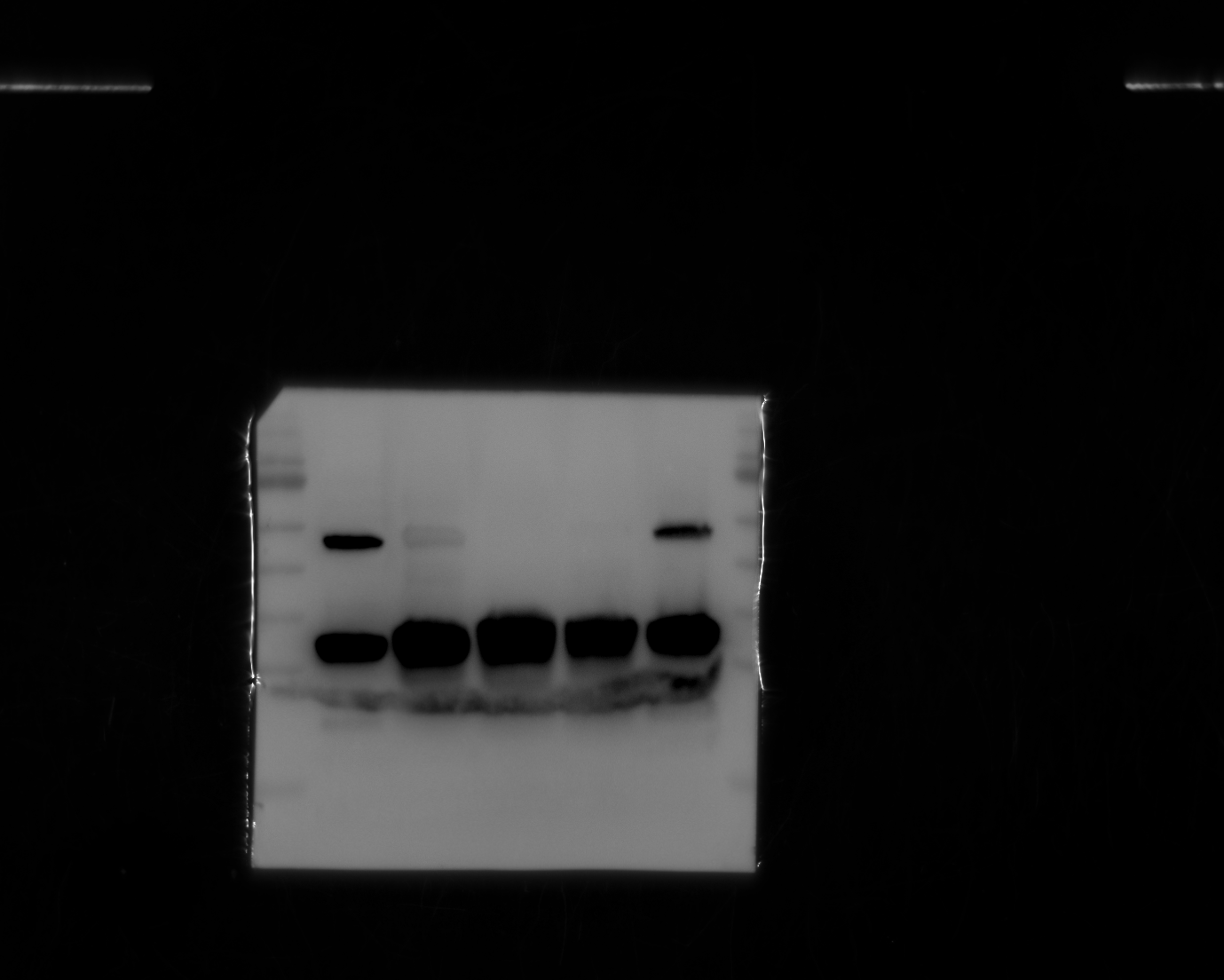

Supplement: Supplementary file 3 [file DataSheet1.zip › Supplementary_Raw_Blot/Supplementary_Raw_Blot_Fig9D_DRAM1---2---(black background).tif]

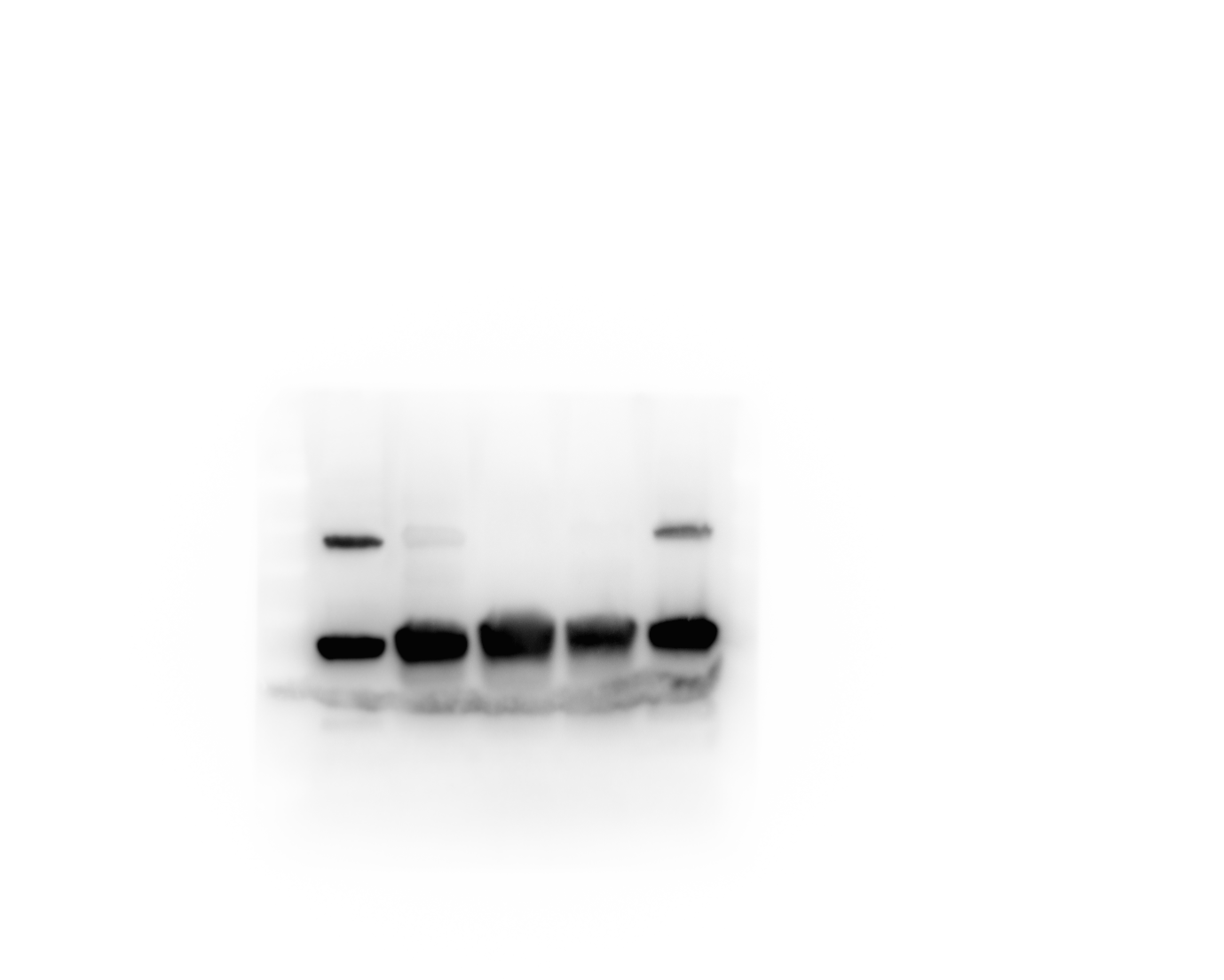

Supplement: Supplementary file 3 [file DataSheet1.zip › Supplementary_Raw_Blot/Supplementary_Raw_Blot_Fig9D_DRAM1---2---(white background).tif]

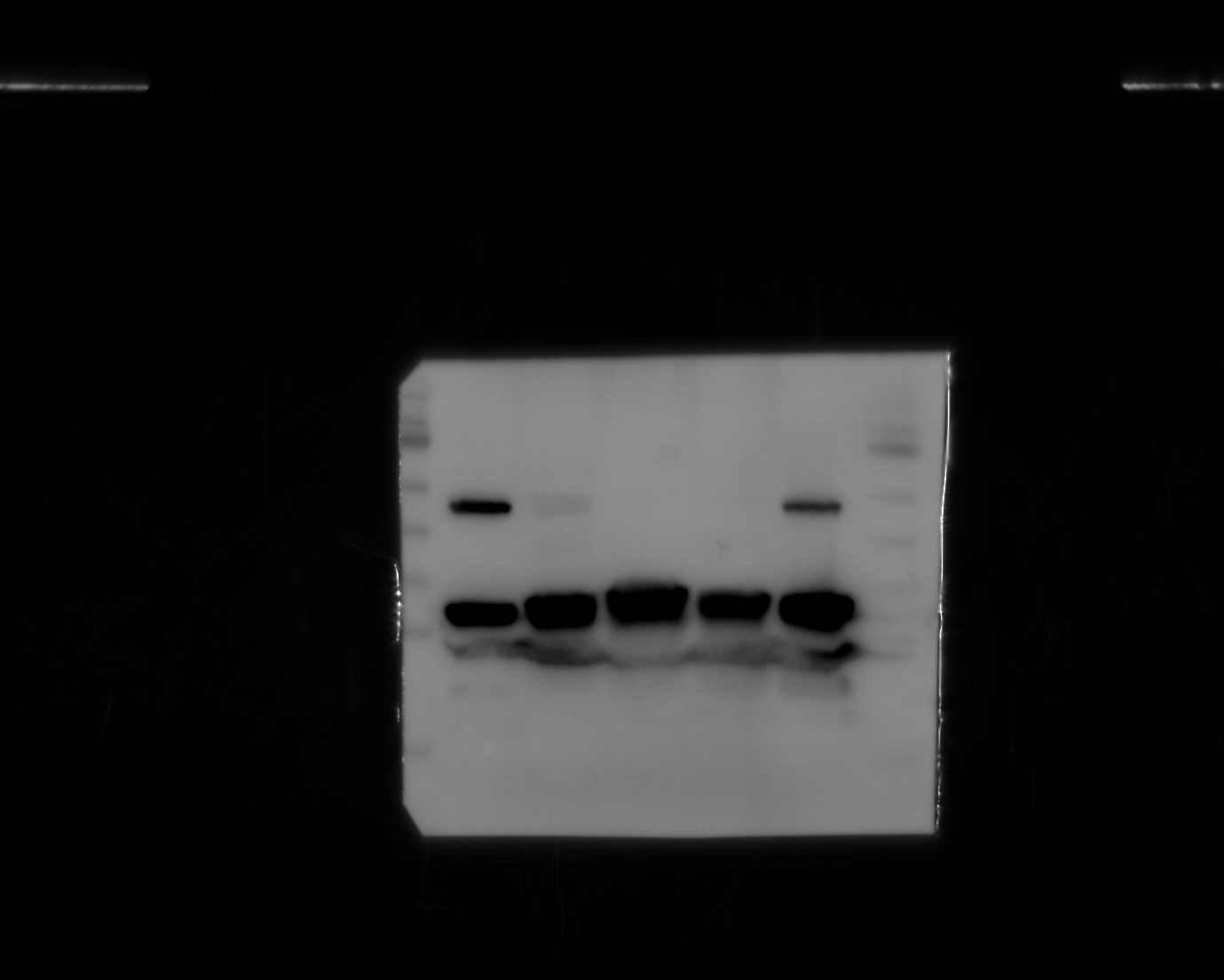

Supplement: Supplementary file 3 [file DataSheet1.zip › Supplementary_Raw_Blot/Supplementary_Raw_Blot_Fig9D_DRAM1---3---(black background).tif]

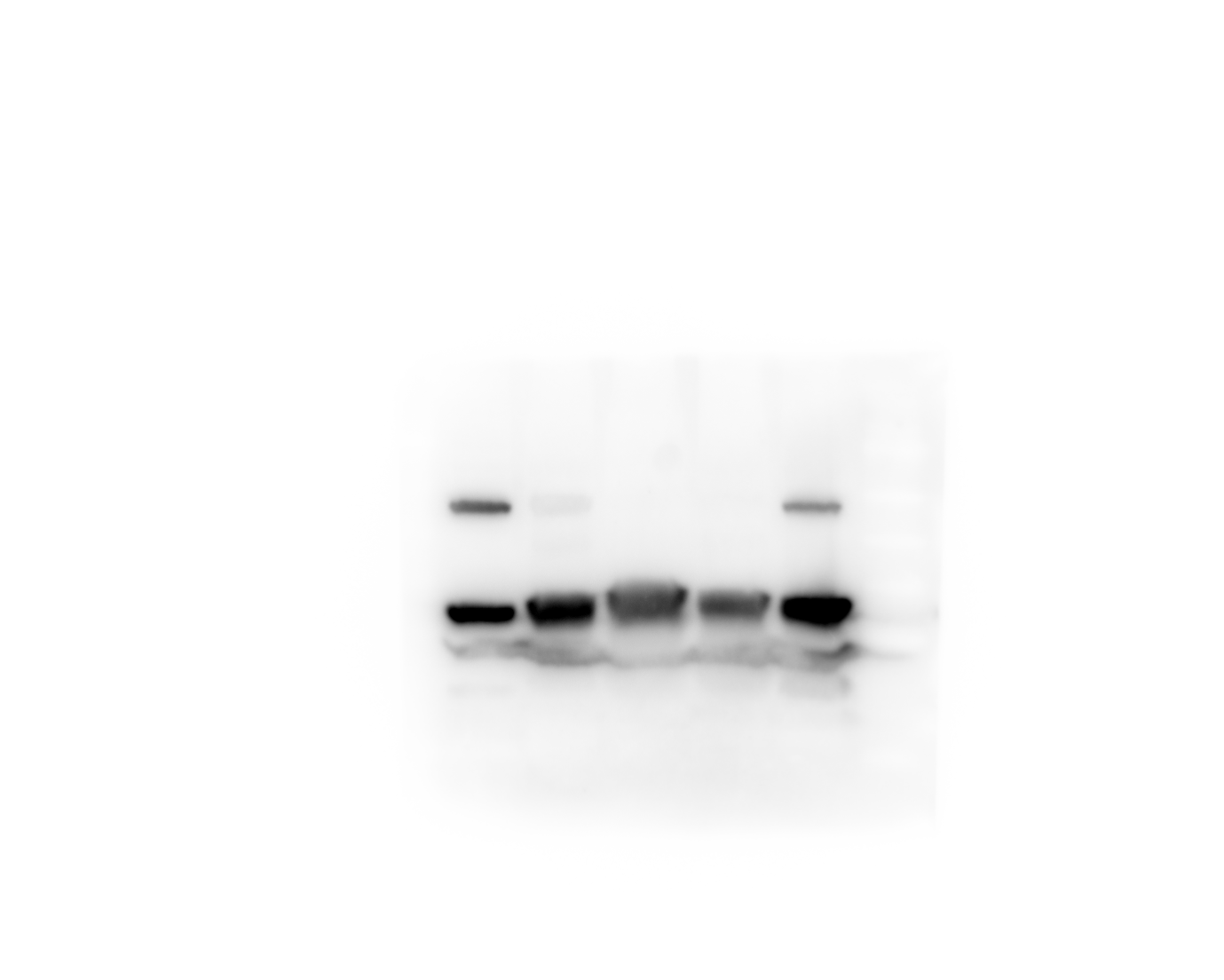

Supplement: Supplementary file 3 [file DataSheet1.zip › Supplementary_Raw_Blot/Supplementary_Raw_Blot_Fig9D_DRAM1---3---(white background).tif]

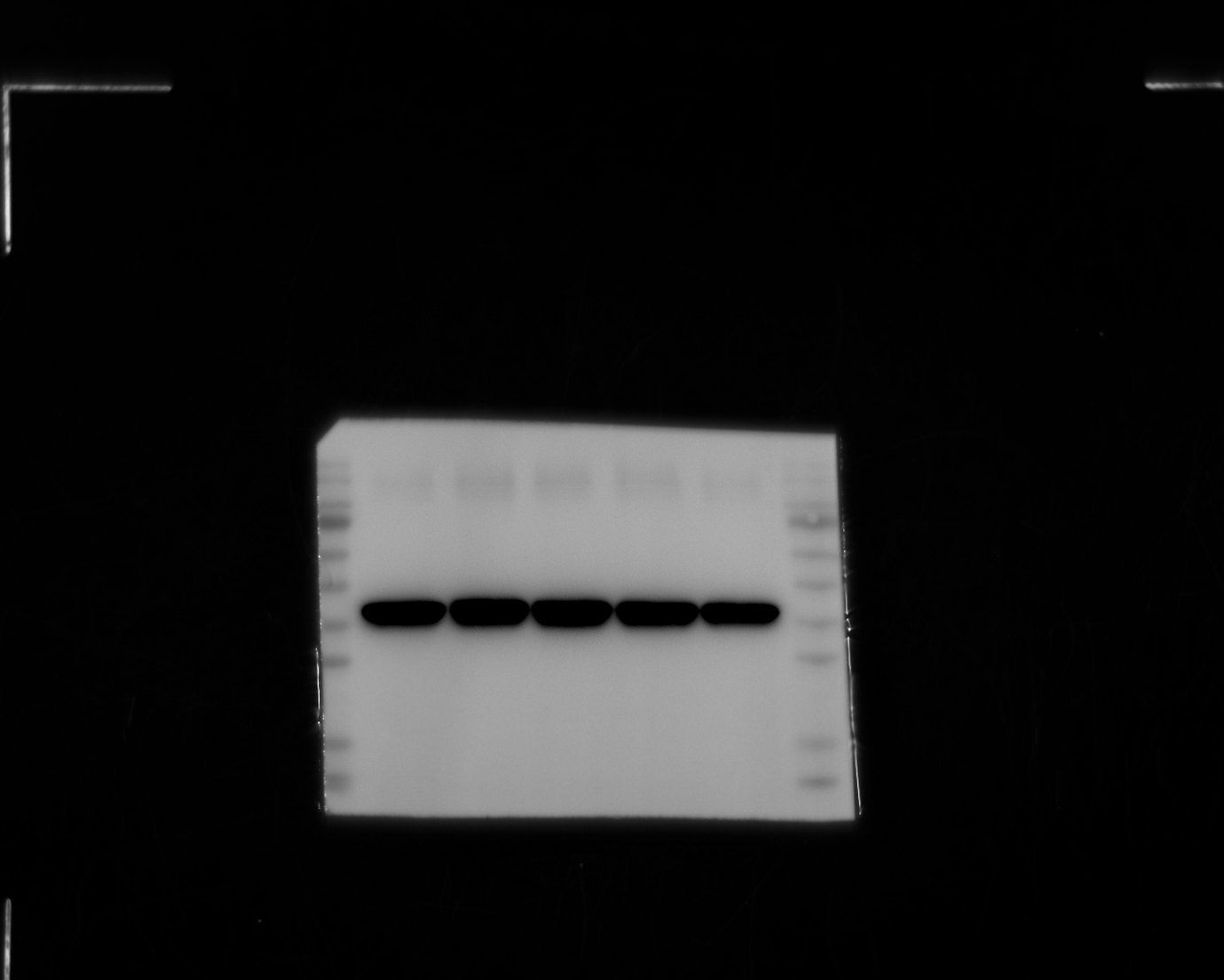

Supplement: Supplementary file 3 [file DataSheet1.zip › Supplementary_Raw_Blot/Supplementary_Raw_Blot_Fig9D_GAPDH---1---(black background).tif]

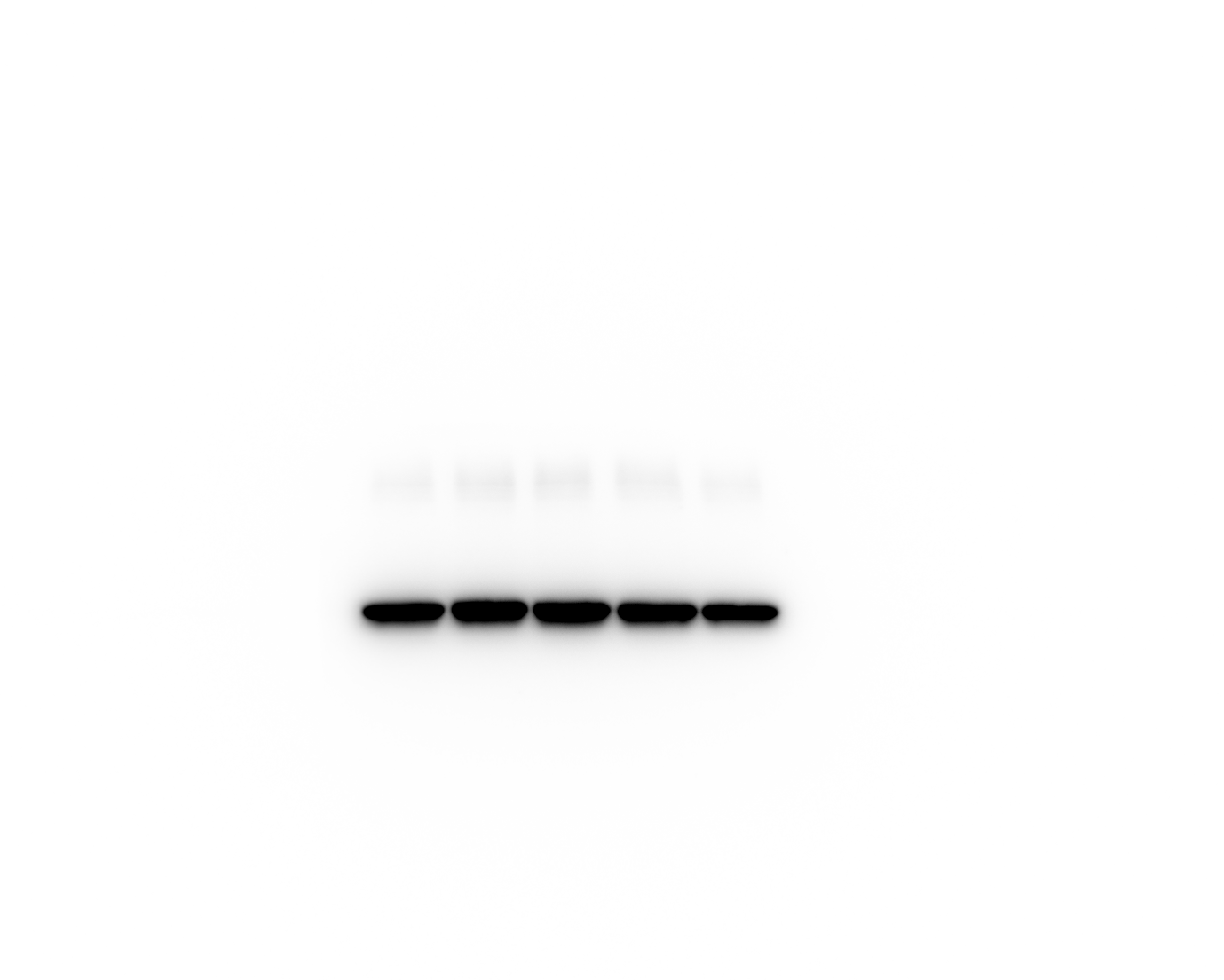

Supplement: Supplementary file 3 [file DataSheet1.zip › Supplementary_Raw_Blot/Supplementary_Raw_Blot_Fig9D_GAPDH---1---(white background).tif]

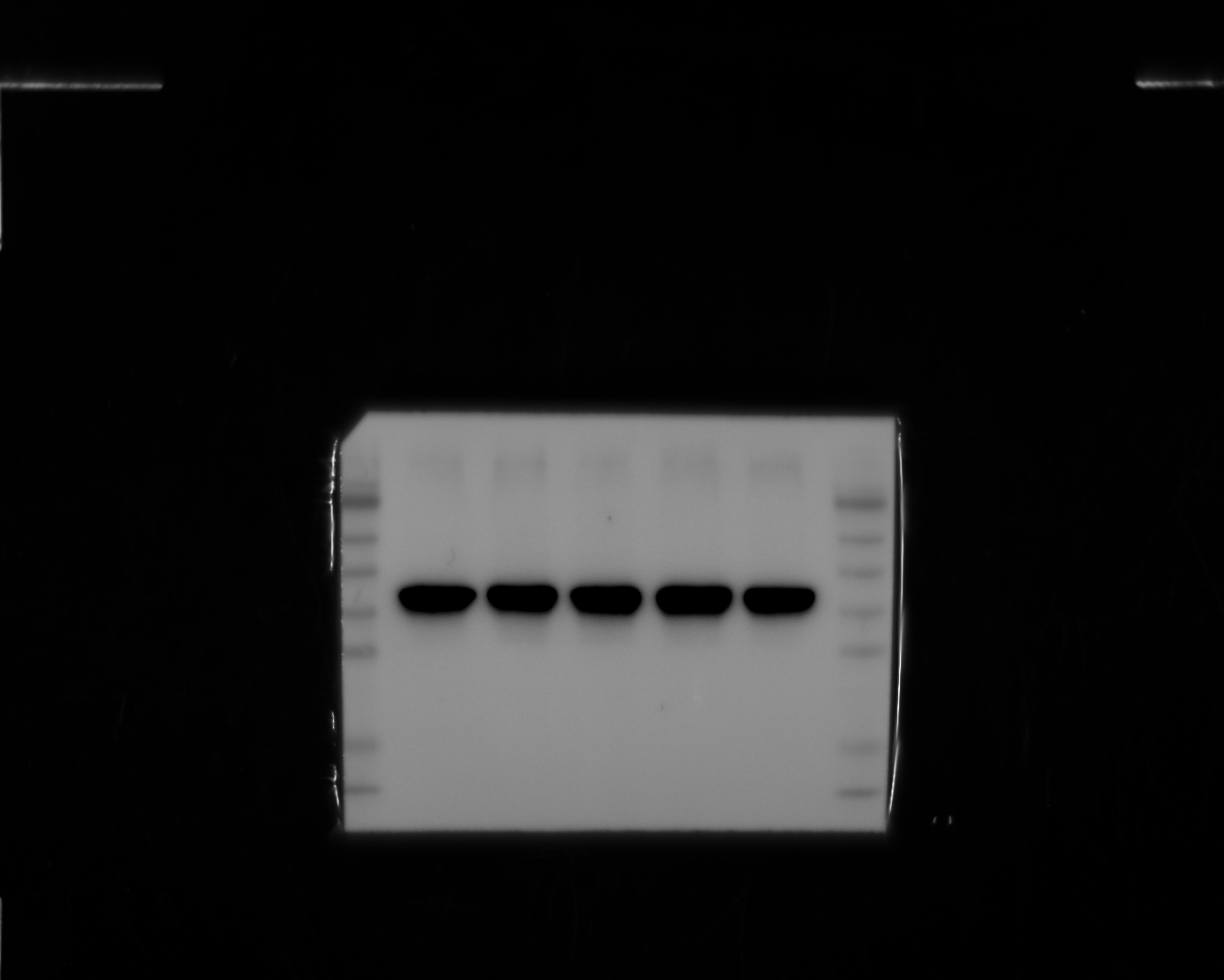

Supplement: Supplementary file 3 [file DataSheet1.zip › Supplementary_Raw_Blot/Supplementary_Raw_Blot_Fig9D_GAPDH---2---(black background).tif]

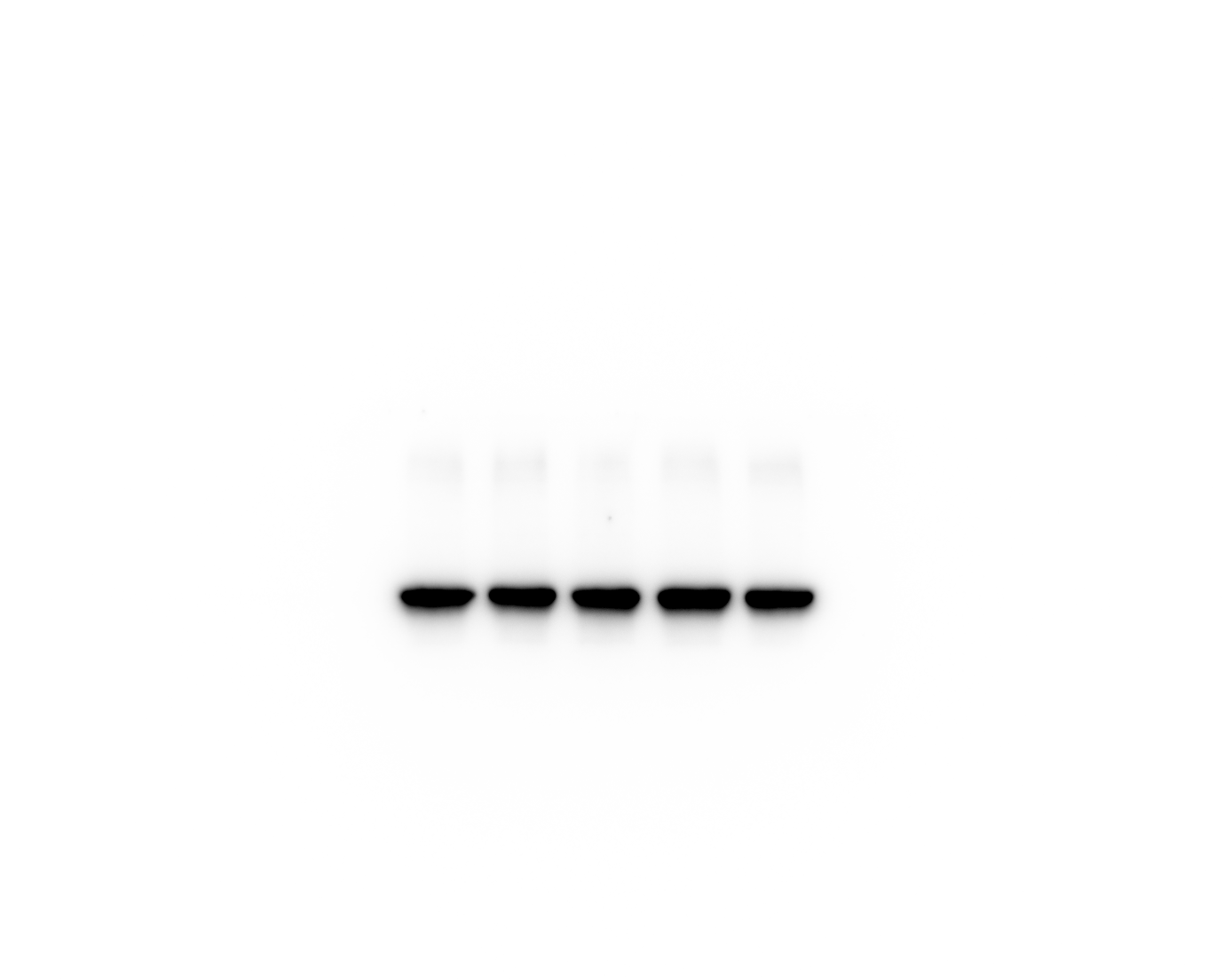

Supplement: Supplementary file 3 [file DataSheet1.zip › Supplementary_Raw_Blot/Supplementary_Raw_Blot_Fig9D_GAPDH---2---(white background).tif]

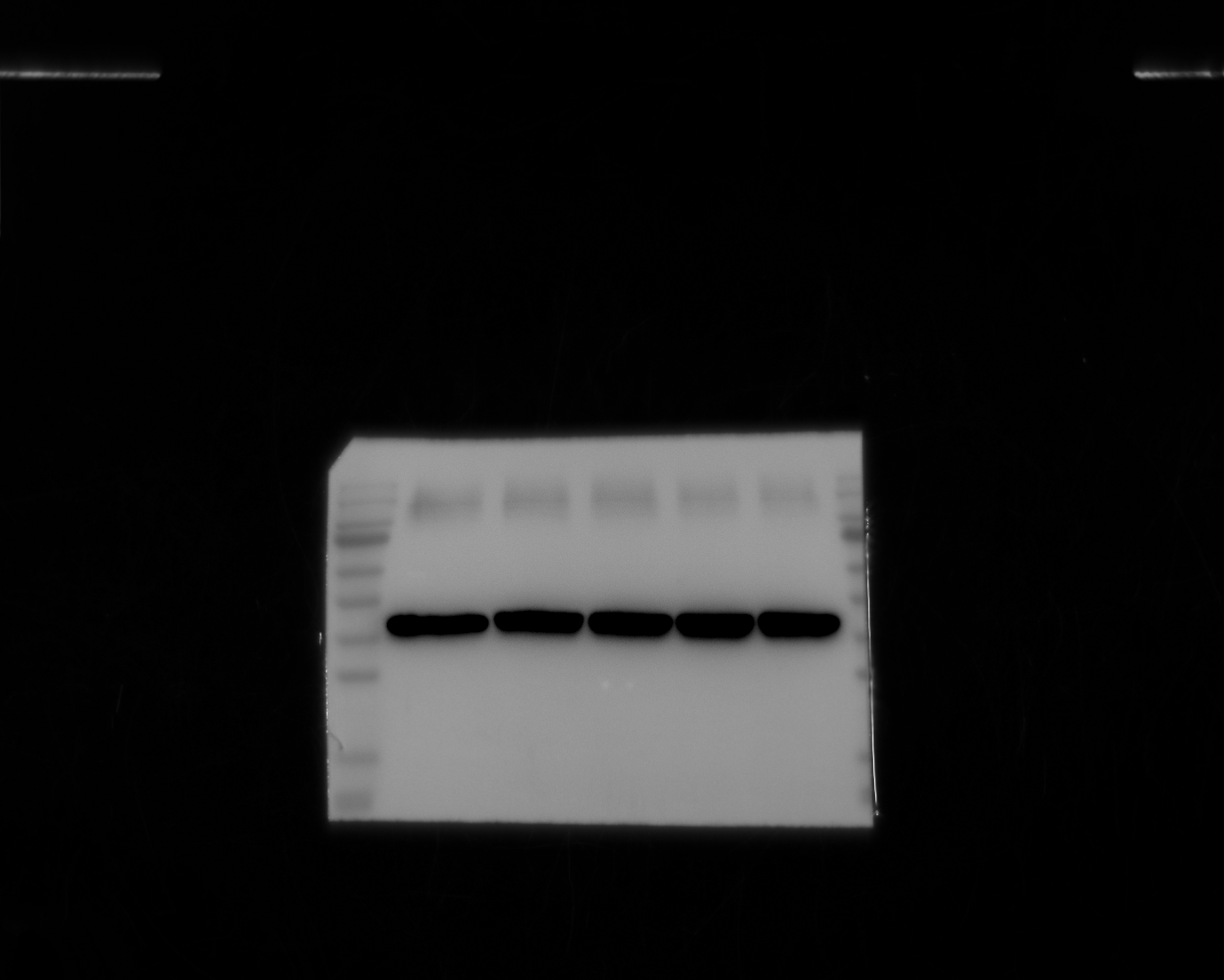

Supplement: Supplementary file 3 [file DataSheet1.zip › Supplementary_Raw_Blot/Supplementary_Raw_Blot_Fig9D_GAPDH---3---(black background).tif]

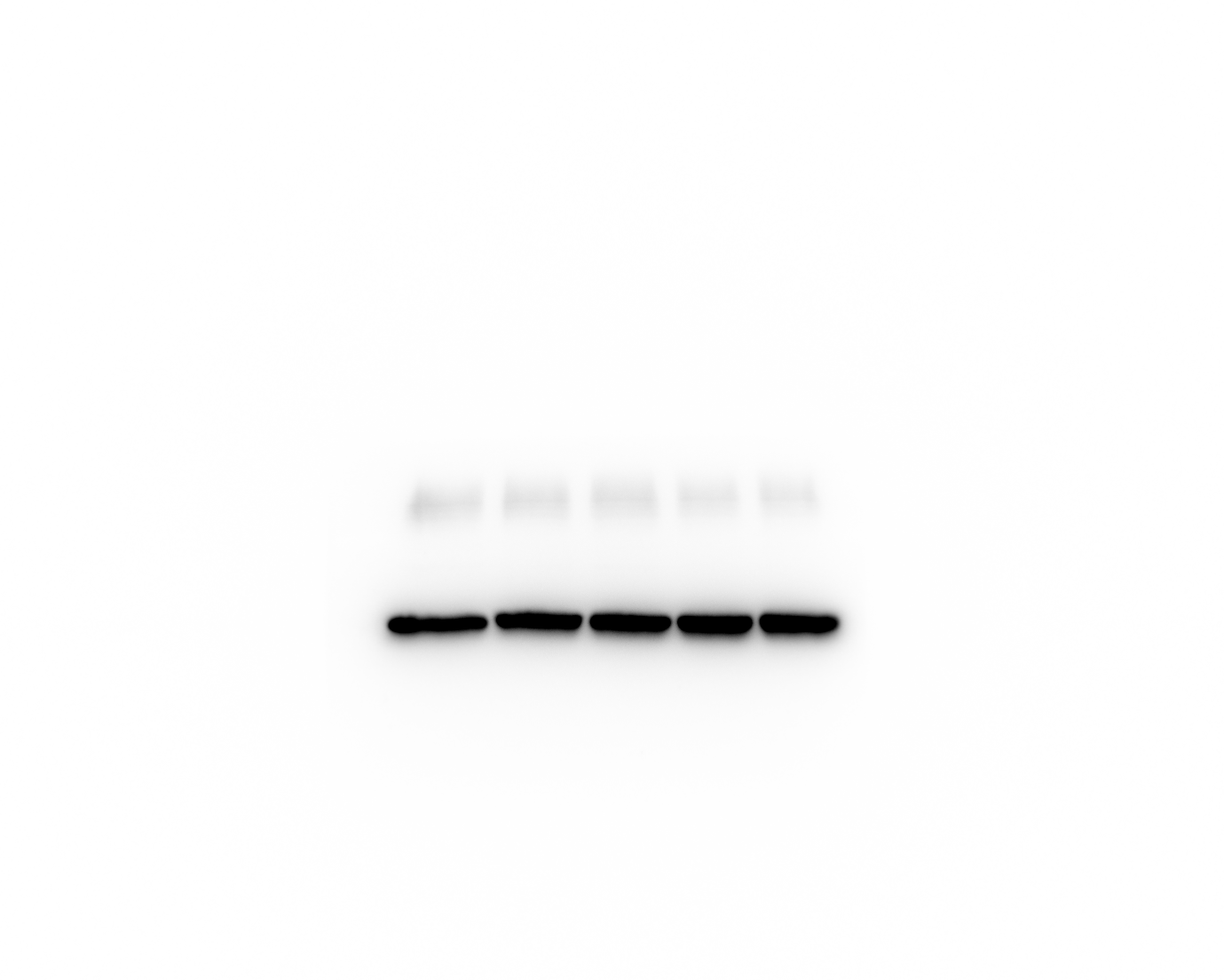

Supplement: Supplementary file 3 [file DataSheet1.zip › Supplementary_Raw_Blot/Supplementary_Raw_Blot_Fig9D_GAPDH---3---(white background).tif]

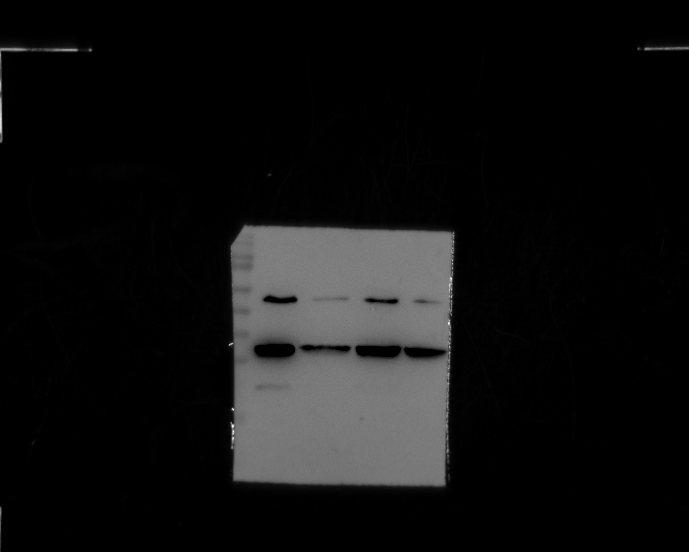

Supplement: Supplementary file 3 [file DataSheet1.zip › Supplementary_Raw_Blot/Supplementary_Raw_Blot_Fig9F_DRAM1---1---(black background).tif]

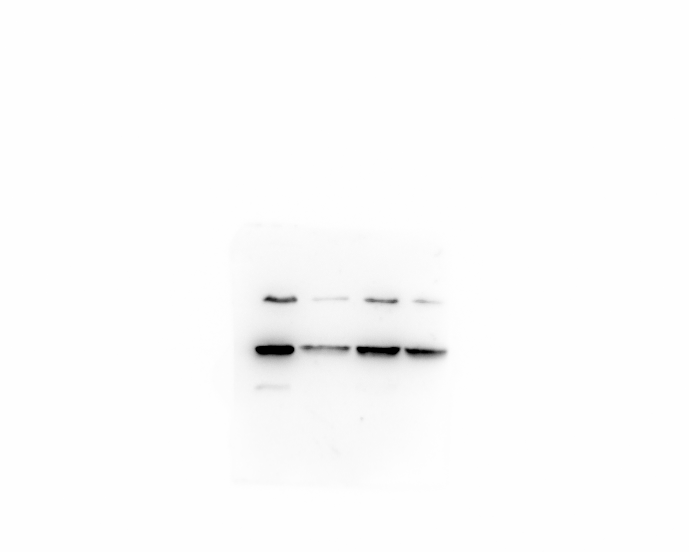

Supplement: Supplementary file 3 [file DataSheet1.zip › Supplementary_Raw_Blot/Supplementary_Raw_Blot_Fig9F_DRAM1---1---(white background).tif]

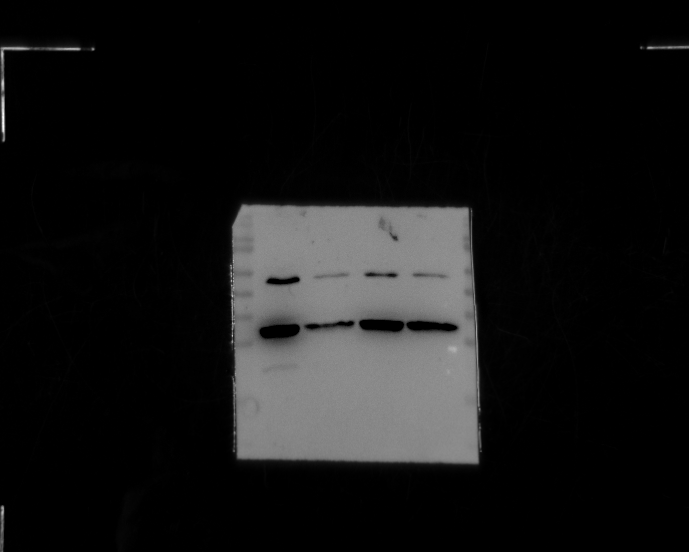

Supplement: Supplementary file 3 [file DataSheet1.zip › Supplementary_Raw_Blot/Supplementary_Raw_Blot_Fig9F_DRAM1---2---(black background).tif]

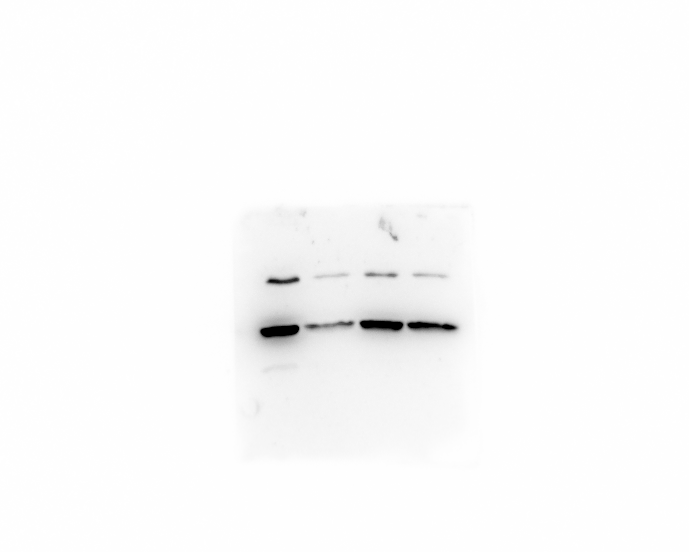

Supplement: Supplementary file 3 [file DataSheet1.zip › Supplementary_Raw_Blot/Supplementary_Raw_Blot_Fig9F_DRAM1---2---(white background).tif]

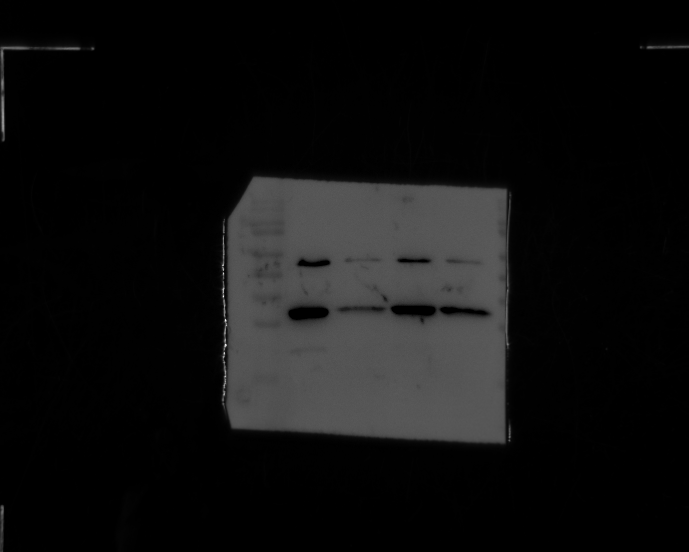

Supplement: Supplementary file 3 [file DataSheet1.zip › Supplementary_Raw_Blot/Supplementary_Raw_Blot_Fig9F_DRAM1---3---(black background).tif]

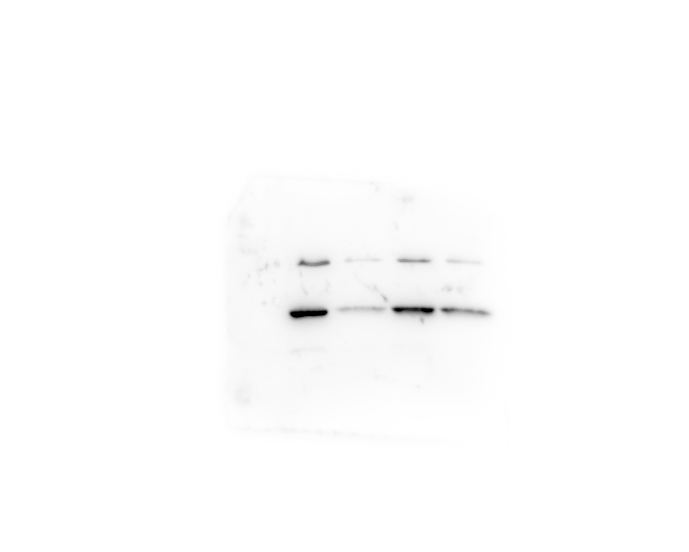

Supplement: Supplementary file 3 [file DataSheet1.zip › Supplementary_Raw_Blot/Supplementary_Raw_Blot_Fig9F_DRAM1---3---(white background).tif]

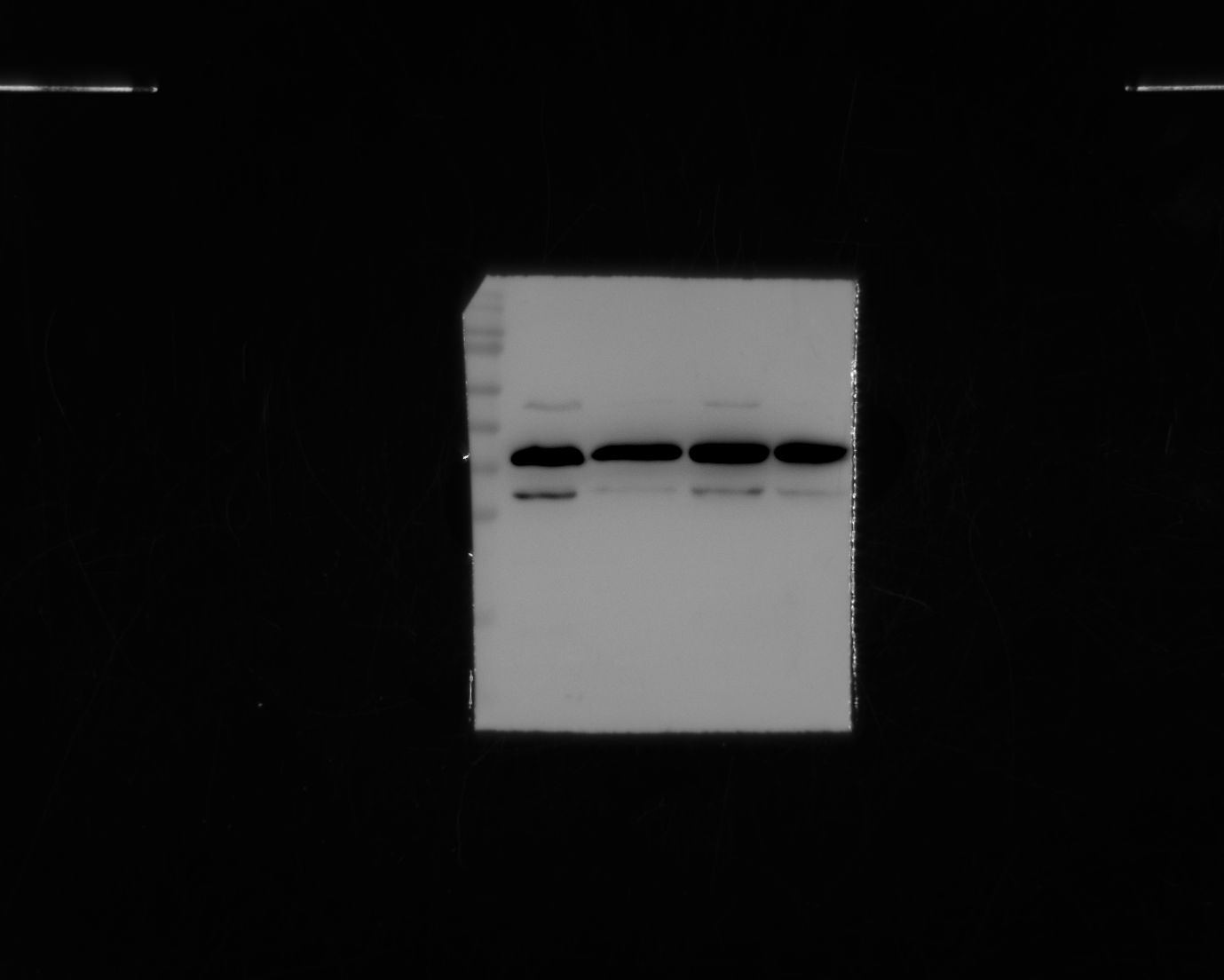

Supplement: Supplementary file 3 [file DataSheet1.zip › Supplementary_Raw_Blot/Supplementary_Raw_Blot_Fig9F_GAPDH---1---(black background).tif]

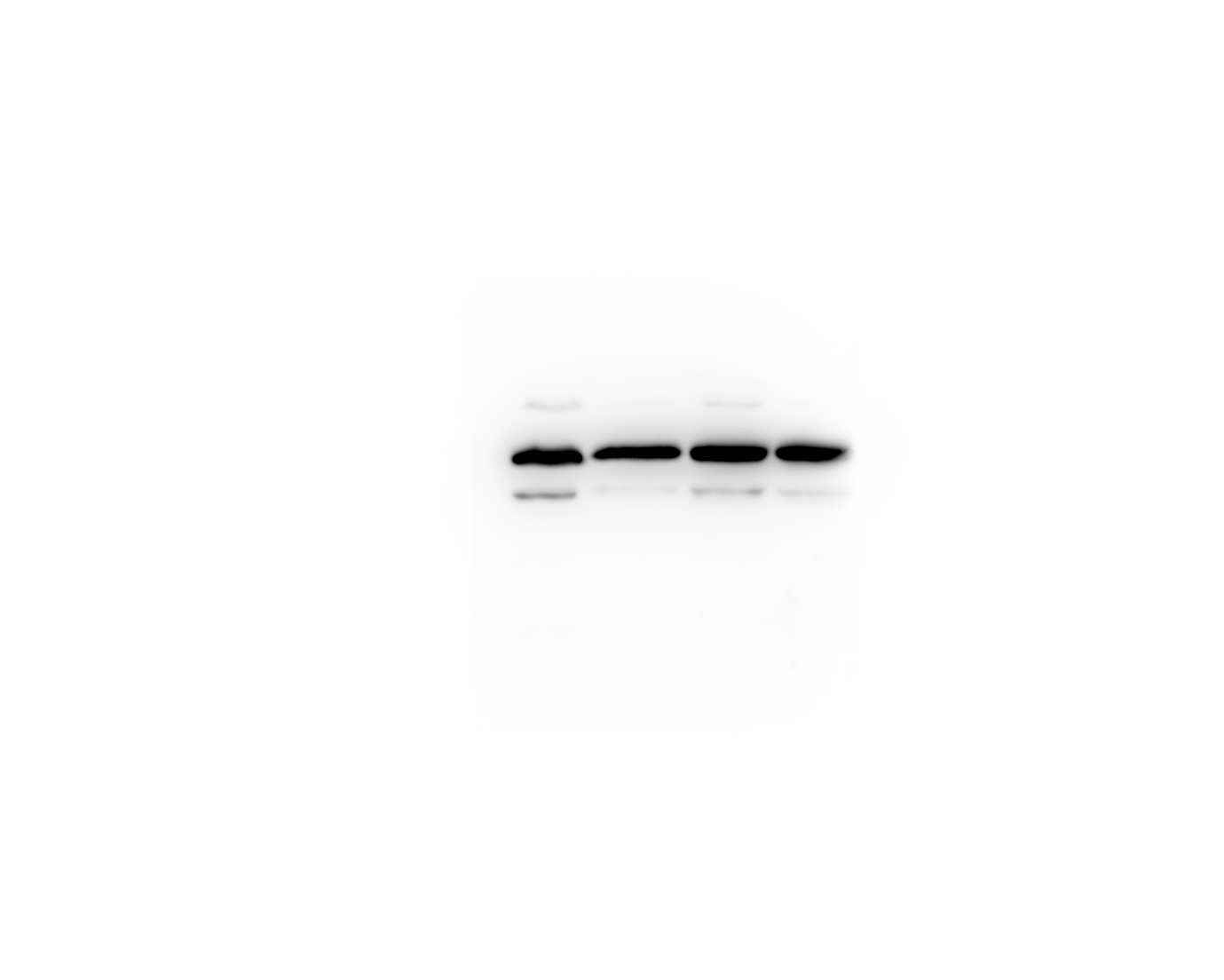

Supplement: Supplementary file 3 [file DataSheet1.zip › Supplementary_Raw_Blot/Supplementary_Raw_Blot_Fig9F_GAPDH---1---(white background).tif]

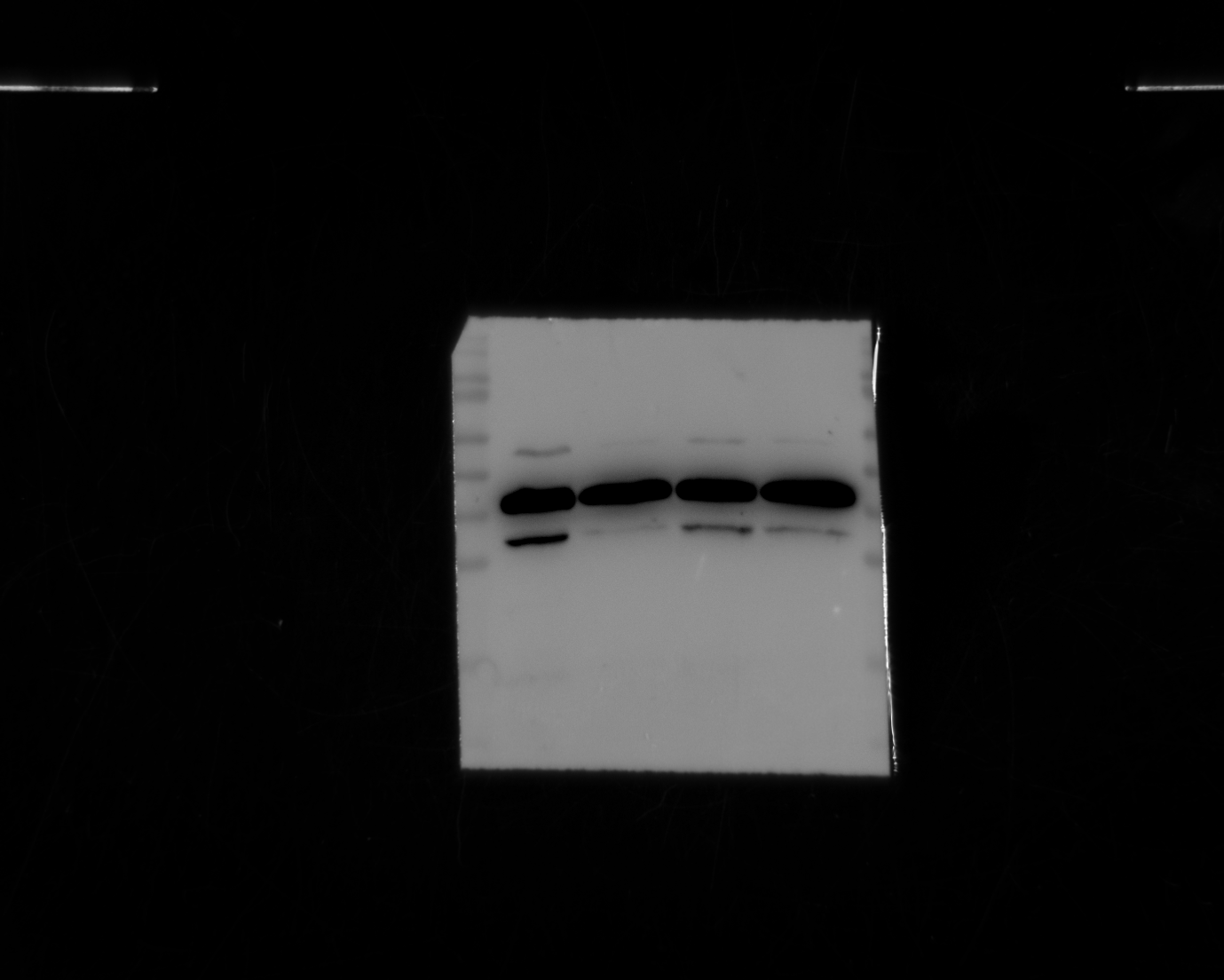

Supplement: Supplementary file 3 [file DataSheet1.zip › Supplementary_Raw_Blot/Supplementary_Raw_Blot_Fig9F_GAPDH---2---(black background).tif]

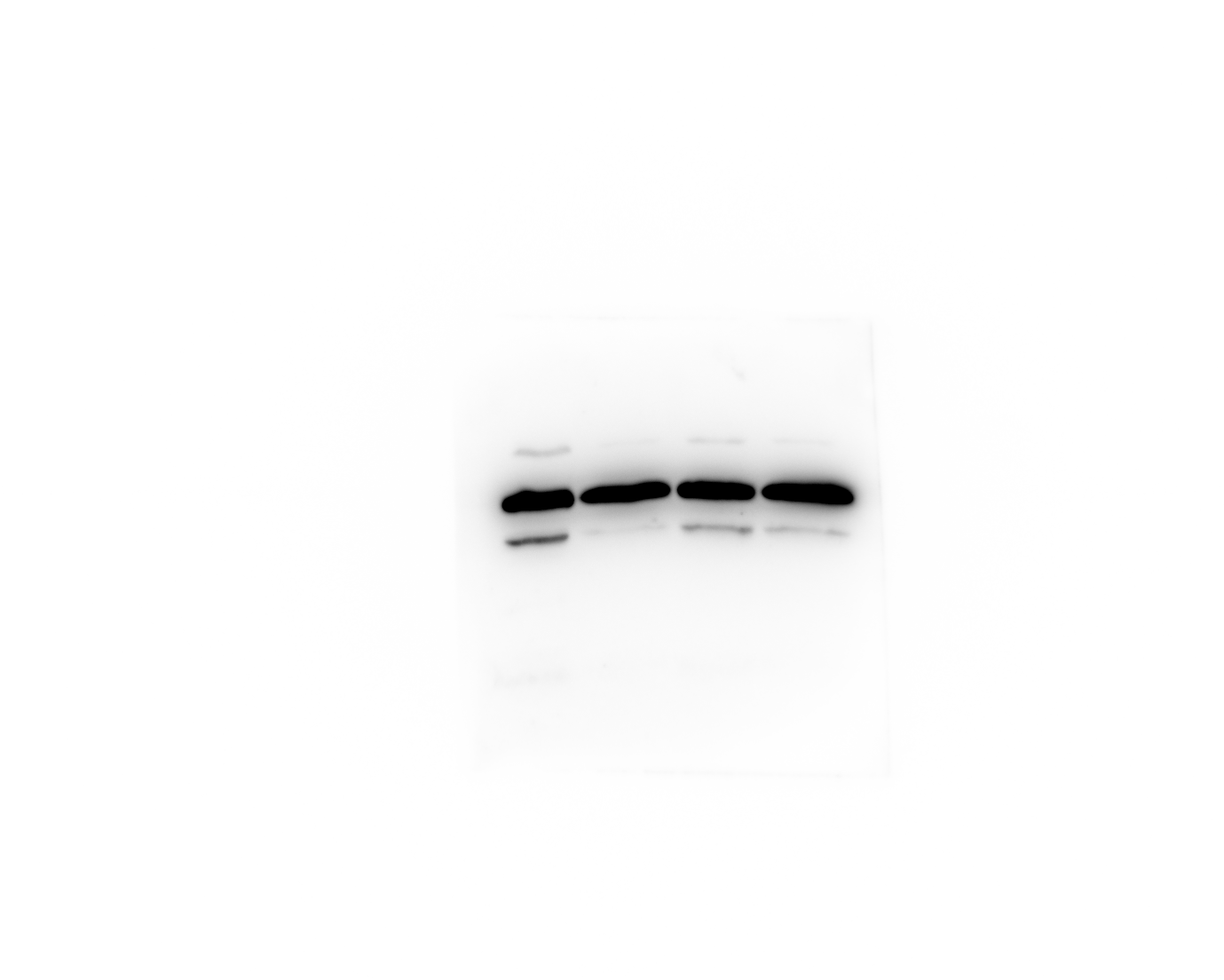

Supplement: Supplementary file 3 [file DataSheet1.zip › Supplementary_Raw_Blot/Supplementary_Raw_Blot_Fig9F_GAPDH---2---(white background).tif]

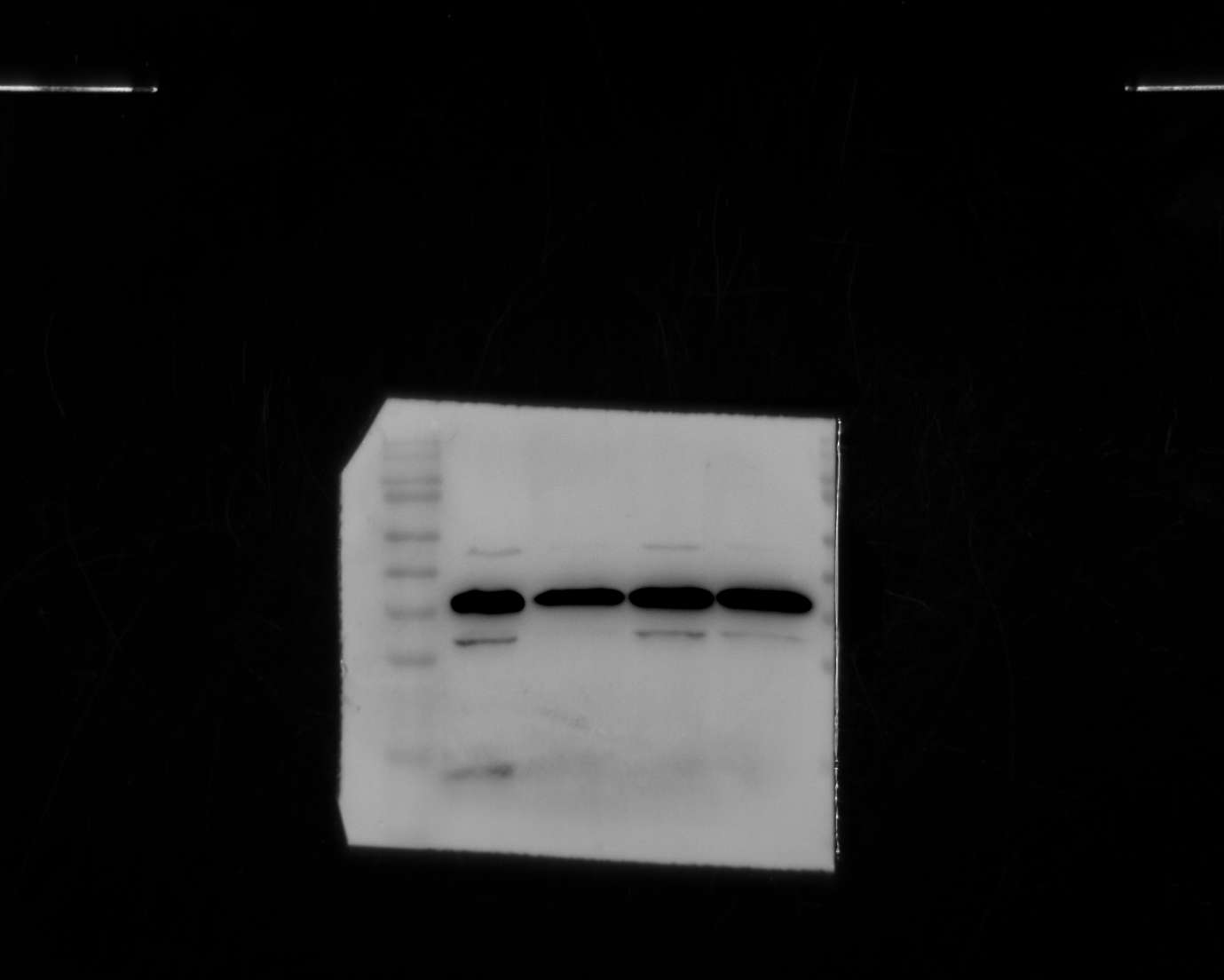

Supplement: Supplementary file 3 [file DataSheet1.zip › Supplementary_Raw_Blot/Supplementary_Raw_Blot_Fig9F_GAPDH---3---(black background).tif]

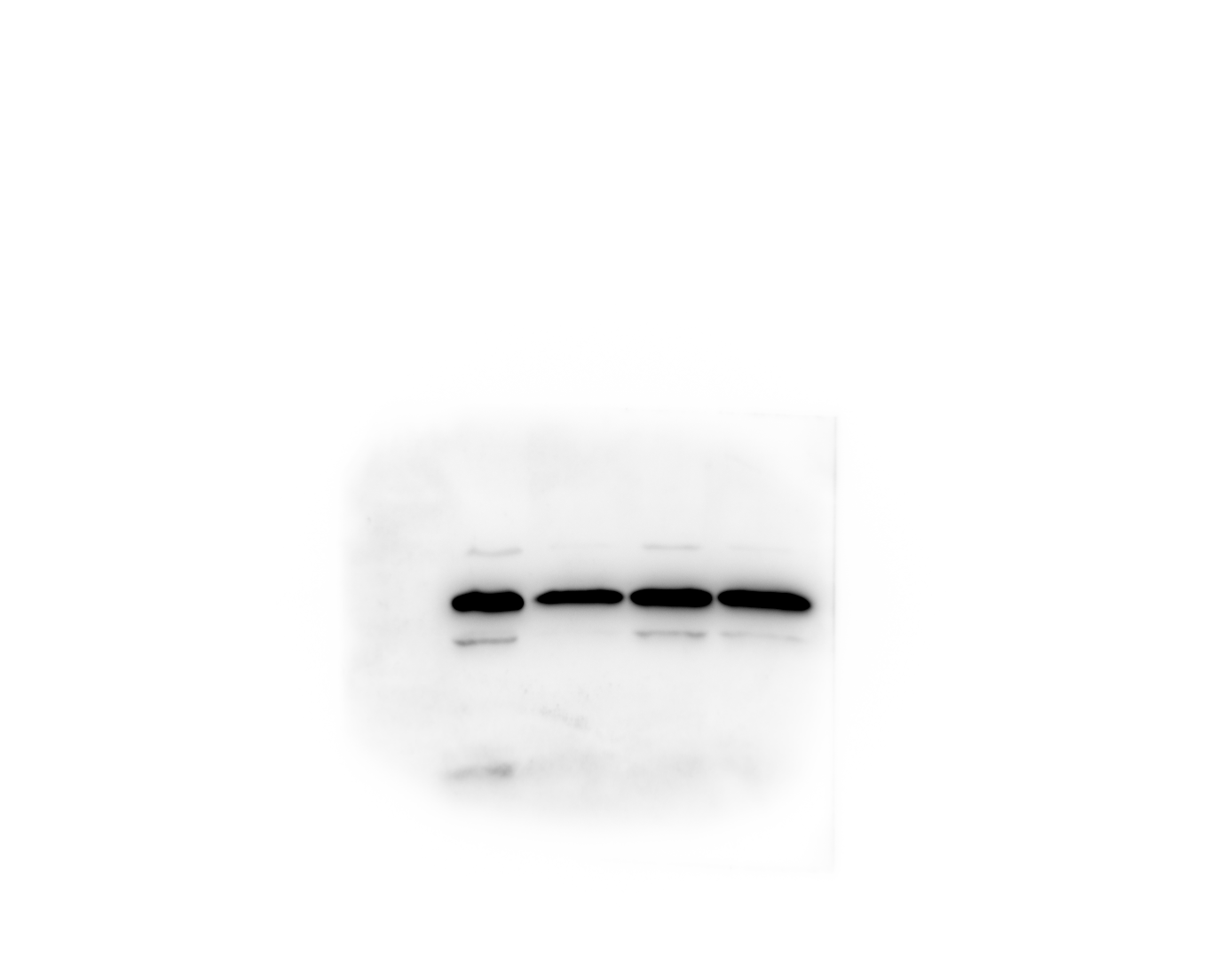

Supplement: Supplementary file 3 [file DataSheet1.zip › Supplementary_Raw_Blot/Supplementary_Raw_Blot_Fig9F_GAPDH---3---(white background).tif]

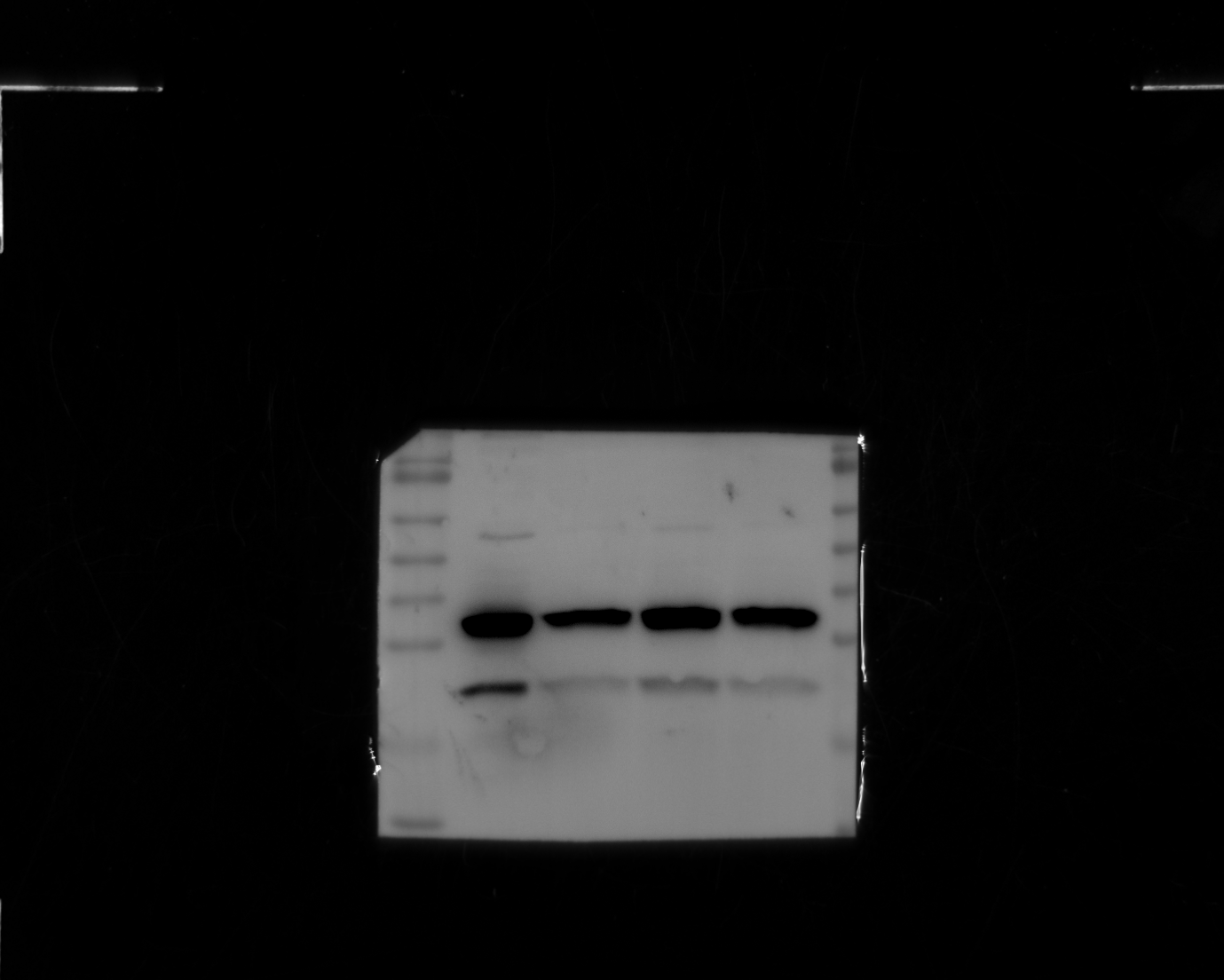

Supplement: Supplementary file 3 [file DataSheet1.zip › Supplementary_Raw_Blot/Supplementary_Raw_Blot_Fig9H_DRAM1---1---(black background).tif]

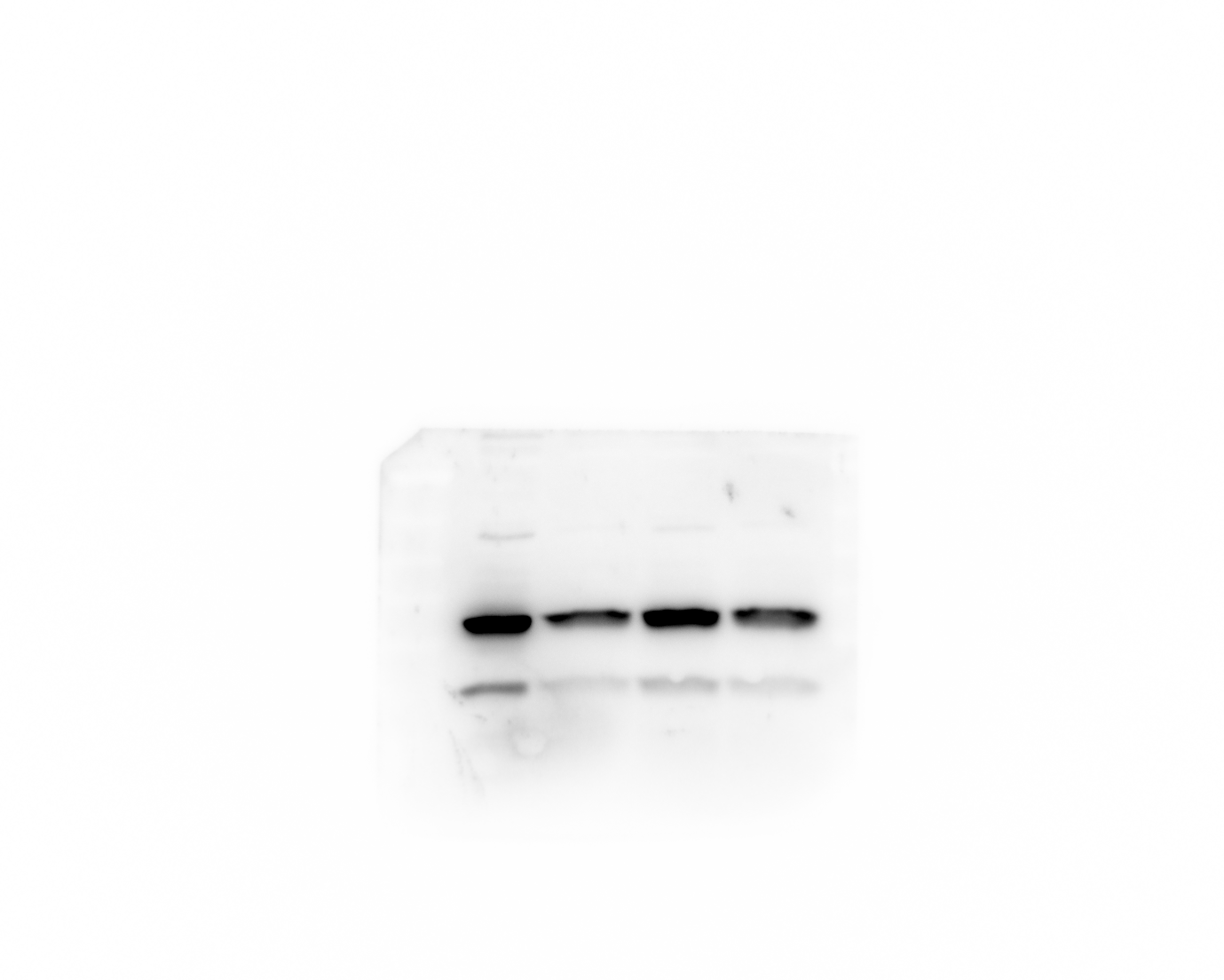

Supplement: Supplementary file 3 [file DataSheet1.zip › Supplementary_Raw_Blot/Supplementary_Raw_Blot_Fig9H_DRAM1---1---(white background).tif]

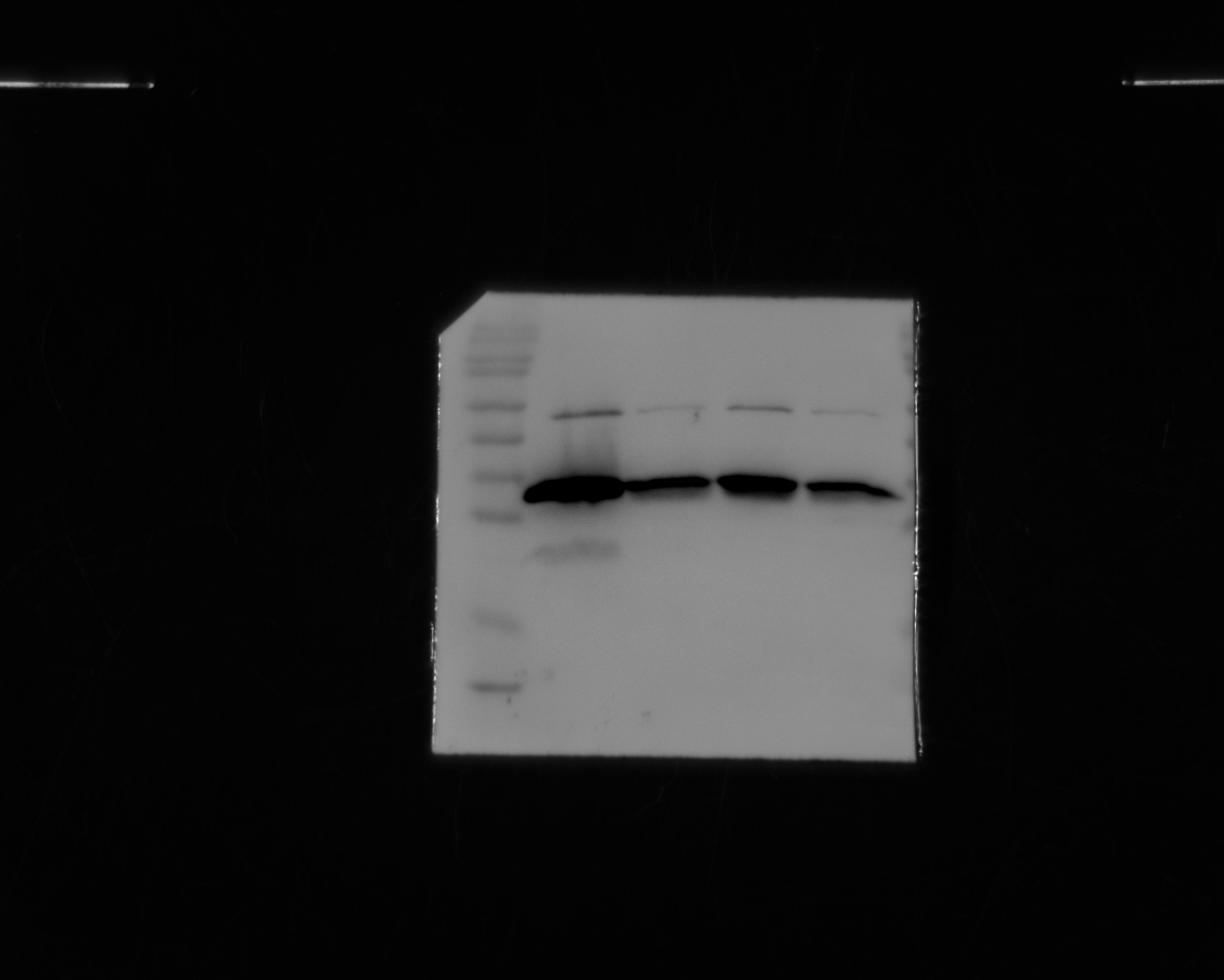

Supplement: Supplementary file 3 [file DataSheet1.zip › Supplementary_Raw_Blot/Supplementary_Raw_Blot_Fig9H_DRAM1---2---(black background).tif]

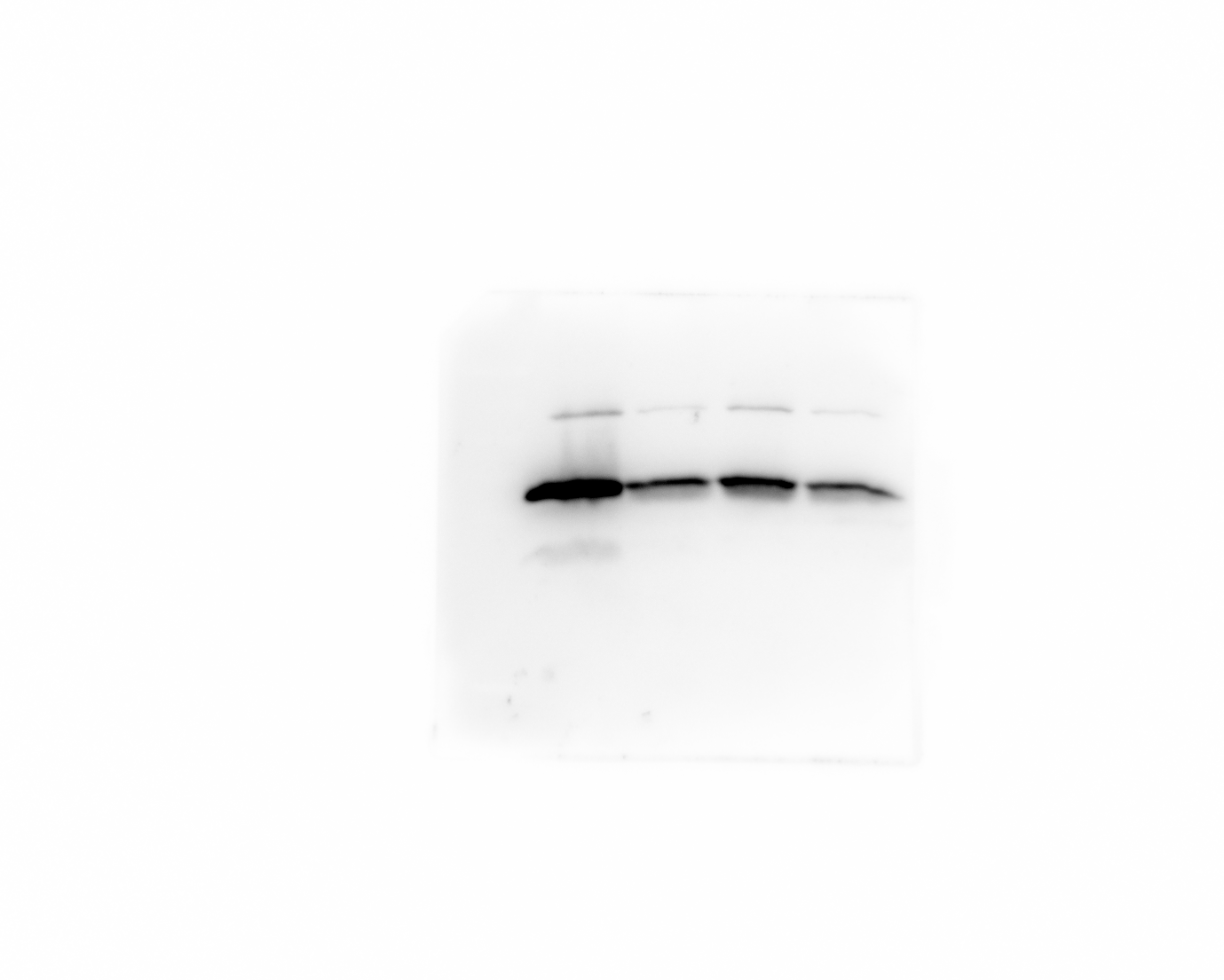

Supplement: Supplementary file 3 [file DataSheet1.zip › Supplementary_Raw_Blot/Supplementary_Raw_Blot_Fig9H_DRAM1---2---(white background).tif]

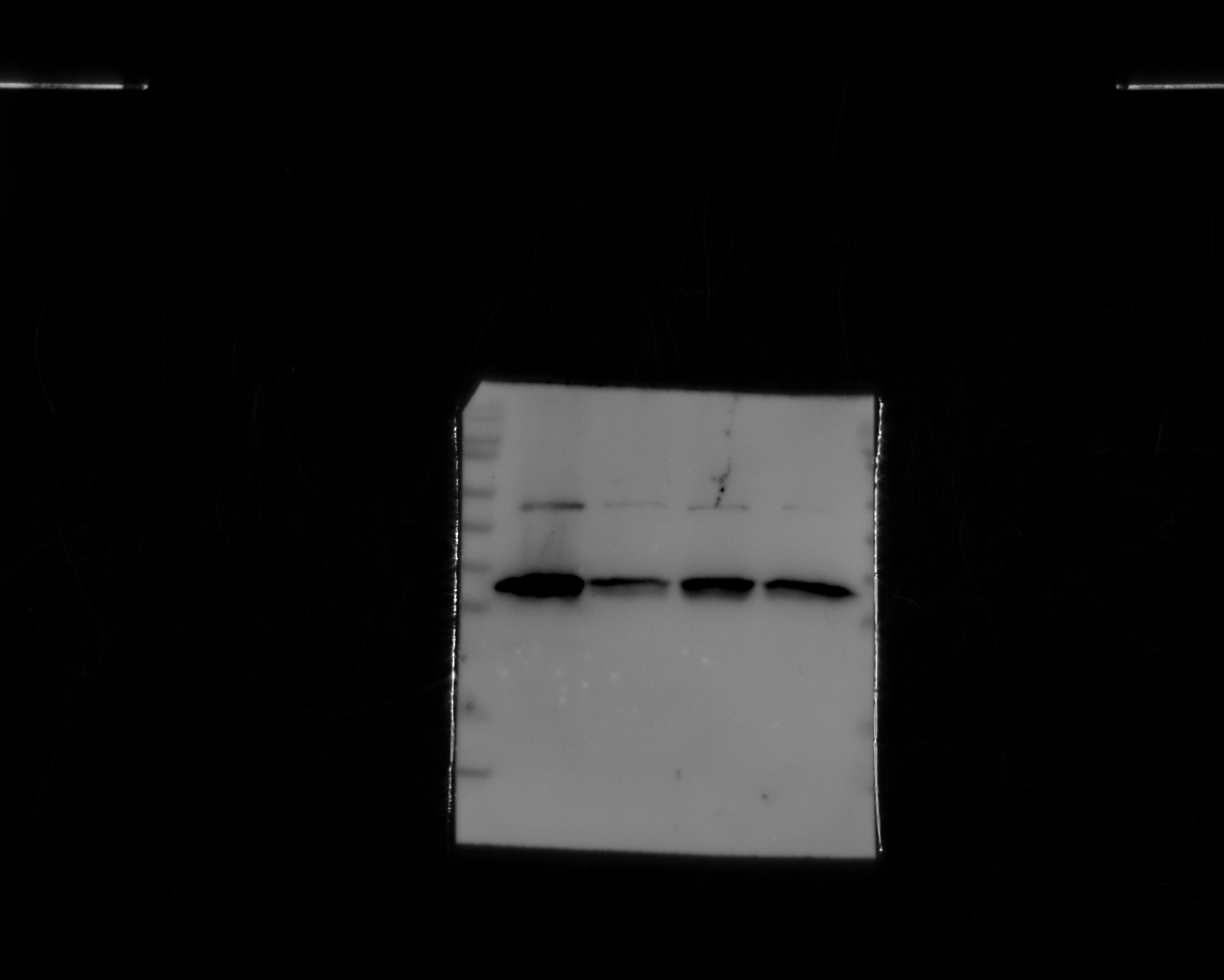

Supplement: Supplementary file 3 [file DataSheet1.zip › Supplementary_Raw_Blot/Supplementary_Raw_Blot_Fig9H_DRAM1---3---(black background).tif]

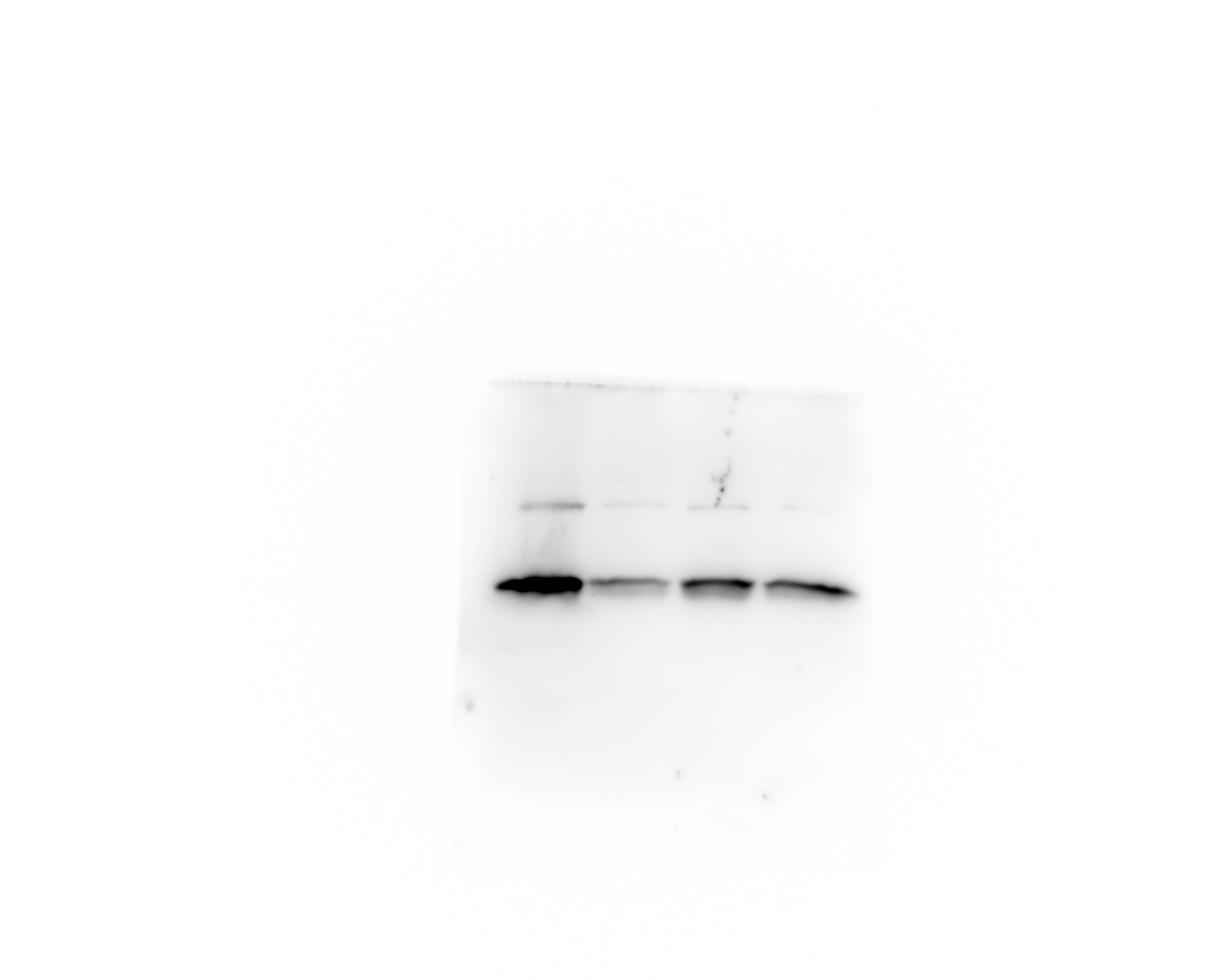

Supplement: Supplementary file 3 [file DataSheet1.zip › Supplementary_Raw_Blot/Supplementary_Raw_Blot_Fig9H_DRAM1---3---(white background).tif]

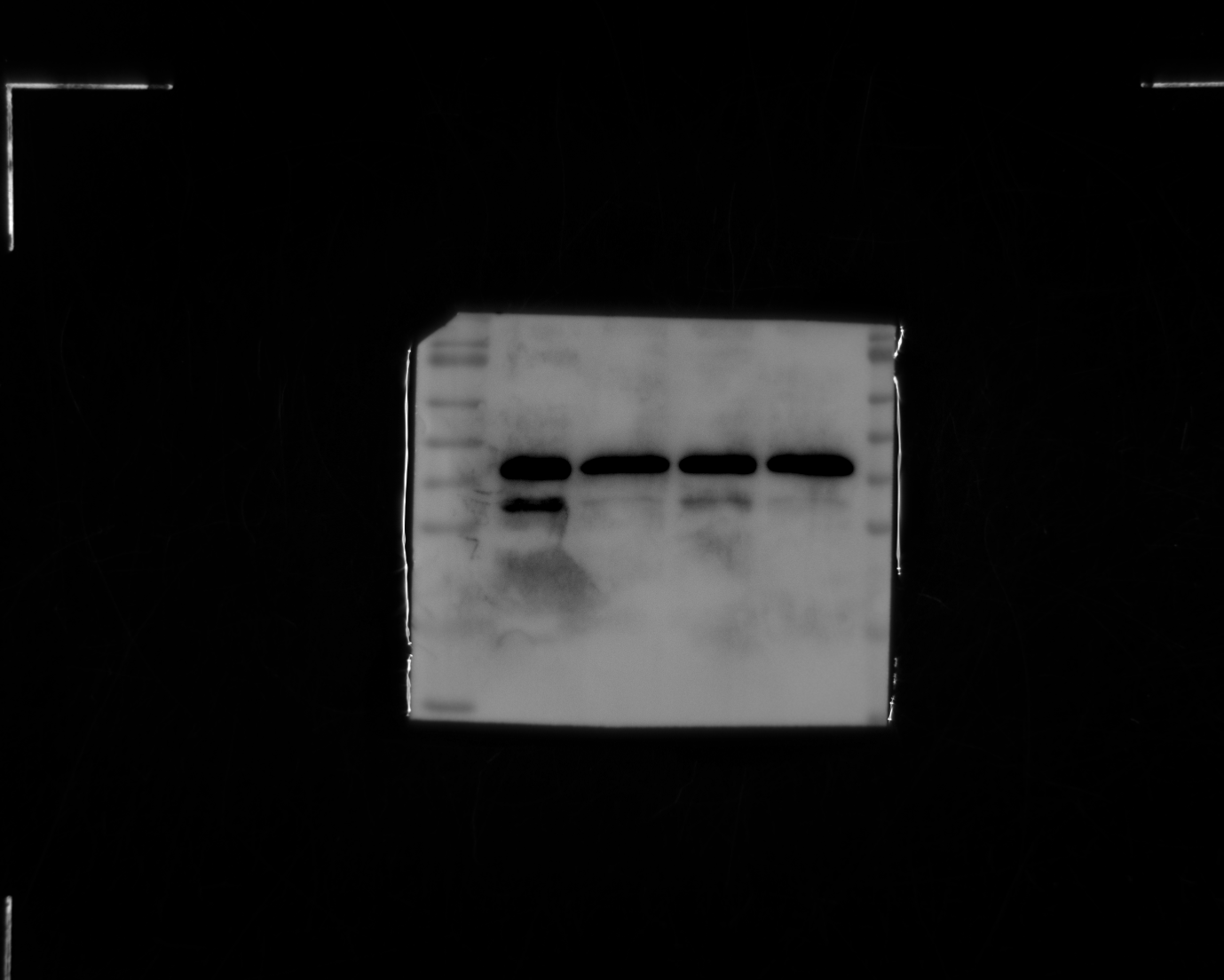

Supplement: Supplementary file 3 [file DataSheet1.zip › Supplementary_Raw_Blot/Supplementary_Raw_Blot_Fig9H_GAPDH---1---(black background).tif]

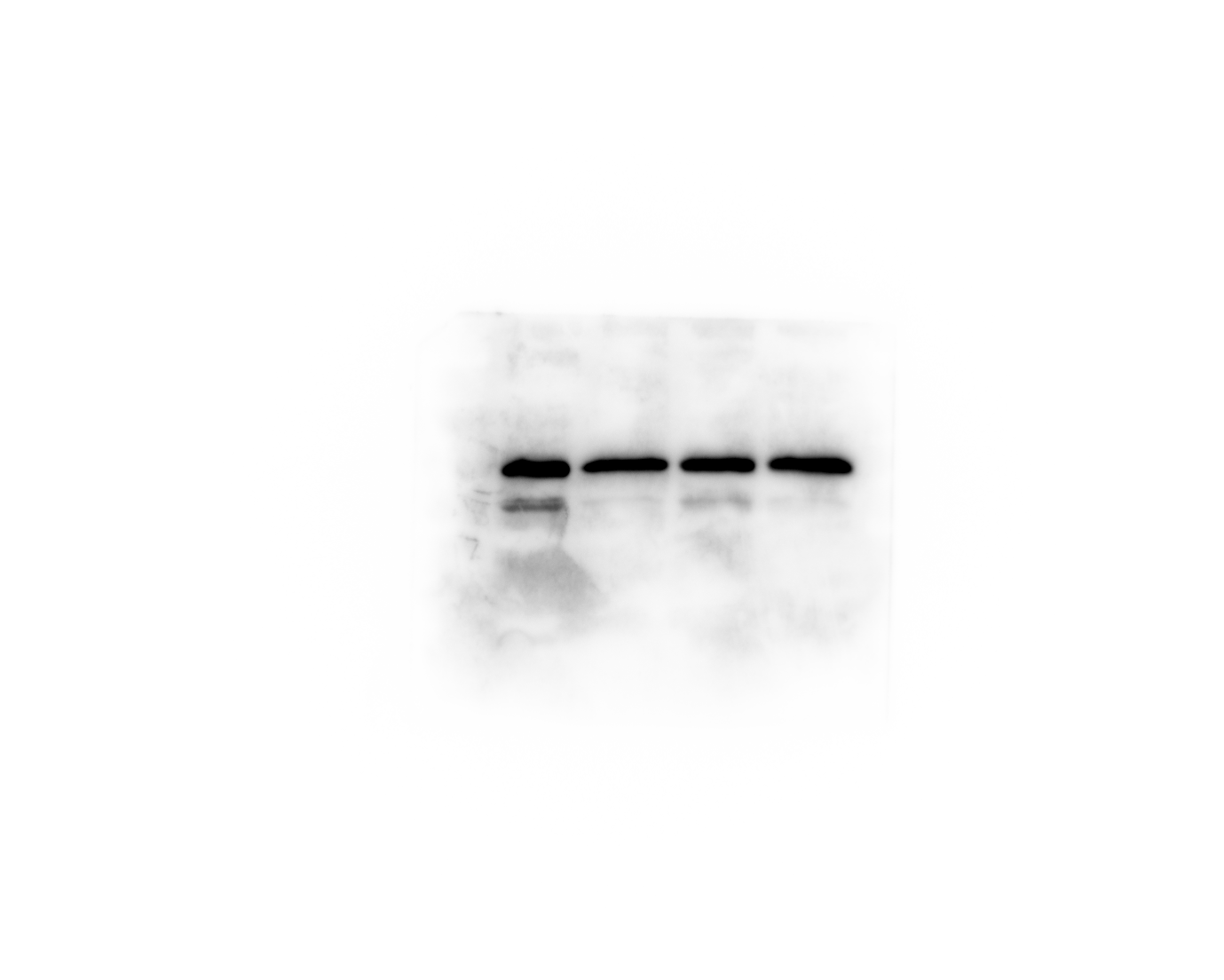

Supplement: Supplementary file 3 [file DataSheet1.zip › Supplementary_Raw_Blot/Supplementary_Raw_Blot_Fig9H_GAPDH---1---(white background).tif]

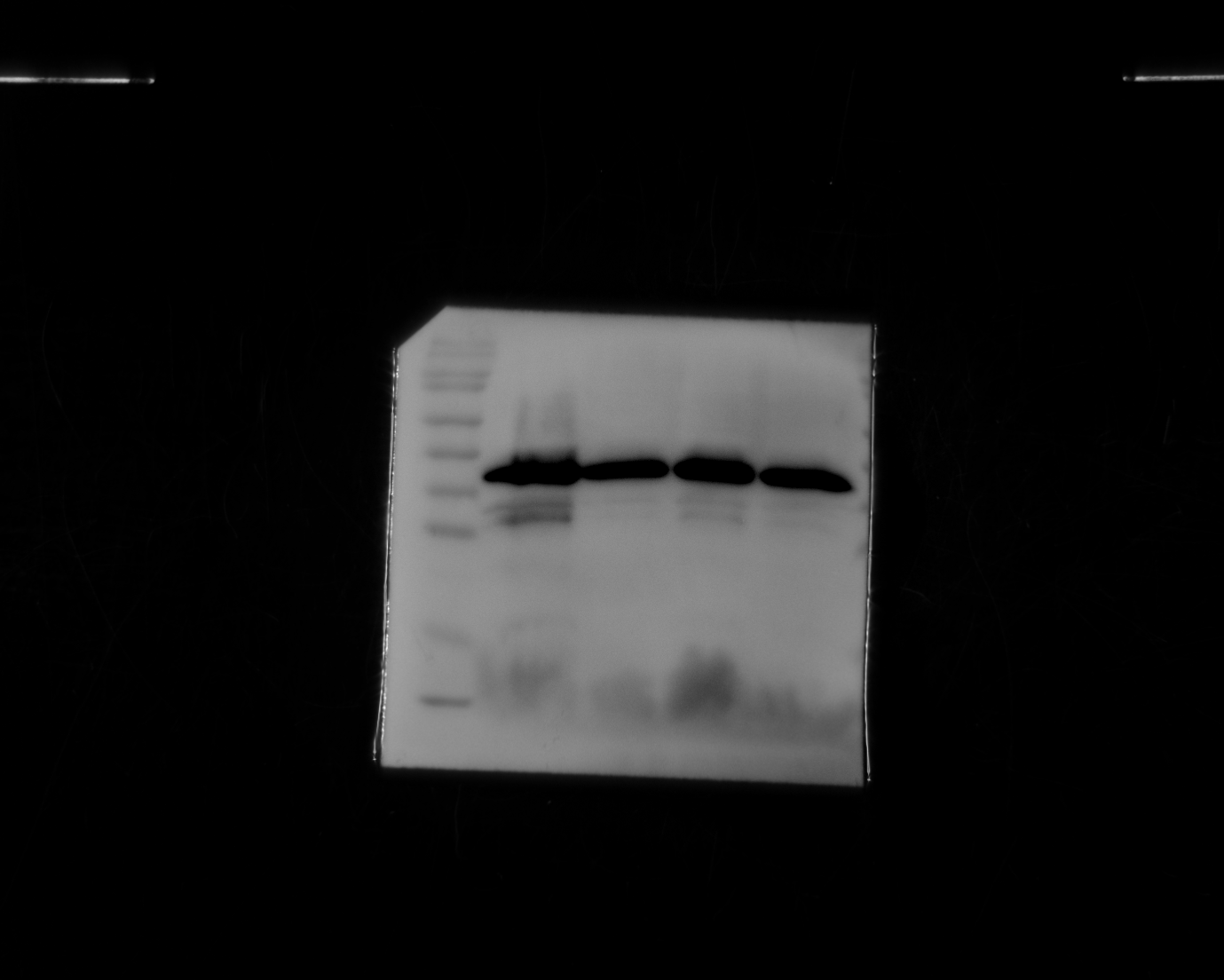

Supplement: Supplementary file 3 [file DataSheet1.zip › Supplementary_Raw_Blot/Supplementary_Raw_Blot_Fig9H_GAPDH---2---(black background).tif]

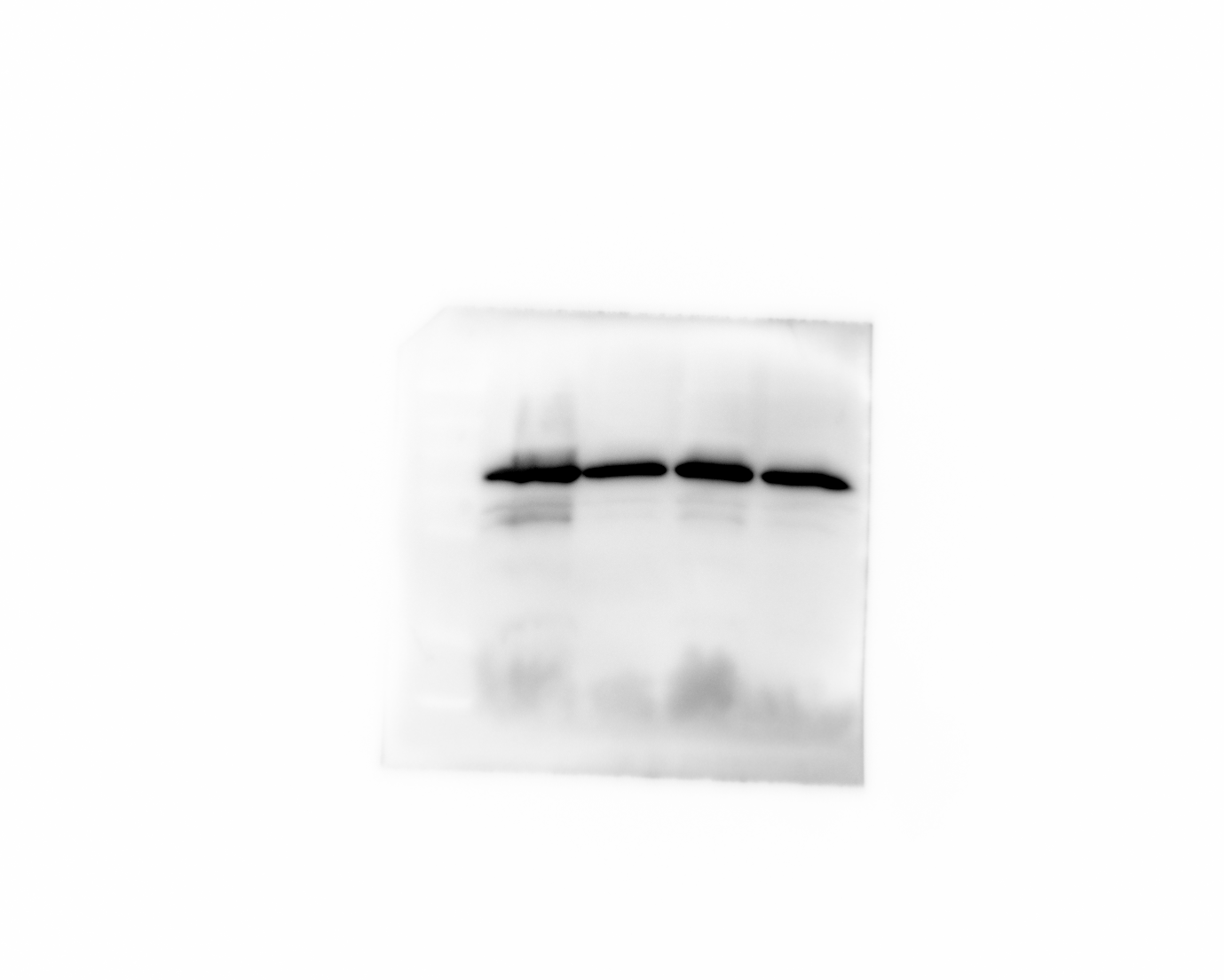

Supplement: Supplementary file 3 [file DataSheet1.zip › Supplementary_Raw_Blot/Supplementary_Raw_Blot_Fig9H_GAPDH---2---(white background).tif]

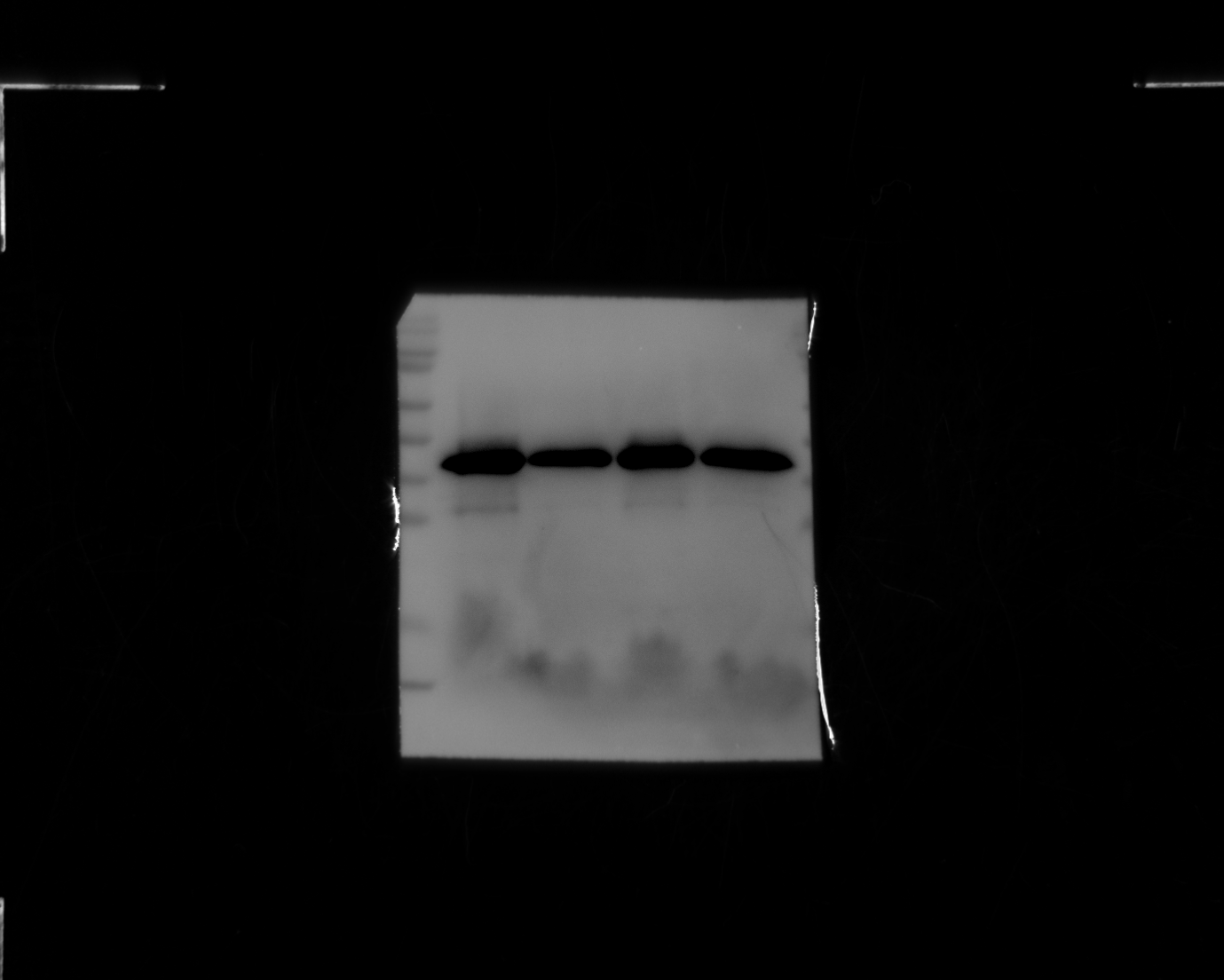

Supplement: Supplementary file 3 [file DataSheet1.zip › Supplementary_Raw_Blot/Supplementary_Raw_Blot_Fig9H_GAPDH---3---(black background).tif]

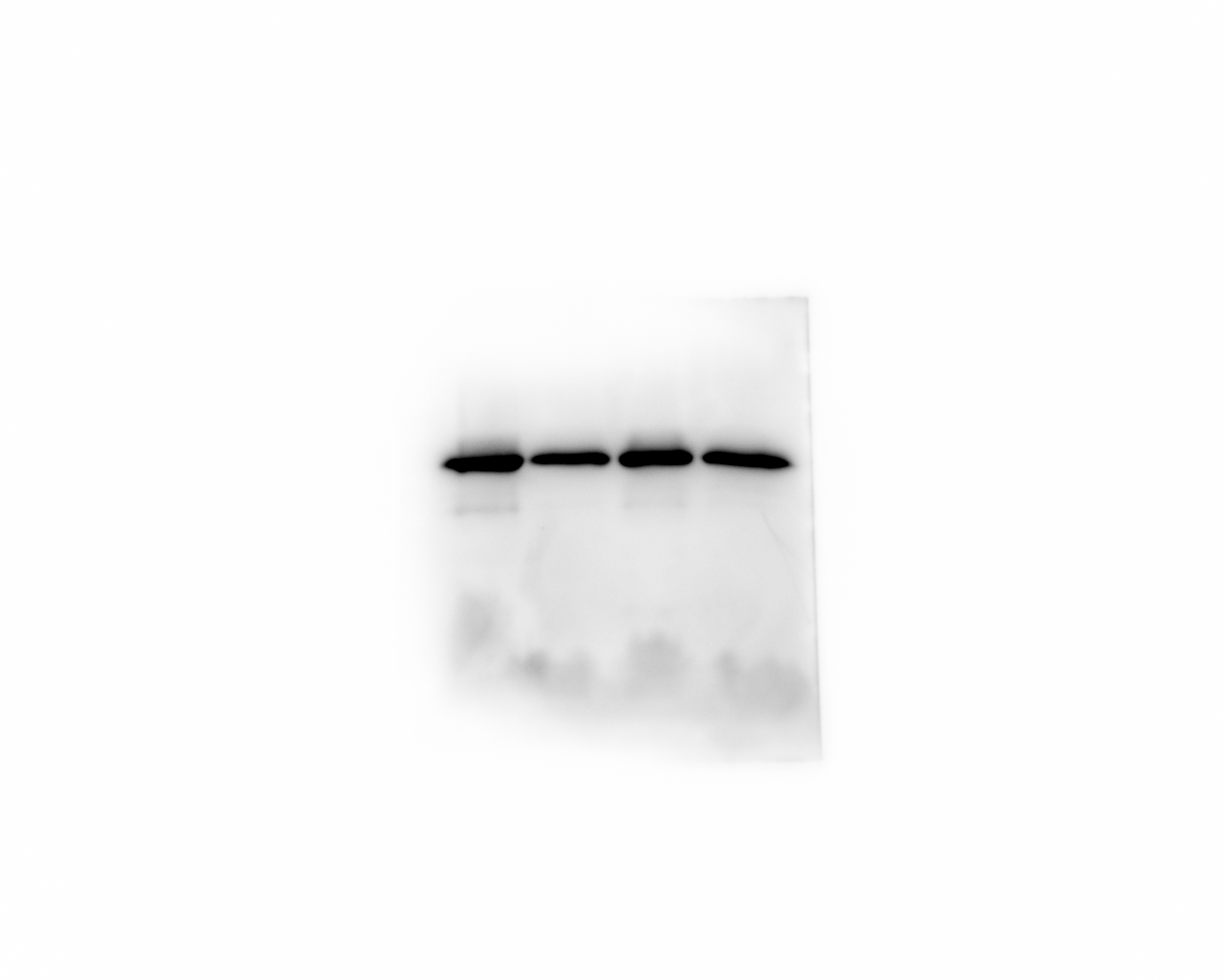

Supplement: Supplementary file 3 [file DataSheet1.zip › Supplementary_Raw_Blot/Supplementary_Raw_Blot_Fig9H_GAPDH---3---(white background).tif]
