# Supplementary material for: Integrative single-cell and bulk transcriptomic analyses identify DRAM1 as a candidate gene from fibroblast-associated transcriptional programs in colorectal cancer
Source: Front Oncol. 2026 Jun 11;16:1862796. doi: 10.3389/fonc.2026.1862796 (PMC13293847; doi:10.3389/fonc.2026.1862796)
Supplement: Supplementary file 4 [file DataSheet2.docx]

**Supplementary Figures**


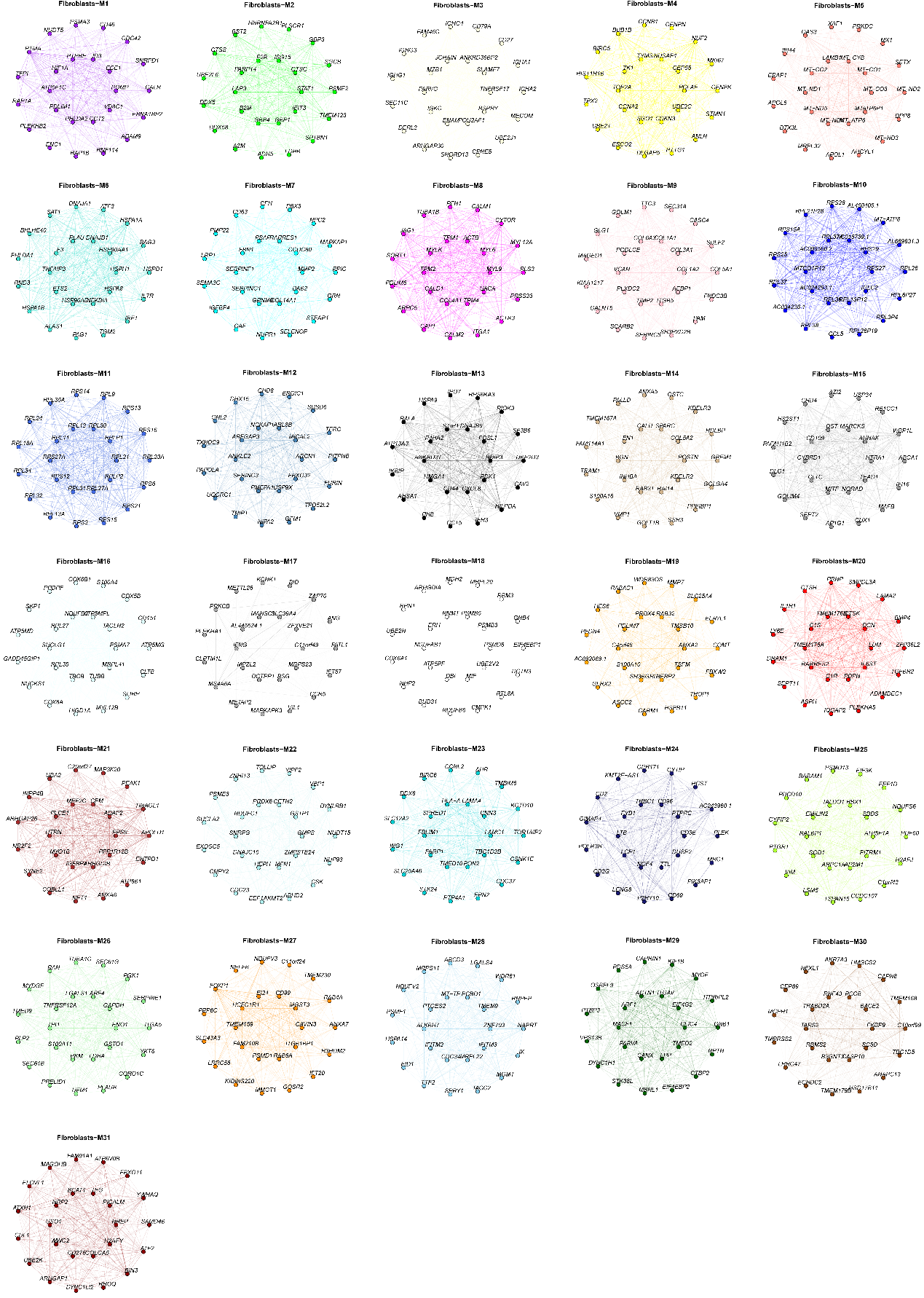


Supplementary Fig.1. Gene network for the top 25 genes in each fibroblast-associated module.


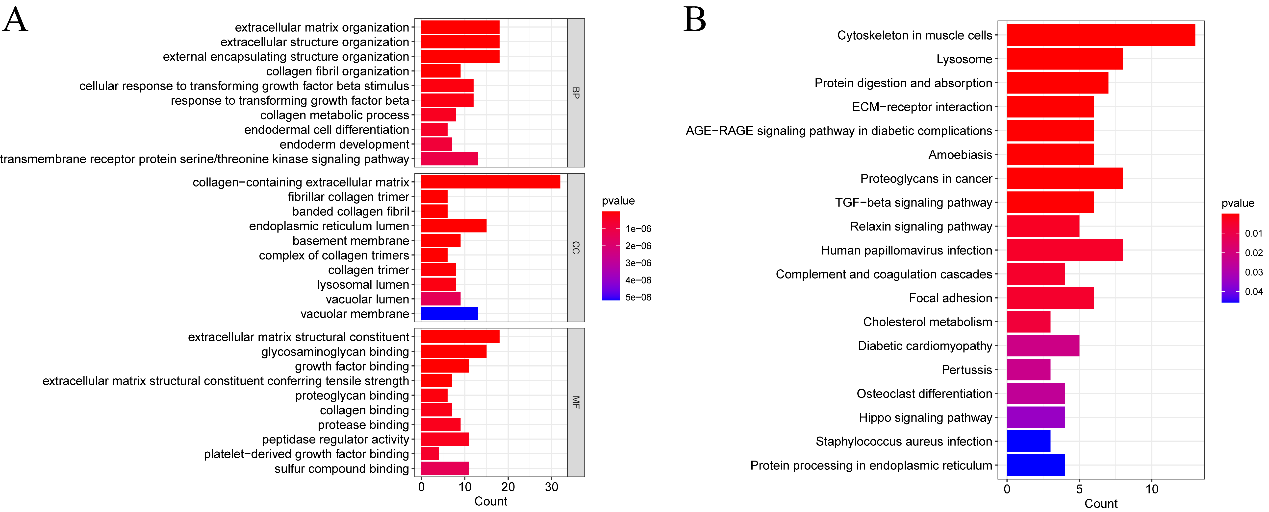


Supplementary Fig.2. (A) GO analysis, including biological processes (BP), cellular components (CC), and molecular functions (MF). (B) KEGG pathway analysis.


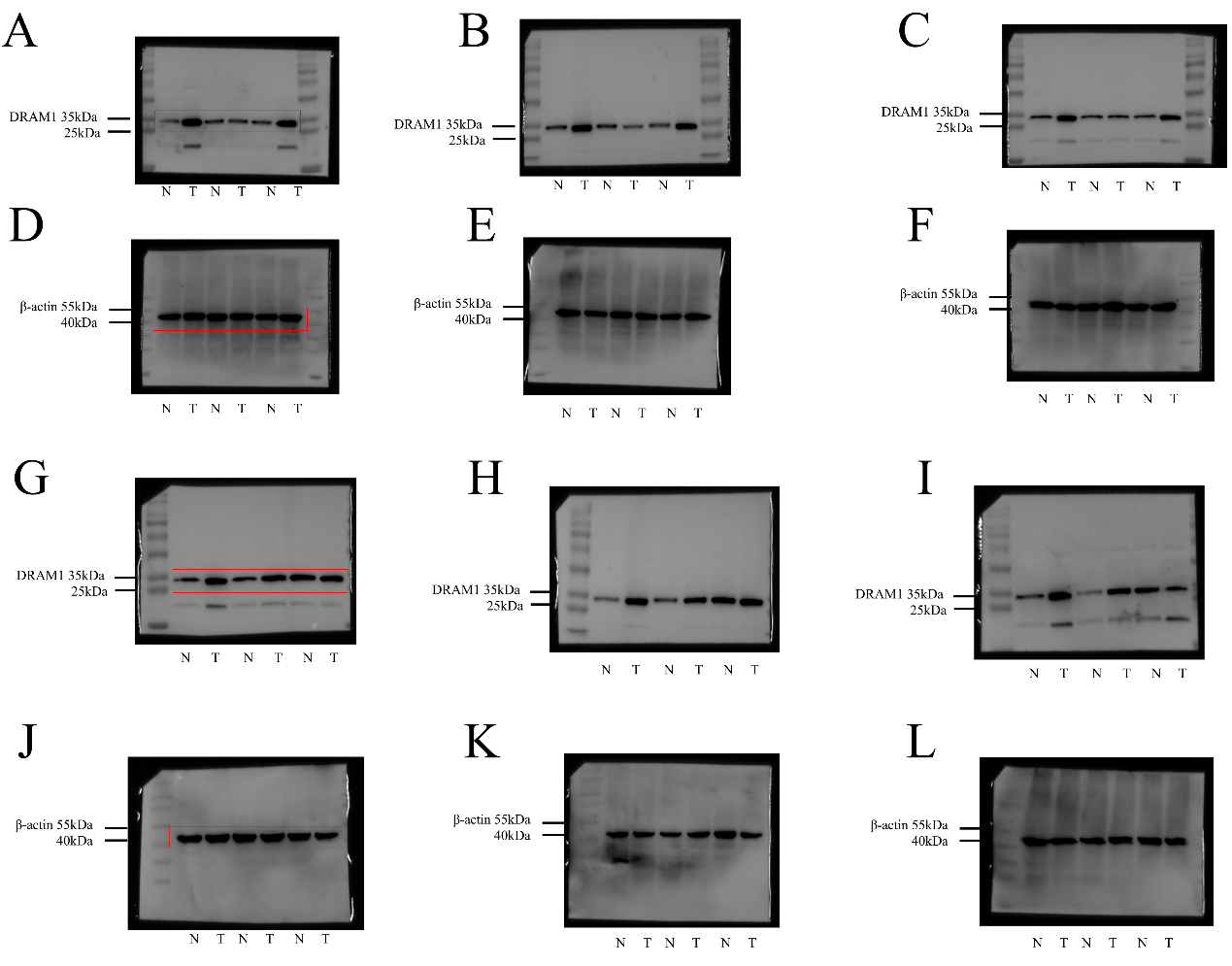


Supplementary Fig. 3. Raw data of the Western blots presented in Fig. 9A–B.

(A–F) Raw Western blot data of DRAM1 and β‑actin expression for patients 1–3, corresponding to Fig. 9A. (G–L) Raw Western blot data of DRAM1 and β‑actin expression for patients 4–6, corresponding to Fig. 9B. The bands highlighted by the red rectangles are those used in Fig. 9.


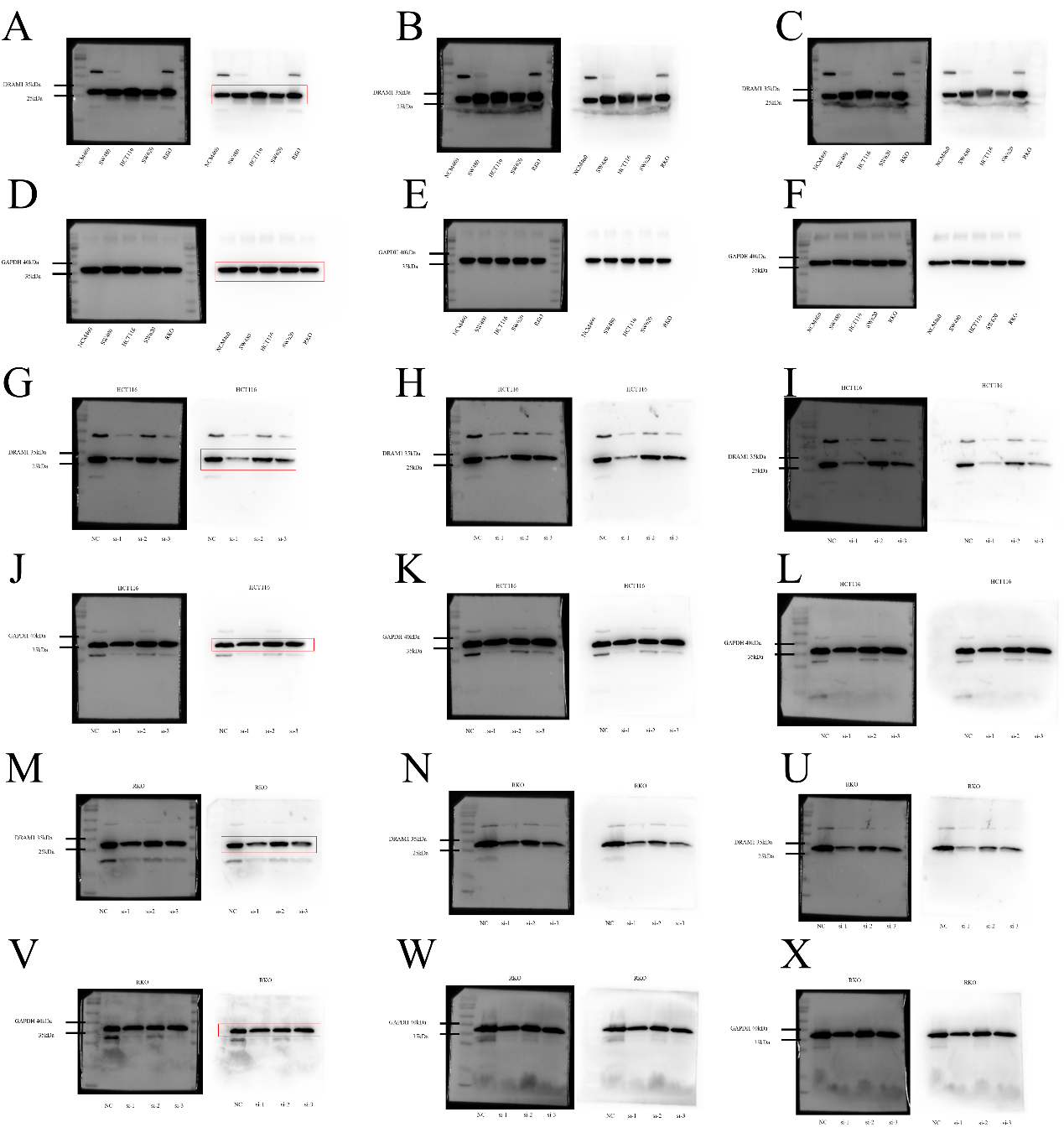


Supplementary Fig.4. Raw data of the Western blots shown in Fig. 9D, 9F, and 9H.

(A-F) Raw data of the Western blot bands showing DRAM1 and GAPDH expression in normal colon epithelial cell line NCM460 and colorectal cancer cell lines in Fig. 9D. (G-L) Raw data of the Western blot results validating DRAM1 knockout efficiency using three siRNAs in HCT116 cells in Fig. 9F. (M-X) Raw data of the Western blot results validating DRAM1 knockout efficiency using three siRNAs in RKO cells in Fig. 9H. The bands highlighted by the red rectangles were used in Fig. 9.
