# Supplementary material for: Integrative single-cell and bulk transcriptomic analyses identify DRAM1 as a candidate gene from fibroblast-associated transcriptional programs in colorectal cancer
Source: Front Oncol. 2026 Jun 11;16:1862796. doi: 10.3389/fonc.2026.1862796 (PMC13293847; doi:10.3389/fonc.2026.1862796)
Supplement: Supplementary file 6 [file Table1.docx]

Supplementary Table 1: siRNA sequences used in this study

| **Name** | **Sense sequence 5′ to 3′** | **Manufacturer** |
| --- | --- | --- |
| si-DRAM1-1 | AGCCACGAUGUAUACAAGATT | Sangon Biotech, Shanghai, China |
| si-DRAM1-2 | CCACAGAAAUCAAUGGUGATT | Sangon Biotech, Shanghai, China |
| si-DRAM1-3 | CCACGAUGUAUACAAGAUATT | Sangon Biotech, Shanghai, China |
| si-NC | UUCUCUCCGAACGUGUCACGUTT | Sangon Biotech, Shanghai, China |

Supplementary Table 2: Primer sequences used for qRT-PCR

| **Gene** | **Primer name** | **Sequence 5′ to 3′** | **Manufacturer** |
| --- | --- | --- | --- |
| DRAM1 | DRAM1-F | AGTGCTTGGATTGGTGGGATG | Sangon Biotech, Shanghai, China |
| DRAM1 | DRAM1-R | GATGGACTGTAGGAGCGTGTA | Sangon Biotech, Shanghai, China |
| ACTB | ACTB-F | CTGGAACGGTGAAGGTGACA | Sangon Biotech, Shanghai, China |
| ACTB | ACTB-R | AAGGGACTTCCTGTAACAACGCA | Sangon Biotech, Shanghai, China |

Supplementary Table 3: Antibodies used in this study

| **Antibody** | **Host** | **Manufacturer** | **Catalog No.** | **Dilution** |
| --- | --- | --- | --- | --- |
| DRAM1 Monoclonal Antibody | Mouse | BYabscience | BYmab-07344 | 1:1000 |
| ACTB Monoclonal Antibody | Mouse | BYabscience | BYmab-06768 | 1:2000 |
| GAPDH Monoclonal Antibody | Mouse | Proteintech Group, Wuhan, China | 60004-1-Ig | 1:50000 |
| HRP-conjugated Goat Anti-Mouse IgG (H+L) | Goat | Proteintech Group, Wuhan, China | SA00001-1 | 1:10000 |

Supplementary Table 4: Gene relationships corresponding to different colorectal cancer-associated fibroblast modules.

| gene_name | module | color |
| --- | --- | --- |
| HNRNPK | Fibroblasts-M1 | purple |
| B2M | Fibroblasts-M2 | green |
| ARHGAP30 | Fibroblasts-M3 | lightyellow |
| TPM3 | Fibroblasts-M4 | yellow |
| MT-CO2 | Fibroblasts-M5 | salmon |
| IGKC | Fibroblasts-M3 | lightyellow |
| NFKBIA | Fibroblasts-M6 | turquoise |
| DCAF5 | Fibroblasts-M2 | green |
| JCHAIN | Fibroblasts-M3 | lightyellow |
| ELF3 | Fibroblasts-M7 | cyan |
| HIF1A | Fibroblasts-M1 | purple |
| UBC | Fibroblasts-M6 | turquoise |
| PFN1 | Fibroblasts-M8 | magenta |
| BRWD1 | Fibroblasts-M9 | pink |
| RPS27 | Fibroblasts-M10 | blue |
| MT-RNR2 | Fibroblasts-M6 | turquoise |
| RPS3 | Fibroblasts-M11 | royalblue |
| KTN1 | Fibroblasts-M12 | steelblue |
| IGHA1 | Fibroblasts-M3 | lightyellow |
| MDM4 | Fibroblasts-M13 | black |
| RPS28 | Fibroblasts-M10 | blue |
| JUN | Fibroblasts-M6 | turquoise |
| IGHA2 | Fibroblasts-M3 | lightyellow |
| H3F3B | Fibroblasts-M6 | turquoise |
| RGS1 | Fibroblasts-M6 | turquoise |
| BTG1 | Fibroblasts-M13 | black |
| IGHG4 | Fibroblasts-M3 | lightyellow |
| RPS28P7 | Fibroblasts-M10 | blue |
| PRRC2C | Fibroblasts-M9 | pink |
| TAX1BP1 | Fibroblasts-M2 | green |
| MT-ND4 | Fibroblasts-M5 | salmon |
| CD79A | Fibroblasts-M3 | lightyellow |
| TMSB4X | Fibroblasts-M6 | turquoise |
| TYROBP | Fibroblasts-M14 | tan |
| MT-CO3 | Fibroblasts-M5 | salmon |
| MT-CO1 | Fibroblasts-M5 | salmon |
| MT-ND1 | Fibroblasts-M5 | salmon |
| AP1G1 | Fibroblasts-M15 | grey60 |
| COX5B | Fibroblasts-M16 | lightcyan |
| BIK | Fibroblasts-M17 | darkgrey |
| NKG7 | Fibroblasts-M10 | blue |
| MT-ATP6 | Fibroblasts-M5 | salmon |
| NDUFA5 | Fibroblasts-M18 | white |
| RPLP0 | Fibroblasts-M11 | royalblue |
| BTG3 | Fibroblasts-M19 | orange |
| CCNL1 | Fibroblasts-M6 | turquoise |
| RPL5 | Fibroblasts-M11 | royalblue |
| PTMA | Fibroblasts-M1 | purple |
| MYL12B | Fibroblasts-M16 | lightcyan |
| MT-ND2 | Fibroblasts-M5 | salmon |
| NSD2 | Fibroblasts-M2 | green |
| RPL15 | Fibroblasts-M11 | royalblue |
| PSAP | Fibroblasts-M7 | cyan |
| LRP10 | Fibroblasts-M6 | turquoise |
| MT-CYB | Fibroblasts-M5 | salmon |
| RPL27A | Fibroblasts-M11 | royalblue |
| HMGB2 | Fibroblasts-M4 | yellow |
| MT-ATP8 | Fibroblasts-M10 | blue |
| CCL5 | Fibroblasts-M10 | blue |
| GIT2 | Fibroblasts-M9 | pink |
| CKS2 | Fibroblasts-M6 | turquoise |
| UBB | Fibroblasts-M6 | turquoise |
| ANAPC15 | Fibroblasts-M2 | green |
| MT-ND5 | Fibroblasts-M5 | salmon |
| RWDD1 | Fibroblasts-M16 | lightcyan |
| LEPROT | Fibroblasts-M20 | red |
| HIST1H1E | Fibroblasts-M4 | yellow |
| ARHGEF12 | Fibroblasts-M21 | brown |
| RPS19 | Fibroblasts-M11 | royalblue |
| PSMB3 | Fibroblasts-M18 | white |
| IRF1 | Fibroblasts-M6 | turquoise |
| TAOK1 | Fibroblasts-M9 | pink |
| ACAA2 | Fibroblasts-M7 | cyan |
| RPS12 | Fibroblasts-M11 | royalblue |
| BAIAP2L1 | Fibroblasts-M22 | paleturquoise |
| HP1BP3 | Fibroblasts-M7 | cyan |
| RARRES3 | Fibroblasts-M2 | green |
| RPL3 | Fibroblasts-M11 | royalblue |
| PABPC1 | Fibroblasts-M10 | blue |
| MT-RNR1 | Fibroblasts-M6 | turquoise |
| FOS | Fibroblasts-M6 | turquoise |
| HLA-B | Fibroblasts-M23 | darkturquoise |
| RPS6 | Fibroblasts-M11 | royalblue |
| RGS10 | Fibroblasts-M6 | turquoise |
| DUSP22 | Fibroblasts-M7 | cyan |
| SUCLG2 | Fibroblasts-M23 | darkturquoise |
| RPL11 | Fibroblasts-M11 | royalblue |
| ARHGAP15 | Fibroblasts-M21 | brown |
| RPL10 | Fibroblasts-M11 | royalblue |
| RPL34 | Fibroblasts-M11 | royalblue |
| DEK | Fibroblasts-M4 | yellow |
| HSPA8 | Fibroblasts-M6 | turquoise |
| IGLC2 | Fibroblasts-M10 | blue |
| RPL37 | Fibroblasts-M10 | blue |
| GPR171 | Fibroblasts-M24 | midnightblue |
| GLRX | Fibroblasts-M13 | black |
| ZNF143 | Fibroblasts-M6 | turquoise |
| TMSB10 | Fibroblasts-M19 | orange |
| MTCO1P12 | Fibroblasts-M10 | blue |
| SP140L | Fibroblasts-M25 | greenyellow |
| PSMD13 | Fibroblasts-M25 | greenyellow |
| SEC61B | Fibroblasts-M26 | lightgreen |
| ZFP36L2 | Fibroblasts-M20 | red |
| RCSD1 | Fibroblasts-M21 | brown |
| H2AFZ | Fibroblasts-M4 | yellow |
| COMTD1 | Fibroblasts-M27 | darkorange |
| IFITM2 | Fibroblasts-M28 | skyblue |
| 6-Sep | Fibroblasts-M22 | paleturquoise |
| IER2 | Fibroblasts-M10 | blue |
| CD69 | Fibroblasts-M24 | midnightblue |
| RPL36AL | Fibroblasts-M26 | lightgreen |
| SAT1 | Fibroblasts-M6 | turquoise |
| CPVL | Fibroblasts-M7 | cyan |
| RPL13P12 | Fibroblasts-M10 | blue |
| IL13RA1 | Fibroblasts-M5 | salmon |
| SERF2 | Fibroblasts-M16 | lightcyan |
| HIST1H4C | Fibroblasts-M4 | yellow |
| CITED2 | Fibroblasts-M6 | turquoise |
| MYL6 | Fibroblasts-M8 | magenta |
| FAU | Fibroblasts-M11 | royalblue |
| GADD45A | Fibroblasts-M1 | purple |
| AC005912.1 | Fibroblasts-M11 | royalblue |
| CCL4 | Fibroblasts-M21 | brown |
| MRPS5 | Fibroblasts-M21 | brown |
| EEF1B2 | Fibroblasts-M11 | royalblue |
| JUNB | Fibroblasts-M6 | turquoise |
| AL445524.1 | Fibroblasts-M17 | darkgrey |
| RPLP1 | Fibroblasts-M11 | royalblue |
| HOPX | Fibroblasts-M29 | darkgreen |
| ATP5PF | Fibroblasts-M18 | white |
| AC116533.1 | Fibroblasts-M10 | blue |
| GAPDH | Fibroblasts-M26 | lightgreen |
| MAPK1 | Fibroblasts-M2 | green |
| SPATS2L | Fibroblasts-M9 | pink |
| CD3E | Fibroblasts-M24 | midnightblue |
| FAM111A | Fibroblasts-M5 | salmon |
| RPL23 | Fibroblasts-M11 | royalblue |
| HSP90AB1 | Fibroblasts-M6 | turquoise |
| RPL35 | Fibroblasts-M16 | lightcyan |
| ANXA2 | Fibroblasts-M19 | orange |
| RPS2 | Fibroblasts-M11 | royalblue |
| MAP1LC3B | Fibroblasts-M6 | turquoise |
| TBC1D10C | Fibroblasts-M24 | midnightblue |
| PEBP1 | Fibroblasts-M7 | cyan |
| IER3 | Fibroblasts-M13 | black |
| EPB41L1 | Fibroblasts-M21 | brown |
| GCC2 | Fibroblasts-M9 | pink |
| VAMP5 | Fibroblasts-M4 | yellow |
| FUS | Fibroblasts-M6 | turquoise |
| RPL9 | Fibroblasts-M11 | royalblue |
| NEAT1 | Fibroblasts-M6 | turquoise |
| RPL14 | Fibroblasts-M11 | royalblue |
| RSU1 | Fibroblasts-M8 | magenta |
| PHF20L1 | Fibroblasts-M20 | red |
| YWHAB | Fibroblasts-M8 | magenta |
| BTG2 | Fibroblasts-M6 | turquoise |
| RPL30 | Fibroblasts-M11 | royalblue |
| CD52 | Fibroblasts-M28 | skyblue |
| RPS27A | Fibroblasts-M11 | royalblue |
| CISD2 | Fibroblasts-M13 | black |
| POLR2G | Fibroblasts-M21 | brown |
| ATP5MC3 | Fibroblasts-M8 | magenta |
| RPL24 | Fibroblasts-M11 | royalblue |
| HCFC1R1 | Fibroblasts-M27 | darkorange |
| RPL13A | Fibroblasts-M11 | royalblue |
| AP3B1 | Fibroblasts-M5 | salmon |
| HLTF | Fibroblasts-M30 | saddlebrown |
| NMI | Fibroblasts-M14 | tan |
| MINOS1 | Fibroblasts-M10 | blue |
| AL590867.2 | Fibroblasts-M10 | blue |
| YWHAZ | Fibroblasts-M8 | magenta |
| CMTR2 | Fibroblasts-M4 | yellow |
| HLA-DPA1 | Fibroblasts-M10 | blue |
| FABP1 | Fibroblasts-M13 | black |
| WDR83OS | Fibroblasts-M19 | orange |
| DLGAP4 | Fibroblasts-M19 | orange |
| RPS7P10 | Fibroblasts-M11 | royalblue |
| H1F0 | Fibroblasts-M13 | black |
| CYSTM1 | Fibroblasts-M21 | brown |
| TFF3 | Fibroblasts-M11 | royalblue |
| RPL32 | Fibroblasts-M11 | royalblue |
| NDUFB1 | Fibroblasts-M11 | royalblue |
| SF3A3 | Fibroblasts-M2 | green |
| NUCKS1 | Fibroblasts-M16 | lightcyan |
| RPL6 | Fibroblasts-M11 | royalblue |
| HSP90AA1 | Fibroblasts-M6 | turquoise |
| RPS5 | Fibroblasts-M11 | royalblue |
| ZNF331 | Fibroblasts-M24 | midnightblue |
| BTF3 | Fibroblasts-M11 | royalblue |
| KLF6 | Fibroblasts-M6 | turquoise |
| SESN3 | Fibroblasts-M7 | cyan |
| SLBP | Fibroblasts-M4 | yellow |
| RPL31 | Fibroblasts-M11 | royalblue |
| SRRM1 | Fibroblasts-M20 | red |
| CD3D | Fibroblasts-M10 | blue |
| XPA | Fibroblasts-M16 | lightcyan |
| SH3GLB1 | Fibroblasts-M8 | magenta |
| ACTB | Fibroblasts-M8 | magenta |
| SERP1 | Fibroblasts-M26 | lightgreen |
| H1FX | Fibroblasts-M24 | midnightblue |
| KRT20 | Fibroblasts-M10 | blue |
| SLF2 | Fibroblasts-M5 | salmon |
| LPAR6 | Fibroblasts-M5 | salmon |
| RPS18 | Fibroblasts-M11 | royalblue |
| CARS | Fibroblasts-M25 | greenyellow |
| PINK1 | Fibroblasts-M7 | cyan |
| LGALS4 | Fibroblasts-M28 | skyblue |
| ZNF655 | Fibroblasts-M5 | salmon |
| MTATP6P1 | Fibroblasts-M5 | salmon |
| COQ4 | Fibroblasts-M25 | greenyellow |
| ACIN1 | Fibroblasts-M9 | pink |
| CALM2 | Fibroblasts-M8 | magenta |
| POLR1D | Fibroblasts-M27 | darkorange |
| SUCLG1 | Fibroblasts-M16 | lightcyan |
| IGHG2 | Fibroblasts-M3 | lightyellow |
| RPL35A | Fibroblasts-M11 | royalblue |
| CALM1 | Fibroblasts-M8 | magenta |
| ARL1 | Fibroblasts-M14 | tan |
| DUSP1 | Fibroblasts-M6 | turquoise |
| RPE | Fibroblasts-M5 | salmon |
| SELENOF | Fibroblasts-M20 | red |
| ASAH1 | Fibroblasts-M21 | brown |
| RPL13 | Fibroblasts-M11 | royalblue |
| TTC37 | Fibroblasts-M9 | pink |
| CHTF8 | Fibroblasts-M28 | skyblue |
| RPS14 | Fibroblasts-M11 | royalblue |
| ANAPC16 | Fibroblasts-M10 | blue |
| MMD | Fibroblasts-M20 | red |
| ATP5PO | Fibroblasts-M21 | brown |
| ZBTB7A | Fibroblasts-M29 | darkgreen |
| MT1E | Fibroblasts-M18 | white |
| RPL29 | Fibroblasts-M11 | royalblue |
| NDUFS1 | Fibroblasts-M9 | pink |
| CXCR4 | Fibroblasts-M24 | midnightblue |
| CHD4 | Fibroblasts-M15 | grey60 |
| RPL21 | Fibroblasts-M11 | royalblue |
| FCER1G | Fibroblasts-M24 | midnightblue |
| CWC25 | Fibroblasts-M12 | steelblue |
| GPR65 | Fibroblasts-M25 | greenyellow |
| COX7C | Fibroblasts-M11 | royalblue |
| RPS23 | Fibroblasts-M11 | royalblue |
| AL669831.3 | Fibroblasts-M10 | blue |
| CCT8 | Fibroblasts-M6 | turquoise |
| INSIG1 | Fibroblasts-M6 | turquoise |
| NACA | Fibroblasts-M13 | black |
| ADAP1 | Fibroblasts-M4 | yellow |
| TAOK3 | Fibroblasts-M5 | salmon |
| EIF1 | Fibroblasts-M13 | black |
| GIMAP7 | Fibroblasts-M10 | blue |
| RPS8 | Fibroblasts-M11 | royalblue |
| RNF213 | Fibroblasts-M2 | green |
| PSMD6 | Fibroblasts-M29 | darkgreen |
| RPS4Y1 | Fibroblasts-M10 | blue |
| KNL1 | Fibroblasts-M4 | yellow |
| EZH2 | Fibroblasts-M4 | yellow |
| PQBP1 | Fibroblasts-M25 | greenyellow |
| RPLP2 | Fibroblasts-M11 | royalblue |
| S100A10 | Fibroblasts-M19 | orange |
| RPS24 | Fibroblasts-M11 | royalblue |
| ACTR3 | Fibroblasts-M8 | magenta |
| PRKCB | Fibroblasts-M17 | darkgrey |
| RPL18A | Fibroblasts-M11 | royalblue |
| STAT1 | Fibroblasts-M2 | green |
| ELMO1 | Fibroblasts-M17 | darkgrey |
| GOLGB1 | Fibroblasts-M9 | pink |
| CYTIP | Fibroblasts-M24 | midnightblue |
| TAF7 | Fibroblasts-M6 | turquoise |
| RBM3 | Fibroblasts-M18 | white |
| LDHB | Fibroblasts-M2 | green |
| RUNX1 | Fibroblasts-M9 | pink |
| CSNK1G3 | Fibroblasts-M20 | red |
| RPS9 | Fibroblasts-M11 | royalblue |
| IFITM3 | Fibroblasts-M28 | skyblue |
| SUMO1 | Fibroblasts-M1 | purple |
| CD7 | Fibroblasts-M17 | darkgrey |
| AP3S1 | Fibroblasts-M15 | grey60 |
| PRDX5 | Fibroblasts-M10 | blue |
| LINC02446 | Fibroblasts-M21 | brown |
| AC109326.1 | Fibroblasts-M10 | blue |
| BCAP29 | Fibroblasts-M14 | tan |
| LINC01133 | Fibroblasts-M10 | blue |
| TRBC1 | Fibroblasts-M24 | midnightblue |
| RPL10A | Fibroblasts-M11 | royalblue |
| ADM | Fibroblasts-M6 | turquoise |
| COX7A2 | Fibroblasts-M8 | magenta |
| SMARCA4 | Fibroblasts-M1 | purple |
| HMGN1 | Fibroblasts-M10 | blue |
| FAHD2A | Fibroblasts-M4 | yellow |
| HBB | Fibroblasts-M10 | blue |
| CXCL3 | Fibroblasts-M6 | turquoise |
| UBXN4 | Fibroblasts-M9 | pink |
| CDC42 | Fibroblasts-M1 | purple |
| UTRN | Fibroblasts-M21 | brown |
| ARHGDIB | Fibroblasts-M21 | brown |
| PDE4D | Fibroblasts-M24 | midnightblue |
| ACAP1 | Fibroblasts-M10 | blue |
| UTP18 | Fibroblasts-M23 | darkturquoise |
| RPL24P2 | Fibroblasts-M10 | blue |
| RPL28 | Fibroblasts-M10 | blue |
| HLA-A | Fibroblasts-M23 | darkturquoise |
| MT-ND3 | Fibroblasts-M5 | salmon |
| MRPL9 | Fibroblasts-M2 | green |
| RPS15 | Fibroblasts-M11 | royalblue |
| SNRPG | Fibroblasts-M22 | paleturquoise |
| RPS13 | Fibroblasts-M11 | royalblue |
| TMEM260 | Fibroblasts-M2 | green |
| RPS3AP26 | Fibroblasts-M31 | darkred |
| PDCL3 | Fibroblasts-M18 | white |
| CFL1 | Fibroblasts-M8 | magenta |
| RPS27L | Fibroblasts-M18 | white |
| HNRNPC | Fibroblasts-M13 | black |
| IGHM | Fibroblasts-M10 | blue |
| ZFP36 | Fibroblasts-M6 | turquoise |
| ITSN2 | Fibroblasts-M25 | greenyellow |
| 7-Sep | Fibroblasts-M21 | brown |
| RPL7A | Fibroblasts-M11 | royalblue |
| SH3BGRL3 | Fibroblasts-M19 | orange |
| MT2A | Fibroblasts-M13 | black |
| CCL2 | Fibroblasts-M20 | red |
| S100A9 | Fibroblasts-M28 | skyblue |
| ARID4B | Fibroblasts-M20 | red |
| ATP5F1A | Fibroblasts-M25 | greenyellow |
| LGALS1 | Fibroblasts-M26 | lightgreen |
| RPS16 | Fibroblasts-M11 | royalblue |
| HBA2 | Fibroblasts-M10 | blue |
| GOLGA4 | Fibroblasts-M14 | tan |
| PPP6R2 | Fibroblasts-M6 | turquoise |
| POU2AF1 | Fibroblasts-M3 | lightyellow |
| PPP1R12B | Fibroblasts-M21 | brown |
| SMC5 | Fibroblasts-M25 | greenyellow |
| TAGLN2 | Fibroblasts-M16 | lightcyan |
| RPL7 | Fibroblasts-M11 | royalblue |
| CD99 | Fibroblasts-M27 | darkorange |
| GTF3C6 | Fibroblasts-M16 | lightcyan |
| PPP1R37 | Fibroblasts-M10 | blue |
| RPL39 | Fibroblasts-M10 | blue |
| TMEM87A | Fibroblasts-M14 | tan |
| LRRFIP1 | Fibroblasts-M13 | black |
| RSRP1 | Fibroblasts-M6 | turquoise |
| ZFAS1 | Fibroblasts-M11 | royalblue |
| COX6B1 | Fibroblasts-M16 | lightcyan |
| EFHD2 | Fibroblasts-M5 | salmon |
| RPL27 | Fibroblasts-M16 | lightcyan |
| FCMR | Fibroblasts-M6 | turquoise |
| MBD4 | Fibroblasts-M15 | grey60 |
| HSPA1B | Fibroblasts-M6 | turquoise |
| EXOSC1 | Fibroblasts-M9 | pink |
| EVL | Fibroblasts-M21 | brown |
| HNRNPA2B1 | Fibroblasts-M2 | green |
| TCF25 | Fibroblasts-M30 | saddlebrown |
| IFRD1 | Fibroblasts-M6 | turquoise |
| NFKBIZ | Fibroblasts-M6 | turquoise |
| PTPRC | Fibroblasts-M24 | midnightblue |
| RPS15A | Fibroblasts-M10 | blue |
| IGHG1 | Fibroblasts-M3 | lightyellow |
| RHOC | Fibroblasts-M8 | magenta |
| TSTD1 | Fibroblasts-M10 | blue |
| RPS29 | Fibroblasts-M10 | blue |
| ARHGAP5 | Fibroblasts-M13 | black |
| WSB1 | Fibroblasts-M6 | turquoise |
| SLAMF7 | Fibroblasts-M3 | lightyellow |
| KIAA2026 | Fibroblasts-M31 | darkred |
| AC099336.2 | Fibroblasts-M10 | blue |
| GOLGA5 | Fibroblasts-M31 | darkred |
| MYOF | Fibroblasts-M29 | darkgreen |
| DGKD | Fibroblasts-M6 | turquoise |
| RPL23A | Fibroblasts-M11 | royalblue |
| FAHD1 | Fibroblasts-M8 | magenta |
| SEC62 | Fibroblasts-M9 | pink |
| DNAJB1 | Fibroblasts-M6 | turquoise |
| HMGB1 | Fibroblasts-M4 | yellow |
| NABP1 | Fibroblasts-M7 | cyan |
| GIMAP4 | Fibroblasts-M24 | midnightblue |
| TOLLIP | Fibroblasts-M22 | paleturquoise |
| RREB1 | Fibroblasts-M21 | brown |
| ECE1 | Fibroblasts-M1 | purple |
| ANP32B | Fibroblasts-M4 | yellow |
| MLXIP | Fibroblasts-M9 | pink |
| PNRC1 | Fibroblasts-M10 | blue |
| HLA-DRA | Fibroblasts-M11 | royalblue |
| FAM208A | Fibroblasts-M9 | pink |
| ARPC2 | Fibroblasts-M8 | magenta |
| YRDC | Fibroblasts-M13 | black |
| NDUFB9 | Fibroblasts-M21 | brown |
| ISG20 | Fibroblasts-M6 | turquoise |
| IFNG | Fibroblasts-M17 | darkgrey |
| TCIM | Fibroblasts-M6 | turquoise |
| LRRC40 | Fibroblasts-M17 | darkgrey |
| AL118516.1 | Fibroblasts-M13 | black |
| CALR | Fibroblasts-M1 | purple |
| MIA3 | Fibroblasts-M9 | pink |
| GOLGA1 | Fibroblasts-M3 | lightyellow |
| PRKRIP1 | Fibroblasts-M9 | pink |
| PSMA7 | Fibroblasts-M16 | lightcyan |
| OSGEP | Fibroblasts-M12 | steelblue |
| NIT1 | Fibroblasts-M31 | darkred |
| TIMM50 | Fibroblasts-M1 | purple |
| ODF2L | Fibroblasts-M1 | purple |
| CD3G | Fibroblasts-M24 | midnightblue |
| SGK1 | Fibroblasts-M6 | turquoise |
| KAT6B | Fibroblasts-M7 | cyan |
| PTPN1 | Fibroblasts-M22 | paleturquoise |
| SUB1 | Fibroblasts-M6 | turquoise |
| S100A11 | Fibroblasts-M26 | lightgreen |
| FKBP3 | Fibroblasts-M8 | magenta |
| PMAIP1 | Fibroblasts-M6 | turquoise |
| PRSS23 | Fibroblasts-M8 | magenta |
| PDZK1IP1 | Fibroblasts-M25 | greenyellow |
| CENPF | Fibroblasts-M4 | yellow |
| COMMD8 | Fibroblasts-M8 | magenta |
| EIF4G2 | Fibroblasts-M29 | darkgreen |
| RTN4 | Fibroblasts-M14 | tan |
| RPS3A | Fibroblasts-M11 | royalblue |
| DOCK10 | Fibroblasts-M21 | brown |
| BBS2 | Fibroblasts-M10 | blue |
| DNAJC5 | Fibroblasts-M13 | black |
| FYB1 | Fibroblasts-M24 | midnightblue |
| RPS4X | Fibroblasts-M20 | red |
| CD8B | Fibroblasts-M10 | blue |
| GOLIM4 | Fibroblasts-M15 | grey60 |
| RC3H2 | Fibroblasts-M12 | steelblue |
| PAN3 | Fibroblasts-M8 | magenta |
| C1R | Fibroblasts-M20 | red |
| AP001324.1 | Fibroblasts-M10 | blue |
| GNAS | Fibroblasts-M21 | brown |
| MSRA | Fibroblasts-M27 | darkorange |
| DHRS7 | Fibroblasts-M6 | turquoise |
| CALU | Fibroblasts-M14 | tan |
| SRSF5 | Fibroblasts-M6 | turquoise |
| COL1A1 | Fibroblasts-M9 | pink |
| ABHD17C | Fibroblasts-M5 | salmon |
| COX17 | Fibroblasts-M8 | magenta |
| RPL37A | Fibroblasts-M10 | blue |
| TMEM179B | Fibroblasts-M30 | saddlebrown |
| MRPL33 | Fibroblasts-M16 | lightcyan |
| PSMA4 | Fibroblasts-M2 | green |
| PSMD11 | Fibroblasts-M14 | tan |
| DDX24 | Fibroblasts-M6 | turquoise |
| LTB | Fibroblasts-M24 | midnightblue |
| SNHG8 | Fibroblasts-M11 | royalblue |
| TMPRSS2 | Fibroblasts-M30 | saddlebrown |
| CPM | Fibroblasts-M21 | brown |
| ATF3 | Fibroblasts-M6 | turquoise |
| MRPL34 | Fibroblasts-M25 | greenyellow |
| AP1G2 | Fibroblasts-M28 | skyblue |
| ERP44 | Fibroblasts-M6 | turquoise |
| CCNDBP1 | Fibroblasts-M28 | skyblue |
| COX8A | Fibroblasts-M16 | lightcyan |
| TPSB2 | Fibroblasts-M10 | blue |
| VPREB3 | Fibroblasts-M10 | blue |
| SBDS | Fibroblasts-M25 | greenyellow |
| MICA | Fibroblasts-M6 | turquoise |
| TRBC2 | Fibroblasts-M10 | blue |
| TCP1 | Fibroblasts-M6 | turquoise |
| RALBP1 | Fibroblasts-M25 | greenyellow |
| NUDCD2 | Fibroblasts-M8 | magenta |
| TOMM7 | Fibroblasts-M11 | royalblue |
| PSMB9 | Fibroblasts-M2 | green |
| HSD11B2 | Fibroblasts-M21 | brown |
| G3BP1 | Fibroblasts-M6 | turquoise |
| AC016739.1 | Fibroblasts-M10 | blue |
| HTRA2 | Fibroblasts-M22 | paleturquoise |
| CLIC1 | Fibroblasts-M8 | magenta |
| TRA2B | Fibroblasts-M6 | turquoise |
| IFI16 | Fibroblasts-M15 | grey60 |
| CRACR2B | Fibroblasts-M6 | turquoise |
| TMEM258 | Fibroblasts-M10 | blue |
| R3HDM2 | Fibroblasts-M27 | darkorange |
| NBEAL1 | Fibroblasts-M1 | purple |
| UBE2D1 | Fibroblasts-M6 | turquoise |
| HSPD1 | Fibroblasts-M6 | turquoise |
| C16orf54 | Fibroblasts-M10 | blue |
| CCDC137 | Fibroblasts-M4 | yellow |
| RPL26 | Fibroblasts-M10 | blue |
| ITGB7 | Fibroblasts-M24 | midnightblue |
| SPG7 | Fibroblasts-M9 | pink |
| PDE7A | Fibroblasts-M1 | purple |
| TCOF1 | Fibroblasts-M4 | yellow |
| ARRDC3 | Fibroblasts-M13 | black |
| PTP4A1 | Fibroblasts-M23 | darkturquoise |
| POMP | Fibroblasts-M1 | purple |
| ELAVL1 | Fibroblasts-M19 | orange |
| S100A4 | Fibroblasts-M16 | lightcyan |
| MIEN1 | Fibroblasts-M18 | white |
| NR4A2 | Fibroblasts-M6 | turquoise |
| PPP1R15A | Fibroblasts-M6 | turquoise |
| THOC6 | Fibroblasts-M16 | lightcyan |
| AC034236.1 | Fibroblasts-M10 | blue |
| TFG | Fibroblasts-M31 | darkred |
| PDXDC1 | Fibroblasts-M9 | pink |
| BLNK | Fibroblasts-M6 | turquoise |
| TMEM259 | Fibroblasts-M6 | turquoise |
| PHLDA1 | Fibroblasts-M6 | turquoise |
| LAPTM4A | Fibroblasts-M7 | cyan |
| NR4A1 | Fibroblasts-M6 | turquoise |
| GNB1 | Fibroblasts-M29 | darkgreen |
| RRP36 | Fibroblasts-M1 | purple |
| NSRP1 | Fibroblasts-M22 | paleturquoise |
| FOSB | Fibroblasts-M6 | turquoise |
| MRPL12 | Fibroblasts-M16 | lightcyan |
| SYNGR2 | Fibroblasts-M1 | purple |
| ATP8B1 | Fibroblasts-M21 | brown |
| KIFAP3 | Fibroblasts-M7 | cyan |
| CKAP5 | Fibroblasts-M7 | cyan |
| PPP1CA | Fibroblasts-M8 | magenta |
| HCG18 | Fibroblasts-M13 | black |
| TMEM14A | Fibroblasts-M16 | lightcyan |
| RF00004 | Fibroblasts-M10 | blue |
| GPATCH4 | Fibroblasts-M5 | salmon |
| C12orf65 | Fibroblasts-M20 | red |
| AUP1 | Fibroblasts-M4 | yellow |
| ZMAT2 | Fibroblasts-M20 | red |
| NUSAP1 | Fibroblasts-M4 | yellow |
| CD2 | Fibroblasts-M24 | midnightblue |
| SLC2A1 | Fibroblasts-M13 | black |
| AKAP17A | Fibroblasts-M3 | lightyellow |
| TMBIM4 | Fibroblasts-M10 | blue |
| CTTNBP2NL | Fibroblasts-M5 | salmon |
| PHLDA2 | Fibroblasts-M1 | purple |
| RAD54L2 | Fibroblasts-M6 | turquoise |
| DYNC1LI2 | Fibroblasts-M31 | darkred |
| NDUFA6 | Fibroblasts-M8 | magenta |
| RGCC | Fibroblasts-M6 | turquoise |
| VIM | Fibroblasts-M25 | greenyellow |
| NDUFB4 | Fibroblasts-M26 | lightgreen |
| ITM2A | Fibroblasts-M10 | blue |
| PIM3 | Fibroblasts-M6 | turquoise |
| HDGF | Fibroblasts-M1 | purple |
| KLRB1 | Fibroblasts-M17 | darkgrey |
| SYTL2 | Fibroblasts-M8 | magenta |
| ADGRE5 | Fibroblasts-M6 | turquoise |
| NADSYN1 | Fibroblasts-M20 | red |
| COMMD4 | Fibroblasts-M25 | greenyellow |
| HERPUD1 | Fibroblasts-M13 | black |
| DUSP5 | Fibroblasts-M6 | turquoise |
| MCL1 | Fibroblasts-M6 | turquoise |
| RNF168 | Fibroblasts-M31 | darkred |
| GYPC | Fibroblasts-M10 | blue |
| AGTPBP1 | Fibroblasts-M25 | greenyellow |
| ABI2 | Fibroblasts-M21 | brown |
| APLP2 | Fibroblasts-M6 | turquoise |
| FAM120AOS | Fibroblasts-M9 | pink |
| AL390728.4 | Fibroblasts-M8 | magenta |
| DDX5 | Fibroblasts-M2 | green |
| PPIG | Fibroblasts-M2 | green |
| PUS7L | Fibroblasts-M22 | paleturquoise |
| NUDT5 | Fibroblasts-M1 | purple |
| MRPS26 | Fibroblasts-M1 | purple |
| WDR70 | Fibroblasts-M10 | blue |
| GSTP1 | Fibroblasts-M22 | paleturquoise |
| FAM98C | Fibroblasts-M24 | midnightblue |
| ZMYND8 | Fibroblasts-M27 | darkorange |
| GZMA | Fibroblasts-M28 | skyblue |
| THAP6 | Fibroblasts-M25 | greenyellow |
| MPHOSPH10 | Fibroblasts-M20 | red |
| ZNF276 | Fibroblasts-M2 | green |
| MRPL41 | Fibroblasts-M16 | lightcyan |
| SH3KBP1 | Fibroblasts-M28 | skyblue |
| DCN | Fibroblasts-M20 | red |
| TAF1D | Fibroblasts-M6 | turquoise |
| APRT | Fibroblasts-M10 | blue |
| GOLGA2 | Fibroblasts-M9 | pink |
| HSPA5 | Fibroblasts-M6 | turquoise |
| ZRANB2 | Fibroblasts-M20 | red |
| TBCB | Fibroblasts-M16 | lightcyan |
| KLF5 | Fibroblasts-M6 | turquoise |
| SEPT7P2 | Fibroblasts-M21 | brown |
| SVIL | Fibroblasts-M2 | green |
| EPB41L4A-AS1 | Fibroblasts-M10 | blue |
| SAMHD1 | Fibroblasts-M21 | brown |
| TMEM109 | Fibroblasts-M29 | darkgreen |
| MGLL | Fibroblasts-M6 | turquoise |
| EID1 | Fibroblasts-M28 | skyblue |
| CKB | Fibroblasts-M21 | brown |
| IGLC3 | Fibroblasts-M10 | blue |
| DNAJB11 | Fibroblasts-M6 | turquoise |
| TMBIM6 | Fibroblasts-M23 | darkturquoise |
| MACF1 | Fibroblasts-M29 | darkgreen |
| UPP1 | Fibroblasts-M1 | purple |
| MEF2C | Fibroblasts-M21 | brown |
| EIF3I | Fibroblasts-M8 | magenta |
| PARD6G-AS1 | Fibroblasts-M6 | turquoise |
| SLC4A1AP | Fibroblasts-M31 | darkred |
| ATP5PD | Fibroblasts-M8 | magenta |
| SYNRG | Fibroblasts-M9 | pink |
| MYDGF | Fibroblasts-M26 | lightgreen |
| HSPA1A | Fibroblasts-M6 | turquoise |
| TMEM59 | Fibroblasts-M2 | green |
| TOMM20 | Fibroblasts-M13 | black |
| CD151 | Fibroblasts-M16 | lightcyan |
| BSG | Fibroblasts-M17 | darkgrey |
| RPS20 | Fibroblasts-M11 | royalblue |
| ABHD14B | Fibroblasts-M21 | brown |
| CUTALP | Fibroblasts-M25 | greenyellow |
| NME6 | Fibroblasts-M12 | steelblue |
| CKLF | Fibroblasts-M4 | yellow |
| EEF1D | Fibroblasts-M25 | greenyellow |
| UBE2D3 | Fibroblasts-M13 | black |
| YME1L1 | Fibroblasts-M1 | purple |
| HSPE1 | Fibroblasts-M6 | turquoise |
| MUC12 | Fibroblasts-M6 | turquoise |
| CASD1 | Fibroblasts-M5 | salmon |
| MDFIC | Fibroblasts-M7 | cyan |
| SNX1 | Fibroblasts-M29 | darkgreen |
| KRT10 | Fibroblasts-M26 | lightgreen |
| ARPC3 | Fibroblasts-M21 | brown |
| PRDX4 | Fibroblasts-M19 | orange |
| LINC01871 | Fibroblasts-M10 | blue |
| TXNL4A | Fibroblasts-M16 | lightcyan |
| TMPO | Fibroblasts-M4 | yellow |
| FIP1L1 | Fibroblasts-M20 | red |
| TNFSF13B | Fibroblasts-M7 | cyan |
| REX1BD | Fibroblasts-M20 | red |
| RPL3P4 | Fibroblasts-M10 | blue |
| TRAC | Fibroblasts-M10 | blue |
| RPL12 | Fibroblasts-M11 | royalblue |
| APOE | Fibroblasts-M10 | blue |
| HSPH1 | Fibroblasts-M6 | turquoise |
| HLA-C | Fibroblasts-M2 | green |
| EXOSC9 | Fibroblasts-M4 | yellow |
| PDCD10 | Fibroblasts-M25 | greenyellow |
| SCP2 | Fibroblasts-M20 | red |
| AC025164.1 | Fibroblasts-M10 | blue |
| ATP5MG | Fibroblasts-M16 | lightcyan |
| RAD9A | Fibroblasts-M9 | pink |
| IQGAP1 | Fibroblasts-M8 | magenta |
| UBE2A | Fibroblasts-M4 | yellow |
| FNDC3B | Fibroblasts-M9 | pink |
| CDC37L1 | Fibroblasts-M2 | green |
| FRG1BP | Fibroblasts-M10 | blue |
| SAMD9 | Fibroblasts-M5 | salmon |
| NUPR1 | Fibroblasts-M7 | cyan |
| CSRNP1 | Fibroblasts-M6 | turquoise |
| PBX3 | Fibroblasts-M7 | cyan |
| LAMTOR3 | Fibroblasts-M1 | purple |
| CCDC69 | Fibroblasts-M10 | blue |
| DDX6 | Fibroblasts-M23 | darkturquoise |
| MCRIP1 | Fibroblasts-M30 | saddlebrown |
| ZNF207 | Fibroblasts-M2 | green |
| NUDT15 | Fibroblasts-M22 | paleturquoise |
| PHTF2 | Fibroblasts-M14 | tan |
| SKAP1 | Fibroblasts-M25 | greenyellow |
| RNASE1 | Fibroblasts-M25 | greenyellow |
| KDELR2 | Fibroblasts-M14 | tan |
| RPL36 | Fibroblasts-M11 | royalblue |
| SUGT1 | Fibroblasts-M1 | purple |
| SON | Fibroblasts-M7 | cyan |
| ATP5ME | Fibroblasts-M10 | blue |
| FBLIM1 | Fibroblasts-M23 | darkturquoise |
| ATF7IP | Fibroblasts-M15 | grey60 |
| CRIM1 | Fibroblasts-M8 | magenta |
| VPS26B | Fibroblasts-M19 | orange |
| DOCK11 | Fibroblasts-M21 | brown |
| PSMA3 | Fibroblasts-M1 | purple |
| STK17A | Fibroblasts-M19 | orange |
| LSP1 | Fibroblasts-M10 | blue |
| CDV3 | Fibroblasts-M6 | turquoise |
| LRMP | Fibroblasts-M3 | lightyellow |
| DNAJC8 | Fibroblasts-M4 | yellow |
| TBCC | Fibroblasts-M21 | brown |
| TXNDC17 | Fibroblasts-M26 | lightgreen |
| 1-Sep | Fibroblasts-M25 | greenyellow |
| TMA16 | Fibroblasts-M15 | grey60 |
| MCM7 | Fibroblasts-M4 | yellow |
| ITGA4 | Fibroblasts-M21 | brown |
| MGST3 | Fibroblasts-M27 | darkorange |
| IK | Fibroblasts-M28 | skyblue |
| FLNA | Fibroblasts-M8 | magenta |
| CHURC1 | Fibroblasts-M25 | greenyellow |
| TIMP3 | Fibroblasts-M14 | tan |
| ACSS2 | Fibroblasts-M25 | greenyellow |
| MUC2 | Fibroblasts-M10 | blue |
| APPL1 | Fibroblasts-M7 | cyan |
| SRSF10 | Fibroblasts-M4 | yellow |
| PTGES2 | Fibroblasts-M28 | skyblue |
| A2M | Fibroblasts-M2 | green |
| USP38 | Fibroblasts-M18 | white |
| FMNL1 | Fibroblasts-M10 | blue |
| NDUFV1 | Fibroblasts-M21 | brown |
| IP6K2 | Fibroblasts-M7 | cyan |
| TMEM107 | Fibroblasts-M27 | darkorange |
| FXYD5 | Fibroblasts-M14 | tan |
| ACTG1 | Fibroblasts-M8 | magenta |
| OXNAD1 | Fibroblasts-M26 | lightgreen |
| ATRAID | Fibroblasts-M20 | red |
| RASL11A | Fibroblasts-M25 | greenyellow |
| FBL | Fibroblasts-M1 | purple |
| ZNF195 | Fibroblasts-M1 | purple |
| HIST2H2BE | Fibroblasts-M30 | saddlebrown |
| RARS | Fibroblasts-M26 | lightgreen |
| GRIPAP1 | Fibroblasts-M20 | red |
| C9orf78 | Fibroblasts-M2 | green |
| RPL22 | Fibroblasts-M11 | royalblue |
| HCST | Fibroblasts-M24 | midnightblue |
| NCOA7 | Fibroblasts-M6 | turquoise |
| NAP1L4 | Fibroblasts-M27 | darkorange |
| MS4A1 | Fibroblasts-M10 | blue |
| ARPC1A | Fibroblasts-M25 | greenyellow |
| PRPF4B | Fibroblasts-M29 | darkgreen |
| AC020916.1 | Fibroblasts-M6 | turquoise |
| CHCHD10 | Fibroblasts-M8 | magenta |
| CLDN4 | Fibroblasts-M23 | darkturquoise |
| CAPRIN1 | Fibroblasts-M29 | darkgreen |
| TERF1 | Fibroblasts-M22 | paleturquoise |
| RRBP1 | Fibroblasts-M20 | red |
| AL031864.2 | Fibroblasts-M10 | blue |
| EIF4A2 | Fibroblasts-M6 | turquoise |
| FAM133B | Fibroblasts-M17 | darkgrey |
| LGALS3 | Fibroblasts-M10 | blue |
| ADRM1 | Fibroblasts-M31 | darkred |
| PRELID1 | Fibroblasts-M26 | lightgreen |
| PSME1 | Fibroblasts-M1 | purple |
| BDH2 | Fibroblasts-M20 | red |
| LCN2 | Fibroblasts-M25 | greenyellow |
| SNORD13 | Fibroblasts-M3 | lightyellow |
| SMIM29 | Fibroblasts-M13 | black |
| CBX3 | Fibroblasts-M6 | turquoise |
| AHNAK | Fibroblasts-M15 | grey60 |
| CD63 | Fibroblasts-M7 | cyan |
| MAL2 | Fibroblasts-M28 | skyblue |
| SCAF11 | Fibroblasts-M15 | grey60 |
| PRIM1 | Fibroblasts-M4 | yellow |
| DNAJA1 | Fibroblasts-M6 | turquoise |
| CD46 | Fibroblasts-M1 | purple |
| CTSS | Fibroblasts-M2 | green |
| ALG5 | Fibroblasts-M4 | yellow |
| EIF2AK2 | Fibroblasts-M20 | red |
| SLC20A1 | Fibroblasts-M26 | lightgreen |
| PMPCB | Fibroblasts-M23 | darkturquoise |
| CFAP97 | Fibroblasts-M9 | pink |
| CD160 | Fibroblasts-M19 | orange |
| RNF125 | Fibroblasts-M10 | blue |
| RPS21 | Fibroblasts-M11 | royalblue |
| PSMB5 | Fibroblasts-M18 | white |
| SRSF11 | Fibroblasts-M20 | red |
| HSBP1 | Fibroblasts-M16 | lightcyan |
| SMDT1 | Fibroblasts-M21 | brown |
| CCND2 | Fibroblasts-M7 | cyan |
| HNMT | Fibroblasts-M18 | white |
| PAPOLA | Fibroblasts-M12 | steelblue |
| CST3 | Fibroblasts-M10 | blue |
| VPS4B | Fibroblasts-M1 | purple |
| ADIRF | Fibroblasts-M21 | brown |
| VPS41 | Fibroblasts-M9 | pink |
| LAPTM5 | Fibroblasts-M21 | brown |
| PUS7 | Fibroblasts-M13 | black |
| RCN1 | Fibroblasts-M2 | green |
| MUM1 | Fibroblasts-M2 | green |
| GABARAPL2 | Fibroblasts-M13 | black |
| PRDX6 | Fibroblasts-M22 | paleturquoise |
| EIF3M | Fibroblasts-M13 | black |
| SNRPA1 | Fibroblasts-M26 | lightgreen |
| COX10 | Fibroblasts-M1 | purple |
| THOC2 | Fibroblasts-M12 | steelblue |
| MGAT4A | Fibroblasts-M2 | green |
| FAUP1 | Fibroblasts-M10 | blue |
| VAMP2 | Fibroblasts-M25 | greenyellow |
| HEBP2 | Fibroblasts-M19 | orange |
| RBM17 | Fibroblasts-M6 | turquoise |
| TMEM256 | Fibroblasts-M10 | blue |
| FAT1 | Fibroblasts-M21 | brown |
| MRE11 | Fibroblasts-M14 | tan |
| COX4I1 | Fibroblasts-M21 | brown |
| HMGCS2 | Fibroblasts-M30 | saddlebrown |
| MAT2B | Fibroblasts-M25 | greenyellow |
| AGPS | Fibroblasts-M2 | green |
| HAX1 | Fibroblasts-M4 | yellow |
| PNP | Fibroblasts-M26 | lightgreen |
| DUSP2 | Fibroblasts-M24 | midnightblue |
| METTL26 | Fibroblasts-M17 | darkgrey |
| MRPL51 | Fibroblasts-M4 | yellow |
| GRWD1 | Fibroblasts-M21 | brown |
| BCL2A1 | Fibroblasts-M22 | paleturquoise |
| TCTN3 | Fibroblasts-M9 | pink |
| C6orf120 | Fibroblasts-M20 | red |
| HOXB9 | Fibroblasts-M25 | greenyellow |
| PLIN2 | Fibroblasts-M6 | turquoise |
| PRAF2 | Fibroblasts-M19 | orange |
| SNHG18 | Fibroblasts-M20 | red |
| USP1 | Fibroblasts-M4 | yellow |
| ATP5MPL | Fibroblasts-M16 | lightcyan |
| PPP1R15B | Fibroblasts-M6 | turquoise |
| MBNL1 | Fibroblasts-M29 | darkgreen |
| IMP3 | Fibroblasts-M11 | royalblue |
| ASPM | Fibroblasts-M4 | yellow |
| KRT18 | Fibroblasts-M8 | magenta |
| FXR1 | Fibroblasts-M24 | midnightblue |
| HELLS | Fibroblasts-M4 | yellow |
| CSK | Fibroblasts-M22 | paleturquoise |
| TFAM | Fibroblasts-M2 | green |
| PLCE1 | Fibroblasts-M21 | brown |
| PSIP1 | Fibroblasts-M10 | blue |
| GGNBP2 | Fibroblasts-M14 | tan |
| CDK2AP2 | Fibroblasts-M29 | darkgreen |
| TRAF3 | Fibroblasts-M9 | pink |
| RPL38 | Fibroblasts-M10 | blue |
| MZB1 | Fibroblasts-M3 | lightyellow |
| MYL12A | Fibroblasts-M8 | magenta |
| RBM25 | Fibroblasts-M9 | pink |
| HIST1H1B | Fibroblasts-M4 | yellow |
| FAM192A | Fibroblasts-M2 | green |
| LMF2 | Fibroblasts-M27 | darkorange |
| NDUFC2 | Fibroblasts-M7 | cyan |
| RABGGTA | Fibroblasts-M25 | greenyellow |
| LIPH | Fibroblasts-M9 | pink |
| LYZ | Fibroblasts-M25 | greenyellow |
| DEDD2 | Fibroblasts-M13 | black |
| RNF181 | Fibroblasts-M25 | greenyellow |
| MFN1 | Fibroblasts-M22 | paleturquoise |
| PKN2 | Fibroblasts-M2 | green |
| CGRRF1 | Fibroblasts-M17 | darkgrey |
| CDK11B | Fibroblasts-M24 | midnightblue |
| GADD45GIP1 | Fibroblasts-M16 | lightcyan |
| CCT4 | Fibroblasts-M6 | turquoise |
| FAM84A | Fibroblasts-M14 | tan |
| BCL2 | Fibroblasts-M8 | magenta |
| CLIC3 | Fibroblasts-M7 | cyan |
| REST | Fibroblasts-M13 | black |
| ARFIP1 | Fibroblasts-M5 | salmon |
| MBP | Fibroblasts-M10 | blue |
| USP16 | Fibroblasts-M25 | greenyellow |
| PTTG1 | Fibroblasts-M4 | yellow |
| PCED1B-AS1 | Fibroblasts-M10 | blue |
| REV3L | Fibroblasts-M6 | turquoise |
| TBC1D7 | Fibroblasts-M14 | tan |
| LENG8 | Fibroblasts-M24 | midnightblue |
| ERO1A | Fibroblasts-M13 | black |
| TBL3 | Fibroblasts-M6 | turquoise |
| RNF149 | Fibroblasts-M20 | red |
| HSP90B1 | Fibroblasts-M6 | turquoise |
| KIF22 | Fibroblasts-M28 | skyblue |
| NDUFV3 | Fibroblasts-M27 | darkorange |
| GSTO1 | Fibroblasts-M26 | lightgreen |
| DSTN | Fibroblasts-M8 | magenta |
| PRDM2 | Fibroblasts-M7 | cyan |
| NDUFB2 | Fibroblasts-M16 | lightcyan |
| GMFG | Fibroblasts-M8 | magenta |
| CD36 | Fibroblasts-M21 | brown |
| MSL2 | Fibroblasts-M2 | green |
| CXCL2 | Fibroblasts-M6 | turquoise |
| TRIM56 | Fibroblasts-M9 | pink |
| PPDPF | Fibroblasts-M16 | lightcyan |
| RANBP1 | Fibroblasts-M4 | yellow |
| SSBP1 | Fibroblasts-M26 | lightgreen |
| HACD3 | Fibroblasts-M20 | red |
| CEBPZ | Fibroblasts-M13 | black |
| DHX38 | Fibroblasts-M7 | cyan |
| GPX1 | Fibroblasts-M16 | lightcyan |
| MLLT3 | Fibroblasts-M20 | red |
| UQCR11 | Fibroblasts-M10 | blue |
| MGMT | Fibroblasts-M28 | skyblue |
| SKP1 | Fibroblasts-M16 | lightcyan |
| JUND | Fibroblasts-M6 | turquoise |
| CEBPD | Fibroblasts-M6 | turquoise |
| MAGED1 | Fibroblasts-M9 | pink |
| TRADD | Fibroblasts-M10 | blue |
| CD53 | Fibroblasts-M25 | greenyellow |
| TNFAIP3 | Fibroblasts-M6 | turquoise |
| KRR1 | Fibroblasts-M2 | green |
| LINC-PINT | Fibroblasts-M13 | black |
| VSIR | Fibroblasts-M10 | blue |
| STMN1 | Fibroblasts-M4 | yellow |
| EIF2AK4 | Fibroblasts-M28 | skyblue |
| PARP14 | Fibroblasts-M2 | green |
| PRMT2 | Fibroblasts-M7 | cyan |
| CFD | Fibroblasts-M10 | blue |
| C11orf49 | Fibroblasts-M17 | darkgrey |
| RBX1 | Fibroblasts-M25 | greenyellow |
| RPSA | Fibroblasts-M13 | black |
| ISG15 | Fibroblasts-M2 | green |
| SFR1 | Fibroblasts-M2 | green |
| ELOB | Fibroblasts-M8 | magenta |
| NCF4 | Fibroblasts-M24 | midnightblue |
| HIST1H1C | Fibroblasts-M4 | yellow |
| HSPB1 | Fibroblasts-M6 | turquoise |
| TINAGL1 | Fibroblasts-M21 | brown |
| ZNF326 | Fibroblasts-M6 | turquoise |
| CD59 | Fibroblasts-M8 | magenta |
| DAZAP2 | Fibroblasts-M2 | green |
| CASP8 | Fibroblasts-M5 | salmon |
| UBA1 | Fibroblasts-M1 | purple |
| TM7SF3 | Fibroblasts-M2 | green |
| URI1 | Fibroblasts-M12 | steelblue |
| VPS35L | Fibroblasts-M9 | pink |
| CDH17 | Fibroblasts-M23 | darkturquoise |
| AC099560.2 | Fibroblasts-M10 | blue |
| TMX1 | Fibroblasts-M4 | yellow |
| TYMP | Fibroblasts-M24 | midnightblue |
| YTHDC2 | Fibroblasts-M24 | midnightblue |
| RAB2A | Fibroblasts-M14 | tan |
| CAPN8 | Fibroblasts-M30 | saddlebrown |
| HBA1 | Fibroblasts-M25 | greenyellow |
| DNAJA4 | Fibroblasts-M1 | purple |
| ZNF267 | Fibroblasts-M6 | turquoise |
| C9orf16 | Fibroblasts-M6 | turquoise |
| CD48 | Fibroblasts-M21 | brown |
| MARS | Fibroblasts-M6 | turquoise |
| RALA | Fibroblasts-M13 | black |
| C4orf3 | Fibroblasts-M1 | purple |
| LRPAP1 | Fibroblasts-M10 | blue |
| TLN2 | Fibroblasts-M9 | pink |
| CCL3 | Fibroblasts-M25 | greenyellow |
| KCNAB2 | Fibroblasts-M4 | yellow |
| NDUFC1 | Fibroblasts-M22 | paleturquoise |
| RAB32 | Fibroblasts-M19 | orange |
| STAT6 | Fibroblasts-M2 | green |
| MAN1A2 | Fibroblasts-M5 | salmon |
| HNRNPA1 | Fibroblasts-M4 | yellow |
| DDIT4 | Fibroblasts-M13 | black |
| RNH1 | Fibroblasts-M11 | royalblue |
| FAM120A | Fibroblasts-M9 | pink |
| DNAJB4 | Fibroblasts-M6 | turquoise |
| PDE4B | Fibroblasts-M6 | turquoise |
| AK3 | Fibroblasts-M2 | green |
| SLC2A3 | Fibroblasts-M6 | turquoise |
| TSEN34 | Fibroblasts-M20 | red |
| RHOF | Fibroblasts-M30 | saddlebrown |
| TOB2 | Fibroblasts-M2 | green |
| APOBEC3G | Fibroblasts-M6 | turquoise |
| ATAD2 | Fibroblasts-M4 | yellow |
| LSM5 | Fibroblasts-M25 | greenyellow |
| ARPP19 | Fibroblasts-M12 | steelblue |
| IL1R2 | Fibroblasts-M4 | yellow |
| DGUOK | Fibroblasts-M1 | purple |
| CHORDC1 | Fibroblasts-M6 | turquoise |
| ILF3 | Fibroblasts-M24 | midnightblue |
| STK17B | Fibroblasts-M14 | tan |
| SLC4A7 | Fibroblasts-M14 | tan |
| PAG1 | Fibroblasts-M6 | turquoise |
| SPTY2D1 | Fibroblasts-M13 | black |
| TMEM2 | Fibroblasts-M5 | salmon |
| NFIA | Fibroblasts-M10 | blue |
| SULT1A1 | Fibroblasts-M10 | blue |
| LMO4 | Fibroblasts-M10 | blue |
| PDLIM2 | Fibroblasts-M7 | cyan |
| AHCYL1 | Fibroblasts-M5 | salmon |
| TM9SF4 | Fibroblasts-M20 | red |
| ST8SIA4 | Fibroblasts-M3 | lightyellow |
| CNPY2 | Fibroblasts-M22 | paleturquoise |
| CHD9 | Fibroblasts-M15 | grey60 |
| CORO1C | Fibroblasts-M26 | lightgreen |
| SMC2 | Fibroblasts-M4 | yellow |
| TATDN1 | Fibroblasts-M18 | white |
| DPP7 | Fibroblasts-M10 | blue |
| SOD1 | Fibroblasts-M25 | greenyellow |
| TPM4 | Fibroblasts-M8 | magenta |
| ATAD5 | Fibroblasts-M4 | yellow |
| RAB5A | Fibroblasts-M1 | purple |
| BUB1 | Fibroblasts-M4 | yellow |
| COX6A1 | Fibroblasts-M18 | white |
| NENF | Fibroblasts-M10 | blue |
| SF3B2 | Fibroblasts-M28 | skyblue |
| CFL2 | Fibroblasts-M8 | magenta |
| GPNMB | Fibroblasts-M7 | cyan |
| HNRNPA0 | Fibroblasts-M6 | turquoise |
| GNAQ | Fibroblasts-M9 | pink |
| CACYBP | Fibroblasts-M6 | turquoise |
| RPL21P93 | Fibroblasts-M3 | lightyellow |
| SLIRP | Fibroblasts-M16 | lightcyan |
| APOL6 | Fibroblasts-M5 | salmon |
| RPS25 | Fibroblasts-M10 | blue |
| Z93241.1 | Fibroblasts-M3 | lightyellow |
| GTF2I | Fibroblasts-M7 | cyan |
| ZCCHC6 | Fibroblasts-M9 | pink |
| SNRPE | Fibroblasts-M4 | yellow |
| 2-Sep | Fibroblasts-M15 | grey60 |
| KLHDC2 | Fibroblasts-M28 | skyblue |
| PLA2G2A | Fibroblasts-M10 | blue |
| SELENOK | Fibroblasts-M13 | black |
| CDK4 | Fibroblasts-M4 | yellow |
| MITD1 | Fibroblasts-M17 | darkgrey |
| IL32 | Fibroblasts-M26 | lightgreen |
| SLAIN2 | Fibroblasts-M26 | lightgreen |
| RAB11B | Fibroblasts-M25 | greenyellow |
| NARS | Fibroblasts-M2 | green |
| CD96 | Fibroblasts-M24 | midnightblue |
| MPP7 | Fibroblasts-M6 | turquoise |
| CCSER2 | Fibroblasts-M8 | magenta |
| PABPC1P3 | Fibroblasts-M10 | blue |
| RPL26P19 | Fibroblasts-M10 | blue |
| RNASEH2B | Fibroblasts-M27 | darkorange |
| CLASRP | Fibroblasts-M13 | black |
| CDK12 | Fibroblasts-M2 | green |
| RAN | Fibroblasts-M26 | lightgreen |
| TSPAN14 | Fibroblasts-M1 | purple |
| ACTN4 | Fibroblasts-M8 | magenta |
| CD82 | Fibroblasts-M6 | turquoise |
| MDH2 | Fibroblasts-M18 | white |
| CYSLTR1 | Fibroblasts-M2 | green |
| CLPTM1L | Fibroblasts-M17 | darkgrey |
| ID3 | Fibroblasts-M1 | purple |
| SVIP | Fibroblasts-M3 | lightyellow |
| TPM1 | Fibroblasts-M8 | magenta |
| RNF126 | Fibroblasts-M25 | greenyellow |
| CST7 | Fibroblasts-M24 | midnightblue |
| TRAM1 | Fibroblasts-M14 | tan |
| PBX1 | Fibroblasts-M7 | cyan |
| TSEN54 | Fibroblasts-M20 | red |
| SSR3 | Fibroblasts-M14 | tan |
| SDHC | Fibroblasts-M5 | salmon |
| SIAH2 | Fibroblasts-M6 | turquoise |
| MYC | Fibroblasts-M1 | purple |
| AP2M1 | Fibroblasts-M25 | greenyellow |
| ANAPC4 | Fibroblasts-M6 | turquoise |
| SERBP1 | Fibroblasts-M1 | purple |
| CMBL | Fibroblasts-M6 | turquoise |
| C14orf119 | Fibroblasts-M5 | salmon |
| TLN1 | Fibroblasts-M8 | magenta |
| GNG2 | Fibroblasts-M6 | turquoise |
| MFGE8 | Fibroblasts-M1 | purple |
| NDUFB6 | Fibroblasts-M18 | white |
| SMPDL3A | Fibroblasts-M20 | red |
| NLRP1 | Fibroblasts-M6 | turquoise |
| NUF2 | Fibroblasts-M4 | yellow |
| SP140 | Fibroblasts-M21 | brown |
| GATA3 | Fibroblasts-M24 | midnightblue |
| FBXW11 | Fibroblasts-M26 | lightgreen |
| C1orf35 | Fibroblasts-M21 | brown |
| HACL1 | Fibroblasts-M28 | skyblue |
| GPR183 | Fibroblasts-M3 | lightyellow |
| USP34 | Fibroblasts-M15 | grey60 |
| TAX1BP3 | Fibroblasts-M16 | lightcyan |
| ARF1 | Fibroblasts-M29 | darkgreen |
| ATP6V0B | Fibroblasts-M31 | darkred |
| VMP1 | Fibroblasts-M14 | tan |
| TMEM50A | Fibroblasts-M8 | magenta |
| RAB4A | Fibroblasts-M27 | darkorange |
| RSBN1L | Fibroblasts-M2 | green |
| DUT | Fibroblasts-M4 | yellow |
| STK4 | Fibroblasts-M13 | black |
| ZFC3H1 | Fibroblasts-M6 | turquoise |
| FARP1 | Fibroblasts-M23 | darkturquoise |
| LAT2 | Fibroblasts-M7 | cyan |
| MUS81 | Fibroblasts-M24 | midnightblue |
| NPDC1 | Fibroblasts-M10 | blue |
| SAP18 | Fibroblasts-M1 | purple |
| CD44 | Fibroblasts-M13 | black |
| IL27RA | Fibroblasts-M6 | turquoise |
| SNU13 | Fibroblasts-M13 | black |
| RIN3 | Fibroblasts-M24 | midnightblue |
| UQCRH | Fibroblasts-M16 | lightcyan |
| PAWR | Fibroblasts-M8 | magenta |
| TPI1 | Fibroblasts-M26 | lightgreen |
| SNHG25 | Fibroblasts-M16 | lightcyan |
| SELENOP | Fibroblasts-M7 | cyan |
| FAM102A | Fibroblasts-M7 | cyan |
| MPRIP | Fibroblasts-M8 | magenta |
| RHOH | Fibroblasts-M3 | lightyellow |
| ZKSCAN1 | Fibroblasts-M5 | salmon |
| SELENOT | Fibroblasts-M2 | green |
| EGR1 | Fibroblasts-M6 | turquoise |
| HNRNPD | Fibroblasts-M21 | brown |
| ABCC3 | Fibroblasts-M13 | black |
| TAGAP | Fibroblasts-M3 | lightyellow |
| SETX | Fibroblasts-M5 | salmon |
| MRPL18 | Fibroblasts-M6 | turquoise |
| RPL10AP6 | Fibroblasts-M10 | blue |
| ANKRD36BP2 | Fibroblasts-M3 | lightyellow |
| SELENOW | Fibroblasts-M21 | brown |
| WIPI2 | Fibroblasts-M16 | lightcyan |
| GBP4 | Fibroblasts-M2 | green |
| HPCAL1 | Fibroblasts-M6 | turquoise |
| AGPAT5 | Fibroblasts-M1 | purple |
| PXK | Fibroblasts-M17 | darkgrey |
| ZNF644 | Fibroblasts-M20 | red |
| ZNHIT6 | Fibroblasts-M20 | red |
| CXCL1 | Fibroblasts-M6 | turquoise |
| NFATC2IP | Fibroblasts-M20 | red |
| FUCA2 | Fibroblasts-M20 | red |
| ACTR2 | Fibroblasts-M8 | magenta |
| EHD1 | Fibroblasts-M13 | black |
| BAZ1A | Fibroblasts-M2 | green |
| APOBR | Fibroblasts-M21 | brown |
| SYF2 | Fibroblasts-M25 | greenyellow |
| ABCB1 | Fibroblasts-M9 | pink |
| RPL9P9 | Fibroblasts-M30 | saddlebrown |
| EIF3K | Fibroblasts-M25 | greenyellow |
| SFT2D2 | Fibroblasts-M15 | grey60 |
| HNRNPM | Fibroblasts-M4 | yellow |
| SLC46A3 | Fibroblasts-M6 | turquoise |
| SAP30BP | Fibroblasts-M15 | grey60 |
| CDX1 | Fibroblasts-M1 | purple |
| TTC3 | Fibroblasts-M9 | pink |
| ZFR | Fibroblasts-M4 | yellow |
| DRAP1 | Fibroblasts-M18 | white |
| APMAP | Fibroblasts-M5 | salmon |
| GCFC2 | Fibroblasts-M18 | white |
| UBE2J1 | Fibroblasts-M3 | lightyellow |
| CYBA | Fibroblasts-M16 | lightcyan |
| REL | Fibroblasts-M9 | pink |
| AC243960.1 | Fibroblasts-M24 | midnightblue |
| DBI | Fibroblasts-M18 | white |
| RNF167 | Fibroblasts-M27 | darkorange |
| ACADS | Fibroblasts-M20 | red |
| INSR | Fibroblasts-M25 | greenyellow |
| MZT2A | Fibroblasts-M16 | lightcyan |
| BRD2 | Fibroblasts-M6 | turquoise |
| PDLIM7 | Fibroblasts-M19 | orange |
| KLHL24 | Fibroblasts-M2 | green |
| MEI1 | Fibroblasts-M20 | red |
| MLLT6 | Fibroblasts-M27 | darkorange |
| CASP4 | Fibroblasts-M6 | turquoise |
| SSNA1 | Fibroblasts-M4 | yellow |
| CAP1 | Fibroblasts-M8 | magenta |
| VPS13B | Fibroblasts-M29 | darkgreen |
| STIP1 | Fibroblasts-M13 | black |
| DNMT1 | Fibroblasts-M4 | yellow |
| PCMTD2 | Fibroblasts-M9 | pink |
| PSMF1 | Fibroblasts-M28 | skyblue |
| GATAD1 | Fibroblasts-M5 | salmon |
| DST | Fibroblasts-M15 | grey60 |
| ARFGAP3 | Fibroblasts-M12 | steelblue |
| EIF3A | Fibroblasts-M14 | tan |
| MESD | Fibroblasts-M16 | lightcyan |
| FNTA | Fibroblasts-M20 | red |
| GSTM4 | Fibroblasts-M1 | purple |
| COL6A2 | Fibroblasts-M9 | pink |
| CDC34 | Fibroblasts-M28 | skyblue |
| BAZ2B | Fibroblasts-M20 | red |
| RPL13AP5 | Fibroblasts-M10 | blue |
| EIF4A3 | Fibroblasts-M6 | turquoise |
| SYNE1 | Fibroblasts-M15 | grey60 |
| GLT8D1 | Fibroblasts-M9 | pink |
| FNIP1 | Fibroblasts-M13 | black |
| JAML | Fibroblasts-M16 | lightcyan |
| ALYREF | Fibroblasts-M20 | red |
| SSSCA1 | Fibroblasts-M24 | midnightblue |
| OXA1L | Fibroblasts-M5 | salmon |
| SPRED1 | Fibroblasts-M23 | darkturquoise |
| PSMD12 | Fibroblasts-M26 | lightgreen |
| CA2 | Fibroblasts-M8 | magenta |
| VRK1 | Fibroblasts-M4 | yellow |
| CD47 | Fibroblasts-M23 | darkturquoise |
| MRPS23 | Fibroblasts-M17 | darkgrey |
| ZNHIT1 | Fibroblasts-M8 | magenta |
| MAPKAP1 | Fibroblasts-M7 | cyan |
| CDK6 | Fibroblasts-M29 | darkgreen |
| IFT20 | Fibroblasts-M27 | darkorange |
| MEF2A | Fibroblasts-M7 | cyan |
| SNHG5 | Fibroblasts-M6 | turquoise |
| CEACAM1 | Fibroblasts-M24 | midnightblue |
| UACA | Fibroblasts-M8 | magenta |
| TBCA | Fibroblasts-M16 | lightcyan |
| DYNC1I2 | Fibroblasts-M16 | lightcyan |
| THOP1 | Fibroblasts-M19 | orange |
| MED10 | Fibroblasts-M25 | greenyellow |
| MKNK2 | Fibroblasts-M6 | turquoise |
| CDC5L | Fibroblasts-M13 | black |
| PCNT | Fibroblasts-M4 | yellow |
| ATP2B1 | Fibroblasts-M1 | purple |
| PHF1 | Fibroblasts-M25 | greenyellow |
| DSP | Fibroblasts-M26 | lightgreen |
| HSPA9 | Fibroblasts-M13 | black |
| GLUL | Fibroblasts-M9 | pink |
| IL7R | Fibroblasts-M6 | turquoise |
| SEMA3C | Fibroblasts-M7 | cyan |
| IGFBP7 | Fibroblasts-M21 | brown |
| SFT2D1 | Fibroblasts-M16 | lightcyan |
| GADD45G | Fibroblasts-M10 | blue |
| SUMO2 | Fibroblasts-M4 | yellow |
| TUBB | Fibroblasts-M16 | lightcyan |
| PCNA | Fibroblasts-M4 | yellow |
| KMT2E-AS1 | Fibroblasts-M24 | midnightblue |
| HES1 | Fibroblasts-M6 | turquoise |
| UQCRC1 | Fibroblasts-M12 | steelblue |
| SOCS3 | Fibroblasts-M6 | turquoise |
| PPP2R5C | Fibroblasts-M8 | magenta |
| STAU1 | Fibroblasts-M11 | royalblue |
| STXBP3 | Fibroblasts-M13 | black |
| POF1B | Fibroblasts-M13 | black |
| HSD17B12 | Fibroblasts-M29 | darkgreen |
| MECP2 | Fibroblasts-M20 | red |
| ABRACL | Fibroblasts-M14 | tan |
| OXSR1 | Fibroblasts-M26 | lightgreen |
| ENOSF1 | Fibroblasts-M30 | saddlebrown |
| TM9SF3 | Fibroblasts-M9 | pink |
| MED14 | Fibroblasts-M29 | darkgreen |
| GCHFR | Fibroblasts-M25 | greenyellow |
| MGST1 | Fibroblasts-M10 | blue |
| COQ7 | Fibroblasts-M5 | salmon |
| BUB1B | Fibroblasts-M4 | yellow |
| NCOA3 | Fibroblasts-M9 | pink |
| PRSS3 | Fibroblasts-M6 | turquoise |
| GBF1 | Fibroblasts-M26 | lightgreen |
| ANXA5 | Fibroblasts-M14 | tan |
| LGALS9 | Fibroblasts-M9 | pink |
| MNAT1 | Fibroblasts-M1 | purple |
| DERL3 | Fibroblasts-M3 | lightyellow |
| TMX2 | Fibroblasts-M5 | salmon |
| CENPJ | Fibroblasts-M5 | salmon |
| TMED9 | Fibroblasts-M26 | lightgreen |
| PTTG1IP | Fibroblasts-M13 | black |
| SREK1IP1 | Fibroblasts-M20 | red |
| ARL4C | Fibroblasts-M14 | tan |
| RRM2B | Fibroblasts-M25 | greenyellow |
| DSG2 | Fibroblasts-M26 | lightgreen |
| VKORC1 | Fibroblasts-M10 | blue |
| ACTN1 | Fibroblasts-M29 | darkgreen |
| STX12 | Fibroblasts-M14 | tan |
| PYHIN1 | Fibroblasts-M17 | darkgrey |
| SPRY1 | Fibroblasts-M28 | skyblue |
| NPC2 | Fibroblasts-M7 | cyan |
| PPP2R2A | Fibroblasts-M22 | paleturquoise |
| AQR | Fibroblasts-M9 | pink |
| CMPK1 | Fibroblasts-M18 | white |
| SEC11C | Fibroblasts-M3 | lightyellow |
| ATF2 | Fibroblasts-M31 | darkred |
| ANXA1 | Fibroblasts-M6 | turquoise |
| SOS2 | Fibroblasts-M27 | darkorange |
| KIAA0040 | Fibroblasts-M21 | brown |
| RGS2 | Fibroblasts-M6 | turquoise |
| AC007969.1 | Fibroblasts-M10 | blue |
| YWHAQ | Fibroblasts-M31 | darkred |
| CHMP1B | Fibroblasts-M6 | turquoise |
| PPIP5K2 | Fibroblasts-M9 | pink |
| CEP70 | Fibroblasts-M5 | salmon |
| SMARCC2 | Fibroblasts-M2 | green |
| PYCR2 | Fibroblasts-M21 | brown |
| BIRC3 | Fibroblasts-M13 | black |
| ZAP70 | Fibroblasts-M17 | darkgrey |
| KMT2A | Fibroblasts-M15 | grey60 |
| CALM3 | Fibroblasts-M21 | brown |
| SNX17 | Fibroblasts-M8 | magenta |
| ANKRD36BP1 | Fibroblasts-M10 | blue |
| PRKAR1A | Fibroblasts-M21 | brown |
| EIF5 | Fibroblasts-M13 | black |
| IER5 | Fibroblasts-M6 | turquoise |
| CLDND1 | Fibroblasts-M23 | darkturquoise |
| PTBP3 | Fibroblasts-M29 | darkgreen |
| SGK3 | Fibroblasts-M20 | red |
| EBNA1BP2 | Fibroblasts-M1 | purple |
| LBR | Fibroblasts-M21 | brown |
| SLC7A5 | Fibroblasts-M6 | turquoise |
| HSPA13 | Fibroblasts-M13 | black |
| RNVU1-6 | Fibroblasts-M16 | lightcyan |
| LRRC75A-AS1 | Fibroblasts-M11 | royalblue |
| PECAM1 | Fibroblasts-M13 | black |
| LDHA | Fibroblasts-M26 | lightgreen |
| DCK | Fibroblasts-M26 | lightgreen |
| C11orf58 | Fibroblasts-M1 | purple |
| DNAJC9 | Fibroblasts-M4 | yellow |
| GLS | Fibroblasts-M20 | red |
| HEXB | Fibroblasts-M20 | red |
| ENO1 | Fibroblasts-M26 | lightgreen |
| EEF2 | Fibroblasts-M26 | lightgreen |
| TMEM248 | Fibroblasts-M9 | pink |
| RUNX3 | Fibroblasts-M18 | white |
| CLU | Fibroblasts-M10 | blue |
| FRG1HP | Fibroblasts-M10 | blue |
| ANP32E | Fibroblasts-M4 | yellow |
| FCGRT | Fibroblasts-M20 | red |
| SLC12A2 | Fibroblasts-M23 | darkturquoise |
| SLC25A11 | Fibroblasts-M6 | turquoise |
| KLF4 | Fibroblasts-M6 | turquoise |
| DLG1 | Fibroblasts-M15 | grey60 |
| RHOBTB3 | Fibroblasts-M9 | pink |
| C5orf56 | Fibroblasts-M20 | red |
| DNMBP | Fibroblasts-M23 | darkturquoise |
| RSL24D1 | Fibroblasts-M23 | darkturquoise |
| MEF2D | Fibroblasts-M2 | green |
| TFRC | Fibroblasts-M12 | steelblue |
| CEP152 | Fibroblasts-M4 | yellow |
| SPCS3 | Fibroblasts-M14 | tan |
| ISCA1 | Fibroblasts-M25 | greenyellow |
| LAMTOR5 | Fibroblasts-M21 | brown |
| TSPAN1 | Fibroblasts-M6 | turquoise |
| UBE2J2 | Fibroblasts-M25 | greenyellow |
| ARHGEF38 | Fibroblasts-M6 | turquoise |
| EML2 | Fibroblasts-M6 | turquoise |
| DTX3L | Fibroblasts-M5 | salmon |
| PGRMC1 | Fibroblasts-M14 | tan |
| TMF1 | Fibroblasts-M26 | lightgreen |
| AC022706.1 | Fibroblasts-M25 | greenyellow |
| CARD16 | Fibroblasts-M6 | turquoise |
| ENAM | Fibroblasts-M3 | lightyellow |
| NAA50 | Fibroblasts-M6 | turquoise |
| ZCCHC11 | Fibroblasts-M20 | red |
| SLC5A3 | Fibroblasts-M20 | red |
| CD9 | Fibroblasts-M21 | brown |
| LARP4 | Fibroblasts-M5 | salmon |
| GPI | Fibroblasts-M25 | greenyellow |
| USF2 | Fibroblasts-M3 | lightyellow |
| ZBTB20 | Fibroblasts-M9 | pink |
| DDX3X | Fibroblasts-M6 | turquoise |
| SLC25A37 | Fibroblasts-M6 | turquoise |
| VBP1 | Fibroblasts-M22 | paleturquoise |
| USP12 | Fibroblasts-M6 | turquoise |
| PER1 | Fibroblasts-M2 | green |
| ECHDC2 | Fibroblasts-M30 | saddlebrown |
| STX7 | Fibroblasts-M18 | white |
| SLC38A2 | Fibroblasts-M6 | turquoise |
| ILVBL | Fibroblasts-M10 | blue |
| TOR1AIP1 | Fibroblasts-M20 | red |
| HECTD1 | Fibroblasts-M7 | cyan |
| CEP89 | Fibroblasts-M30 | saddlebrown |
| XPO1 | Fibroblasts-M9 | pink |
| CYTOR | Fibroblasts-M8 | magenta |
| MARCKS | Fibroblasts-M15 | grey60 |
| LGALS8 | Fibroblasts-M29 | darkgreen |
| ARID5B | Fibroblasts-M9 | pink |
| SLC50A1 | Fibroblasts-M18 | white |
| DPYD | Fibroblasts-M7 | cyan |
| OTUB1 | Fibroblasts-M8 | magenta |
| NOXO1 | Fibroblasts-M10 | blue |
| TMOD3 | Fibroblasts-M6 | turquoise |
| GFPT1 | Fibroblasts-M14 | tan |
| MRPL14 | Fibroblasts-M16 | lightcyan |
| TMEM159 | Fibroblasts-M27 | darkorange |
| TTC14 | Fibroblasts-M2 | green |
| MAPRE2 | Fibroblasts-M21 | brown |
| BCL7C | Fibroblasts-M21 | brown |
| N4BP1 | Fibroblasts-M9 | pink |
| OSBPL3 | Fibroblasts-M21 | brown |
| RAB18 | Fibroblasts-M26 | lightgreen |
| RAB30 | Fibroblasts-M21 | brown |
| GPX4 | Fibroblasts-M10 | blue |
| SRGAP1 | Fibroblasts-M20 | red |
| RPL37P2 | Fibroblasts-M10 | blue |
| ACYP2 | Fibroblasts-M21 | brown |
| MAD2L1 | Fibroblasts-M4 | yellow |
| TMEM123 | Fibroblasts-M2 | green |
| C19orf33 | Fibroblasts-M10 | blue |
| NDUFAF4 | Fibroblasts-M20 | red |
| HSP90AA2P | Fibroblasts-M6 | turquoise |
| C1S | Fibroblasts-M20 | red |
| RPL41 | Fibroblasts-M11 | royalblue |
| EMB | Fibroblasts-M24 | midnightblue |
| HRH2 | Fibroblasts-M25 | greenyellow |
| FARP2 | Fibroblasts-M23 | darkturquoise |
| CCDC186 | Fibroblasts-M2 | green |
| PLBD1 | Fibroblasts-M20 | red |
| UFM1 | Fibroblasts-M26 | lightgreen |
| PGK1 | Fibroblasts-M26 | lightgreen |
| PREX1 | Fibroblasts-M1 | purple |
| SDCBP2 | Fibroblasts-M6 | turquoise |
| SGCB | Fibroblasts-M2 | green |
| C7orf50 | Fibroblasts-M19 | orange |
| DKC1 | Fibroblasts-M6 | turquoise |
| TGIF1 | Fibroblasts-M6 | turquoise |
| PLA2G16 | Fibroblasts-M6 | turquoise |
| MAP2K7 | Fibroblasts-M27 | darkorange |
| CELF2 | Fibroblasts-M10 | blue |
| RAC1 | Fibroblasts-M8 | magenta |
| SRSF6 | Fibroblasts-M20 | red |
| BAZ1B | Fibroblasts-M20 | red |
| INO80 | Fibroblasts-M20 | red |
| UPF1 | Fibroblasts-M2 | green |
| RAP1B | Fibroblasts-M1 | purple |
| DECR2 | Fibroblasts-M9 | pink |
| RPL30P4 | Fibroblasts-M10 | blue |
| CTNNB1 | Fibroblasts-M6 | turquoise |
| PPM1G | Fibroblasts-M16 | lightcyan |
| SULF2 | Fibroblasts-M9 | pink |
| GSPT1 | Fibroblasts-M8 | magenta |
| SNHG15 | Fibroblasts-M21 | brown |
| MRPL47 | Fibroblasts-M6 | turquoise |
| LSM3 | Fibroblasts-M14 | tan |
| UPF3A | Fibroblasts-M20 | red |
| GMNN | Fibroblasts-M4 | yellow |
| RBM26 | Fibroblasts-M9 | pink |
| MFSD1 | Fibroblasts-M9 | pink |
| WWP1 | Fibroblasts-M6 | turquoise |
| STXBP2 | Fibroblasts-M19 | orange |
| HLA-DRB6 | Fibroblasts-M10 | blue |
| PPCS | Fibroblasts-M20 | red |
| JPT1 | Fibroblasts-M25 | greenyellow |
| TNFRSF12A | Fibroblasts-M26 | lightgreen |
| YWHAH | Fibroblasts-M29 | darkgreen |
| MYL9 | Fibroblasts-M8 | magenta |
| LARP1B | Fibroblasts-M4 | yellow |
| CCT2 | Fibroblasts-M1 | purple |
| TP53BP1 | Fibroblasts-M9 | pink |
| LYAR | Fibroblasts-M19 | orange |
| SCRN2 | Fibroblasts-M7 | cyan |
| ZFAND2B | Fibroblasts-M20 | red |
| TES | Fibroblasts-M8 | magenta |
| ZNF830 | Fibroblasts-M6 | turquoise |
| TRAPPC1 | Fibroblasts-M16 | lightcyan |
| CAPNS1 | Fibroblasts-M4 | yellow |
| VIL1 | Fibroblasts-M17 | darkgrey |
| SREK1 | Fibroblasts-M14 | tan |
| VILL | Fibroblasts-M9 | pink |
| MLLT10 | Fibroblasts-M20 | red |
| ARHGAP9 | Fibroblasts-M3 | lightyellow |
| MICAL1 | Fibroblasts-M23 | darkturquoise |
| CEBPB | Fibroblasts-M6 | turquoise |
| SLMAP | Fibroblasts-M21 | brown |
| RTL8A | Fibroblasts-M18 | white |
| MEAF6 | Fibroblasts-M9 | pink |
| FH | Fibroblasts-M21 | brown |
| TRDC | Fibroblasts-M25 | greenyellow |
| GAR1 | Fibroblasts-M5 | salmon |
| C1orf21 | Fibroblasts-M10 | blue |
| HDAC3 | Fibroblasts-M25 | greenyellow |
| FRMD4B | Fibroblasts-M24 | midnightblue |
| PSME2 | Fibroblasts-M2 | green |
| MRPL54 | Fibroblasts-M18 | white |
| EGLN1 | Fibroblasts-M25 | greenyellow |
| ZNF433-AS1 | Fibroblasts-M21 | brown |
| DYNC1H1 | Fibroblasts-M29 | darkgreen |
| TNIK | Fibroblasts-M7 | cyan |
| SELENBP1 | Fibroblasts-M20 | red |
| IL2RG | Fibroblasts-M21 | brown |
| BST2 | Fibroblasts-M2 | green |
| ZC3H12A | Fibroblasts-M6 | turquoise |
| APBB1IP | Fibroblasts-M10 | blue |
| ARSD | Fibroblasts-M7 | cyan |
| PLK2 | Fibroblasts-M6 | turquoise |
| GNAI2 | Fibroblasts-M18 | white |
| HBEGF | Fibroblasts-M6 | turquoise |
| PCLAF | Fibroblasts-M4 | yellow |
| RHOA | Fibroblasts-M8 | magenta |
| DCP1A | Fibroblasts-M13 | black |
| SCAMP1 | Fibroblasts-M8 | magenta |
| ERI1 | Fibroblasts-M4 | yellow |
| FKBP15 | Fibroblasts-M17 | darkgrey |
| CCDC18-AS1 | Fibroblasts-M10 | blue |
| MAPRE1 | Fibroblasts-M8 | magenta |
| C1D | Fibroblasts-M21 | brown |
| IFT43 | Fibroblasts-M16 | lightcyan |
| FURIN | Fibroblasts-M12 | steelblue |
| RRAS | Fibroblasts-M21 | brown |
| NDUFA11 | Fibroblasts-M10 | blue |
| LY86 | Fibroblasts-M4 | yellow |
| LITAF | Fibroblasts-M6 | turquoise |
| USO1 | Fibroblasts-M31 | darkred |
| FOXN3 | Fibroblasts-M9 | pink |
| PAK1 | Fibroblasts-M21 | brown |
| UFC1 | Fibroblasts-M25 | greenyellow |
| TRPM4 | Fibroblasts-M28 | skyblue |
| IL16 | Fibroblasts-M30 | saddlebrown |
| LDLRAD4 | Fibroblasts-M9 | pink |
| MYO5A | Fibroblasts-M19 | orange |
| GSE1 | Fibroblasts-M15 | grey60 |
| CLK1 | Fibroblasts-M6 | turquoise |
| RMND5A | Fibroblasts-M6 | turquoise |
| DOCK8 | Fibroblasts-M10 | blue |
| ZC3H6 | Fibroblasts-M7 | cyan |
| PRELID3B | Fibroblasts-M13 | black |
| NFXL1 | Fibroblasts-M30 | saddlebrown |
| VDAC2 | Fibroblasts-M13 | black |
| PLEKHA2 | Fibroblasts-M21 | brown |
| CDK7 | Fibroblasts-M26 | lightgreen |
| TMEM106B | Fibroblasts-M9 | pink |
| ACADVL | Fibroblasts-M4 | yellow |
| LCP1 | Fibroblasts-M24 | midnightblue |
| MIB1 | Fibroblasts-M9 | pink |
| PARP6 | Fibroblasts-M13 | black |
| ADIPOR2 | Fibroblasts-M6 | turquoise |
| UBE2B | Fibroblasts-M13 | black |
| RPN1 | Fibroblasts-M18 | white |
| GSKIP | Fibroblasts-M1 | purple |
| FAM114A1 | Fibroblasts-M14 | tan |
| TNS3 | Fibroblasts-M8 | magenta |
| TBC1D5 | Fibroblasts-M30 | saddlebrown |
| RFC1 | Fibroblasts-M27 | darkorange |
| SEC31A | Fibroblasts-M9 | pink |
| SERPING1 | Fibroblasts-M7 | cyan |
| HIPK2 | Fibroblasts-M1 | purple |
| CRTAP | Fibroblasts-M10 | blue |
| MAFB | Fibroblasts-M15 | grey60 |
| RBBP4 | Fibroblasts-M15 | grey60 |
| KLHL18 | Fibroblasts-M23 | darkturquoise |
| NAMPT | Fibroblasts-M6 | turquoise |
| GSTK1 | Fibroblasts-M2 | green |
| HYI | Fibroblasts-M20 | red |
| VDAC1 | Fibroblasts-M1 | purple |
| TUBA1C | Fibroblasts-M26 | lightgreen |
| MGA | Fibroblasts-M13 | black |
| RABAC1 | Fibroblasts-M19 | orange |
| ERGIC3 | Fibroblasts-M6 | turquoise |
| FAM173A | Fibroblasts-M2 | green |
| ZC3H7B | Fibroblasts-M10 | blue |
| ME2 | Fibroblasts-M3 | lightyellow |
| CBR1 | Fibroblasts-M16 | lightcyan |
| CHMP4B | Fibroblasts-M6 | turquoise |
| CD55 | Fibroblasts-M10 | blue |
| WDR82 | Fibroblasts-M9 | pink |
| TRIM27 | Fibroblasts-M1 | purple |
| THAP12 | Fibroblasts-M14 | tan |
| PIP4P2 | Fibroblasts-M5 | salmon |
| SIRT2 | Fibroblasts-M1 | purple |
| ERH | Fibroblasts-M18 | white |
| CLECL1 | Fibroblasts-M17 | darkgrey |
| COMT | Fibroblasts-M19 | orange |
| ATXN2 | Fibroblasts-M9 | pink |
| ZNHIT3 | Fibroblasts-M22 | paleturquoise |
| RNF220 | Fibroblasts-M25 | greenyellow |
| UBE2N | Fibroblasts-M18 | white |
| STARD4 | Fibroblasts-M22 | paleturquoise |
| MAP3K4 | Fibroblasts-M18 | white |
| SPAG9 | Fibroblasts-M27 | darkorange |
| DNAJC15 | Fibroblasts-M22 | paleturquoise |
| KIF21A | Fibroblasts-M10 | blue |
| XRN2 | Fibroblasts-M4 | yellow |
| PPP2R2D | Fibroblasts-M25 | greenyellow |
| LPP | Fibroblasts-M29 | darkgreen |
| NDUFS6 | Fibroblasts-M25 | greenyellow |
| PELP1 | Fibroblasts-M10 | blue |
| TEX264 | Fibroblasts-M30 | saddlebrown |
| SLC39A10 | Fibroblasts-M31 | darkred |
| PRKDC | Fibroblasts-M5 | salmon |
| MDK | Fibroblasts-M9 | pink |
| KIDINS220 | Fibroblasts-M27 | darkorange |
| DUSP16 | Fibroblasts-M21 | brown |
| SPTLC2 | Fibroblasts-M6 | turquoise |
| HIGD2A | Fibroblasts-M25 | greenyellow |
| NDRG1 | Fibroblasts-M6 | turquoise |
| TRAF3IP3 | Fibroblasts-M3 | lightyellow |
| SERINC3 | Fibroblasts-M12 | steelblue |
| SUMO3 | Fibroblasts-M7 | cyan |
| CCDC115 | Fibroblasts-M6 | turquoise |
| SIGIRR | Fibroblasts-M28 | skyblue |
| YEATS4 | Fibroblasts-M10 | blue |
| ARF4 | Fibroblasts-M26 | lightgreen |
| TSPYL2 | Fibroblasts-M6 | turquoise |
| TUBA1B | Fibroblasts-M8 | magenta |
| ATAD1 | Fibroblasts-M1 | purple |
| CSNK1G2 | Fibroblasts-M29 | darkgreen |
| DCTPP1 | Fibroblasts-M17 | darkgrey |
| TSPAN3 | Fibroblasts-M2 | green |
| RPL6P27 | Fibroblasts-M10 | blue |
| ENY2 | Fibroblasts-M29 | darkgreen |
| ZFAND1 | Fibroblasts-M25 | greenyellow |
| ITGA6 | Fibroblasts-M6 | turquoise |
| LAMP2 | Fibroblasts-M9 | pink |
| VEGFA | Fibroblasts-M6 | turquoise |
| AZI2 | Fibroblasts-M15 | grey60 |
| TERF2 | Fibroblasts-M17 | darkgrey |
| GARS | Fibroblasts-M6 | turquoise |
| AKAP13 | Fibroblasts-M14 | tan |
| PLEKHA1 | Fibroblasts-M17 | darkgrey |
| STX18 | Fibroblasts-M20 | red |
| SEC13 | Fibroblasts-M13 | black |
| SLC9A3R1 | Fibroblasts-M21 | brown |
| NDUFA13 | Fibroblasts-M10 | blue |
| TMEM45B | Fibroblasts-M6 | turquoise |
| SF3B1 | Fibroblasts-M9 | pink |
| GNL2 | Fibroblasts-M12 | steelblue |
| ATP5F1C | Fibroblasts-M1 | purple |
| PUS1 | Fibroblasts-M2 | green |
| RBBP7 | Fibroblasts-M2 | green |
| DYNLRB1 | Fibroblasts-M22 | paleturquoise |
| CEP57 | Fibroblasts-M4 | yellow |
| KAT7 | Fibroblasts-M21 | brown |
| LYRM7 | Fibroblasts-M17 | darkgrey |
| PRKACB | Fibroblasts-M20 | red |
| SYNE2 | Fibroblasts-M21 | brown |
| CYTH4 | Fibroblasts-M17 | darkgrey |
| CNTLN | Fibroblasts-M7 | cyan |
| NDUFAB1 | Fibroblasts-M18 | white |
| PNPT1 | Fibroblasts-M2 | green |
| BUD31 | Fibroblasts-M18 | white |
| TNF | Fibroblasts-M24 | midnightblue |
| GTF2H5 | Fibroblasts-M16 | lightcyan |
| DYNLL2 | Fibroblasts-M14 | tan |
| TUBA4A | Fibroblasts-M21 | brown |
| ARPC5 | Fibroblasts-M8 | magenta |
| LIMS1 | Fibroblasts-M25 | greenyellow |
| RASD1 | Fibroblasts-M1 | purple |
| CAVIN1 | Fibroblasts-M8 | magenta |
| SRA1 | Fibroblasts-M25 | greenyellow |
| MINK1 | Fibroblasts-M2 | green |
| EGLN3 | Fibroblasts-M6 | turquoise |
| MIGA1 | Fibroblasts-M6 | turquoise |
| PSMB6 | Fibroblasts-M8 | magenta |
| BHLHE40 | Fibroblasts-M6 | turquoise |
| MIB2 | Fibroblasts-M10 | blue |
| RAP2B | Fibroblasts-M9 | pink |
| ACTR6 | Fibroblasts-M28 | skyblue |
| KLHL5 | Fibroblasts-M6 | turquoise |
| OPA1 | Fibroblasts-M9 | pink |
| SF3B6 | Fibroblasts-M13 | black |
| HDLBP | Fibroblasts-M14 | tan |
| CYB561D2 | Fibroblasts-M5 | salmon |
| DHX9 | Fibroblasts-M6 | turquoise |
| P4HA1 | Fibroblasts-M6 | turquoise |
| AC024293.1 | Fibroblasts-M10 | blue |
| MCM3 | Fibroblasts-M4 | yellow |
| TRG-AS1 | Fibroblasts-M25 | greenyellow |
| ARID5A | Fibroblasts-M25 | greenyellow |
| GUSB | Fibroblasts-M10 | blue |
| BAIAP2 | Fibroblasts-M13 | black |
| IRAK4 | Fibroblasts-M18 | white |
| SMARCC1 | Fibroblasts-M20 | red |
| STOML2 | Fibroblasts-M1 | purple |
| CERS6 | Fibroblasts-M21 | brown |
| MRPS18C | Fibroblasts-M8 | magenta |
| H2AFJ | Fibroblasts-M25 | greenyellow |
| VCAN | Fibroblasts-M9 | pink |
| OCIAD1 | Fibroblasts-M2 | green |
| CRTAM | Fibroblasts-M25 | greenyellow |
| TMEM161B | Fibroblasts-M31 | darkred |
| PLP2 | Fibroblasts-M26 | lightgreen |
| PARVB | Fibroblasts-M25 | greenyellow |
| PHF10 | Fibroblasts-M6 | turquoise |
| TIPARP | Fibroblasts-M6 | turquoise |
| PCCB | Fibroblasts-M30 | saddlebrown |
| OCIAD2 | Fibroblasts-M4 | yellow |
| CCDC14 | Fibroblasts-M2 | green |
| CTDSP1 | Fibroblasts-M9 | pink |
| HEXA | Fibroblasts-M7 | cyan |
| ITGB1 | Fibroblasts-M8 | magenta |
| CCM2 | Fibroblasts-M25 | greenyellow |
| ACP5 | Fibroblasts-M6 | turquoise |
| CGAS | Fibroblasts-M24 | midnightblue |
| INO80C | Fibroblasts-M13 | black |
| CLTC | Fibroblasts-M15 | grey60 |
| VPS28 | Fibroblasts-M16 | lightcyan |
| N4BP2L2 | Fibroblasts-M9 | pink |
| FDFT1 | Fibroblasts-M29 | darkgreen |
| TP53INP1 | Fibroblasts-M5 | salmon |
| UBQLN1 | Fibroblasts-M26 | lightgreen |
| PLEKHA5 | Fibroblasts-M20 | red |
| ARHGAP21 | Fibroblasts-M20 | red |
| EDRF1 | Fibroblasts-M6 | turquoise |
| IDI1 | Fibroblasts-M6 | turquoise |
| TRMT11 | Fibroblasts-M23 | darkturquoise |
| EWSR1 | Fibroblasts-M6 | turquoise |
| PDCL | Fibroblasts-M6 | turquoise |
| S100A8 | Fibroblasts-M24 | midnightblue |
| NR3C1 | Fibroblasts-M13 | black |
| BCL2L15 | Fibroblasts-M28 | skyblue |
| MCM3AP | Fibroblasts-M6 | turquoise |
| TMEM176B | Fibroblasts-M20 | red |
| MLEC | Fibroblasts-M7 | cyan |
| DNPEP | Fibroblasts-M6 | turquoise |
| B4GALT1 | Fibroblasts-M9 | pink |
| C12orf75 | Fibroblasts-M4 | yellow |
| ADSS | Fibroblasts-M21 | brown |
| ASPH | Fibroblasts-M20 | red |
| TNFRSF1A | Fibroblasts-M7 | cyan |
| VPS39 | Fibroblasts-M21 | brown |
| MT-TP | Fibroblasts-M28 | skyblue |
| ARHGAP45 | Fibroblasts-M17 | darkgrey |
| TTTY15 | Fibroblasts-M28 | skyblue |
| MED6 | Fibroblasts-M2 | green |
| SEC61G | Fibroblasts-M26 | lightgreen |
| JAG1 | Fibroblasts-M8 | magenta |
| MYH9 | Fibroblasts-M8 | magenta |
| HMGN2 | Fibroblasts-M4 | yellow |
| DOCK2 | Fibroblasts-M4 | yellow |
| HAUS1 | Fibroblasts-M4 | yellow |
| CDC42SE2 | Fibroblasts-M5 | salmon |
| PYM1 | Fibroblasts-M2 | green |
| ASXL1 | Fibroblasts-M6 | turquoise |
| WDR61 | Fibroblasts-M28 | skyblue |
| TOP2B | Fibroblasts-M9 | pink |
| FCGBP | Fibroblasts-M25 | greenyellow |
| ABL2 | Fibroblasts-M13 | black |
| CDC42EP3 | Fibroblasts-M6 | turquoise |
| LMAN2 | Fibroblasts-M20 | red |
| MTERF4 | Fibroblasts-M19 | orange |
| TFB1M | Fibroblasts-M13 | black |
| RHOB | Fibroblasts-M6 | turquoise |
| MCMBP | Fibroblasts-M2 | green |
| CDCP1 | Fibroblasts-M6 | turquoise |
| UBE2C | Fibroblasts-M4 | yellow |
| EIF5A | Fibroblasts-M8 | magenta |
| TWISTNB | Fibroblasts-M14 | tan |
| RIOK3 | Fibroblasts-M13 | black |
| HIST1H2AG | Fibroblasts-M4 | yellow |
| EXOSC5 | Fibroblasts-M22 | paleturquoise |
| PTPRE | Fibroblasts-M1 | purple |
| ASH1L | Fibroblasts-M9 | pink |
| TADA3 | Fibroblasts-M26 | lightgreen |
| C12orf10 | Fibroblasts-M14 | tan |
| ELOVL1 | Fibroblasts-M31 | darkred |
| CEP290 | Fibroblasts-M2 | green |
| NHP2 | Fibroblasts-M18 | white |
| MORF4L1 | Fibroblasts-M14 | tan |
| LAS1L | Fibroblasts-M18 | white |
| CCDC91 | Fibroblasts-M6 | turquoise |
| PPP2R1A | Fibroblasts-M21 | brown |
| TMSB4XP4 | Fibroblasts-M2 | green |
| PIP4K2A | Fibroblasts-M24 | midnightblue |
| CKAP2 | Fibroblasts-M4 | yellow |
| IL6ST | Fibroblasts-M20 | red |
| PKM | Fibroblasts-M26 | lightgreen |
| ETHE1 | Fibroblasts-M1 | purple |
| PLEKHB2 | Fibroblasts-M1 | purple |
| GBA2 | Fibroblasts-M5 | salmon |
| FUT2 | Fibroblasts-M13 | black |
| SPINK4 | Fibroblasts-M10 | blue |
| PIP5K1B | Fibroblasts-M28 | skyblue |
| RSPRY1 | Fibroblasts-M9 | pink |
| GLUD1 | Fibroblasts-M21 | brown |
| IKBIP | Fibroblasts-M13 | black |
| CD24 | Fibroblasts-M6 | turquoise |
| CALD1 | Fibroblasts-M8 | magenta |
| MAP2K1 | Fibroblasts-M13 | black |
| RHEB | Fibroblasts-M13 | black |
| CDC42SE1 | Fibroblasts-M25 | greenyellow |
| SLK | Fibroblasts-M14 | tan |
| CENPBD1P1 | Fibroblasts-M21 | brown |
| HSPB11 | Fibroblasts-M19 | orange |
| MAFF | Fibroblasts-M6 | turquoise |
| TAF9 | Fibroblasts-M13 | black |
| CCT5 | Fibroblasts-M13 | black |
| ITPR2 | Fibroblasts-M13 | black |
| ALG8 | Fibroblasts-M20 | red |
| UTP6 | Fibroblasts-M13 | black |
| LY6E | Fibroblasts-M20 | red |
| PTPN18 | Fibroblasts-M6 | turquoise |
| HIST1H1D | Fibroblasts-M23 | darkturquoise |
| STOM | Fibroblasts-M21 | brown |
| CCNYL1 | Fibroblasts-M4 | yellow |
| TOMM40 | Fibroblasts-M26 | lightgreen |
| CCDC125 | Fibroblasts-M20 | red |
| ZNF281 | Fibroblasts-M14 | tan |
| C4orf48 | Fibroblasts-M19 | orange |
| ZNF106 | Fibroblasts-M20 | red |
| MGAT1 | Fibroblasts-M14 | tan |
| GAS5 | Fibroblasts-M13 | black |
| ICA1 | Fibroblasts-M10 | blue |
| MRPL11 | Fibroblasts-M20 | red |
| SRP54 | Fibroblasts-M9 | pink |
| ETFA | Fibroblasts-M2 | green |
| DDX39A | Fibroblasts-M13 | black |
| RNU6-6P | Fibroblasts-M28 | skyblue |
| GDF15 | Fibroblasts-M6 | turquoise |
| SLF1 | Fibroblasts-M21 | brown |
| HINT2 | Fibroblasts-M16 | lightcyan |
| FDPS | Fibroblasts-M13 | black |
| LSM6 | Fibroblasts-M4 | yellow |
| BPNT1 | Fibroblasts-M19 | orange |
| NFRKB | Fibroblasts-M6 | turquoise |
| TRIOBP | Fibroblasts-M16 | lightcyan |
| TSPO | Fibroblasts-M8 | magenta |
| MRRF | Fibroblasts-M2 | green |
| TMEM230 | Fibroblasts-M27 | darkorange |
| VGLL4 | Fibroblasts-M9 | pink |
| COA1 | Fibroblasts-M26 | lightgreen |
| ATXN7L3B | Fibroblasts-M7 | cyan |
| TMEM205 | Fibroblasts-M7 | cyan |
| NECAP1 | Fibroblasts-M17 | darkgrey |
| OFD1 | Fibroblasts-M27 | darkorange |
| MGME1 | Fibroblasts-M4 | yellow |
| TMEM167A | Fibroblasts-M14 | tan |
| MPZL1 | Fibroblasts-M9 | pink |
| PSMG4 | Fibroblasts-M2 | green |
| SPTSSA | Fibroblasts-M6 | turquoise |
| H2AFY | Fibroblasts-M31 | darkred |
| ANKLE2 | Fibroblasts-M12 | steelblue |
| FAM136A | Fibroblasts-M23 | darkturquoise |
| MAP7D1 | Fibroblasts-M16 | lightcyan |
| ANKMY2 | Fibroblasts-M20 | red |
| ATP2A2 | Fibroblasts-M6 | turquoise |
| MAZ | Fibroblasts-M4 | yellow |
| OSTC | Fibroblasts-M14 | tan |
| DPM2 | Fibroblasts-M26 | lightgreen |
| EPS8 | Fibroblasts-M21 | brown |
| RRNAD1 | Fibroblasts-M21 | brown |
| ZNF271P | Fibroblasts-M1 | purple |
| LMBRD1 | Fibroblasts-M21 | brown |
| STAT3 | Fibroblasts-M20 | red |
| TMEM106C | Fibroblasts-M4 | yellow |
| GBP3 | Fibroblasts-M2 | green |
| VCL | Fibroblasts-M8 | magenta |
| MINDY2 | Fibroblasts-M14 | tan |
| ZNF32 | Fibroblasts-M20 | red |
| MRPL52 | Fibroblasts-M1 | purple |
| ARL16 | Fibroblasts-M2 | green |
| HIST1H2AE | Fibroblasts-M6 | turquoise |
| SERINC5 | Fibroblasts-M9 | pink |
| DCTN2 | Fibroblasts-M25 | greenyellow |
| C19orf66 | Fibroblasts-M20 | red |
| MPST | Fibroblasts-M6 | turquoise |
| NUP153 | Fibroblasts-M9 | pink |
| ASRGL1 | Fibroblasts-M4 | yellow |
| NET1 | Fibroblasts-M21 | brown |
| HPRT1 | Fibroblasts-M1 | purple |
| OPN3 | Fibroblasts-M14 | tan |
| TPD52L2 | Fibroblasts-M12 | steelblue |
| CENPU | Fibroblasts-M4 | yellow |
| UGDH | Fibroblasts-M14 | tan |
| MBD1 | Fibroblasts-M9 | pink |
| NDUFS2 | Fibroblasts-M21 | brown |
| DARS | Fibroblasts-M1 | purple |
| TRAPPC2B | Fibroblasts-M26 | lightgreen |
| CHMP2B | Fibroblasts-M7 | cyan |
| DCAF8 | Fibroblasts-M6 | turquoise |
| TMX3 | Fibroblasts-M2 | green |
| TRIR | Fibroblasts-M10 | blue |
| ETS2 | Fibroblasts-M6 | turquoise |
| SPTBN1 | Fibroblasts-M2 | green |
| EPRS | Fibroblasts-M14 | tan |
| AC026979.2 | Fibroblasts-M24 | midnightblue |
| PRKD2 | Fibroblasts-M3 | lightyellow |
| SMC4 | Fibroblasts-M4 | yellow |
| POP5 | Fibroblasts-M4 | yellow |
| ABCF1 | Fibroblasts-M7 | cyan |
| TPGS2 | Fibroblasts-M30 | saddlebrown |
| ERLIN2 | Fibroblasts-M14 | tan |
| ZNF354A | Fibroblasts-M25 | greenyellow |
| KHDRBS1 | Fibroblasts-M13 | black |
| ERCC1 | Fibroblasts-M14 | tan |
| APOO | Fibroblasts-M4 | yellow |
| C19orf12 | Fibroblasts-M2 | green |
| GBP1 | Fibroblasts-M2 | green |
| ANKRD37 | Fibroblasts-M13 | black |
| CD83 | Fibroblasts-M6 | turquoise |
| AC007388.1 | Fibroblasts-M6 | turquoise |
| YBX3 | Fibroblasts-M20 | red |
| DPP3 | Fibroblasts-M8 | magenta |
| TOR1AIP2 | Fibroblasts-M23 | darkturquoise |
| CCP110 | Fibroblasts-M20 | red |
| HIP1R | Fibroblasts-M19 | orange |
| STARD3NL | Fibroblasts-M2 | green |
| COA4 | Fibroblasts-M4 | yellow |
| NUP93 | Fibroblasts-M22 | paleturquoise |
| ZNF720 | Fibroblasts-M9 | pink |
| COL14A1 | Fibroblasts-M7 | cyan |
| ICAM3 | Fibroblasts-M6 | turquoise |
| KIAA0930 | Fibroblasts-M20 | red |
| HIST1H2AC | Fibroblasts-M6 | turquoise |
| XAF1 | Fibroblasts-M5 | salmon |
| DDX55 | Fibroblasts-M2 | green |
| PTS | Fibroblasts-M6 | turquoise |
| ERN1 | Fibroblasts-M6 | turquoise |
| GTF3C2 | Fibroblasts-M14 | tan |
| AL050331.1 | Fibroblasts-M19 | orange |
| ATP5MD | Fibroblasts-M16 | lightcyan |
| ETS1 | Fibroblasts-M8 | magenta |
| DANCR | Fibroblasts-M20 | red |
| RNPEP | Fibroblasts-M28 | skyblue |
| PUF60 | Fibroblasts-M25 | greenyellow |
| BBX | Fibroblasts-M2 | green |
| H2AFV | Fibroblasts-M4 | yellow |
| PRKAB1 | Fibroblasts-M2 | green |
| BID | Fibroblasts-M17 | darkgrey |
| POLR3GL | Fibroblasts-M16 | lightcyan |
| NIPSNAP2 | Fibroblasts-M9 | pink |
| SOD3 | Fibroblasts-M21 | brown |
| MIOS | Fibroblasts-M2 | green |
| UBE2E3 | Fibroblasts-M17 | darkgrey |
| CHCHD7 | Fibroblasts-M20 | red |
| ALDH2 | Fibroblasts-M10 | blue |
| PCBD1 | Fibroblasts-M28 | skyblue |
| CTDSPL2 | Fibroblasts-M15 | grey60 |
| TMEM11 | Fibroblasts-M25 | greenyellow |
| CAVIN3 | Fibroblasts-M27 | darkorange |
| EFL1 | Fibroblasts-M26 | lightgreen |
| COA5 | Fibroblasts-M16 | lightcyan |
| FOXN2 | Fibroblasts-M8 | magenta |
| MXI1 | Fibroblasts-M13 | black |
| TCEAL8 | Fibroblasts-M4 | yellow |
| TMEM138 | Fibroblasts-M2 | green |
| FBXL15 | Fibroblasts-M10 | blue |
| SLC25A24 | Fibroblasts-M8 | magenta |
| C11orf96 | Fibroblasts-M6 | turquoise |
| GSN | Fibroblasts-M10 | blue |
| IMPA2 | Fibroblasts-M28 | skyblue |
| ZC3HAV1 | Fibroblasts-M13 | black |
| CSRP1 | Fibroblasts-M8 | magenta |
| IFT27 | Fibroblasts-M6 | turquoise |
| HSD17B11 | Fibroblasts-M30 | saddlebrown |
| INO80D | Fibroblasts-M5 | salmon |
| FABP5 | Fibroblasts-M2 | green |
| RGS19 | Fibroblasts-M4 | yellow |
| EMP1 | Fibroblasts-M6 | turquoise |
| MGST2 | Fibroblasts-M23 | darkturquoise |
| RNU2-63P | Fibroblasts-M3 | lightyellow |
| NREP | Fibroblasts-M31 | darkred |
| DDX49 | Fibroblasts-M13 | black |
| ID1 | Fibroblasts-M2 | green |
| C1orf122 | Fibroblasts-M16 | lightcyan |
| G0S2 | Fibroblasts-M13 | black |
| CDKN3 | Fibroblasts-M4 | yellow |
| DCTN3 | Fibroblasts-M18 | white |
| ZBTB38 | Fibroblasts-M14 | tan |
| SLC26A2 | Fibroblasts-M15 | grey60 |
| CD8A | Fibroblasts-M10 | blue |
| DDX52 | Fibroblasts-M1 | purple |
| C10orf99 | Fibroblasts-M30 | saddlebrown |
| WAS | Fibroblasts-M24 | midnightblue |
| P2RY10 | Fibroblasts-M24 | midnightblue |
| RAP1A | Fibroblasts-M1 | purple |
| MRPL32 | Fibroblasts-M5 | salmon |
| SPAST | Fibroblasts-M15 | grey60 |
| SPTAN1 | Fibroblasts-M19 | orange |
| ST6GAL1 | Fibroblasts-M3 | lightyellow |
| PHACTR2 | Fibroblasts-M6 | turquoise |
| C1orf43 | Fibroblasts-M25 | greenyellow |
| SLC35E3 | Fibroblasts-M20 | red |
| CIR1 | Fibroblasts-M16 | lightcyan |
| CHD8 | Fibroblasts-M12 | steelblue |
| EEF1A1P5 | Fibroblasts-M30 | saddlebrown |
| STK16 | Fibroblasts-M8 | magenta |
| RNF114 | Fibroblasts-M1 | purple |
| DENND2D | Fibroblasts-M30 | saddlebrown |
| CASC4 | Fibroblasts-M9 | pink |
| LSM4 | Fibroblasts-M13 | black |
| SLC1A5 | Fibroblasts-M6 | turquoise |
| SNRNP35 | Fibroblasts-M6 | turquoise |
| FAM162A | Fibroblasts-M13 | black |
| AC131235.1 | Fibroblasts-M10 | blue |
| ALKBH7 | Fibroblasts-M28 | skyblue |
| ERGIC2 | Fibroblasts-M20 | red |
| PKN1 | Fibroblasts-M21 | brown |
| PLD3 | Fibroblasts-M7 | cyan |
| POC1B | Fibroblasts-M2 | green |
| DPYSL2 | Fibroblasts-M10 | blue |
| CLTB | Fibroblasts-M16 | lightcyan |
| CCDC107 | Fibroblasts-M25 | greenyellow |
| NKIRAS2 | Fibroblasts-M6 | turquoise |
| CDC37 | Fibroblasts-M23 | darkturquoise |
| CMTM7 | Fibroblasts-M7 | cyan |
| ZYX | Fibroblasts-M8 | magenta |
| ANXA7 | Fibroblasts-M27 | darkorange |
| TLE3 | Fibroblasts-M23 | darkturquoise |
| CLK4 | Fibroblasts-M15 | grey60 |
| GFM1 | Fibroblasts-M12 | steelblue |
| SP2 | Fibroblasts-M7 | cyan |
| MYEF2 | Fibroblasts-M10 | blue |
| STAMBPL1 | Fibroblasts-M2 | green |
| PIAS1 | Fibroblasts-M2 | green |
| GLG1 | Fibroblasts-M9 | pink |
| OAS1 | Fibroblasts-M2 | green |
| DNAJA3 | Fibroblasts-M2 | green |
| EPHA2 | Fibroblasts-M6 | turquoise |
| CENPE | Fibroblasts-M4 | yellow |
| LACTB | Fibroblasts-M1 | purple |
| UBE2L6 | Fibroblasts-M2 | green |
| HIST1H2BG | Fibroblasts-M2 | green |
| RAB10 | Fibroblasts-M21 | brown |
| CCS | Fibroblasts-M10 | blue |
| MLKL | Fibroblasts-M1 | purple |
| ATP9B | Fibroblasts-M14 | tan |
| PIBF1 | Fibroblasts-M5 | salmon |
| OSER1 | Fibroblasts-M6 | turquoise |
| RRP8 | Fibroblasts-M21 | brown |
| MARCKSL1 | Fibroblasts-M21 | brown |
| MSMO1 | Fibroblasts-M6 | turquoise |
| CD37 | Fibroblasts-M10 | blue |
| XRCC1 | Fibroblasts-M10 | blue |
| LACC1 | Fibroblasts-M6 | turquoise |
| CNN3 | Fibroblasts-M23 | darkturquoise |
| CASP1 | Fibroblasts-M6 | turquoise |
| MAP7D3 | Fibroblasts-M21 | brown |
| GLCE | Fibroblasts-M7 | cyan |
| MPZL2 | Fibroblasts-M17 | darkgrey |
| PLCB2 | Fibroblasts-M24 | midnightblue |
| HMGA1 | Fibroblasts-M13 | black |
| MAP3K13 | Fibroblasts-M21 | brown |
| CORO1A | Fibroblasts-M10 | blue |
| RBFOX2 | Fibroblasts-M31 | darkred |
| CTSH | Fibroblasts-M20 | red |
| PNRC2 | Fibroblasts-M13 | black |
| USP33 | Fibroblasts-M1 | purple |
| PHF19 | Fibroblasts-M26 | lightgreen |
| RILPL2 | Fibroblasts-M10 | blue |
| PIGT | Fibroblasts-M10 | blue |
| IFI35 | Fibroblasts-M2 | green |
| NFKBID | Fibroblasts-M21 | brown |
| CYFIP2 | Fibroblasts-M25 | greenyellow |
| FBXO34 | Fibroblasts-M31 | darkred |
| CENPW | Fibroblasts-M4 | yellow |
| AKAP10 | Fibroblasts-M19 | orange |
| FGD4 | Fibroblasts-M2 | green |
| ITGB3BP | Fibroblasts-M4 | yellow |
| CXCL16 | Fibroblasts-M20 | red |
| GPCPD1 | Fibroblasts-M8 | magenta |
| SETDB2 | Fibroblasts-M9 | pink |
| MLST8 | Fibroblasts-M4 | yellow |
| MTAP | Fibroblasts-M29 | darkgreen |
| PSENEN | Fibroblasts-M26 | lightgreen |
| PABPC4 | Fibroblasts-M26 | lightgreen |
| CSKMT | Fibroblasts-M28 | skyblue |
| SLC25A25 | Fibroblasts-M6 | turquoise |
| NRIP1 | Fibroblasts-M9 | pink |
| SIAE | Fibroblasts-M21 | brown |
| NFKB2 | Fibroblasts-M17 | darkgrey |
| DAB2 | Fibroblasts-M7 | cyan |
| DIP2A | Fibroblasts-M4 | yellow |
| SNHG9 | Fibroblasts-M10 | blue |
| PPP1CB | Fibroblasts-M18 | white |
| APH1B | Fibroblasts-M6 | turquoise |
| FXN | Fibroblasts-M28 | skyblue |
| SNHG11 | Fibroblasts-M10 | blue |
| RPRD1A | Fibroblasts-M18 | white |
| BANF1 | Fibroblasts-M4 | yellow |
| ZNF430 | Fibroblasts-M20 | red |
| COL1A2 | Fibroblasts-M9 | pink |
| ARID3A | Fibroblasts-M6 | turquoise |
| TGS1 | Fibroblasts-M4 | yellow |
| COPG1 | Fibroblasts-M1 | purple |
| ITPR3 | Fibroblasts-M20 | red |
| CEPT1 | Fibroblasts-M20 | red |
| C11orf24 | Fibroblasts-M27 | darkorange |
| KAT2B | Fibroblasts-M20 | red |
| TDG | Fibroblasts-M14 | tan |
| CHPT1 | Fibroblasts-M6 | turquoise |
| ASS1 | Fibroblasts-M13 | black |
| GALNT5 | Fibroblasts-M9 | pink |
| RABGAP1 | Fibroblasts-M21 | brown |
| MISP | Fibroblasts-M25 | greenyellow |
| STX17 | Fibroblasts-M21 | brown |
| PDIA3P1 | Fibroblasts-M10 | blue |
| TNIP1 | Fibroblasts-M12 | steelblue |
| TARS2 | Fibroblasts-M30 | saddlebrown |
| CD2BP2 | Fibroblasts-M21 | brown |
| BIRC5 | Fibroblasts-M4 | yellow |
| AKAP5 | Fibroblasts-M30 | saddlebrown |
| NXF1 | Fibroblasts-M23 | darkturquoise |
| CRIPT | Fibroblasts-M12 | steelblue |
| MAP2K3 | Fibroblasts-M12 | steelblue |
| ELK3 | Fibroblasts-M15 | grey60 |
| PLK3 | Fibroblasts-M6 | turquoise |
| TIMM23 | Fibroblasts-M4 | yellow |
| MXD1 | Fibroblasts-M1 | purple |
| UTY | Fibroblasts-M28 | skyblue |
| MBIP | Fibroblasts-M20 | red |
| THEMIS | Fibroblasts-M24 | midnightblue |
| SNORD3A | Fibroblasts-M20 | red |
| C19orf48 | Fibroblasts-M4 | yellow |
| DIS3 | Fibroblasts-M12 | steelblue |
| PLEKHN1 | Fibroblasts-M3 | lightyellow |
| SLC30A7 | Fibroblasts-M14 | tan |
| YLPM1 | Fibroblasts-M5 | salmon |
| AP2S1 | Fibroblasts-M8 | magenta |
| SREBF2 | Fibroblasts-M23 | darkturquoise |
| CLIC4 | Fibroblasts-M29 | darkgreen |
| NRP2 | Fibroblasts-M31 | darkred |
| CD68 | Fibroblasts-M10 | blue |
| ATIC | Fibroblasts-M18 | white |
| ANXA11 | Fibroblasts-M9 | pink |
| ZEB1 | Fibroblasts-M6 | turquoise |
| MYLK | Fibroblasts-M8 | magenta |
| TMEM173 | Fibroblasts-M2 | green |
| CRYBG3 | Fibroblasts-M2 | green |
| RECQL | Fibroblasts-M8 | magenta |
| RPL15P3 | Fibroblasts-M31 | darkred |
| MRPS34 | Fibroblasts-M16 | lightcyan |
| CUTC | Fibroblasts-M10 | blue |
| SNRPC | Fibroblasts-M8 | magenta |
| SLC39A14 | Fibroblasts-M6 | turquoise |
| SYPL1 | Fibroblasts-M4 | yellow |
| RANGAP1 | Fibroblasts-M17 | darkgrey |
| PTPRF | Fibroblasts-M7 | cyan |
| TRAT1 | Fibroblasts-M21 | brown |
| ENSA | Fibroblasts-M4 | yellow |
| PRKX | Fibroblasts-M2 | green |
| TMED2 | Fibroblasts-M29 | darkgreen |
| NFIX | Fibroblasts-M10 | blue |
| AAGAB | Fibroblasts-M1 | purple |
| BTAF1 | Fibroblasts-M1 | purple |
| CCDC174 | Fibroblasts-M7 | cyan |
| PICALM | Fibroblasts-M31 | darkred |
| LACTB2 | Fibroblasts-M25 | greenyellow |
| EPN2 | Fibroblasts-M23 | darkturquoise |
| FAM91A1 | Fibroblasts-M31 | darkred |
| RAB13 | Fibroblasts-M14 | tan |
| AC113935.1 | Fibroblasts-M10 | blue |
| FAM46C | Fibroblasts-M3 | lightyellow |
| METAP2 | Fibroblasts-M17 | darkgrey |
| BIRC6 | Fibroblasts-M23 | darkturquoise |
| ARMCX3 | Fibroblasts-M14 | tan |
| ARHGAP26 | Fibroblasts-M21 | brown |
| ESYT2 | Fibroblasts-M14 | tan |
| RPL24P8 | Fibroblasts-M10 | blue |
| MAGOH | Fibroblasts-M4 | yellow |
| ZNF148 | Fibroblasts-M6 | turquoise |
| DAPK3 | Fibroblasts-M29 | darkgreen |
| SLCO3A1 | Fibroblasts-M21 | brown |
| ZNF609 | Fibroblasts-M5 | salmon |
| LAIR1 | Fibroblasts-M24 | midnightblue |
| FUT4 | Fibroblasts-M31 | darkred |
| POLR2E | Fibroblasts-M4 | yellow |
| ZNF33A | Fibroblasts-M30 | saddlebrown |
| MIS12 | Fibroblasts-M13 | black |
| UBE2T | Fibroblasts-M4 | yellow |
| SAMD9L | Fibroblasts-M5 | salmon |
| ERAL1 | Fibroblasts-M13 | black |
| TFPI | Fibroblasts-M1 | purple |
| RPS10 | Fibroblasts-M10 | blue |
| CENPK | Fibroblasts-M4 | yellow |
| MTO1 | Fibroblasts-M20 | red |
| MRPS18A | Fibroblasts-M6 | turquoise |
| SLC25A46 | Fibroblasts-M23 | darkturquoise |
| ANKRD13A | Fibroblasts-M6 | turquoise |
| TBCD | Fibroblasts-M1 | purple |
| RPL21P28 | Fibroblasts-M10 | blue |
| MAPK9 | Fibroblasts-M20 | red |
| DHX15 | Fibroblasts-M12 | steelblue |
| AK2 | Fibroblasts-M26 | lightgreen |
| HLA-F | Fibroblasts-M2 | green |
| CPD | Fibroblasts-M2 | green |
| PPP4C | Fibroblasts-M13 | black |
| USP3 | Fibroblasts-M1 | purple |
| SH3YL1 | Fibroblasts-M9 | pink |
| C12orf4 | Fibroblasts-M2 | green |
| HIST1H2BD | Fibroblasts-M6 | turquoise |
| IFI44 | Fibroblasts-M5 | salmon |
| MED25 | Fibroblasts-M4 | yellow |
| SDC4 | Fibroblasts-M6 | turquoise |
| COA7 | Fibroblasts-M6 | turquoise |
| SPRYD3 | Fibroblasts-M14 | tan |
| PPID | Fibroblasts-M5 | salmon |
| MRPS6 | Fibroblasts-M12 | steelblue |
| SLC16A1 | Fibroblasts-M13 | black |
| AL021453.1 | Fibroblasts-M17 | darkgrey |
| PPA1 | Fibroblasts-M2 | green |
| TNFRSF17 | Fibroblasts-M3 | lightyellow |
| CLMN | Fibroblasts-M21 | brown |
| LCK | Fibroblasts-M10 | blue |
| MICU1 | Fibroblasts-M7 | cyan |
| MMGT1 | Fibroblasts-M27 | darkorange |
| NDUFA9 | Fibroblasts-M1 | purple |
| FUNDC1 | Fibroblasts-M16 | lightcyan |
| EVA1B | Fibroblasts-M22 | paleturquoise |
| CKS1B | Fibroblasts-M16 | lightcyan |
| DLGAP1-AS1 | Fibroblasts-M26 | lightgreen |
| NUP160 | Fibroblasts-M14 | tan |
| PPP1R12A | Fibroblasts-M8 | magenta |
| UBE3C | Fibroblasts-M24 | midnightblue |
| SHC1 | Fibroblasts-M2 | green |
| TAZ | Fibroblasts-M13 | black |
| CHCHD5 | Fibroblasts-M12 | steelblue |
| MSH3 | Fibroblasts-M20 | red |
| NR2F2 | Fibroblasts-M21 | brown |
| LUM | Fibroblasts-M20 | red |
| REEP3 | Fibroblasts-M8 | magenta |
| AKR1B1 | Fibroblasts-M6 | turquoise |
| TGFBR2 | Fibroblasts-M20 | red |
| ANAPC10 | Fibroblasts-M16 | lightcyan |
| PIK3AP1 | Fibroblasts-M24 | midnightblue |
| CCNA2 | Fibroblasts-M4 | yellow |
| CLUH | Fibroblasts-M20 | red |
| EXOSC6 | Fibroblasts-M13 | black |
| WEE1 | Fibroblasts-M20 | red |
| EPN1 | Fibroblasts-M21 | brown |
| SLC35E1 | Fibroblasts-M12 | steelblue |
| FCRL5 | Fibroblasts-M21 | brown |
| SRPK2 | Fibroblasts-M20 | red |
| RBMS2 | Fibroblasts-M30 | saddlebrown |
| EXOC2 | Fibroblasts-M27 | darkorange |
| PSMD1 | Fibroblasts-M27 | darkorange |
| ENC1 | Fibroblasts-M1 | purple |
| SRP68 | Fibroblasts-M20 | red |
| UBE2K | Fibroblasts-M31 | darkred |
| SMARCA2 | Fibroblasts-M7 | cyan |
| CYP3A5 | Fibroblasts-M6 | turquoise |
| BAG3 | Fibroblasts-M6 | turquoise |
| SUN2 | Fibroblasts-M3 | lightyellow |
| ANXA4 | Fibroblasts-M7 | cyan |
| MRM2 | Fibroblasts-M5 | salmon |
| ABHD18 | Fibroblasts-M17 | darkgrey |
| PON2 | Fibroblasts-M23 | darkturquoise |
| CHD3 | Fibroblasts-M9 | pink |
| HMGB1P8 | Fibroblasts-M10 | blue |
| C22orf39 | Fibroblasts-M16 | lightcyan |
| PRRC1 | Fibroblasts-M14 | tan |
| DDX60L | Fibroblasts-M1 | purple |
| KLF10 | Fibroblasts-M6 | turquoise |
| NR1H2 | Fibroblasts-M15 | grey60 |
| HAUS3 | Fibroblasts-M9 | pink |
| SLC2A6 | Fibroblasts-M21 | brown |
| LAMB1 | Fibroblasts-M5 | salmon |
| NTPCR | Fibroblasts-M6 | turquoise |
| P2RX4 | Fibroblasts-M23 | darkturquoise |
| SERPINA1 | Fibroblasts-M10 | blue |
| UBE2Q2 | Fibroblasts-M6 | turquoise |
| IMMP1L | Fibroblasts-M3 | lightyellow |
| RAPGEF1 | Fibroblasts-M31 | darkred |
| TSPAN15 | Fibroblasts-M25 | greenyellow |
| ACSL5 | Fibroblasts-M5 | salmon |
| KRIT1 | Fibroblasts-M31 | darkred |
| CWF19L1 | Fibroblasts-M13 | black |
| MAP4K4 | Fibroblasts-M9 | pink |
| TRAP1 | Fibroblasts-M10 | blue |
| ZFAND2A | Fibroblasts-M6 | turquoise |
| LSM2 | Fibroblasts-M4 | yellow |
| UFL1 | Fibroblasts-M29 | darkgreen |
| MKRN2 | Fibroblasts-M9 | pink |
| KCTD12 | Fibroblasts-M9 | pink |
| DGKH | Fibroblasts-M21 | brown |
| C18orf21 | Fibroblasts-M13 | black |
| 11-Sep | Fibroblasts-M20 | red |
| GALNT2 | Fibroblasts-M14 | tan |
| OS9 | Fibroblasts-M9 | pink |
| SPTLC1 | Fibroblasts-M14 | tan |
| RBMXL1 | Fibroblasts-M2 | green |
| AC021078.1 | Fibroblasts-M20 | red |
| KIAA1217 | Fibroblasts-M9 | pink |
| ACLY | Fibroblasts-M2 | green |
| HMGCS1 | Fibroblasts-M6 | turquoise |
| SLC3A2 | Fibroblasts-M6 | turquoise |
| EIF4EBP2 | Fibroblasts-M29 | darkgreen |
| PPP6C | Fibroblasts-M27 | darkorange |
| SMS | Fibroblasts-M13 | black |
| TRIM28 | Fibroblasts-M1 | purple |
| KRCC1 | Fibroblasts-M5 | salmon |
| CDC20 | Fibroblasts-M5 | salmon |
| SLC25A13 | Fibroblasts-M21 | brown |
| QSER1 | Fibroblasts-M14 | tan |
| WDR1 | Fibroblasts-M8 | magenta |
| MCPH1 | Fibroblasts-M30 | saddlebrown |
| AIFM1 | Fibroblasts-M25 | greenyellow |
| PIGX | Fibroblasts-M1 | purple |
| CPNE1 | Fibroblasts-M6 | turquoise |
| MTIF3 | Fibroblasts-M20 | red |
| USP47 | Fibroblasts-M2 | green |
| ADAMDEC1 | Fibroblasts-M20 | red |
| NSMCE3 | Fibroblasts-M3 | lightyellow |
| RARRES2 | Fibroblasts-M20 | red |
| CCDC88C | Fibroblasts-M6 | turquoise |
| PDCD4 | Fibroblasts-M20 | red |
| SGMS1 | Fibroblasts-M1 | purple |
| MMP2 | Fibroblasts-M7 | cyan |
| MVD | Fibroblasts-M28 | skyblue |
| PPP1R7 | Fibroblasts-M1 | purple |
| FAM118A | Fibroblasts-M10 | blue |
| RAD51AP1 | Fibroblasts-M4 | yellow |
| EDN1 | Fibroblasts-M22 | paleturquoise |
| PPIE | Fibroblasts-M14 | tan |
| UQCRFS1 | Fibroblasts-M16 | lightcyan |
| HELZ | Fibroblasts-M7 | cyan |
| PAPSS2 | Fibroblasts-M5 | salmon |
| KIAA0141 | Fibroblasts-M31 | darkred |
| DPP9 | Fibroblasts-M20 | red |
| SUSD3 | Fibroblasts-M3 | lightyellow |
| MAN2A1 | Fibroblasts-M2 | green |
| CCNL2 | Fibroblasts-M23 | darkturquoise |
| HEPH | Fibroblasts-M22 | paleturquoise |
| SNRPD1 | Fibroblasts-M1 | purple |
| RBFA | Fibroblasts-M21 | brown |
| ME1 | Fibroblasts-M6 | turquoise |
| QRICH1 | Fibroblasts-M25 | greenyellow |
| RAB31 | Fibroblasts-M14 | tan |
| TBC1D14 | Fibroblasts-M28 | skyblue |
| BMPR2 | Fibroblasts-M9 | pink |
| AL450405.1 | Fibroblasts-M10 | blue |
| GUCD1 | Fibroblasts-M1 | purple |
| REXO2 | Fibroblasts-M16 | lightcyan |
| WASL | Fibroblasts-M14 | tan |
| SAMM50 | Fibroblasts-M31 | darkred |
| CXCL8 | Fibroblasts-M13 | black |
| RAB30-AS1 | Fibroblasts-M10 | blue |
| IER5L | Fibroblasts-M20 | red |
| THBS1 | Fibroblasts-M20 | red |
| CTBP2 | Fibroblasts-M29 | darkgreen |
| ZC3H7A | Fibroblasts-M20 | red |
| P4HTM | Fibroblasts-M20 | red |
| SUCLA2 | Fibroblasts-M22 | paleturquoise |
| KIF3A | Fibroblasts-M27 | darkorange |
| MYNN | Fibroblasts-M2 | green |
| NOTCH1 | Fibroblasts-M21 | brown |
| SLC25A39 | Fibroblasts-M1 | purple |
| TPRA1 | Fibroblasts-M31 | darkred |
| ABHD17A | Fibroblasts-M10 | blue |
| PTPN6 | Fibroblasts-M21 | brown |
| RF00003 | Fibroblasts-M10 | blue |
| ISY1 | Fibroblasts-M21 | brown |
| DLG3 | Fibroblasts-M21 | brown |
| EIF3D | Fibroblasts-M13 | black |
| BCL2L11 | Fibroblasts-M15 | grey60 |
| SSBP4 | Fibroblasts-M1 | purple |
| 9-Mar | Fibroblasts-M2 | green |
| RPRD2 | Fibroblasts-M9 | pink |
| WBP11 | Fibroblasts-M5 | salmon |
| DHX32 | Fibroblasts-M1 | purple |
| MPC1 | Fibroblasts-M25 | greenyellow |
| DOCK1 | Fibroblasts-M20 | red |
| SEC14L1 | Fibroblasts-M1 | purple |
| OSTF1 | Fibroblasts-M21 | brown |
| SH3BP1 | Fibroblasts-M1 | purple |
| SUPT20H | Fibroblasts-M25 | greenyellow |
| CCDC167 | Fibroblasts-M18 | white |
| CCDC66 | Fibroblasts-M15 | grey60 |
| PRNP | Fibroblasts-M20 | red |
| GNS | Fibroblasts-M20 | red |
| NNMT | Fibroblasts-M18 | white |
| SLPI | Fibroblasts-M10 | blue |
| TALDO1 | Fibroblasts-M25 | greenyellow |
| CHD7 | Fibroblasts-M17 | darkgrey |
| GLRX2 | Fibroblasts-M19 | orange |
| SUN1 | Fibroblasts-M29 | darkgreen |
| STAT5B | Fibroblasts-M21 | brown |
| KIAA0232 | Fibroblasts-M17 | darkgrey |
| METTL3 | Fibroblasts-M2 | green |
| ZDHHC20 | Fibroblasts-M14 | tan |
| FBXO32 | Fibroblasts-M12 | steelblue |
| DUSP4 | Fibroblasts-M25 | greenyellow |
| BCKDHB | Fibroblasts-M10 | blue |
| FAM89B | Fibroblasts-M14 | tan |
| IDH2 | Fibroblasts-M8 | magenta |
| PTMAP5 | Fibroblasts-M16 | lightcyan |
| PDZD11 | Fibroblasts-M29 | darkgreen |
| SDHAF1 | Fibroblasts-M10 | blue |
| ELP2 | Fibroblasts-M12 | steelblue |
| PAFAH1B3 | Fibroblasts-M4 | yellow |
| CXXC1 | Fibroblasts-M6 | turquoise |
| LINC00467 | Fibroblasts-M10 | blue |
| MBNL3 | Fibroblasts-M4 | yellow |
| FAR1 | Fibroblasts-M5 | salmon |
| LINC00511 | Fibroblasts-M13 | black |
| AP2A2 | Fibroblasts-M17 | darkgrey |
| NEDD4L | Fibroblasts-M13 | black |
| NT5DC1 | Fibroblasts-M9 | pink |
| GOLGA8A | Fibroblasts-M6 | turquoise |
| HIST2H2AC | Fibroblasts-M4 | yellow |
| RALB | Fibroblasts-M6 | turquoise |
| ADAM28 | Fibroblasts-M24 | midnightblue |
| SLC38A6 | Fibroblasts-M6 | turquoise |
| IL4I1 | Fibroblasts-M9 | pink |
| MPHOSPH6 | Fibroblasts-M6 | turquoise |
| EEA1 | Fibroblasts-M7 | cyan |
| GCNT3 | Fibroblasts-M1 | purple |
| EMILIN2 | Fibroblasts-M25 | greenyellow |
| RND3 | Fibroblasts-M6 | turquoise |
| TRGC1 | Fibroblasts-M24 | midnightblue |
| SLC25A1 | Fibroblasts-M25 | greenyellow |
| TMEM63A | Fibroblasts-M2 | green |
| CCNH | Fibroblasts-M31 | darkred |
| PLSCR1 | Fibroblasts-M2 | green |
| TMED10 | Fibroblasts-M23 | darkturquoise |
| AZIN1 | Fibroblasts-M6 | turquoise |
| LAMP1 | Fibroblasts-M9 | pink |
| POLR2L | Fibroblasts-M10 | blue |
| PRMT9 | Fibroblasts-M2 | green |
| PHB | Fibroblasts-M13 | black |
| CNOT7 | Fibroblasts-M22 | paleturquoise |
| ATG3 | Fibroblasts-M13 | black |
| RWDD4 | Fibroblasts-M5 | salmon |
| TCIRG1 | Fibroblasts-M19 | orange |
| TCN2 | Fibroblasts-M7 | cyan |
| GSAP | Fibroblasts-M20 | red |
| HIST1H2BC | Fibroblasts-M6 | turquoise |
| CCDC80 | Fibroblasts-M7 | cyan |
| MYLIP | Fibroblasts-M13 | black |
| ST6GALNAC4 | Fibroblasts-M8 | magenta |
| MID1IP1 | Fibroblasts-M6 | turquoise |
| RNASEH1 | Fibroblasts-M15 | grey60 |
| CANX | Fibroblasts-M29 | darkgreen |
| MFSD3 | Fibroblasts-M7 | cyan |
| LEO1 | Fibroblasts-M8 | magenta |
| DTYMK | Fibroblasts-M4 | yellow |
| PLEK | Fibroblasts-M24 | midnightblue |
| POLD3 | Fibroblasts-M4 | yellow |
| CAV2 | Fibroblasts-M13 | black |
| NFIC | Fibroblasts-M7 | cyan |
| PAIP2 | Fibroblasts-M1 | purple |
| VPS26A | Fibroblasts-M1 | purple |
| WARS | Fibroblasts-M6 | turquoise |
| STX3 | Fibroblasts-M13 | black |
| MTUS1 | Fibroblasts-M25 | greenyellow |
| 6-Mar | Fibroblasts-M9 | pink |
| MTFR1 | Fibroblasts-M8 | magenta |
| DHDDS | Fibroblasts-M28 | skyblue |
| ARHGDIA | Fibroblasts-M18 | white |
| OPTN | Fibroblasts-M2 | green |
| ITPR1 | Fibroblasts-M5 | salmon |
| SNX4 | Fibroblasts-M8 | magenta |
| SUPV3L1 | Fibroblasts-M6 | turquoise |
| PDLIM1 | Fibroblasts-M1 | purple |
| ARAP2 | Fibroblasts-M21 | brown |
| MRPS11 | Fibroblasts-M28 | skyblue |
| NCF1C | Fibroblasts-M3 | lightyellow |
| LPIN1 | Fibroblasts-M28 | skyblue |
| HES6 | Fibroblasts-M19 | orange |
| BNIP2 | Fibroblasts-M6 | turquoise |
| STK39 | Fibroblasts-M9 | pink |
| AKR7A2 | Fibroblasts-M20 | red |
| CHMP5 | Fibroblasts-M1 | purple |
| TSPYL1 | Fibroblasts-M23 | darkturquoise |
| MSRB2 | Fibroblasts-M16 | lightcyan |
| BCL3 | Fibroblasts-M23 | darkturquoise |
| DIAPH3 | Fibroblasts-M4 | yellow |
| SEMA4D | Fibroblasts-M6 | turquoise |
| KIF1B | Fibroblasts-M29 | darkgreen |
| ABLIM1 | Fibroblasts-M10 | blue |
| ST6GALNAC1 | Fibroblasts-M28 | skyblue |
| NUP58 | Fibroblasts-M13 | black |
| YKT6 | Fibroblasts-M26 | lightgreen |
| NSMCE4A | Fibroblasts-M25 | greenyellow |
| IQGAP2 | Fibroblasts-M20 | red |
| SLC35A3 | Fibroblasts-M5 | salmon |
| TK1 | Fibroblasts-M4 | yellow |
| ELL2 | Fibroblasts-M13 | black |
| CSNK2A2 | Fibroblasts-M13 | black |
| MRPL45 | Fibroblasts-M1 | purple |
| BTN3A2 | Fibroblasts-M2 | green |
| MORF4L2 | Fibroblasts-M6 | turquoise |
| SURF1 | Fibroblasts-M25 | greenyellow |
| CTPS1 | Fibroblasts-M2 | green |
| RCHY1 | Fibroblasts-M27 | darkorange |
| ITGA1 | Fibroblasts-M8 | magenta |
| TET2 | Fibroblasts-M14 | tan |
| DNAJC11 | Fibroblasts-M6 | turquoise |
| RPS6KB2 | Fibroblasts-M25 | greenyellow |
| PAFAH1B1 | Fibroblasts-M31 | darkred |
| BLCAP | Fibroblasts-M7 | cyan |
| PEX1 | Fibroblasts-M17 | darkgrey |
| POU2F2 | Fibroblasts-M13 | black |
| GTPBP4 | Fibroblasts-M5 | salmon |
| SLC40A1 | Fibroblasts-M21 | brown |
| BTK | Fibroblasts-M10 | blue |
| CD14 | Fibroblasts-M6 | turquoise |
| CTSC | Fibroblasts-M2 | green |
| SERINC2 | Fibroblasts-M9 | pink |
| B4GALT4 | Fibroblasts-M22 | paleturquoise |
| PMP22 | Fibroblasts-M7 | cyan |
| CABIN1 | Fibroblasts-M23 | darkturquoise |
| ITPRIPL2 | Fibroblasts-M29 | darkgreen |
| KIAA0355 | Fibroblasts-M14 | tan |
| STAT2 | Fibroblasts-M20 | red |
| KANSL2 | Fibroblasts-M20 | red |
| AFAP1 | Fibroblasts-M14 | tan |
| UBE2M | Fibroblasts-M4 | yellow |
| LRP1 | Fibroblasts-M7 | cyan |
| CTSZ | Fibroblasts-M8 | magenta |
| HNRNPF | Fibroblasts-M4 | yellow |
| DDOST | Fibroblasts-M4 | yellow |
| ZMIZ1 | Fibroblasts-M21 | brown |
| TSPAN13 | Fibroblasts-M26 | lightgreen |
| AHSA1 | Fibroblasts-M13 | black |
| ENDOG | Fibroblasts-M21 | brown |
| LDLR | Fibroblasts-M6 | turquoise |
| OGFRL1 | Fibroblasts-M13 | black |
| FAS | Fibroblasts-M27 | darkorange |
| CMC2 | Fibroblasts-M26 | lightgreen |
| IGFBP4 | Fibroblasts-M7 | cyan |
| CASK | Fibroblasts-M20 | red |
| ATMIN | Fibroblasts-M9 | pink |
| ANO9 | Fibroblasts-M24 | midnightblue |
| ELMSAN1 | Fibroblasts-M6 | turquoise |
| TCEAL1 | Fibroblasts-M29 | darkgreen |
| ZBTB44 | Fibroblasts-M20 | red |
| ATP6AP2 | Fibroblasts-M2 | green |
| CGGBP1 | Fibroblasts-M13 | black |
| DDAH1 | Fibroblasts-M21 | brown |
| VASP | Fibroblasts-M21 | brown |
| FAM13A | Fibroblasts-M6 | turquoise |
| STAB1 | Fibroblasts-M6 | turquoise |
| PITPNB | Fibroblasts-M12 | steelblue |
| MX1 | Fibroblasts-M5 | salmon |
| LMBR1 | Fibroblasts-M23 | darkturquoise |
| TRIM14 | Fibroblasts-M30 | saddlebrown |
| MLH3 | Fibroblasts-M20 | red |
| RIPK2 | Fibroblasts-M6 | turquoise |
| DVL1 | Fibroblasts-M10 | blue |
| POLB | Fibroblasts-M26 | lightgreen |
| AASDH | Fibroblasts-M16 | lightcyan |
| ST3GAL1 | Fibroblasts-M6 | turquoise |
| ST3GAL5 | Fibroblasts-M7 | cyan |
| ZNF438 | Fibroblasts-M2 | green |
| RAB25 | Fibroblasts-M21 | brown |
| SEL1L | Fibroblasts-M7 | cyan |
| GLTP | Fibroblasts-M25 | greenyellow |
| RPA2 | Fibroblasts-M16 | lightcyan |
| COA6 | Fibroblasts-M13 | black |
| PRKRA | Fibroblasts-M26 | lightgreen |
| ANXA6 | Fibroblasts-M21 | brown |
| HIST1H2BN | Fibroblasts-M6 | turquoise |
| BANK1 | Fibroblasts-M10 | blue |
| GABARAPL1 | Fibroblasts-M6 | turquoise |
| GALNT7 | Fibroblasts-M9 | pink |
| DGKA | Fibroblasts-M2 | green |
| AKR7A3 | Fibroblasts-M30 | saddlebrown |
| NCOR2 | Fibroblasts-M14 | tan |
| HES4 | Fibroblasts-M8 | magenta |
| PACSIN2 | Fibroblasts-M8 | magenta |
| NXT1 | Fibroblasts-M6 | turquoise |
| CANT1 | Fibroblasts-M21 | brown |
| UBXN11 | Fibroblasts-M10 | blue |
| ADAM15 | Fibroblasts-M16 | lightcyan |
| ARL5B | Fibroblasts-M6 | turquoise |
| MBNL2 | Fibroblasts-M9 | pink |
| MECOM | Fibroblasts-M3 | lightyellow |
| DYRK4 | Fibroblasts-M18 | white |
| UVRAG | Fibroblasts-M2 | green |
| TMEM9 | Fibroblasts-M28 | skyblue |
| DDX56 | Fibroblasts-M5 | salmon |
| COL3A1 | Fibroblasts-M9 | pink |
| SART1 | Fibroblasts-M13 | black |
| ENO2 | Fibroblasts-M13 | black |
| SP1 | Fibroblasts-M2 | green |
| PDS5A | Fibroblasts-M29 | darkgreen |
| KPNA1 | Fibroblasts-M25 | greenyellow |
| TP53I11 | Fibroblasts-M6 | turquoise |
| SNHG12 | Fibroblasts-M2 | green |
| SPARC | Fibroblasts-M14 | tan |
| SMC1A | Fibroblasts-M5 | salmon |
| ARHGAP17 | Fibroblasts-M21 | brown |
| SEMA4B | Fibroblasts-M21 | brown |
| DPP4 | Fibroblasts-M15 | grey60 |
| RABL3 | Fibroblasts-M6 | turquoise |
| ATP10D | Fibroblasts-M20 | red |
| CCL20 | Fibroblasts-M29 | darkgreen |
| LMNB1 | Fibroblasts-M4 | yellow |
| MASTL | Fibroblasts-M1 | purple |
| PNKD | Fibroblasts-M21 | brown |
| TMEM9B | Fibroblasts-M2 | green |
| VHL | Fibroblasts-M1 | purple |
| NACA3P | Fibroblasts-M16 | lightcyan |
| DDX50 | Fibroblasts-M12 | steelblue |
| F2R | Fibroblasts-M2 | green |
| AEBP1 | Fibroblasts-M9 | pink |
| SHISA5 | Fibroblasts-M7 | cyan |
| ABCG1 | Fibroblasts-M2 | green |
| NAGLU | Fibroblasts-M7 | cyan |
| WASHC2C | Fibroblasts-M9 | pink |
| RASSF6 | Fibroblasts-M6 | turquoise |
| KDM3B | Fibroblasts-M15 | grey60 |
| ELOVL6 | Fibroblasts-M2 | green |
| LRRC58 | Fibroblasts-M27 | darkorange |
| KIF3B | Fibroblasts-M14 | tan |
| CPSF3 | Fibroblasts-M31 | darkred |
| GTF2H3 | Fibroblasts-M1 | purple |
| SPCS2 | Fibroblasts-M3 | lightyellow |
| EPB41L2 | Fibroblasts-M20 | red |
| MIF | Fibroblasts-M18 | white |
| PABPN1 | Fibroblasts-M22 | paleturquoise |
| ZDHHC4 | Fibroblasts-M22 | paleturquoise |
| NCKAP1 | Fibroblasts-M12 | steelblue |
| KIAA0319L | Fibroblasts-M21 | brown |
| PTPRK | Fibroblasts-M15 | grey60 |
| ALAS1 | Fibroblasts-M6 | turquoise |
| ESRRA | Fibroblasts-M18 | white |
| DHRSX | Fibroblasts-M14 | tan |
| MKI67 | Fibroblasts-M4 | yellow |
| RPL23AP2 | Fibroblasts-M16 | lightcyan |
| BAZ2A | Fibroblasts-M2 | green |
| CACYBPP2 | Fibroblasts-M21 | brown |
| FOXP1 | Fibroblasts-M27 | darkorange |
| SLC25A36 | Fibroblasts-M6 | turquoise |
| CAMK2D | Fibroblasts-M7 | cyan |
| KIN | Fibroblasts-M20 | red |
| CNST | Fibroblasts-M2 | green |
| TTF2 | Fibroblasts-M28 | skyblue |
| CDKN1A | Fibroblasts-M6 | turquoise |
| PELI1 | Fibroblasts-M21 | brown |
| NASP | Fibroblasts-M4 | yellow |
| GDE1 | Fibroblasts-M1 | purple |
| STK26 | Fibroblasts-M4 | yellow |
| CDIP1 | Fibroblasts-M6 | turquoise |
| MICAL2 | Fibroblasts-M12 | steelblue |
| COL4A2 | Fibroblasts-M8 | magenta |
| ACD | Fibroblasts-M26 | lightgreen |
| NEDD9 | Fibroblasts-M6 | turquoise |
| GPR35 | Fibroblasts-M4 | yellow |
| YWHAG | Fibroblasts-M6 | turquoise |
| RUFY2 | Fibroblasts-M21 | brown |
| SAE1 | Fibroblasts-M25 | greenyellow |
| NCK2 | Fibroblasts-M1 | purple |
| NORAD | Fibroblasts-M15 | grey60 |
| PACS2 | Fibroblasts-M20 | red |
| OTUD6B | Fibroblasts-M14 | tan |
| NIPA2 | Fibroblasts-M12 | steelblue |
| HERC5 | Fibroblasts-M7 | cyan |
| GDI1 | Fibroblasts-M18 | white |
| FGL2 | Fibroblasts-M7 | cyan |
| SNRNP48 | Fibroblasts-M8 | magenta |
| LINC01881 | Fibroblasts-M4 | yellow |
| TMEM154 | Fibroblasts-M4 | yellow |
| DHX8 | Fibroblasts-M23 | darkturquoise |
| ADD3 | Fibroblasts-M7 | cyan |
| MAPKAPK2 | Fibroblasts-M6 | turquoise |
| B3GNT2 | Fibroblasts-M21 | brown |
| MAP2K4 | Fibroblasts-M5 | salmon |
| STK38L | Fibroblasts-M29 | darkgreen |
| GNAI3 | Fibroblasts-M13 | black |
| UGCG | Fibroblasts-M20 | red |
| GAS6 | Fibroblasts-M21 | brown |
| ATXN1 | Fibroblasts-M31 | darkred |
| PPP1R35 | Fibroblasts-M16 | lightcyan |
| CETN2 | Fibroblasts-M22 | paleturquoise |
| RPL34P18 | Fibroblasts-M10 | blue |
| VIRMA | Fibroblasts-M8 | magenta |
| UGGT1 | Fibroblasts-M14 | tan |
| GTSE1 | Fibroblasts-M4 | yellow |
| TOPORS | Fibroblasts-M23 | darkturquoise |
| MRFAP1 | Fibroblasts-M13 | black |
| RBMS1 | Fibroblasts-M30 | saddlebrown |
| ROCK1 | Fibroblasts-M21 | brown |
| MTHFD2 | Fibroblasts-M21 | brown |
| RANBP2 | Fibroblasts-M14 | tan |
| DDX59 | Fibroblasts-M22 | paleturquoise |
| SNHG17 | Fibroblasts-M21 | brown |
| SAMD8 | Fibroblasts-M23 | darkturquoise |
| CCDC71L | Fibroblasts-M20 | red |
| UBA2 | Fibroblasts-M21 | brown |
| PSMG1 | Fibroblasts-M6 | turquoise |
| ABHD11 | Fibroblasts-M21 | brown |
| MRPL48 | Fibroblasts-M27 | darkorange |
| DYRK2 | Fibroblasts-M29 | darkgreen |
| GRAMD1A | Fibroblasts-M4 | yellow |
| TMEM170A | Fibroblasts-M4 | yellow |
| TOR3A | Fibroblasts-M15 | grey60 |
| MTG2 | Fibroblasts-M5 | salmon |
| PEPD | Fibroblasts-M15 | grey60 |
| NCSTN | Fibroblasts-M28 | skyblue |
| HSP90AB3P | Fibroblasts-M11 | royalblue |
| GPAA1 | Fibroblasts-M31 | darkred |
| MCM4 | Fibroblasts-M4 | yellow |
| HNRNPH3 | Fibroblasts-M8 | magenta |
| NPTN | Fibroblasts-M29 | darkgreen |
| SENP6 | Fibroblasts-M2 | green |
| NAA16 | Fibroblasts-M2 | green |
| DDX19B | Fibroblasts-M25 | greenyellow |
| RPS19P1 | Fibroblasts-M16 | lightcyan |
| PTEN | Fibroblasts-M21 | brown |
| C12orf45 | Fibroblasts-M13 | black |
| MLX | Fibroblasts-M14 | tan |
| USH1C | Fibroblasts-M17 | darkgrey |
| MIR4435-2HG | Fibroblasts-M8 | magenta |
| GGT6 | Fibroblasts-M13 | black |
| SAMD4B | Fibroblasts-M31 | darkred |
| ATF5 | Fibroblasts-M6 | turquoise |
| ATG13 | Fibroblasts-M9 | pink |
| PRR7 | Fibroblasts-M25 | greenyellow |
| ARV1 | Fibroblasts-M15 | grey60 |
| LSR | Fibroblasts-M6 | turquoise |
| 9-Sep | Fibroblasts-M21 | brown |
| FBXO11 | Fibroblasts-M31 | darkred |
| IL4R | Fibroblasts-M6 | turquoise |
| HRAS | Fibroblasts-M21 | brown |
| ABCA1 | Fibroblasts-M15 | grey60 |
| BSPRY | Fibroblasts-M3 | lightyellow |
| CHST11 | Fibroblasts-M8 | magenta |
| CLN8 | Fibroblasts-M1 | purple |
| GNB5 | Fibroblasts-M27 | darkorange |
| MIS18A | Fibroblasts-M20 | red |
| LTA4H | Fibroblasts-M20 | red |
| CTSW | Fibroblasts-M21 | brown |
| MPG | Fibroblasts-M6 | turquoise |
| BMS1 | Fibroblasts-M2 | green |
| TMEM176A | Fibroblasts-M20 | red |
| ITGAL | Fibroblasts-M24 | midnightblue |
| ARL8B | Fibroblasts-M12 | steelblue |
| RB1CC1 | Fibroblasts-M15 | grey60 |
| G6PC3 | Fibroblasts-M30 | saddlebrown |
| RNF5 | Fibroblasts-M18 | white |
| CARM1 | Fibroblasts-M19 | orange |
| ACADM | Fibroblasts-M2 | green |
| BASP1 | Fibroblasts-M10 | blue |
| AIFM2 | Fibroblasts-M6 | turquoise |
| PTGER4 | Fibroblasts-M6 | turquoise |
| RDX | Fibroblasts-M7 | cyan |
| CUX1 | Fibroblasts-M15 | grey60 |
| AC092069.1 | Fibroblasts-M19 | orange |
| RRAD | Fibroblasts-M6 | turquoise |
| UBXN6 | Fibroblasts-M7 | cyan |
| TTL | Fibroblasts-M24 | midnightblue |
| GRN | Fibroblasts-M7 | cyan |
| TMEM38B | Fibroblasts-M25 | greenyellow |
| REPS1 | Fibroblasts-M15 | grey60 |
| GOSR2 | Fibroblasts-M27 | darkorange |
| SMIM37 | Fibroblasts-M20 | red |
| ACSL4 | Fibroblasts-M6 | turquoise |
| COP1 | Fibroblasts-M24 | midnightblue |
| NUDT9 | Fibroblasts-M31 | darkred |
| UBR4 | Fibroblasts-M31 | darkred |
| MRPL20 | Fibroblasts-M18 | white |
| CWF19L2 | Fibroblasts-M2 | green |
| MRPL30 | Fibroblasts-M31 | darkred |
| LAP3 | Fibroblasts-M2 | green |
| EIF4EBP1 | Fibroblasts-M18 | white |
| MAML2 | Fibroblasts-M17 | darkgrey |
| USP9X | Fibroblasts-M12 | steelblue |
| INPP5D | Fibroblasts-M11 | royalblue |
| OSTM1 | Fibroblasts-M13 | black |
| SQOR | Fibroblasts-M6 | turquoise |
| GK5 | Fibroblasts-M22 | paleturquoise |
| TIMM29 | Fibroblasts-M24 | midnightblue |
| PPFIBP1 | Fibroblasts-M14 | tan |
| PYCARD | Fibroblasts-M2 | green |
| CPQ | Fibroblasts-M10 | blue |
| TRABD2A | Fibroblasts-M30 | saddlebrown |
| CDK17 | Fibroblasts-M12 | steelblue |
| AP1AR | Fibroblasts-M21 | brown |
| CLIP4 | Fibroblasts-M20 | red |
| PARVG | Fibroblasts-M3 | lightyellow |
| USP11 | Fibroblasts-M6 | turquoise |
| H2AFX | Fibroblasts-M4 | yellow |
| PKP4 | Fibroblasts-M4 | yellow |
| YIPF1 | Fibroblasts-M8 | magenta |
| PPP6R3 | Fibroblasts-M1 | purple |
| GOT2 | Fibroblasts-M5 | salmon |
| PIN4 | Fibroblasts-M21 | brown |
| GOT1 | Fibroblasts-M6 | turquoise |
| MED15 | Fibroblasts-M13 | black |
| SRSF1 | Fibroblasts-M5 | salmon |
| SEMA5A | Fibroblasts-M21 | brown |
| RIOK1 | Fibroblasts-M2 | green |
| SLFN12 | Fibroblasts-M2 | green |
| PTK2 | Fibroblasts-M8 | magenta |
| EEF1E1 | Fibroblasts-M13 | black |
| Z93930.2 | Fibroblasts-M10 | blue |
| HSD17B7 | Fibroblasts-M2 | green |
| VMA21 | Fibroblasts-M20 | red |
| OSGIN2 | Fibroblasts-M20 | red |
| KLHDC3 | Fibroblasts-M8 | magenta |
| SOWAHC | Fibroblasts-M13 | black |
| ZNFX1 | Fibroblasts-M5 | salmon |
| ICAM1 | Fibroblasts-M6 | turquoise |
| NBEAL2 | Fibroblasts-M19 | orange |
| INTS8 | Fibroblasts-M14 | tan |
| GNA11 | Fibroblasts-M21 | brown |
| SLC25A29 | Fibroblasts-M21 | brown |
| RPL5P1 | Fibroblasts-M10 | blue |
| MYO10 | Fibroblasts-M6 | turquoise |
| NBR1 | Fibroblasts-M5 | salmon |
| AC026403.1 | Fibroblasts-M10 | blue |
| FKBP14 | Fibroblasts-M9 | pink |
| TPM2 | Fibroblasts-M8 | magenta |
| HDDC2 | Fibroblasts-M20 | red |
| HADH | Fibroblasts-M4 | yellow |
| PRCP | Fibroblasts-M7 | cyan |
| SPART | Fibroblasts-M2 | green |
| PCGF1 | Fibroblasts-M6 | turquoise |
| CCDC93 | Fibroblasts-M14 | tan |
| DCTN6 | Fibroblasts-M1 | purple |
| SSPN | Fibroblasts-M9 | pink |
| SMIM12 | Fibroblasts-M21 | brown |
| IKBKE | Fibroblasts-M1 | purple |
| PIGL | Fibroblasts-M20 | red |
| HEATR5B | Fibroblasts-M25 | greenyellow |
| TATDN3 | Fibroblasts-M28 | skyblue |
| PAPD5 | Fibroblasts-M13 | black |
| TRIM52-AS1 | Fibroblasts-M25 | greenyellow |
| HMMR | Fibroblasts-M30 | saddlebrown |
| B3GNT5 | Fibroblasts-M4 | yellow |
| JMJD6 | Fibroblasts-M6 | turquoise |
| OSBPL9 | Fibroblasts-M29 | darkgreen |
| COASY | Fibroblasts-M2 | green |
| ITGB1BP1 | Fibroblasts-M27 | darkorange |
| ZC2HC1A | Fibroblasts-M7 | cyan |
| STX8 | Fibroblasts-M21 | brown |
| CCDC34 | Fibroblasts-M13 | black |
| TIFA | Fibroblasts-M13 | black |
| NMB | Fibroblasts-M13 | black |
| ITGA5 | Fibroblasts-M26 | lightgreen |
| MOCS2 | Fibroblasts-M5 | salmon |
| FBXO25 | Fibroblasts-M7 | cyan |
| RNF113A | Fibroblasts-M2 | green |
| OAZ2 | Fibroblasts-M21 | brown |
| PDCD7 | Fibroblasts-M20 | red |
| GUF1 | Fibroblasts-M1 | purple |
| GMPS | Fibroblasts-M22 | paleturquoise |
| HCCS | Fibroblasts-M6 | turquoise |
| BOP1 | Fibroblasts-M8 | magenta |
| GGCT | Fibroblasts-M4 | yellow |
| AL080243.2 | Fibroblasts-M16 | lightcyan |
| RNF20 | Fibroblasts-M21 | brown |
| PCCA | Fibroblasts-M6 | turquoise |
| BHLHE41 | Fibroblasts-M14 | tan |
| GEMIN6 | Fibroblasts-M4 | yellow |
| BLZF1 | Fibroblasts-M5 | salmon |
| TESC | Fibroblasts-M21 | brown |
| UBXN2A | Fibroblasts-M30 | saddlebrown |
| GMEB1 | Fibroblasts-M27 | darkorange |
| NEK3 | Fibroblasts-M31 | darkred |
| TMEM128 | Fibroblasts-M6 | turquoise |
| AP003352.1 | Fibroblasts-M10 | blue |
| MAN1B1 | Fibroblasts-M18 | white |
| TOMM34 | Fibroblasts-M20 | red |
| NELFB | Fibroblasts-M27 | darkorange |
| HDAC1 | Fibroblasts-M29 | darkgreen |
| EAF2 | Fibroblasts-M3 | lightyellow |
| KPNA4 | Fibroblasts-M19 | orange |
| BRD1 | Fibroblasts-M31 | darkred |
| TCF12 | Fibroblasts-M9 | pink |
| MED7 | Fibroblasts-M4 | yellow |
| SRBD1 | Fibroblasts-M2 | green |
| BMP2K | Fibroblasts-M20 | red |
| BICDL2 | Fibroblasts-M31 | darkred |
| UBXN7 | Fibroblasts-M17 | darkgrey |
| HMGN2P5 | Fibroblasts-M10 | blue |
| TPX2 | Fibroblasts-M4 | yellow |
| TAF1 | Fibroblasts-M29 | darkgreen |
| ATP6V0D1 | Fibroblasts-M6 | turquoise |
| CCR5 | Fibroblasts-M17 | darkgrey |
| TSR3 | Fibroblasts-M30 | saddlebrown |
| SIL1 | Fibroblasts-M15 | grey60 |
| GIMAP2 | Fibroblasts-M20 | red |
| PIGC | Fibroblasts-M7 | cyan |
| RAB20 | Fibroblasts-M6 | turquoise |
| TCEAL4 | Fibroblasts-M10 | blue |
| SPATA20 | Fibroblasts-M6 | turquoise |
| COL6A3 | Fibroblasts-M9 | pink |
| METTL17 | Fibroblasts-M17 | darkgrey |
| MAN2C1 | Fibroblasts-M25 | greenyellow |
| ZNF652 | Fibroblasts-M9 | pink |
| SDE2 | Fibroblasts-M6 | turquoise |
| BPGM | Fibroblasts-M6 | turquoise |
| FAM210A | Fibroblasts-M13 | black |
| AC246787.1 | Fibroblasts-M10 | blue |
| SGMS2 | Fibroblasts-M21 | brown |
| CCL3L1 | Fibroblasts-M30 | saddlebrown |
| HCFC1 | Fibroblasts-M15 | grey60 |
| MOB4 | Fibroblasts-M1 | purple |
| PRSS8 | Fibroblasts-M15 | grey60 |
| LYPLA1 | Fibroblasts-M6 | turquoise |
| PTGS2 | Fibroblasts-M6 | turquoise |
| IDH3A | Fibroblasts-M12 | steelblue |
| TRIM33 | Fibroblasts-M5 | salmon |
| CPNE5 | Fibroblasts-M3 | lightyellow |
| GPX3 | Fibroblasts-M10 | blue |
| NCLN | Fibroblasts-M5 | salmon |
| RPS6KA3 | Fibroblasts-M13 | black |
| BRCA2 | Fibroblasts-M4 | yellow |
| RCAN3 | Fibroblasts-M10 | blue |
| NIF3L1 | Fibroblasts-M29 | darkgreen |
| TRIP10 | Fibroblasts-M5 | salmon |
| ASNSD1 | Fibroblasts-M16 | lightcyan |
| IPO7 | Fibroblasts-M13 | black |
| HIGD1A | Fibroblasts-M16 | lightcyan |
| GRK6 | Fibroblasts-M13 | black |
| PEAK1 | Fibroblasts-M21 | brown |
| SH2D2A | Fibroblasts-M24 | midnightblue |
| CFH | Fibroblasts-M7 | cyan |
| ENDOD1 | Fibroblasts-M7 | cyan |
| TRMT13 | Fibroblasts-M2 | green |
| NUCB1 | Fibroblasts-M20 | red |
| C9orf72 | Fibroblasts-M21 | brown |
| SATB1 | Fibroblasts-M7 | cyan |
| EIF2B3 | Fibroblasts-M21 | brown |
| FMNL2 | Fibroblasts-M9 | pink |
| POLDIP2 | Fibroblasts-M13 | black |
| CKAP4 | Fibroblasts-M9 | pink |
| TRAK1 | Fibroblasts-M23 | darkturquoise |
| EIF1AY | Fibroblasts-M21 | brown |
| VDAC3 | Fibroblasts-M8 | magenta |
| HMBOX1 | Fibroblasts-M23 | darkturquoise |
| RSAD2 | Fibroblasts-M2 | green |
| HIST3H2A | Fibroblasts-M10 | blue |
| PLAUR | Fibroblasts-M26 | lightgreen |
| FGD6 | Fibroblasts-M9 | pink |
| BNIP3L | Fibroblasts-M13 | black |
| HK2 | Fibroblasts-M13 | black |
| C2CD5 | Fibroblasts-M9 | pink |
| CD302 | Fibroblasts-M20 | red |
| HCK | Fibroblasts-M14 | tan |
| PPIF | Fibroblasts-M6 | turquoise |
| ELP6 | Fibroblasts-M4 | yellow |
| PAICS | Fibroblasts-M4 | yellow |
| AKR1C3 | Fibroblasts-M10 | blue |
| C1QC | Fibroblasts-M4 | yellow |
| HSBP1L1 | Fibroblasts-M6 | turquoise |
| AKAP7 | Fibroblasts-M24 | midnightblue |
| ANAPC13 | Fibroblasts-M30 | saddlebrown |
| RPP30 | Fibroblasts-M31 | darkred |
| SLC8B1 | Fibroblasts-M2 | green |
| LMO7 | Fibroblasts-M14 | tan |
| PRR34-AS1 | Fibroblasts-M25 | greenyellow |
| ANO6 | Fibroblasts-M7 | cyan |
| CUL1 | Fibroblasts-M31 | darkred |
| PRR14 | Fibroblasts-M13 | black |
| CTNNBIP1 | Fibroblasts-M30 | saddlebrown |
| Z74021.1 | Fibroblasts-M10 | blue |
| ABI3 | Fibroblasts-M4 | yellow |
| ODF3B | Fibroblasts-M21 | brown |
| IGHG3 | Fibroblasts-M3 | lightyellow |
| MPHOSPH9 | Fibroblasts-M20 | red |
| HERC2P2 | Fibroblasts-M20 | red |
| MRPL39 | Fibroblasts-M1 | purple |
| OAS3 | Fibroblasts-M5 | salmon |
| ZNF768 | Fibroblasts-M11 | royalblue |
| CTSK | Fibroblasts-M20 | red |
| IKZF3 | Fibroblasts-M10 | blue |
| SPIN1 | Fibroblasts-M9 | pink |
| MLH1 | Fibroblasts-M8 | magenta |
| AIG1 | Fibroblasts-M1 | purple |
| RAD18 | Fibroblasts-M4 | yellow |
| SBF2 | Fibroblasts-M6 | turquoise |
| SPINT1 | Fibroblasts-M10 | blue |
| TMEM223 | Fibroblasts-M2 | green |
| QRSL1 | Fibroblasts-M21 | brown |
| SSBP3 | Fibroblasts-M21 | brown |
| MIF4GD | Fibroblasts-M21 | brown |
| MSANTD4 | Fibroblasts-M5 | salmon |
| LRRC47 | Fibroblasts-M30 | saddlebrown |
| RFK | Fibroblasts-M6 | turquoise |
| JSRP1 | Fibroblasts-M11 | royalblue |
| RAD23B | Fibroblasts-M26 | lightgreen |
| PDK1 | Fibroblasts-M13 | black |
| CRYZL2P | Fibroblasts-M30 | saddlebrown |
| NCAPD3 | Fibroblasts-M21 | brown |
| YIF1A | Fibroblasts-M8 | magenta |
| ABHD2 | Fibroblasts-M22 | paleturquoise |
| ZCCHC8 | Fibroblasts-M20 | red |
| AC004453.1 | Fibroblasts-M10 | blue |
| IFIT3 | Fibroblasts-M2 | green |
| TBRG4 | Fibroblasts-M27 | darkorange |
| CENPM | Fibroblasts-M4 | yellow |
| DNAJB12 | Fibroblasts-M25 | greenyellow |
| SF3A2 | Fibroblasts-M27 | darkorange |
| SMTN | Fibroblasts-M23 | darkturquoise |
| GGH | Fibroblasts-M4 | yellow |
| AKIP1 | Fibroblasts-M2 | green |
| ORC4 | Fibroblasts-M25 | greenyellow |
| MUC3A | Fibroblasts-M6 | turquoise |
| ZCCHC9 | Fibroblasts-M7 | cyan |
| MAP2K2 | Fibroblasts-M19 | orange |
| FAM98B | Fibroblasts-M2 | green |
| PIP4P1 | Fibroblasts-M2 | green |
| PFKP | Fibroblasts-M1 | purple |
| TARBP1 | Fibroblasts-M15 | grey60 |
| MFSD14C | Fibroblasts-M22 | paleturquoise |
| EPB41L3 | Fibroblasts-M10 | blue |
| SLC30A9 | Fibroblasts-M21 | brown |
| MRPL22 | Fibroblasts-M28 | skyblue |
| PARP8 | Fibroblasts-M2 | green |
| DUSP10 | Fibroblasts-M6 | turquoise |
| LIG3 | Fibroblasts-M29 | darkgreen |
| NUP50 | Fibroblasts-M25 | greenyellow |
| SH3D19 | Fibroblasts-M9 | pink |
| SERPINB6 | Fibroblasts-M8 | magenta |
| KRI1 | Fibroblasts-M30 | saddlebrown |
| VCP | Fibroblasts-M18 | white |
| KIF13A | Fibroblasts-M15 | grey60 |
| FBP1 | Fibroblasts-M11 | royalblue |
| RPS6KA5 | Fibroblasts-M17 | darkgrey |
| ZNF3 | Fibroblasts-M9 | pink |
| EXPH5 | Fibroblasts-M10 | blue |
| HIST1H2BJ | Fibroblasts-M6 | turquoise |
| JAZF1 | Fibroblasts-M6 | turquoise |
| THEMIS2 | Fibroblasts-M14 | tan |
| ASCC2 | Fibroblasts-M19 | orange |
| COQ2 | Fibroblasts-M4 | yellow |
| ATN1 | Fibroblasts-M12 | steelblue |
| CCNG1 | Fibroblasts-M7 | cyan |
| BCL2L14 | Fibroblasts-M31 | darkred |
| LENG1 | Fibroblasts-M23 | darkturquoise |
| DENND6A | Fibroblasts-M2 | green |
| CMTM8 | Fibroblasts-M4 | yellow |
| TSC1 | Fibroblasts-M24 | midnightblue |
| MAP1LC3A | Fibroblasts-M21 | brown |
| THUMPD2 | Fibroblasts-M27 | darkorange |
| NIT2 | Fibroblasts-M21 | brown |
| YBEY | Fibroblasts-M28 | skyblue |
| TMEM191A | Fibroblasts-M25 | greenyellow |
| ANTXR2 | Fibroblasts-M9 | pink |
| FBXW2 | Fibroblasts-M19 | orange |
| SCD | Fibroblasts-M6 | turquoise |
| NFKBIE | Fibroblasts-M6 | turquoise |
| PRKD3 | Fibroblasts-M9 | pink |
| EEF1A1P6 | Fibroblasts-M3 | lightyellow |
| TUBB2A | Fibroblasts-M6 | turquoise |
| KDM3A | Fibroblasts-M6 | turquoise |
| PPARG | Fibroblasts-M30 | saddlebrown |
| SLCO2B1 | Fibroblasts-M5 | salmon |
| OAT | Fibroblasts-M6 | turquoise |
| ZRANB1 | Fibroblasts-M1 | purple |
| NR2C1 | Fibroblasts-M5 | salmon |
| PPARD | Fibroblasts-M12 | steelblue |
| ZNF124 | Fibroblasts-M6 | turquoise |
| PSMD5 | Fibroblasts-M17 | darkgrey |
| PILRA | Fibroblasts-M10 | blue |
| NBDY | Fibroblasts-M31 | darkred |
| SMAD1 | Fibroblasts-M6 | turquoise |
| AGPAT3 | Fibroblasts-M13 | black |
| RAB35 | Fibroblasts-M20 | red |
| TUBGCP5 | Fibroblasts-M4 | yellow |
| DDX20 | Fibroblasts-M2 | green |
| HSPA14 | Fibroblasts-M28 | skyblue |
| TNIP2 | Fibroblasts-M12 | steelblue |
| FARS2 | Fibroblasts-M1 | purple |
| INPP4B | Fibroblasts-M21 | brown |
| EPS8L2 | Fibroblasts-M13 | black |
| TFDP2 | Fibroblasts-M9 | pink |
| EZH1 | Fibroblasts-M7 | cyan |
| RNF139 | Fibroblasts-M31 | darkred |
| GABPB1 | Fibroblasts-M1 | purple |
| SAR1A | Fibroblasts-M6 | turquoise |
| BRAF | Fibroblasts-M21 | brown |
| ING1 | Fibroblasts-M23 | darkturquoise |
| ITGAE | Fibroblasts-M16 | lightcyan |
| FANCL | Fibroblasts-M4 | yellow |
| ZSWIM6 | Fibroblasts-M25 | greenyellow |
| RHOQ | Fibroblasts-M31 | darkred |
| TRAFD1 | Fibroblasts-M9 | pink |
| MYH14 | Fibroblasts-M19 | orange |
| HDDC3 | Fibroblasts-M15 | grey60 |
| COX15 | Fibroblasts-M21 | brown |
| HILPDA | Fibroblasts-M13 | black |
| C5orf51 | Fibroblasts-M6 | turquoise |
| C15orf61 | Fibroblasts-M19 | orange |
| AC007952.4 | Fibroblasts-M10 | blue |
| SRM | Fibroblasts-M16 | lightcyan |
| PSMG3 | Fibroblasts-M26 | lightgreen |
| POC5 | Fibroblasts-M22 | paleturquoise |
| SLC39A4 | Fibroblasts-M17 | darkgrey |
| ACTL6A | Fibroblasts-M8 | magenta |
| EIPR1 | Fibroblasts-M21 | brown |
| NINJ1 | Fibroblasts-M6 | turquoise |
| DFFA | Fibroblasts-M5 | salmon |
| NOL10 | Fibroblasts-M5 | salmon |
| ZSCAN16-AS1 | Fibroblasts-M7 | cyan |
| PPP1R14B | Fibroblasts-M23 | darkturquoise |
| TMEM242 | Fibroblasts-M21 | brown |
| FAH | Fibroblasts-M6 | turquoise |
| TIA1 | Fibroblasts-M9 | pink |
| TMED3 | Fibroblasts-M20 | red |
| FUOM | Fibroblasts-M4 | yellow |
| SMIM14 | Fibroblasts-M7 | cyan |
| BTN3A3 | Fibroblasts-M2 | green |
| NELFE | Fibroblasts-M1 | purple |
| RASSF7 | Fibroblasts-M1 | purple |
| BABAM1 | Fibroblasts-M25 | greenyellow |
| HMGB3 | Fibroblasts-M4 | yellow |
| PDXK | Fibroblasts-M4 | yellow |
| TNPO1 | Fibroblasts-M9 | pink |
| MRPL10 | Fibroblasts-M20 | red |
| DAPP1 | Fibroblasts-M17 | darkgrey |
| IFT57 | Fibroblasts-M17 | darkgrey |
| TMCC1 | Fibroblasts-M27 | darkorange |
| KLF9 | Fibroblasts-M21 | brown |
| SPON2 | Fibroblasts-M6 | turquoise |
| TRIB1 | Fibroblasts-M6 | turquoise |
| SURF6 | Fibroblasts-M10 | blue |
| ZFAND3 | Fibroblasts-M9 | pink |
| DNAJC25 | Fibroblasts-M19 | orange |
| TMEM126B | Fibroblasts-M5 | salmon |
| GOLM1 | Fibroblasts-M9 | pink |
| CMIP | Fibroblasts-M24 | midnightblue |
| MILR1 | Fibroblasts-M21 | brown |
| VWA5A | Fibroblasts-M6 | turquoise |
| MCM5 | Fibroblasts-M4 | yellow |
| CHMP4C | Fibroblasts-M13 | black |
| HLA-DMB | Fibroblasts-M10 | blue |
| TPCN1 | Fibroblasts-M8 | magenta |
| METAP1 | Fibroblasts-M25 | greenyellow |
| TGFBI | Fibroblasts-M6 | turquoise |
| MAK16 | Fibroblasts-M1 | purple |
| TNFAIP1 | Fibroblasts-M1 | purple |
| CDKAL1 | Fibroblasts-M29 | darkgreen |
| EEF1AKMT2 | Fibroblasts-M22 | paleturquoise |
| TMEM60 | Fibroblasts-M5 | salmon |
| TBXAS1 | Fibroblasts-M6 | turquoise |
| MS4A6A | Fibroblasts-M17 | darkgrey |
| SPPL3 | Fibroblasts-M15 | grey60 |
| C1orf131 | Fibroblasts-M14 | tan |
| PKIG | Fibroblasts-M6 | turquoise |
| TXNDC9 | Fibroblasts-M12 | steelblue |
| ERAP1 | Fibroblasts-M5 | salmon |
| SGO2 | Fibroblasts-M4 | yellow |
| COL5A1 | Fibroblasts-M9 | pink |
| FAM208B | Fibroblasts-M29 | darkgreen |
| COQ8A | Fibroblasts-M10 | blue |
| NQO1 | Fibroblasts-M6 | turquoise |
| CEP104 | Fibroblasts-M29 | darkgreen |
| ISCA2 | Fibroblasts-M24 | midnightblue |
| MX2 | Fibroblasts-M2 | green |
| GIMAP1 | Fibroblasts-M25 | greenyellow |
| LRRK1 | Fibroblasts-M20 | red |
| ZBTB21 | Fibroblasts-M6 | turquoise |
| NCK1 | Fibroblasts-M6 | turquoise |
| KPNA2 | Fibroblasts-M6 | turquoise |
| DNAJB9 | Fibroblasts-M13 | black |
| PSMD8 | Fibroblasts-M18 | white |
| FN1 | Fibroblasts-M14 | tan |
| DTWD1 | Fibroblasts-M9 | pink |
| RICTOR | Fibroblasts-M31 | darkred |
| TM4SF1 | Fibroblasts-M6 | turquoise |
| ZMPSTE24 | Fibroblasts-M22 | paleturquoise |
| APOL1 | Fibroblasts-M5 | salmon |
| SNX27 | Fibroblasts-M15 | grey60 |
| C1orf56 | Fibroblasts-M24 | midnightblue |
| ABCD3 | Fibroblasts-M28 | skyblue |
| POP7 | Fibroblasts-M4 | yellow |
| SDF4 | Fibroblasts-M20 | red |
| PDLIM5 | Fibroblasts-M8 | magenta |
| PPTC7 | Fibroblasts-M13 | black |
| KLHDC10 | Fibroblasts-M13 | black |
| NEK7 | Fibroblasts-M8 | magenta |
| DDHD1 | Fibroblasts-M6 | turquoise |
| SENP2 | Fibroblasts-M9 | pink |
| ERRFI1 | Fibroblasts-M6 | turquoise |
| SETD7 | Fibroblasts-M15 | grey60 |
| SLC35C2 | Fibroblasts-M9 | pink |
| HSF1 | Fibroblasts-M6 | turquoise |
| SNRPN | Fibroblasts-M10 | blue |
| RTF2 | Fibroblasts-M1 | purple |
| SELPLG | Fibroblasts-M21 | brown |
| RNF43 | Fibroblasts-M30 | saddlebrown |
| MRTO4 | Fibroblasts-M4 | yellow |
| RANGRF | Fibroblasts-M5 | salmon |
| ACAT2 | Fibroblasts-M6 | turquoise |
| CXXC5 | Fibroblasts-M10 | blue |
| MRPL28 | Fibroblasts-M26 | lightgreen |
| FCGR2B | Fibroblasts-M3 | lightyellow |
| TCF4 | Fibroblasts-M5 | salmon |
| NAA15 | Fibroblasts-M1 | purple |
| SNHG16 | Fibroblasts-M6 | turquoise |
| ABI1 | Fibroblasts-M13 | black |
| CBFB | Fibroblasts-M6 | turquoise |
| KRTCAP3 | Fibroblasts-M13 | black |
| MAPK14 | Fibroblasts-M9 | pink |
| ISOC1 | Fibroblasts-M20 | red |
| TTLL12 | Fibroblasts-M13 | black |
| DAZAP1 | Fibroblasts-M25 | greenyellow |
| VPS13D | Fibroblasts-M15 | grey60 |
| MITF | Fibroblasts-M15 | grey60 |
| FOXO1 | Fibroblasts-M9 | pink |
| MAP3K2 | Fibroblasts-M9 | pink |
| SLC25A19 | Fibroblasts-M30 | saddlebrown |
| CROCCP2 | Fibroblasts-M10 | blue |
| ATP2B4 | Fibroblasts-M8 | magenta |
| CINP | Fibroblasts-M7 | cyan |
| MTMR14 | Fibroblasts-M18 | white |
| SLC20A2 | Fibroblasts-M18 | white |
| DGKZ | Fibroblasts-M21 | brown |
| CSF3R | Fibroblasts-M21 | brown |
| ZNF121 | Fibroblasts-M8 | magenta |
| RNF216 | Fibroblasts-M27 | darkorange |
| GNL3L | Fibroblasts-M30 | saddlebrown |
| ATP6V1B2 | Fibroblasts-M13 | black |
| CAPN1 | Fibroblasts-M21 | brown |
| ANKFY1 | Fibroblasts-M9 | pink |
| TNFRSF10A | Fibroblasts-M13 | black |
| METTL7A | Fibroblasts-M7 | cyan |
| CTSO | Fibroblasts-M9 | pink |
| AHR | Fibroblasts-M23 | darkturquoise |
| SORL1 | Fibroblasts-M19 | orange |
| PTK2B | Fibroblasts-M10 | blue |
| TC2N | Fibroblasts-M17 | darkgrey |
| PALLD | Fibroblasts-M14 | tan |
| UBE2V2 | Fibroblasts-M18 | white |
| LINC00909 | Fibroblasts-M15 | grey60 |
| PPM1A | Fibroblasts-M20 | red |
| PDHX | Fibroblasts-M2 | green |
| ATL2 | Fibroblasts-M13 | black |
| TBC1D2B | Fibroblasts-M23 | darkturquoise |
| CETN3 | Fibroblasts-M28 | skyblue |
| CCNQ | Fibroblasts-M6 | turquoise |
| PEA15 | Fibroblasts-M13 | black |
| PPP5C | Fibroblasts-M6 | turquoise |
| COQ10B | Fibroblasts-M6 | turquoise |
| ARHGAP27 | Fibroblasts-M10 | blue |
| LINC01681 | Fibroblasts-M30 | saddlebrown |
| PROCR | Fibroblasts-M20 | red |
| COG6 | Fibroblasts-M9 | pink |
| PFDN4 | Fibroblasts-M19 | orange |
| ABRAXAS2 | Fibroblasts-M5 | salmon |
| PLTP | Fibroblasts-M10 | blue |
| USP5 | Fibroblasts-M4 | yellow |
| SURF4 | Fibroblasts-M1 | purple |
| MVB12A | Fibroblasts-M21 | brown |
| PTRH2 | Fibroblasts-M6 | turquoise |
| GIPC1 | Fibroblasts-M16 | lightcyan |
| DCUN1D5 | Fibroblasts-M20 | red |
| CGN | Fibroblasts-M14 | tan |
| TMEM161A | Fibroblasts-M15 | grey60 |
| PLCG2 | Fibroblasts-M13 | black |
| SLC25A26 | Fibroblasts-M13 | black |
| BANP | Fibroblasts-M25 | greenyellow |
| TACC2 | Fibroblasts-M28 | skyblue |
| JARID2 | Fibroblasts-M21 | brown |
| PDCD2 | Fibroblasts-M19 | orange |
| TBC1D9B | Fibroblasts-M13 | black |
| PSD3 | Fibroblasts-M6 | turquoise |
| CDCA8 | Fibroblasts-M4 | yellow |
| TMEM140 | Fibroblasts-M5 | salmon |
| MAGT1 | Fibroblasts-M5 | salmon |
| NR2C2AP | Fibroblasts-M4 | yellow |
| CBL | Fibroblasts-M21 | brown |
| NUDT1 | Fibroblasts-M7 | cyan |
| XPOT | Fibroblasts-M9 | pink |
| RAB3GAP2 | Fibroblasts-M5 | salmon |
| LAMA3 | Fibroblasts-M15 | grey60 |
| NUDT16L1 | Fibroblasts-M18 | white |
| EDEM1 | Fibroblasts-M6 | turquoise |
| FAM213A | Fibroblasts-M2 | green |
| AP000769.1 | Fibroblasts-M13 | black |
| TACC1 | Fibroblasts-M21 | brown |
| ATP11C | Fibroblasts-M31 | darkred |
| HAVCR2 | Fibroblasts-M21 | brown |
| CYR61 | Fibroblasts-M9 | pink |
| SGO1 | Fibroblasts-M4 | yellow |
| AEBP2 | Fibroblasts-M20 | red |
| ERGIC1 | Fibroblasts-M12 | steelblue |
| KMT2B | Fibroblasts-M20 | red |
| SLC30A1 | Fibroblasts-M6 | turquoise |
| NBL1 | Fibroblasts-M20 | red |
| SLC10A3 | Fibroblasts-M4 | yellow |
| ITGB2 | Fibroblasts-M19 | orange |
| MRPL58 | Fibroblasts-M25 | greenyellow |
| TNFRSF10B | Fibroblasts-M6 | turquoise |
| GSTZ1 | Fibroblasts-M10 | blue |
| YAE1D1 | Fibroblasts-M14 | tan |
| EMC2 | Fibroblasts-M6 | turquoise |
| ZNF580 | Fibroblasts-M1 | purple |
| MAPK8 | Fibroblasts-M26 | lightgreen |
| RASSF2 | Fibroblasts-M2 | green |
| PGAM1 | Fibroblasts-M13 | black |
| ADK | Fibroblasts-M28 | skyblue |
| PSME3 | Fibroblasts-M22 | paleturquoise |
| POLR2C | Fibroblasts-M25 | greenyellow |
| ATP13A3 | Fibroblasts-M13 | black |
| WIPI1 | Fibroblasts-M15 | grey60 |
| NFKBIB | Fibroblasts-M6 | turquoise |
| DDAH2 | Fibroblasts-M10 | blue |
| AL049873.1 | Fibroblasts-M10 | blue |
| LINC01184 | Fibroblasts-M21 | brown |
| EBPL | Fibroblasts-M20 | red |
| TMEM33 | Fibroblasts-M4 | yellow |
| ZDHHC24 | Fibroblasts-M22 | paleturquoise |
| LXN | Fibroblasts-M7 | cyan |
| SCAP | Fibroblasts-M27 | darkorange |
| PYCR1 | Fibroblasts-M14 | tan |
| FUNDC2 | Fibroblasts-M7 | cyan |
| CYBRD1 | Fibroblasts-M15 | grey60 |
| 2-Mar | Fibroblasts-M1 | purple |
| FAM105A | Fibroblasts-M2 | green |
| SMAD7 | Fibroblasts-M26 | lightgreen |
| KDM5C | Fibroblasts-M2 | green |
| VWA1 | Fibroblasts-M10 | blue |
| PCSK7 | Fibroblasts-M14 | tan |
| NFIB | Fibroblasts-M10 | blue |
| TYMS | Fibroblasts-M4 | yellow |
| PLEKHA6 | Fibroblasts-M20 | red |
| NAPRT | Fibroblasts-M28 | skyblue |
| MAGOHB | Fibroblasts-M31 | darkred |
| UTP11 | Fibroblasts-M18 | white |
| AQP3 | Fibroblasts-M10 | blue |
| TUBGCP3 | Fibroblasts-M29 | darkgreen |
| TAF6 | Fibroblasts-M9 | pink |
| SLC39A3 | Fibroblasts-M3 | lightyellow |
| BAIAP2L2 | Fibroblasts-M4 | yellow |
| PAM | Fibroblasts-M9 | pink |
| AKAP12 | Fibroblasts-M7 | cyan |
| RNF135 | Fibroblasts-M30 | saddlebrown |
| POLR2A | Fibroblasts-M2 | green |
| TACO1 | Fibroblasts-M29 | darkgreen |
| SLC8A1 | Fibroblasts-M21 | brown |
| UBR3 | Fibroblasts-M31 | darkred |
| PFKFB3 | Fibroblasts-M6 | turquoise |
| SEC23A | Fibroblasts-M14 | tan |
| GMPPB | Fibroblasts-M3 | lightyellow |
| MAPKAPK3 | Fibroblasts-M17 | darkgrey |
| COG1 | Fibroblasts-M5 | salmon |
| CDA | Fibroblasts-M13 | black |
| ANKRD13C | Fibroblasts-M15 | grey60 |
| NOL9 | Fibroblasts-M12 | steelblue |
| WAC-AS1 | Fibroblasts-M16 | lightcyan |
| SNX12 | Fibroblasts-M29 | darkgreen |
| HACD4 | Fibroblasts-M9 | pink |
| NUBP2 | Fibroblasts-M21 | brown |
| DHX37 | Fibroblasts-M6 | turquoise |
| SLC22A18 | Fibroblasts-M7 | cyan |
| MYO1B | Fibroblasts-M21 | brown |
| CCRL2 | Fibroblasts-M5 | salmon |
| SH3PXD2A | Fibroblasts-M15 | grey60 |
| IL15RA | Fibroblasts-M6 | turquoise |
| RPL9P7 | Fibroblasts-M10 | blue |
| PGM2 | Fibroblasts-M1 | purple |
| AL138963.3 | Fibroblasts-M10 | blue |
| RGS3 | Fibroblasts-M6 | turquoise |
| CD27 | Fibroblasts-M3 | lightyellow |
| DERA | Fibroblasts-M9 | pink |
| PSMA2 | Fibroblasts-M16 | lightcyan |
| YBX1P1 | Fibroblasts-M10 | blue |
| CREBZF | Fibroblasts-M20 | red |
| GORAB | Fibroblasts-M31 | darkred |
| C20orf27 | Fibroblasts-M21 | brown |
| BCLAF3 | Fibroblasts-M9 | pink |
| PLXDC2 | Fibroblasts-M9 | pink |
| PES1 | Fibroblasts-M6 | turquoise |
| GRAMD2B | Fibroblasts-M17 | darkgrey |
| VARS | Fibroblasts-M2 | green |
| PIKFYVE | Fibroblasts-M9 | pink |
| ZNF160 | Fibroblasts-M2 | green |
| RPS13P2 | Fibroblasts-M10 | blue |
| AGA | Fibroblasts-M30 | saddlebrown |
| PPWD1 | Fibroblasts-M20 | red |
| EREG | Fibroblasts-M4 | yellow |
| RUFY3 | Fibroblasts-M5 | salmon |
| CHPF | Fibroblasts-M14 | tan |
| TARDBP | Fibroblasts-M16 | lightcyan |
| AL031727.1 | Fibroblasts-M24 | midnightblue |
| PTDSS1 | Fibroblasts-M2 | green |
| UEVLD | Fibroblasts-M21 | brown |
| TOP2A | Fibroblasts-M4 | yellow |
| MBOAT7 | Fibroblasts-M13 | black |
| SDC3 | Fibroblasts-M7 | cyan |
| SLC44A1 | Fibroblasts-M9 | pink |
| WDSUB1 | Fibroblasts-M27 | darkorange |
| ZFYVE21 | Fibroblasts-M17 | darkgrey |
| NUP54 | Fibroblasts-M20 | red |
| BICD2 | Fibroblasts-M7 | cyan |
| TRIM69 | Fibroblasts-M4 | yellow |
| SCO1 | Fibroblasts-M2 | green |
| CASP10 | Fibroblasts-M30 | saddlebrown |
| PPIC | Fibroblasts-M7 | cyan |
| APOLD1 | Fibroblasts-M21 | brown |
| SELENOH | Fibroblasts-M10 | blue |
| PET100 | Fibroblasts-M10 | blue |
| SERTAD2 | Fibroblasts-M20 | red |
| CYB561A3 | Fibroblasts-M15 | grey60 |
| HS2ST1 | Fibroblasts-M15 | grey60 |
| TRIM26 | Fibroblasts-M4 | yellow |
| TRIM25 | Fibroblasts-M8 | magenta |
| SYK | Fibroblasts-M24 | midnightblue |
| NUP88 | Fibroblasts-M13 | black |
| NFIL3 | Fibroblasts-M24 | midnightblue |
| SDC1 | Fibroblasts-M4 | yellow |
| RNF8 | Fibroblasts-M14 | tan |
| STK24 | Fibroblasts-M23 | darkturquoise |
| AC245140.2 | Fibroblasts-M10 | blue |
| C1RL | Fibroblasts-M9 | pink |
| SCPEP1 | Fibroblasts-M7 | cyan |
| ITGAV | Fibroblasts-M29 | darkgreen |
| SNX8 | Fibroblasts-M12 | steelblue |
| CREB3L1 | Fibroblasts-M9 | pink |
| SH3RF1 | Fibroblasts-M21 | brown |
| COBLL1 | Fibroblasts-M21 | brown |
| DDB2 | Fibroblasts-M20 | red |
| MRPL50 | Fibroblasts-M21 | brown |
| DLAT | Fibroblasts-M21 | brown |
| DTX2 | Fibroblasts-M29 | darkgreen |
| NMT1 | Fibroblasts-M10 | blue |
| AIMP2 | Fibroblasts-M1 | purple |
| DMKN | Fibroblasts-M20 | red |
| U2AF2 | Fibroblasts-M13 | black |
| PIGH | Fibroblasts-M12 | steelblue |
| DUSP18 | Fibroblasts-M13 | black |
| SLC25A4 | Fibroblasts-M19 | orange |
| HTATIP2 | Fibroblasts-M21 | brown |
| SCARB2 | Fibroblasts-M9 | pink |
| NUP85 | Fibroblasts-M4 | yellow |
| PRR11 | Fibroblasts-M4 | yellow |
| P4HA2 | Fibroblasts-M13 | black |
| BRD3 | Fibroblasts-M15 | grey60 |
| COG4 | Fibroblasts-M4 | yellow |
| HSD17B8 | Fibroblasts-M30 | saddlebrown |
| L3MBTL2 | Fibroblasts-M9 | pink |
| DDX58 | Fibroblasts-M2 | green |
| HIVEP2 | Fibroblasts-M12 | steelblue |
| SORD | Fibroblasts-M28 | skyblue |
| RNFT1 | Fibroblasts-M31 | darkred |
| FUT8 | Fibroblasts-M9 | pink |
| TPST2 | Fibroblasts-M26 | lightgreen |
| MTMR2 | Fibroblasts-M8 | magenta |
| ZNF506 | Fibroblasts-M6 | turquoise |
| TBC1D1 | Fibroblasts-M8 | magenta |
| WASHC5 | Fibroblasts-M5 | salmon |
| FKBP4 | Fibroblasts-M6 | turquoise |
| C5AR1 | Fibroblasts-M6 | turquoise |
| SLC25A38 | Fibroblasts-M2 | green |
| DRAM1 | Fibroblasts-M20 | red |
| PIAS2 | Fibroblasts-M6 | turquoise |
| RPL41P5 | Fibroblasts-M10 | blue |
| CARD8 | Fibroblasts-M29 | darkgreen |
| LRBA | Fibroblasts-M15 | grey60 |
| SH3BP5 | Fibroblasts-M7 | cyan |
| CSE1L | Fibroblasts-M13 | black |
| EED | Fibroblasts-M14 | tan |
| TMEM165 | Fibroblasts-M8 | magenta |
| RPL12P4 | Fibroblasts-M10 | blue |
| DEF8 | Fibroblasts-M19 | orange |
| C11orf54 | Fibroblasts-M5 | salmon |
| BRWD3 | Fibroblasts-M6 | turquoise |
| SMG5 | Fibroblasts-M4 | yellow |
| ZBP1 | Fibroblasts-M3 | lightyellow |
| GPR107 | Fibroblasts-M20 | red |
| AC008038.1 | Fibroblasts-M6 | turquoise |
| C2orf69 | Fibroblasts-M5 | salmon |
| JRKL | Fibroblasts-M22 | paleturquoise |
| ITGAM | Fibroblasts-M6 | turquoise |
| MMP7 | Fibroblasts-M19 | orange |
| CENPN | Fibroblasts-M4 | yellow |
| HEIH | Fibroblasts-M16 | lightcyan |
| EP300 | Fibroblasts-M9 | pink |
| BRAP | Fibroblasts-M6 | turquoise |
| CPT2 | Fibroblasts-M20 | red |
| KCTD10 | Fibroblasts-M23 | darkturquoise |
| RRM1 | Fibroblasts-M4 | yellow |
| TRIM24 | Fibroblasts-M22 | paleturquoise |
| RAB6A | Fibroblasts-M27 | darkorange |
| WDR5 | Fibroblasts-M6 | turquoise |
| HSPA6 | Fibroblasts-M6 | turquoise |
| IFIT2 | Fibroblasts-M2 | green |
| AC079250.1 | Fibroblasts-M4 | yellow |
| MYO1D | Fibroblasts-M31 | darkred |
| SIPA1L1 | Fibroblasts-M14 | tan |
| VEZT | Fibroblasts-M5 | salmon |
| SMCR8 | Fibroblasts-M2 | green |
| OAF | Fibroblasts-M7 | cyan |
| ABCD4 | Fibroblasts-M2 | green |
| PTRH1 | Fibroblasts-M2 | green |
| CCNB1 | Fibroblasts-M4 | yellow |
| FAM45A | Fibroblasts-M20 | red |
| SCARB1 | Fibroblasts-M6 | turquoise |
| KCNQ1 | Fibroblasts-M6 | turquoise |
| MEST | Fibroblasts-M2 | green |
| MT-ND6 | Fibroblasts-M25 | greenyellow |
| FRS2 | Fibroblasts-M5 | salmon |
| ERCC6L2 | Fibroblasts-M2 | green |
| ADGRE2 | Fibroblasts-M2 | green |
| NR2F6 | Fibroblasts-M16 | lightcyan |
| ABHD14A | Fibroblasts-M10 | blue |
| HMOX1 | Fibroblasts-M6 | turquoise |
| DAG1 | Fibroblasts-M21 | brown |
| TLR2 | Fibroblasts-M4 | yellow |
| FASTKD1 | Fibroblasts-M7 | cyan |
| ARCN1 | Fibroblasts-M12 | steelblue |
| C1orf198 | Fibroblasts-M8 | magenta |
| TSC22D4 | Fibroblasts-M21 | brown |
| RFC2 | Fibroblasts-M4 | yellow |
| RPS2P5 | Fibroblasts-M21 | brown |
| AC115223.1 | Fibroblasts-M12 | steelblue |
| PCBD2 | Fibroblasts-M8 | magenta |
| LAMA2 | Fibroblasts-M20 | red |
| ZWILCH | Fibroblasts-M4 | yellow |
| TRAPPC13 | Fibroblasts-M23 | darkturquoise |
| CAND1 | Fibroblasts-M14 | tan |
| DHRS3 | Fibroblasts-M10 | blue |
| DHFR | Fibroblasts-M4 | yellow |
| KRT7 | Fibroblasts-M10 | blue |
| TEX10 | Fibroblasts-M13 | black |
| ECM1 | Fibroblasts-M6 | turquoise |
| DLGAP5 | Fibroblasts-M4 | yellow |
| GANAB | Fibroblasts-M20 | red |
| HDAC2 | Fibroblasts-M26 | lightgreen |
| DCTN1 | Fibroblasts-M29 | darkgreen |
| CEP63 | Fibroblasts-M16 | lightcyan |
| PGD | Fibroblasts-M15 | grey60 |
| PAFAH1B2 | Fibroblasts-M15 | grey60 |
| TRAPPC6B | Fibroblasts-M22 | paleturquoise |
| EHBP1 | Fibroblasts-M5 | salmon |
| CNOT3 | Fibroblasts-M6 | turquoise |
| PDZD8 | Fibroblasts-M14 | tan |
| CDC23 | Fibroblasts-M22 | paleturquoise |
| ACSF3 | Fibroblasts-M27 | darkorange |
| SLC43A3 | Fibroblasts-M27 | darkorange |
| CIAPIN1 | Fibroblasts-M21 | brown |
| WLS | Fibroblasts-M6 | turquoise |
| LEFTY1 | Fibroblasts-M24 | midnightblue |
| MAD2L2 | Fibroblasts-M4 | yellow |
| NAB1 | Fibroblasts-M25 | greenyellow |
| HTRA1 | Fibroblasts-M15 | grey60 |
| SYMPK | Fibroblasts-M25 | greenyellow |
| ENO3 | Fibroblasts-M10 | blue |
| AIM2 | Fibroblasts-M26 | lightgreen |
| QDPR | Fibroblasts-M20 | red |
| GBE1 | Fibroblasts-M26 | lightgreen |
| SLC44A2 | Fibroblasts-M29 | darkgreen |
| THAP7 | Fibroblasts-M27 | darkorange |
| OSBPL1A | Fibroblasts-M13 | black |
| BAMBI | Fibroblasts-M6 | turquoise |
| RPGR | Fibroblasts-M13 | black |
| TTK | Fibroblasts-M4 | yellow |
| NCAPD2 | Fibroblasts-M4 | yellow |
| PCOLCE | Fibroblasts-M9 | pink |
| PYGL | Fibroblasts-M15 | grey60 |
| ALDH3B1 | Fibroblasts-M6 | turquoise |
| SORT1 | Fibroblasts-M8 | magenta |
| IRF6 | Fibroblasts-M6 | turquoise |
| NDC1 | Fibroblasts-M21 | brown |
| KIF9 | Fibroblasts-M26 | lightgreen |
| PITPNA | Fibroblasts-M2 | green |
| CDK1 | Fibroblasts-M4 | yellow |
| IFNAR2 | Fibroblasts-M10 | blue |
| OASL | Fibroblasts-M2 | green |
| UCKL1 | Fibroblasts-M19 | orange |
| CYP27A1 | Fibroblasts-M7 | cyan |
| PITRM1 | Fibroblasts-M25 | greenyellow |
| PRDM4 | Fibroblasts-M14 | tan |
| MSANTD3 | Fibroblasts-M13 | black |
| UMPS | Fibroblasts-M25 | greenyellow |
| BGN | Fibroblasts-M14 | tan |
| TRIP4 | Fibroblasts-M31 | darkred |
| SCRN1 | Fibroblasts-M2 | green |
| S100A16 | Fibroblasts-M14 | tan |
| BPHL | Fibroblasts-M4 | yellow |
| TMEM168 | Fibroblasts-M30 | saddlebrown |
| TACSTD2 | Fibroblasts-M13 | black |
| GSTO2 | Fibroblasts-M4 | yellow |
| GMFB | Fibroblasts-M14 | tan |
| ZNF92 | Fibroblasts-M4 | yellow |
| TGFBR1 | Fibroblasts-M14 | tan |
| PDPN | Fibroblasts-M20 | red |
| CYB5R4 | Fibroblasts-M6 | turquoise |
| KLK1 | Fibroblasts-M6 | turquoise |
| SUSD6 | Fibroblasts-M12 | steelblue |
| RBM12 | Fibroblasts-M1 | purple |
| BTBD7 | Fibroblasts-M7 | cyan |
| DOCK7 | Fibroblasts-M9 | pink |
| DIEXF | Fibroblasts-M13 | black |
| CSTA | Fibroblasts-M30 | saddlebrown |
| PIGF | Fibroblasts-M14 | tan |
| CEP55 | Fibroblasts-M4 | yellow |
| TWF2 | Fibroblasts-M16 | lightcyan |
| DPP8 | Fibroblasts-M5 | salmon |
| IFIH1 | Fibroblasts-M5 | salmon |
| FAM110A | Fibroblasts-M31 | darkred |
| TMEM243 | Fibroblasts-M5 | salmon |
| MANBA | Fibroblasts-M6 | turquoise |
| PTGR1 | Fibroblasts-M25 | greenyellow |
| LAMA4 | Fibroblasts-M23 | darkturquoise |
| CSNK1E | Fibroblasts-M23 | darkturquoise |
| LONP1 | Fibroblasts-M1 | purple |
| KCNK1 | Fibroblasts-M17 | darkgrey |
| ATP11A | Fibroblasts-M6 | turquoise |
| GLMP | Fibroblasts-M7 | cyan |
| SAP30 | Fibroblasts-M31 | darkred |
| TMEM56 | Fibroblasts-M10 | blue |
| CD40 | Fibroblasts-M2 | green |
| BIN3 | Fibroblasts-M31 | darkred |
| LINC01943 | Fibroblasts-M29 | darkgreen |
| RNF146 | Fibroblasts-M7 | cyan |
| RHNO1 | Fibroblasts-M4 | yellow |
| SNTB1 | Fibroblasts-M6 | turquoise |
| IPMK | Fibroblasts-M6 | turquoise |
| ADH5 | Fibroblasts-M2 | green |
| TMEM251 | Fibroblasts-M13 | black |
| TMEM189 | Fibroblasts-M6 | turquoise |
| XPNPEP3 | Fibroblasts-M13 | black |
| PARVA | Fibroblasts-M29 | darkgreen |
| FKBP11 | Fibroblasts-M4 | yellow |
| FAM174A | Fibroblasts-M27 | darkorange |
| POM121 | Fibroblasts-M1 | purple |
| ZNF703 | Fibroblasts-M28 | skyblue |
| TCEA1P2 | Fibroblasts-M10 | blue |
| SNX14 | Fibroblasts-M16 | lightcyan |
| C6orf106 | Fibroblasts-M6 | turquoise |
| UBXN2B | Fibroblasts-M30 | saddlebrown |
| ARHGAP1 | Fibroblasts-M31 | darkred |
| GCLC | Fibroblasts-M6 | turquoise |
| RIN2 | Fibroblasts-M9 | pink |
| SLC41A2 | Fibroblasts-M13 | black |
| IL33 | Fibroblasts-M10 | blue |
| BACE2 | Fibroblasts-M30 | saddlebrown |
| WRNIP1 | Fibroblasts-M7 | cyan |
| LRRC75A | Fibroblasts-M10 | blue |
| GNPDA1 | Fibroblasts-M20 | red |
| EI24 | Fibroblasts-M27 | darkorange |
| POLR3H | Fibroblasts-M24 | midnightblue |
| ARHGEF6 | Fibroblasts-M2 | green |
| NFYB | Fibroblasts-M20 | red |
| MAP1S | Fibroblasts-M10 | blue |
| GALNT1 | Fibroblasts-M20 | red |
| ODF2 | Fibroblasts-M6 | turquoise |
| WBP1L | Fibroblasts-M15 | grey60 |
| ATG16L1 | Fibroblasts-M12 | steelblue |
| PMEPA1 | Fibroblasts-M12 | steelblue |
| RAI14 | Fibroblasts-M14 | tan |
| LYRM1 | Fibroblasts-M6 | turquoise |
| MRC1 | Fibroblasts-M24 | midnightblue |
| MRPS2 | Fibroblasts-M8 | magenta |
| S100A2 | Fibroblasts-M19 | orange |
| FBXO18 | Fibroblasts-M6 | turquoise |
| AFMID | Fibroblasts-M6 | turquoise |
| PCNX1 | Fibroblasts-M13 | black |
| NCF2 | Fibroblasts-M21 | brown |
| RFC5 | Fibroblasts-M2 | green |
| PPAT | Fibroblasts-M6 | turquoise |
| UBIAD1 | Fibroblasts-M13 | black |
| B4GALT5 | Fibroblasts-M5 | salmon |
| MGRN1 | Fibroblasts-M30 | saddlebrown |
| AL365357.1 | Fibroblasts-M10 | blue |
| REPIN1 | Fibroblasts-M6 | turquoise |
| RANBP6 | Fibroblasts-M2 | green |
| MSH6 | Fibroblasts-M4 | yellow |
| APEX2 | Fibroblasts-M13 | black |
| GPSM2 | Fibroblasts-M20 | red |
| NFE2L3 | Fibroblasts-M20 | red |
| HYOU1 | Fibroblasts-M5 | salmon |
| GEMIN7 | Fibroblasts-M6 | turquoise |
| EEF1B2P6 | Fibroblasts-M10 | blue |
| VPS25 | Fibroblasts-M21 | brown |
| RUVBL2 | Fibroblasts-M8 | magenta |
| NOP16 | Fibroblasts-M21 | brown |
| RNF6 | Fibroblasts-M26 | lightgreen |
| LYRM4 | Fibroblasts-M2 | green |
| EIF2D | Fibroblasts-M1 | purple |
| COMMD10 | Fibroblasts-M2 | green |
| ENTPD1 | Fibroblasts-M21 | brown |
| YIPF2 | Fibroblasts-M22 | paleturquoise |
| ZBED1 | Fibroblasts-M14 | tan |
| CHAF1A | Fibroblasts-M4 | yellow |
| QTRT2 | Fibroblasts-M21 | brown |
| MIF-AS1 | Fibroblasts-M10 | blue |
| KCTD3 | Fibroblasts-M5 | salmon |
| MT-TT | Fibroblasts-M10 | blue |
| UHRF2 | Fibroblasts-M14 | tan |
| FCGR3A | Fibroblasts-M21 | brown |
| KDELR3 | Fibroblasts-M14 | tan |
| TRPS1 | Fibroblasts-M9 | pink |
| SND1 | Fibroblasts-M14 | tan |
| BCL2L13 | Fibroblasts-M19 | orange |
| DHCR24 | Fibroblasts-M25 | greenyellow |
| RARRES1 | Fibroblasts-M7 | cyan |
| TRIQK | Fibroblasts-M8 | magenta |
| MYO15B | Fibroblasts-M15 | grey60 |
| POLR3K | Fibroblasts-M20 | red |
| NPM1P39 | Fibroblasts-M13 | black |
| AXIN2 | Fibroblasts-M7 | cyan |
| LPCAT3 | Fibroblasts-M9 | pink |
| PRR15L | Fibroblasts-M30 | saddlebrown |
| IPO8 | Fibroblasts-M9 | pink |
| SC5D | Fibroblasts-M30 | saddlebrown |
| NUP98 | Fibroblasts-M15 | grey60 |
| TMEM101 | Fibroblasts-M29 | darkgreen |
| FAM207A | Fibroblasts-M8 | magenta |
| SERPINF1 | Fibroblasts-M7 | cyan |
| HSD17B4 | Fibroblasts-M14 | tan |
| TRIB3 | Fibroblasts-M12 | steelblue |
| POLR3F | Fibroblasts-M25 | greenyellow |
| SAV1 | Fibroblasts-M21 | brown |
| LRRK2 | Fibroblasts-M10 | blue |
| MRGBP | Fibroblasts-M20 | red |
| PSPHP1 | Fibroblasts-M3 | lightyellow |
| CORO2A | Fibroblasts-M13 | black |
| LRRC57 | Fibroblasts-M13 | black |
| ZNF561 | Fibroblasts-M15 | grey60 |
| SH3BP2 | Fibroblasts-M21 | brown |
| CREB5 | Fibroblasts-M19 | orange |
| SEC24D | Fibroblasts-M9 | pink |
| GOLT1B | Fibroblasts-M14 | tan |
| TNFRSF21 | Fibroblasts-M6 | turquoise |
| RPS15AP11 | Fibroblasts-M10 | blue |
| RBM18 | Fibroblasts-M26 | lightgreen |
| TEAD1 | Fibroblasts-M15 | grey60 |
| HMGN4 | Fibroblasts-M1 | purple |
| FAM210B | Fibroblasts-M27 | darkorange |
| KLF7 | Fibroblasts-M13 | black |
| FBN1 | Fibroblasts-M7 | cyan |
| AC011446.2 | Fibroblasts-M10 | blue |
| NISCH | Fibroblasts-M5 | salmon |
| CDK9 | Fibroblasts-M6 | turquoise |
| PLPP3 | Fibroblasts-M10 | blue |
| SAMD1 | Fibroblasts-M19 | orange |
| TAF13 | Fibroblasts-M6 | turquoise |
| TMED7 | Fibroblasts-M6 | turquoise |
| ENAH | Fibroblasts-M9 | pink |
| TIMP2 | Fibroblasts-M9 | pink |
| GALNT6 | Fibroblasts-M3 | lightyellow |
| FKBP9 | Fibroblasts-M30 | saddlebrown |
| AUTS2 | Fibroblasts-M1 | purple |
| PIEZO1 | Fibroblasts-M1 | purple |
| CREG1 | Fibroblasts-M7 | cyan |
| RPIA | Fibroblasts-M7 | cyan |
| METTL2A | Fibroblasts-M20 | red |
| G6PD | Fibroblasts-M16 | lightcyan |
| GNPNAT1 | Fibroblasts-M13 | black |
| CENPQ | Fibroblasts-M16 | lightcyan |
| PDK4 | Fibroblasts-M25 | greenyellow |
| PATL1 | Fibroblasts-M17 | darkgrey |
| TARBP2 | Fibroblasts-M15 | grey60 |
| MARCO | Fibroblasts-M7 | cyan |
| BNIP3 | Fibroblasts-M13 | black |
| ANG | Fibroblasts-M17 | darkgrey |
| ADPRHL2 | Fibroblasts-M24 | midnightblue |
| DEFA6 | Fibroblasts-M10 | blue |
| B3GNT3 | Fibroblasts-M30 | saddlebrown |
| PITX1 | Fibroblasts-M10 | blue |
| METTL13 | Fibroblasts-M2 | green |
| HIST1H2BK | Fibroblasts-M1 | purple |
| SLC39A11 | Fibroblasts-M6 | turquoise |
| SBNO2 | Fibroblasts-M20 | red |
| DUSP3 | Fibroblasts-M6 | turquoise |
| ZDHHC9 | Fibroblasts-M6 | turquoise |
| CCNB2 | Fibroblasts-M4 | yellow |
| MTMR6 | Fibroblasts-M14 | tan |
| AC093591.1 | Fibroblasts-M10 | blue |
| ASCL2 | Fibroblasts-M25 | greenyellow |
| COL4A1 | Fibroblasts-M8 | magenta |
| DHTKD1 | Fibroblasts-M9 | pink |
| ESCO2 | Fibroblasts-M4 | yellow |
| NPIPA1 | Fibroblasts-M10 | blue |
| PKNOX1 | Fibroblasts-M17 | darkgrey |
| C6orf132 | Fibroblasts-M19 | orange |
| SH3PXD2B | Fibroblasts-M9 | pink |
| FBXO28 | Fibroblasts-M6 | turquoise |
| SDC2 | Fibroblasts-M8 | magenta |
| KLF11 | Fibroblasts-M6 | turquoise |
| PRMT5 | Fibroblasts-M6 | turquoise |
| PLBD2 | Fibroblasts-M7 | cyan |
| PSMD9 | Fibroblasts-M19 | orange |
| PARPBP | Fibroblasts-M4 | yellow |
| IL1R1 | Fibroblasts-M20 | red |
| MAP3K20 | Fibroblasts-M21 | brown |
| NOX1 | Fibroblasts-M20 | red |
| SERPINE1 | Fibroblasts-M26 | lightgreen |
| CRB3 | Fibroblasts-M5 | salmon |
| ITGA2 | Fibroblasts-M6 | turquoise |
| COL5A2 | Fibroblasts-M14 | tan |
| NEK6 | Fibroblasts-M2 | green |
| IGFBP2 | Fibroblasts-M31 | darkred |
| AP1S3 | Fibroblasts-M30 | saddlebrown |
| LAMC2 | Fibroblasts-M13 | black |
| MFSD14B | Fibroblasts-M5 | salmon |
| HNRNPA1P48 | Fibroblasts-M7 | cyan |
| CD276 | Fibroblasts-M31 | darkred |
| GK | Fibroblasts-M6 | turquoise |
| CCR1 | Fibroblasts-M13 | black |
| TTC7A | Fibroblasts-M6 | turquoise |
| AP1M2 | Fibroblasts-M21 | brown |
| ADAP2 | Fibroblasts-M21 | brown |
| LRCH1 | Fibroblasts-M15 | grey60 |
| SAAL1 | Fibroblasts-M2 | green |
| BCAT1 | Fibroblasts-M31 | darkred |
| MOB1B | Fibroblasts-M1 | purple |
| ADAM9 | Fibroblasts-M1 | purple |
| CASP2 | Fibroblasts-M17 | darkgrey |
| NRP1 | Fibroblasts-M21 | brown |
| ANLN | Fibroblasts-M4 | yellow |
| CD3EAP | Fibroblasts-M4 | yellow |
| MIPEP | Fibroblasts-M14 | tan |
| NUP205 | Fibroblasts-M5 | salmon |
| HSD3B7 | Fibroblasts-M11 | royalblue |
| SLC43A2 | Fibroblasts-M31 | darkred |
| TSFM | Fibroblasts-M19 | orange |
| FOSL1 | Fibroblasts-M13 | black |
| RPL5P34 | Fibroblasts-M10 | blue |
| BMP4 | Fibroblasts-M20 | red |
| NDUFV2 | Fibroblasts-M28 | skyblue |
| KCNE3 | Fibroblasts-M4 | yellow |
| GNB4 | Fibroblasts-M18 | white |
| HNRNPCP2 | Fibroblasts-M10 | blue |
| RPL22P1 | Fibroblasts-M10 | blue |
| PLXNC1 | Fibroblasts-M9 | pink |
| TSSC4 | Fibroblasts-M13 | black |
| SETP14 | Fibroblasts-M10 | blue |
| JPT2 | Fibroblasts-M4 | yellow |
| MANSC1 | Fibroblasts-M17 | darkgrey |
| SLC7A7 | Fibroblasts-M6 | turquoise |
| PLAU | Fibroblasts-M6 | turquoise |
| CCL18 | Fibroblasts-M25 | greenyellow |
| OAS2 | Fibroblasts-M21 | brown |
| CHST15 | Fibroblasts-M6 | turquoise |
| NDUFA7 | Fibroblasts-M25 | greenyellow |
| AARS | Fibroblasts-M29 | darkgreen |
| F3 | Fibroblasts-M6 | turquoise |
| EPHB3 | Fibroblasts-M30 | saddlebrown |
| THBD | Fibroblasts-M6 | turquoise |
| SNRPGP10 | Fibroblasts-M16 | lightcyan |
| TXNP6 | Fibroblasts-M10 | blue |
| SLC35F6 | Fibroblasts-M2 | green |
| RP2 | Fibroblasts-M6 | turquoise |
| RNF128 | Fibroblasts-M8 | magenta |
| ITGB5 | Fibroblasts-M9 | pink |
| UCK2 | Fibroblasts-M1 | purple |
| RAP2A | Fibroblasts-M21 | brown |
| PBK | Fibroblasts-M4 | yellow |
| CR381670.1 | Fibroblasts-M21 | brown |
| FAR2 | Fibroblasts-M21 | brown |
| SELENON | Fibroblasts-M14 | tan |
| FAM198B | Fibroblasts-M7 | cyan |
| TREM2 | Fibroblasts-M29 | darkgreen |
| INHBA | Fibroblasts-M14 | tan |
| RACGAP1 | Fibroblasts-M4 | yellow |
| ARRDC4 | Fibroblasts-M21 | brown |
| PLS3 | Fibroblasts-M8 | magenta |
| C5orf22 | Fibroblasts-M2 | green |
| STEAP1 | Fibroblasts-M7 | cyan |
| MSLN | Fibroblasts-M24 | midnightblue |
| UBL4A | Fibroblasts-M4 | yellow |
| AC026462.1 | Fibroblasts-M10 | blue |
| LAMC1 | Fibroblasts-M23 | darkturquoise |
| CD109 | Fibroblasts-M15 | grey60 |
| TMEM184B | Fibroblasts-M29 | darkgreen |
| KLK10 | Fibroblasts-M13 | black |
| AHNAK2 | Fibroblasts-M7 | cyan |
| CA9 | Fibroblasts-M26 | lightgreen |
| SERPINB8 | Fibroblasts-M6 | turquoise |
| FAIM2 | Fibroblasts-M10 | blue |
| TGM2 | Fibroblasts-M6 | turquoise |
| AC107983.1 | Fibroblasts-M24 | midnightblue |
| WWC2 | Fibroblasts-M31 | darkred |
| GREM1 | Fibroblasts-M14 | tan |
| SLC1A3 | Fibroblasts-M26 | lightgreen |
| POSTN | Fibroblasts-M14 | tan |
| KLK7 | Fibroblasts-M4 | yellow |
| CALB1 | Fibroblasts-M5 | salmon |

Supplementary Table 5: Summary of 5 colorectal cancer-associated fibroblast modules and their corresponding genes.

genes

| MMP2 |
| --- |
| CCDC80 |
| RARRES1 |
| PSAP |
| FBN1 |
| SERPINF1 |
| SERPING1 |
| GPNMB |
| COL14A1 |
| DAB2 |
| PPIC |
| MAPKAP1 |
| NPC2 |
| PBX3 |
| CFH |
| CD63 |
| PMP22 |
| LRP1 |
| SEMA3C |
| IGFBP4 |
| OAF |
| NUPR1 |
| SELENOP |
| STEAP1 |
| GRN |
| COL1A2 |
| COL3A1 |
| COL1A1 |
| COL6A3 |
| PCOLCE |
| VCAN |
| PLXDC2 |
| TIMP2 |
| ITGB5 |
| AEBP1 |
| COL5A1 |
| SULF2 |
| CASC4 |
| SEC31A |
| TTC3 |
| GOLM1 |
| GLG1 |
| MAGED1 |
| KIAA1217 |
| GALNT5 |
| SCARB2 |
| SERINC5 |
| SH3PXD2B |
| PAM |
| FNDC3B |
| POSTN |
| COL5A2 |
| SPARC |
| CALU |
| FN1 |
| BGN |
| INHBA |
| RAB31 |
| RAI14 |
| KDELR2 |
| GREM1 |
| HDLBP |
| KDELR3 |
| OSTC |
| ANXA5 |
| PALLD |
| TMEM167A |
| FAM114A1 |
| TRAM1 |
| S100A16 |
| VMP1 |
| GOLT1B |
| SSR3 |
| PPFIBP1 |
| GOLGA4 |
| HTRA1 |
| AHNAK |
| MARCKS |
| DST |
| CD109 |
| CYBRD1 |
| CLTC |
| MITF |
| NORAD |
| TEAD1 |
| ABCA1 |
| WBP1L |
| RB1CC1 |
| USP34 |
| AZI2 |
| CHD4 |
| HS2ST1 |
| PAFAH1B2 |
| DLG1 |
| GOLIM4 |
| AP1G1 |
| CUX1 |
| MAFB |
| IFI16 |
| LUM |
| DCN |
| CTSK |
| TMEM176B |
| C1S |
| TMEM176A |
| RARRES2 |
| C1R |
| PDPN |
| IL6ST |
| ZFP36L2 |
| BMP4 |
| LAMA2 |
| SMPDL3A |
| PRNP |
| CTSH |
| IL1R1 |
| LY6E |
| DRAM1 |
| ASPH |
| IQGAP2 |
| PLEKHA5 |
| ADAMDEC1 |
| TGFBR2 |
